# A molecular signature for delayed graft function

**DOI:** 10.1111/acel.12825

**Published:** 2018-08-09

**Authors:** Dagmara McGuinness, Suhaib Mohammed, Laura Monaghan, Paul A. Wilson, David B. Kingsmore, Oliver Shapter, Karen S. Stevenson, Shana M. Coley, Luke Devey, Robert B. Kirkpatrick, Paul G. Shiels

**Affiliations:** ^1^ Wolfson Wohl Translational Research Centre, Institute of Cancer Sciences, College of Medical, Veterinary & Life Sciences University of Glasgow Glasgow UK; ^2^ Computational Biology GlaxoSmithKline Medicines Research Centre Stevenage UK; ^3^ Renal Transplant Unit, NHS Greater Glasgow and Clyde South Glasgow University Hospital Glasgow UK; ^4^ Research Institute of Infection Immunity and Inflammation, College of Medical, Veterinary & Life Sciences University of Glasgow Glasgow UK; ^5^ Metabolic Pathways Cardio Therapy Area Unit GlaxoSmithKline King of Prussia Pennsylvania; ^6^ The Pipeline Futures Group GlaxoSmithKline Collegeville Pennsylvania; ^7^ Present address: Wellcome Centre for Molecular Parasitology, Institute of Infection, Immunity and Inflammation, College of Medical, Veterinary & Life Sciences University of Glasgow Glasgow UK; ^8^ Present address: The European Bioinformatics Institute (EMBL‐EBI) Saffron Walden UK; ^9^ Present address: Paul O'Gorman Leukaemia Research Centre Gartnavel General Hospital Glasgow UK

## Abstract

Chronic kidney disease and associated comorbidities (diabetes, cardiovascular diseases) manifest with an accelerated ageing phenotype, leading ultimately to organ failure and renal replacement therapy. This process can be modulated by epigenetic and environmental factors which promote loss of physiological function and resilience to stress earlier, linking biological age with adverse outcomes post‐transplantation including delayed graft function (DGF). The molecular features underpinning this have yet to be fully elucidated. We have determined a molecular signature for loss of resilience and impaired physiological function, via a synchronous genome, transcriptome and proteome snapshot, using human renal allografts as a source of healthy tissue as an in vivo model of ageing in humans. This comprises 42 specific transcripts, related through IFNγ signalling, which in allografts displaying clinically impaired physiological function (DGF) exhibited a greater magnitude of change in transcriptional amplitude and elevated expression of noncoding RNAs and pseudogenes, consistent with increased allostatic load. This was accompanied by increased DNA methylation within the promoter and intragenic regions of the DGF panel in preperfusion allografts with immediate graft function. Pathway analysis indicated that an inability to sufficiently resolve inflammatory responses was enabled by decreased resilience to stress and resulted in impaired physiological function in biologically older allografts. Cross‐comparison with publically available data sets for renal pathologies identified significant transcriptional commonality for over 20 DGF transcripts. Our data are clinically relevant and important, as they provide a clear molecular signature for the burden of “wear and tear” within the kidney and thus age‐related physiological capability and resilience.

**Abbreviations**
ABCA8: ATP‐binding cassette subfamily A member 8
ACKR3: atypical chemokine receptor 3
BTN3A2: butyrophilin subfamily 3 member A2
CCL19: C‐C motif chemokine ligand 19
CHGB: chromogranin B
CXCL10: C‐X‐C motif chemokine ligand 10
CYP17A1: cytochrome P450 family 17 subfamily A member 1
dsRNA: double‐stranded RNA
eIF2: eukaryotic initiation factor 2 signalling, protein ubiquitination
eIF4: eukaryotic initiation factor 4
FCGR1C: Fc fragment of IgG receptor Ic/CD64
FCGR2C: Fc fragment of IgG receptor IIc/CD32
FCRL3: Fc receptor‐like 3
GABBR1: gamma‐aminobutyric acid type B receptor subunit 1
HMGB1: high‐mobility group protein B1
HSPB7: heat‐shock protein family B (small) member 7
IFI44L: interferon‐induced protein 44‐like
ING: interferon gamma
KLRB1: killer cell lectin‐like receptor B1
LMO7: LIM domain 7
LPGAT1: lysophosphatidylglycerol acyltransferase 1
MMP9: matrix metallopeptidase 9
mTOR: mechanistic target of rapamycin
NLRC4: NLR family, CARD domain‐containing 4
NLRP2: NLR family, pyrin domain‐containing 2
OAS2: 2'‐5'‐oligoadenylate synthetase 2
p70Sk6: ribosomal protein S6 kinase beta‐1
PTPRC: protein tyrosine phosphatase, receptor type C
RAP1GAP: RAP1GTPase‐activating protein
REG1B: regenerating family member 1 beta
RRAGD: Ras‐related GTP‐binding D
SEMA3A: semaphorin 3A
SULT1E1: sulfotransferase family 1E member 1
TAGAP: T‐cell‐activation RhoGTPase‐activating protein
TLR4: Toll‐like receptor 4
TREM1: triggering receptor expressed on myeloid cells‐1
UBD: ubiquitin D
ZNF676: zinc finger protein 676

## 1 INTRODUCTION

The changing demographics of age in human society is anticipated to result in a major burden of age‐related morbidities, as improvements in health span have failed to match the increase in average global lifespan. Notably, deaths due to chronic kidney disease have increased globally, despite significant decline in other aetiologies (Christopher & Murray, 2015). This is pertinent to researchers investigating ageing, as accelerated cellular and physiological ageing are underlying components of renal dysfunction (Kooman, Kotanko, Schols, Shiels, & Stenvinkel, 2014; McGlynn et al., 2009; Schmitt, Susnik, & Melk, 2015), where systemic differences are layered on top of the dysregulated ageing process and patients show a higher incidence of mortality in comparison to healthy chronologically age‐matched individuals. As the prevalence of CKD parallels an increased prevalence in type 2 diabetes, obesity and a sedentary lifestyle (Stengel, Tarver‐Carr, Powe, Eberhardt, & Brancati, 2003), an allostatic outcome reflecting the “burden of life style” may be present next to the renal dysfunction.

Allostatic load can be defined as a composite indicator of accumulated biological stress over the life course, which predisposes to morbidity in the face of chronic or repeated stress exposure (Rubin, 2016). It is reflective of the biological age of a tissue organ or organism, as it directly impacts on age‐related physiological function.

We have previously developed the use of renal allografts as an in vivo model to study healthy tissue ageing in humans, whose physiological function can be tracked longitudinally to demonstrate that allograft biological age is more important than chronological age in prognostication of post‐transplant allograft performance (Gingell‐Littlejohn et al., 2013; McGuinness et al., 2016).

One testable prediction following this demonstration is that organs with increased biological age should reflect the cumulative burden of “wear and tear” and thus be less resilient to transplant‐related stresses and display reduced physiological function as a consequence. Assessing the related changes in molecular biology in these renal allografts is not straightforward (Shiels, McGuinness, Eriksson, Kooman, & Stenvinkel, 2017). To do so, we have used an analysis of a notable clinically relevant allograft phenotype displaying impaired physiological function, termed delayed graft function (DGF), to determine whether organs showing DGF are less resilient than those undergoing immediate graft function (IGF). Additionally, we have determined whether features associated with lack of physiological resilience were reflected in the pretransplant transcriptomes of respective organs.

A higher incidence of DGF has been associated with the use of allografts from older extended criteria donors (ECD, age >60, or >50 with two of the following: a history of high blood pressure, a creatinine ≥1.5, or death resulting from a stroke), donation after cardiac death donors (DCD) and increased allograft biological age (Mallon, Summers, Bradley, & Pettigrew, 2015; McGuinness et al., 2016; Menke, Sollinger, Schamberger, Heemann, & Lutz, 2014; Mundt, Yard, Kramer, Benck, & Schnulle, 2015; Schroppel & Legendre, 2014). The extent to which donor and recipient‐related characteristics influence the magnitude of IRI and/or DGF occurrence, beyond accepted clinical risk factors for DGF, remains to be proven (Menke et al., 2014; Mundt et al., 2015; Schroppel & Legendre, 2014), particularly in the context of allograft repair, or regeneration pathways, activated in response to IRI. Increased demand for organ donation, coupled with increasing chronological age and associated comorbidities in the donor population, has necessitated the use of organs that have been previously deemed as marginal for clinical use (Morrissey & Monaco, 2014; Nagaraja et al., 2015).

**Figure 1:**
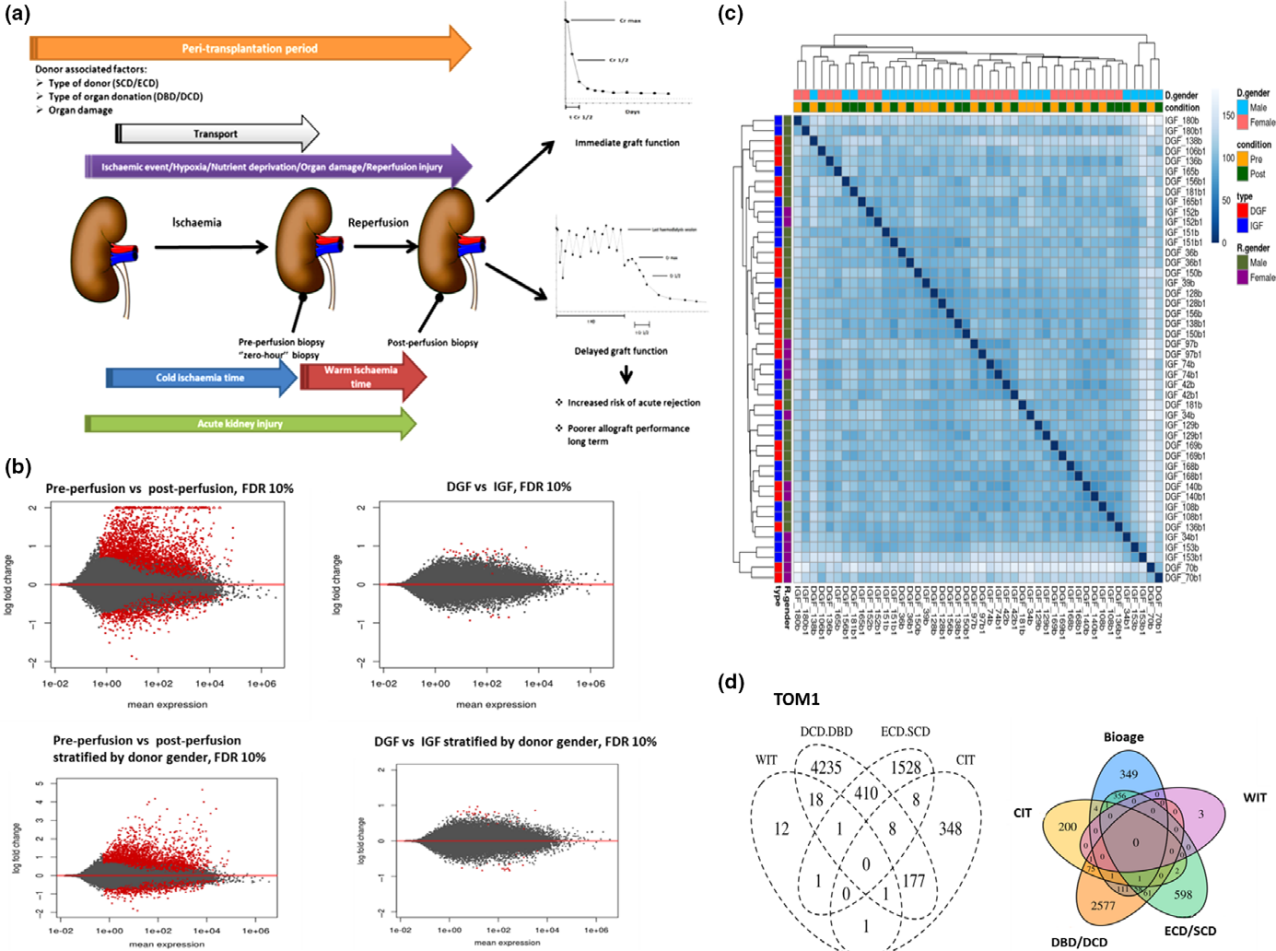
Description of samples with DGF and IGF occurrence selected for RNAseq. (a) Schematic representation of peritransplantation period and its relation to DGF and IGF (b) MA plots representing significantly differential gene expression between the two experimental groups (preperfusion (B) vs. postperfusion (B1) samples and DGF vs. IGF) presented as log2‐fold changes against mean gene expression alone or stratified by donor gender. Red dots represent genes showing significantly different expression (FDR < 0.1). (c) Heatmap representing hierarchical clustering of samples using Euclidean distances calculated from regularized log2 transformation to visualize samples with similar or dissimilar characteristics in relation to analysed outcomes and perfusion status. (d) Gene signatures associated with DGF risk factors analysed in the context of the whole transcriptome (TOM1, left panel) and after adjustment for BioAge (right panel). DBD: donation after brain death; DCD: donation after cardiac death; ECD: extended criteria donor; SCD: standard criteria donor; CIT: cold ischaemia time; WIT: warm ischaemia time (anastomosis time); R.gender: recipient gender; D.gender: donor gender

DGF has also proven refractory to modelling both in vitro and in preclinical model organism studies. This study aimed to identify a human‐specific molecular signature associated with DGF and to enable adjustment for the effects of IRI‐related molecular changes, in the absence of model systems for analysis of DGF mechanisms, as well as providing direct insight into its manifestation. Additionally, this strategy was designed, to enable the validation of any DGF‐associated signature, by comparison with existing publically available renal data sets, in order to elucidate whether there were common underpinning molecular processes in their manifestation and whether these reflected the burden of “wear and tear” in the kidney. We selected a very closely matched clinical cohort based on age, gender, length of ischaemic time and low HLA mismatch. These were divided into two groups based on recovery of organ function after transplantation, with emphasis on extreme functional differences, namely either DGF or IGF (Table1). An extreme DGF phenotype was defined as (a) the need for dialysis within 7 days of transplantation, with the exception of hyperkalaemia on the first postoperative day, and (b) the failure of serum creatinine to reduce by 50% within the first week, which is indicative of poor recovery of renal function (Figure 1a; Aitken et al., 2015). IGF was defined as reduction in serum creatinine by 50% in less than 3 days post‐transplantation. Additionally, we undertook a retrospective analysis of paired allograft biopsies from this cohort, obtained at two time points: preimplantation during the preparatory phase (preperfusion) and after allograft reperfusion when circulation was restored (postperfusion).

**Table 1:** Patient clinical and experimental characteristics for the RNAseq cohort (top panel) and combined cohort (bottom panel)

RNAseq cohort (*n* = 23)	DGF (*n* = 11)		IGF (*n* = 12)	
Variable	Mean (Min‐Max)/Proportion	Standard deviation (if applicable)	Mean (Min‐Max)/Proportion	Standard deviation (if applicable)
Donor gender (males/females)	6/5		5/7	
Donor age (years)	55 (40–74)	17.6	53.2 (40–77)	10.7
Donor serum creatinine at retrieval (µmol/L)	73.25 (44–125)	26.6	78.64 (41–125)	25.6
Donor type				
DBD/DCD	7/4		9/3	
ECD	6		4	
DBD‐ECD	4		3	
DCD‐ECD	2		1	
Cause of death				
Intracranial haemorrhage	7		8	
Hypoxic brain injury	0		2	
Trauma	0		2	
Cardiac arrest	1		0	
Intracranial thrombus	1		0	
Respiratory failure	1		0	
Meningitis	1		0	
Recipient gender (males/females)	8/3		8/4	
Recipient age (years)	57.9 (42–72)	10.1	51.1 (35–70)	10.1
Previous transplantation	0		0	
HLA Mismatch				
HLA‐A (0/1/2)	4/5/2		4/6/1	
HLA‐B (0/1/2)	2/9/0		3/7/1	
HLA‐DR (0/1/2)	4/7/0		3/7/0	
Cold ischaemic time (hr)	12.1 (9–17)	2.8	11.1 (6–20)	4.5
Warm ischaemic time (min)	29.3 (21–40)	6.1	31.3 (22–40)	6.7
T1/2	from 4 to 21 days		less than 3 days	
Serum creatinine level at 6 months (µmol/L)	126.7 (89–158)	2.1	104.4 (84–166)	1.74

Combined patient cohort (*N* = 55)	Mean (Min‐Max)/Proportion	Standard deviation (if applicable)
Donor gender (males/females)	33/22	
Donor age (years)	50.5 (11–77)	16.4
Donor serum creatinine at retrieval (µmol/L)	87.1 (41–195)	35.7
Ethnicity (Caucasian: Asian)	54:1	
Donor type		
DBD/DCD	37/11	
ECD	34	
DBD‐ECD	16	
DCD‐ECD	5	
Cause of death		
Intracranial haemorrhage	35	
Hypoxic brain injury	10	
Trauma	7	
Cardiac arrest	1	
Intracranial thrombus	1	
Respiratory failure	1	
Meningitis	0	
Donor hypotension (*N*)	39	
Donor hypertension (*N*)	23	
Recipient gender (males/females)	32/23	
Recipient age (years)	54.2 (27–73)	10.1
Aetiology of Renal Failure		
IgA nephropathy	9	
Glomerulonephritis	6	
APKD	12	
Reflux/obstructive uropathy	7	
Hypertension	4	
Diabetes mellitus	2	
Unknown	8	
Goodpasture's syndrome	1	
Anatomical anomalies	2	
Drug related	1	
Stone disease	1	
HUS	1	
FSGS	1	
Previous Transplant		
0	45	
1	7	
2	2	
HLA Mismatch		
HLA‐A (0/1/2)	9/18/10	
HLA‐B (0/1/2)	10/23/5	
HLA‐DR (0/1/2)	24/10/0	
Cold ischaemic time (hr)	12.8 (6–21)	3.9
Immunosuppression		
Induction		
Basiliximab/campath/ATG	42/6/6	
Maintenance		
Tacrolimus/Sirol/Cyc	54/0/1	
Prednisolone	51	
Mycophenolatemofetil	55	
BPAR	1	
DGF	19	
Serum creatinine level at 6 months (µmol/L)	131 (61–523)	74.4
MDRD4 at 6 months (ml min^−1^ 1.73 m^−2^)	57.4 (9–102)	22.3
Serum creatinine level at 12 months (µmol/L)	137.6 (67–377)	62.8
MDRD4 at 12 months (ml min^−1^ 1.73 m^−2^)	51.1 (10–89)	20.8

Note

Continuous variables are expressed as mean with standard deviation, whereas categorical variables are expressed as proportions.

We have used this approach to test the hypothesis that DGF is a manifestation of organ “wear and tear” (i.e. its allostatic load as a function of its biological age) and that the impaired physiological capability can be defined using a specific set of molecular features, independently of allograft damage acquired during the peritransplantation period.

## 2 RESULTS

### 2.1 RNAseq cohort characteristics are related to differences associated with DGF and response to reperfusion injury

Differential gene expression associated with perfusion, or DGF status, was assessed alone, or stratified by donor gender (Figure 1b). Significant variation (28%) was observed between donor genders. This was more pronounced than the pre‐/postperfusion or DGF/IGF variance, each of which grouped into distinct gender clusters (Supporting Information Figure S1 in Data S1). The magnitude of change in pre‐ versus postperfusion biopsies was more pronounced than in a comparison of DGF versus IGF biopsies (Figure1b, Supporting Information Figure S2 in Data S1). Hierarchical clustering of the samples is presented in Figure 1c. 3,893 of 4,052 transcripts were differentially expressed between preperfusion and postperfusion samples after adjustment for donor gender. Fifty‐five transcripts correlated uniquely with DGF outcome, and only six of these were significantly different between DGF and IGF after adjustment for donor gender: REG1B, GABBR1, UBD, DAZ1, ABCA7 and BTN3A2.

Comparison of whole transcriptional profiles (TOM1) with different clinical risk factors for DGF including ECD/SCD, DCD/DBD, cold (CIT) and warm ischaemia time (WIT) did not reveal any overt transcriptional changes. However, each factor was independently associated with unique transcript expression (Figure 1d). CDKN2A/p16^ink4a^ expression correlated with DGF occurrence and long‐term allograft function post‐transplantation, when used as a composite BioAge (pretransplant donor risk classification system using CDKN2A and ECD criteria) (Gingell‐Littlejohn et al., 2013; McGuinness et al., 2016). No common targets were associated with BioAge and DGF risk factors. Eight hundred and eighty‐one transcripts were differentially expressed between samples with low versus high CDKN2A/p16^ink4a^ expression (below/above median for sequenced sample set) with 349 being unique for BioAge. Stratification by BioAge revealed 58 common targets for DBD/DCD, ECD/SCD and BioAge (Figure 1d).

Further analysis, in conjunction with (a) perfusion status changes (preperfusion vs. postperfusion, TOM2) and (b) differences between DGF and IGF transcriptomes (TOM3), established a molecular signature for DGF adjusted for the effect of reperfusion (DGF‐specific signature; TOM4).

**Figure 2:**
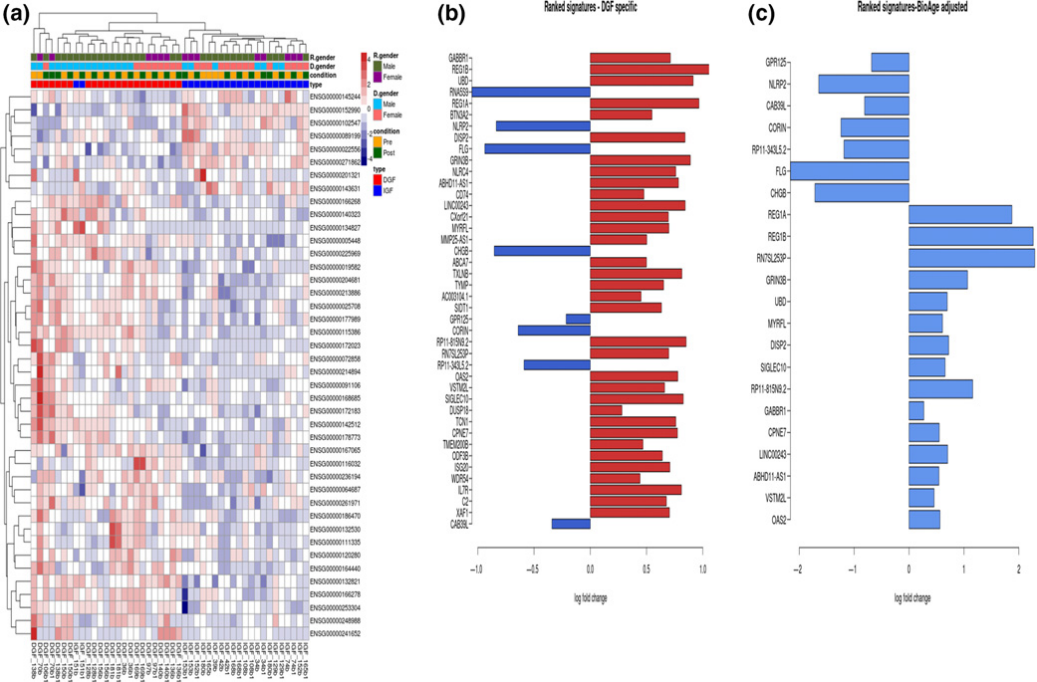
Gene signatures associated with DGF after adjustment for ischaemia reperfusion injury (TOM4) presented as heatmaps, left panel (adjusted *p*‐value < 0.05). The expression counts were normalized by regularized log2 transformation. The phenotypic attributes associated with samples are mapped at the top of the plot. Preperfusion samples (B), postperfusion samples (B1). The right panel consists of top ranked DGF‐specific genes (adjusted *p* < 0.05) and the left panel consists of DGF signature after adjustment for BioAge. Two samples were excluded from the further analysis as they did not pass QA and QC control before bioinformatics analysis (106B and 39B1). R.gender: recipient gender; D.gender: donor gender; DGF: delayed graft function; IGF: immediate graft function

Forty‐nine transcripts were identified as markers of DGF (TOM3). However, adjustment for perfusion status (TOM4) reduced this to 42 differentially expressed transcripts (Supporting Information Table S1 in Data S2, Figure 2). DGF outcome and donor gender were related to the DGF‐specific signature, with male and female donors forming distinct clusters indicative of a donor gender‐driven DGF phenotype (Figure 2a). Further analysis of DGF‐specific transcripts revealed that overall expression changes in response to reperfusion occurred along a similar trajectory in both DGF and IGF, but the magnitude of this change was greater for those exhibiting DGF. This suggests that the degree of response to reperfusion injury is significant in post‐transplant outcome (Supporting Information Figure S2 in Data S1). Further analysis of the transcriptome for DGF‐specific signatures independent of IRI, but stratified by BioAge, revealed the presence of only 22 DGF‐specific targets (Figure 2b). Analysis of the DGF signature across the age groups (<50; 50–60 and >60) revealed significant changes for NLRC4, IL7R and GRIN3B.

Additional analyses, involving comparison of the allograft response to IRI (both for DGF and IGF status), using the respective RNAseq data sets, with publically available data sets for other renal pathologies, were undertaken to identify additional transcripts. These were included in further validation testing (Supporting Information Datas S1 and S3).

### 2.2 DGF, immune response generation and senescence pathways

Initial analysis of the effect of perfusion status on the pathways associated with senescence revealed 22 transcripts that had been significantly affected, these included BMI1, CDK2, CDKN1A, ETS2, RB1, RBL2, p53/Rb signalling (CDKN2B, CITED2, ING1, MYC, PCNA, PIK3C, SERPINE1, SIRT1, SPARC), interferon‐related (CDKN1A, EGR1, INFG, IRF7, RB1), IGF1, MAP2K3, PCNA, as well as cell adhesion molecules affected by senescence (COL1A1, COL3A1).

Ingenuity^®^Pathway Analysis (IPA^®^, QIAGEN Redwood City; USA) indicated that DGF was allied with activation of innate immune responses, including GABA signalling, TREM1 signalling, pattern recognition receptors and B‐cell development (Supporting Information Tables S2–S5 in Data S2). The top ranked networks comprised immune system activation, cell death/survival, cellular fitness and cell–cell communication, renal and urological system development and function. An upstream regulator analysis algorithm also identified activation of the immune system and cell‐mediated immune responses in the development of DGF. The main “drivers” within these processes, typically related to the response to pathogens (bacterial and viral), or sterile inflammation and induction of the secondary INF‐γ‐associated responses to dsRNA, which have been linked to mechanisms underpinning autoimmune diseases (Brencicova & Diebold, 2013; Nellimarla & Mossman, 2014; Pollard, Cauvi, Toomey, Morris, & Kono, 2013) and cellular ageing (Gorbunova, Boeke, Helfand, & Sedivy, 2014).

Further analysis of our data sets, either individually, or in direct comparison with publicly available data sets, demonstrated overt links to inflammatory responses, inherent in renal pathologies, and highlighted the importance of interaction between lymphoid and nonlymphoid cells in the context of renal function (Supporting Information Figures S1–S4 in Data S3). Pathway analysis of differentially expressed targets relative to perfusion status highlighted pathways overlapping with processes inherent in ageing, including eIF2 (eukaryotic initiation factor 2) signalling, protein ubiquitination, eukaryotic initiation factor 4 (eIF4) and ribosomal protein S6 kinase beta‐1 (p70Sk6) signalling and interleukin‐17 (IL‐17)‐mediated cytokine regulation (Supporting Information Tables S1–S3 in Data S5).

These data suggest that DGF outcome may be related to a differential capacity to restore physiological homeostasis following IRI. Further analysis of an additional model (DGF pre‐ vs. postperfusion and IGF pre‐ vs. postperfusion, Model 2) revealed that the transcriptional response to reperfusion injury was similar for allografts, irrespective of their post‐transplant outcome. Resolution of transplant‐related stresses and restoration of physiological homeostasis were, however, exacerbated in those that manifested DGF (Supporting Information Tables S5–S10 in Data S5) suggesting, that donor‐organ resilience to stress may be a key determinant of DGF, which is congruent with recent findings linking organ function to biological age (Gingell‐Littlejohn et al., 2013; McGuinness et al., 2016). Significantly, this is supported by the observation that the transcriptional amplitude of change in DGF signature genes following reperfusion was significantly larger than in IGF. This is consistent with there being a greater degree of allostatic load in organs developing DGF and an inability to restore transcriptional and physiological processes to function within normal physiological parameters as quickly as organs with IGF.

### 2.3 Epigenetic status is linked to DGF and perfusion status

The effect of IRI on epigenetic status associated with DGF outcome was analysed in connection with changes in global DNA methylation and frequency of alternative splicing (AS) events. Alternative splicing is a common post‐transcriptional modification that enables cells to increase protein diversity from a single copy of a gene by generation of unique coding transcripts or regulatory noncoding RNAs. These can be affected by changes in DNA methylation status and GC content at the intron–exon boundaries, ultimately affecting both splicing outcomes, alternative splicing networks and affecting writing and/or maintenance of epigenetic marks and changes in chromatin status (Francisco & Baralle, 2017; Shiran Naftelberg, Ast, & Kornblihtt, 2015). Transcripts associated with chromatin remodelling were significantly affected by the perfusion status in our cohort and included polycomb group genes (BMI1, SUZ12, TRIM27); chromobox/HP1 homologs (CBX4, CBX8); bromodomain proteins (BRD2, WDR11); ING family members (ING1, INg2, ING4) and PHF21B.

DGF‐specific transcripts revealed differential promoter methylation status dependent upon perfusion state and DGF occurrence. An increase in DNA methylation within the promoter and intragenic regions of the respective DGF‐associated genes was observed for IGF compared to DGF in preperfusion samples. This relationship was lost, or reversed after reperfusion, suggesting that reperfusion can directly and immediately affect epigenetic status in tissues. These observations are supported by animal model studies where 30‐min IRI was sufficient to affect global DNA methylation levels (Endres et al., 2000; Meller, Pearson, & Simon, 2015).

**Figure 3:**
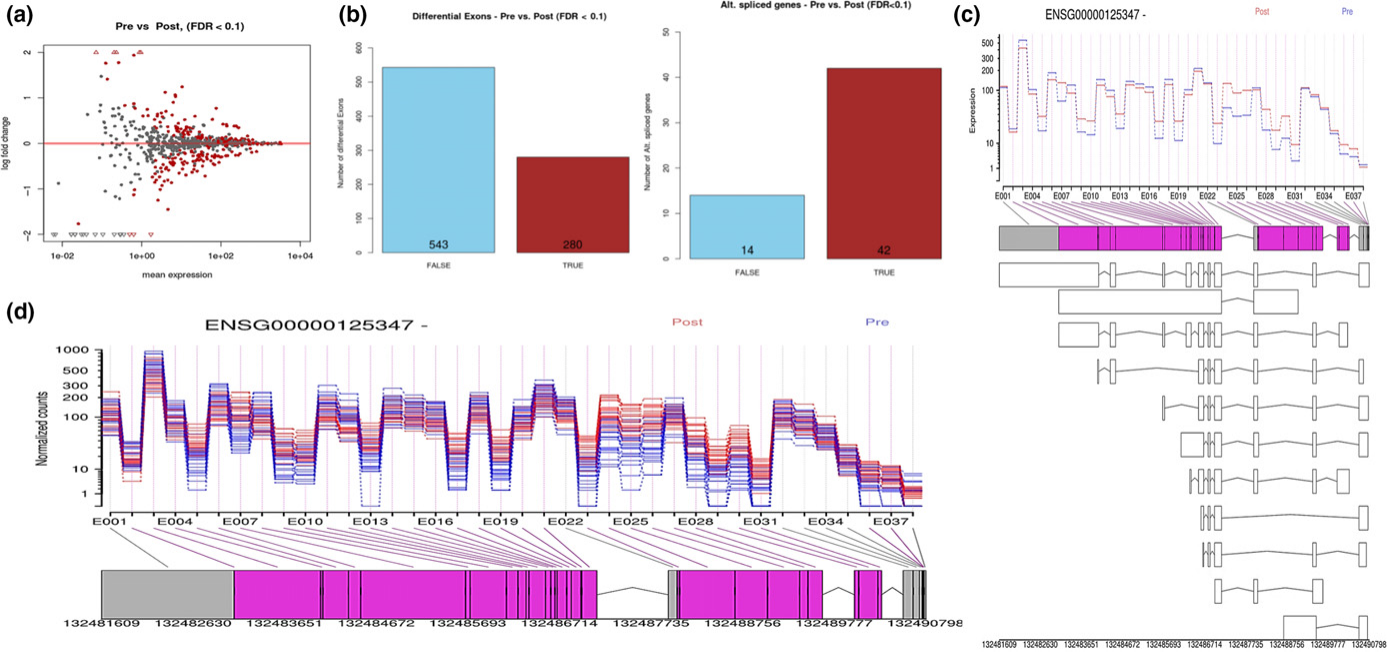
Alternative splicing is associated with reperfusion injury. (a) MA plot representing differential exon expression between preperfusion versus postperfusion samples. (b) Number of differentially expressed exons between perfusion states and number of alternatively spliced genes selected from top 100 targets differentially expressed between perfusion states. (c) Differential exon expression in relation to perfusion state for IRF1. (d) Hypothetical alternative transcript predicted for IRF1 (interferon regulatory factor 1).

A representative comparison between epigenetic status, RNAseq and qPCR data is summarized in Supporting Information Figures S1 and S2 in Data S4. Interestingly, an increased incidence of AS events was associated with reperfusion injury, but not DGF occurrence. No AS events were detected in the DGF‐specific transcript set. The top 100 differentially expressed transcripts between perfusion states were analysed, with only 42 displaying AS events, as indicated by differential exon expression (Figure 3a,b, Supporting Information Data S6). A representative example of an AS event has been illustrated using interferon regulatory factor 1 (IRF1) (Figure 3c,d). This is suggestive of a complex relationship between methylation status and alternative splicing associated with IRI injury that is not observed in organs displaying DGF.

### 2.4 Validation of DGF‐specific transcripts

Transcripts related to DGF, selected based on their ranking by statistical significance of observed expression change and diversity of signalling pathway involvement in relation to publically available data sets, were further validated in 19 paired biopsies from the RNAseq cohort and an independent cohort of 32 pairs of samples. Three samples were excluded from further analysis due to post‐transplant complications. Nineteen genes were validated as markers of DGF.

Transcripts were further analysed in relation to donor characteristics, ischaemic time and estimated glomerular filtration rate (eGFR/MDRD4), as a measure of physiological function, at 3, 6 and 12 months post‐transplantation (Table 2). Interestingly, most of the DGF‐specific transcripts that correlated positively with donor age were negatively correlated with allograft performance after transplantation, including PTPRC, SEMA3A, KLRB1, CD52, CCL19, LNRC4, GABBR1 and UBD. KLRB1 was a common denominator for DGF, ECD status, DCD status and allograft performance, while SEMA3A was differentially expressed in relation to DGF, ECD status; Cr T1/2 and allograft function post‐transplant. FCGR1C was related to DGF outcome, DCD status and allograft function.

**Table 2:** Summary of validated DGF‐specific targets in the RNAseq cohort, the validation cohort (directionality of changes indicated by arrows)

RNAseq cohort (IGF = 11, extreme DGF = 8)	Validation cohort (IGF = 7, DGF = 9)	Combined cohort (Overall DGF = 17; no DGF = 34)	DCD vs. DBD	ECD vs. SCD	Correlations (correlation coefficient; *p*‐value)						
					Donor age	MDRD4 at 3 months	MDRD4 at 6 months	MDRD4 at 12 months	CIT	WIT	Cr T1/2
ACKR3 Pre (*p* = 0.02)↓	C1QB Pre (*p* = 0.029)	ABCA8 Pre (*p* = 0.017)↑	REG1A (*p* = 0.026)↓	CXCL9 Pre (*p* = 0.009)↑	TAGAP Pre (0.46; 0.001)	PTPRC Pre (−0.35; 0.000)	PTPRC Pre (−0.45; 0.001)	GABBR1 Pre (−0.35; 0.028)	ACKR3 Pre (0.33; 0.030)	GABBR1 Post (−0.32; 0.031)	SEMA3A Post (0.33; 0.025)
BTN3A2 Post (*p* = 0.041)↑	FCGR1C Post (*p* = 0.031) ↑	ACKR3 Pre (*p* = 0.007)↓	CHGB Post (*p* = 0.015)↓	UBD pre (*p* = 0.036)↑	PTPRC Pre (0.5; 0.000)	PTPRC Post (−0.34; 0.014)	PTPRC Post (−0.34; 0.020)	ACKR3 Post (−0.37; 0.019)	CHGB Pre (0.35; 0.010)	CORIN Post (−0.29; 0.042)	CHGB Post (−0.27; 0.047)
CCL19 Post (*p* = 0.041)↑	FCGR1C Pre (*p* = 0.001)↑	BTN3A2 Post (*p* = 0.038)↑	CHGB Pre (*p* = 0.021)↓	TAGAP Pre (*p* = 0.001)↑	NLRC4 Pre (0.32; 0.024)	NLRC4 Pre (−0.34; 0.014)	NLRC4 Pre (−0.48; 0.000)	ZNF676 Post (−0.31; 0.043)	REG1B Pre (0.38; 0.006)		
CD52 Pre (*p* = 0.021)↑	KLRB1 Pre (*p* = 0.020) ↑	CD52 Pre (*p* = 0.029)↑	CORIN Post (*p* = 0.001)↓	PTPRC Pre (*p* = 0.001)↑	KLRB1 Pre (0.36; 0.010)	KLRB1 Pre (−0.30; 0.033)	KLRB1 Pre (−0.41; 0.003)				
CHGB Post (*p* = 0.008)↓	NLRC4 Pre (*p* = 0.043)↑	CHGB Post (*p* = 0.012)↓	REG1B Post (*p* = 0.026)↓	KLRB1 Pre (*p* = 0.028) ↑	CD52 Pre (0.38; 0.007)	KLRB1 Post (−0.28; 0.045)	KLRB1 Post (−0.31; 0.023)				
CHGB Pre (*p* = 0.01)↓	OAS2 Post (*p* = 0.045)↑	CXCL10 Post (*p* = 0.039)↑	FCGR1C Pre (*p* = 0.001)↑	CD69 Pre (*p* = 0.008) ↑	CCL19 Pre (0.34; 0.014)	CD52 Pre (−0.32; 0.024)	CD52 Pre (−0.32; 0.024)				
CXCL10 Post (*p* = 0.015)↑	PTPRC Post (*p* = 0.029)↑	FAM34A Pre (*p* = 0.012)↓	TRIM Pre (*p* = 0.031)↑	CD52 Pre (*p* = 0.014) ↑	SEMA3A Pre (0.41; 0.007)	CCL19 Post (−0.34; 0.014)	CCL19 Post (−0.32; 0.021)				
CORIN Pre (*p* = 0.017)↓	PTPRC Pre (*p* = 0.020)↑	FCGR1C Post (*p* = 0.020)↑	TRIM Post (*p* = 0.003)↑	CCL19 Pre (*p* = 0.026) ↑	SEMA3A Post (0.33; 0.028)	CHGB Pre (−0.274; 0.043)	SEMA3A Pre (0.43; 0.003)				
DISP2 Post (*p* = 0.013)↑	SEMA3A Post (*p* = 0.018)↑	FCGR1C Pre (*p* = 0.009) ↑	RSPO1 Pre (*p* = 0.013)↑	C1QB Pre (*p* = 0.033) ↑	UBD Pre (0.32; 0.020)	SEMA3A Post (−0.36; 0.016)	SEMA3A Post (−0.36; 0.014)				
DISP2 Pre (*p* = 0.02)↑		GABBR1 Post (*p* = 0.037)↑	KLRB1 Post (*p* = 0.007)↓	ISG20 Pre (*p* = 0.045)↑	FCGR1C Pre (0.2; 0.022)	GABBR1 Post (−0.32; 0.031)	NLRP2 Post (−0.30; 0.041)				
FAM34A Pre (*p* = 0.043)↓		KLRB1 Pre (*p* = 0.026)↑	FAM34A Pre (*p* = 0.028)↓	LLCIOB Post (*p* = 0.040)↑			GABBR1 Pre (−0.33; 0.028)				
FCGR1C Post (*p* = 0.041)↑		NLRC4 Pre (*p* = 0.018)↑	CXCL10 Pre (*p* = 0.03)↑				GABBR1 Post (−0.34; 0.019)				
FCGR2C Post (*p* = 0.033)↑		NLRP2 Pre (*p* = 0.025)↓	ABCA8 Pre (*p* = 0.001)↑				CORIN Post (−0.32; 0.018)				
FCRL3 Pre (*p* = 0.013)↓		OAS2 Post (*p* = 0.017)↑	ABCA8 Post (*p* = 0.026)↑				FCGR2C Pre (−0.33; 0.017)				
FLG Pre (*p* = 0.042)↓		PTPRC Pre (*p* = 0.014)↑	BTN3A2 Pre (*p* = 0.019)↑				FCGR1C Pre (−0.32; 0.018)				
KLRB1 Pre (*p* = 0.026)↑		SEMA3A Post (*p* = 0.002)↑	BTN3A2 Post (*p* = 0.04)↑								
OAS Post (*p* = 0.027) ↑		TAGAP Post (*p* = 0.01)↑	OAS2 Pre (*p* = 0.000)↑								
REG1B Post (*p* = 0.043)↑		ZNF676 Pre (*p* = 0.032)↓	OAS2 Post (*p* = 0.006)↑								
REG1B Pre (*p* = 0.034)↑			ISG20 Pre (*p* = 0.045)↓								
NLRP2 Pre (*p* = 0.013)↓			ISG20 Post (*p* = 0.031)↓								
UBD Post (*p* = 0.006)↑			INFG Post (*p* = 0.032)↑								
UBD Pre (*p* = 0.013)↑			LLCIOB Pre (*p* = 0.002)↓								
SEMA3A Post (*p* = 0.004)↑			LLCIOB Post (*p* = 0.014)↓								
TAGAP Post (*p* = 0.021)↑											
ZNF676 Pre (*p* < 0.0001)↓											

Notes

The Mann–Whitney *U* test was used to compare variables in relation to different categories analysed. The FDR adjustment for multiple comparisons was performed and all targets retained significance (*p* < 0.05). Spearman correlation was used to analyse DGF‐specific targets with donor age, CIT, WIT, Cr T1/2 and MDRD4 at 3, 6 and 12 months post‐transplant. The table presents unadjusted *p*‐values.

CIT: cold ischaemic time; Cr T1/2: time of reduction in serum creatinine by 50%; DBD: donation after brain death; DCD: donation after cardiac death; ECD: extended criteria donor; IGF: immediate graft function no DGF includes allografts with primary function; MDRD4: estimated glomerular filtration rate modification of diet in renal disease (MDRD) equation; SCD: standard criteria donor; WIT: warm ischaemic time.

Additionally, RNAscope was performed on 18 preperfusion biopsies from the RNAseq cohort for which tissue sections were available, to confirm the presence of five selected transcripts namely CD45, INFγ, ACKR3, SEMA3A, REG1B and PPIB as a positive control.

### 2.5 Validation of DGF gene expression at the protein level

The expression of DGF‐specific genes at the protein level was undertaken using western blotting in a subset of samples to determine whether changes at the protein level could be related to DGF and/or reperfusion injury (Supporting Information Data S7). Samples were isolated sequentially from the same tissue specimen after RNA and genomic DNA isolation and equal amount of total protein was used in the further analysis. Eight DGF‐associated proteins were detected. Four (FCGR1C, FCGR2C, CD52 and PTPRC) were not detectable. Five genes associated with DGF were further validated in the tissue biopsies to verify both their expression and localization at the protein level in 18 preperfusion biopsies. Morphological and histological analyses of these biopsies were undertaken for 17 histological variables associated with renal pathology (including CKD and AKI) and assessed using several published histological scoring systems for kidney quality (Maryland Aggregate Pathology Index, Chronic Allograft Damage Index, Banff Score and Remuzzi Score). These revealed no obvious correlation between overall quality score and the presence or absence of DGF. The expression of CD45, IFNγ, REG1B or SEMA3A showed no significant differences in signal intensity or location between biopsies from donor kidneys that would experience IGF compared with those demonstrating DGF. ACKR3 expression was negative overall with the exception of two samples.

### 2.6 DGF and perfusion status are associated with cellular senescence

The expression of the CDKN2 locus in relation to the DGF outcome and perfusion status at the transcript level, including CDKN2A/p16^INK4^, ARF/p14, CDKN2B, as well as other senescence‐associated markers (TP53, CDKN1A/p21 and CDKN1B/p27) was determined.

Significantly, higher expression of CDKN2B/p15 and CDKN1A was noted in postperfusion biopsies compared to the preperfusion biopsies (*p* = 0.0003 and *p* < 0.0001, respectively).

Furthermore, a positive relationship between CDKN2A/p16^INK4^ and TP53 in preperfusion biopsies (cc = 0.305; *p* = 0.039) was observed; this correlation was lost after reperfusion. CDKN2A/p16^INK4^ was positively correlated with ARF/p14 (cc = 0.48, *p* = 0.000) and CDKN2B/p15 (cc = 0.405, *p* = 0.004) in preperfusion biopsies, whereas ARF/p14 was correlated with CDKN2B (cc = 0.397, *p* = 0.004). CDKN2A/p16^INK4^ was correlated with donor age (cc = 0.489; *p* = 0.000 and cc = 0.419, *p* = 0.002) and MDRD4 at 3 months (cc = −0.473; *p* = 0.001 and cc = −0.310, *p* = 0.030) and 6 months (cc = −0.471; *p* = 0.001 and cc = −0.397, *p* = 0.005) post‐transplant regardless of the perfusion status. However, only preperfusion CDKN2A/p16^INK4^ was negatively correlated with MDRD4 (cc = −0.393, *p* = 0.009). Interestingly, preperfusion TP53 expression was correlated with CIT (cc = 0.406, *p* = 0.004), whereas postperfusion TP53 expression was associated with MDRD4 at 12 months (cc = −0.378; *p* = 0.014).

The expression of preperfusion CDKN1A was positively correlated with CIT (cc = 0.347, *p* = 0.017), whereas serum creatinine at 12 months post‐transplant was positively correlated with postperfusion expression of CDKN1B (cc = 0.333, *p* = 0.033) and CDKN1A (cc = 0.344, *p* = 0.022). Additionally, preperfusion expression of CDKN1B was associated with serum creatinine (cc = 0.432, *p* = 0.003) and MDRD4 (cc = −0.346, *p* = 0.020) at 6 months post‐transplant.

**Figure 4:**
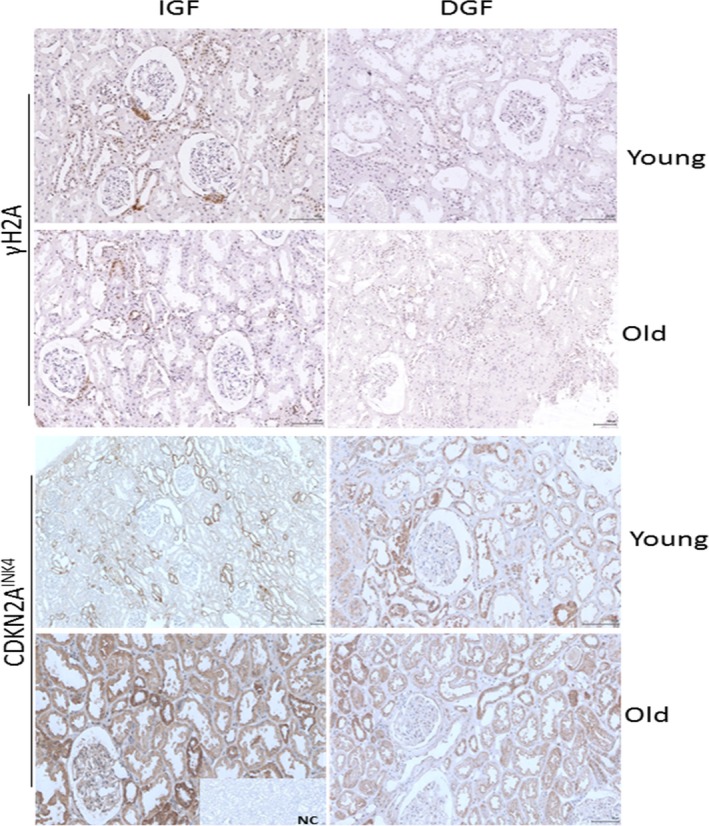
Immunohistochemical staining for γH2A (top panel) and CDKN2A^INK4^ (bottom panel) on kidney biopsies form young and old patients with IGF (immediate graft function) and DGF (delayed graft function) outcomes, respectively. Sections were scanned at 20x magnification. NC: negative control

Four preperfusion biopsies from the RNAseq cohort were selected to visualize histological differences in extreme DGF and IGF outcomes between young (age 40–42) and old (age 74) donors and were stained for CDKN2a/p16^INK4^ and γH2AFX (Figure 4).

Overall CDKN2a/p16^INK4^ positivity appears to be predominately cytoplasmic, with greater positivity within distal tubular epithelium versus proximal tubular epithelium across all samples and with proximal tubular epithelium showing greater positivity in older donors compared to younger donors.

Overall, regardless of DGF/IGF status, positivity for γH2AFX appeared to be predominately nuclear in tubular epithelial cells, at least mild and more often in the distal tubules than the proximal tubules. Interestingly, the subcellular localization of the γH2AFX signal within arterial myocytes appeared to associate with donor age more so than with immediate function status after transplantation, in that biopsies from kidneys from younger donors showed more cytoplasmic than nuclear γH2AFX, with older kidneys showing greater nuclear than cytoplasmic positivity in arterial myocytes.

## 3 DISCUSSION

To our knowledge, this work is the first study in human subjects that demonstrates a unique molecular signature for impaired physiological resilience (DGF), encompassing epigenetic and transcriptomic data sets. Critically, at a translational level, it also provides a platform for the development of a universal IRI signature and the ability to relate it to post‐transplant outcomes. This is also the first study linking DNA methylation status to reperfusion injury and DGF outcome, in the context of immune system status, overall dysregulation of cellular homeostasis and its consequences for allograft performance. These data, together with the validation of DGF‐associated gene products at the protein level, provide a unique and synchronous genome, transcriptome and proteome snapshot.

Our study provides strong evidence that biological age in combination with physiological stress, resulting from immune system activation and generation of inflammatory responses, plays a major role in DGF occurrence and the physiological manifestations of IRI. From a clinical perspective, this also suggests that these effects are driven by donor characteristics, which may therefore be even more discriminating than reperfusion injury itself. Correspondingly, BioAge in the preperfusion biopsy analyses appeared to be a significant determinant of post‐transplant allograft function, in keeping with previous observations centring on the CDKN2 and CDKN1 loci (Gingell‐Littlejohn et al., 2013; McGuinness et al., 2016). Furthermore, it suggests strongly that increased allograft biological age is contributory to less successful outcomes in renal transplantation and poorer post‐transplant performance.

Activation of the immune system may be a prerequisite driver for DGF occurrence, with the predicted top ranked upstream regulators being associated with innate immune system activity. Upregulation of transcription for UBD, NLRC4, IFNγ and IFNγ‐inducible targets OAS2 and CXCL10 is congruent with a model of inflammasome activation leading to the recruitment of corresponding IFNγ effector cells, represented by elevated transcriptional levels observed for CD52, CD45, FCGR1C and KLRB1. These data suggest that therapeutic intervention to modulate the innate immune pathway activation and related events would be expected to mitigate the effect of IRI and reduce acute kidney injury during the peritransplantation period. Furthermore, these observations are consistent with the thesis of inflammaging, whereby increased chronic inflammation may hyperinflate biological ageing processes, thus causing more rapid deterioration of organ function.

Three of the top five ranked DGF‐specific transcripts locate to the major histocompatibility class 1 (HLA) locus. GABBR1 (6p22.1) and ubiquitin D (UBD; 6p21.3) are located very close to the region coding for HLA‐F (Fan et al., 1996), suggesting that this locus may be of importance for the development of DGF and impairment of physiological resilience. The third transcript, BTN3A2, originates from the gene located in the juxta‐telomeric region of the HLA locus and is involved in the adaptive immune response and inhibition of INFγ release. GABBR1 has been associated with proteasome activation and cytoskeleton remodelling, with differential responses to IRI, dependent upon severity of insult (Caldeira, Salazar, Curcio, Canzoniero, & Duarte, 2014). UBD has also previously been implicated in renal pathology, via involvement in inflammatory‐mediated signalling and immune system modulation. UBD directs its substrates for 26S proteasomal degradation (irreversible proteolysis) and accelerates autophagy in nutrient‐deprived conditions (Gong et al., 2010; Schmidtke, Aichem, & Groettrup, 2014). This can be supported by significant transcriptome changes associated with mitochondria and mitochondrial energy metabolism (PMAIP1, SLC25A25, BBC3, SH3GLB1,NEFL, SLC25A2, HSP90AA1, CPT1B, GADD45B, DNAJB1, LRP5L, ARRDC3, HSPA1B, HSPA1A, EDN1) and autophagy (MAP1LC3B, CXCR4, DAPK1, EIF2AK3, INFG, IGF1, PTEN, TNFα, RB1, DRAM2, HSP90AA1).

**Figure 5:**
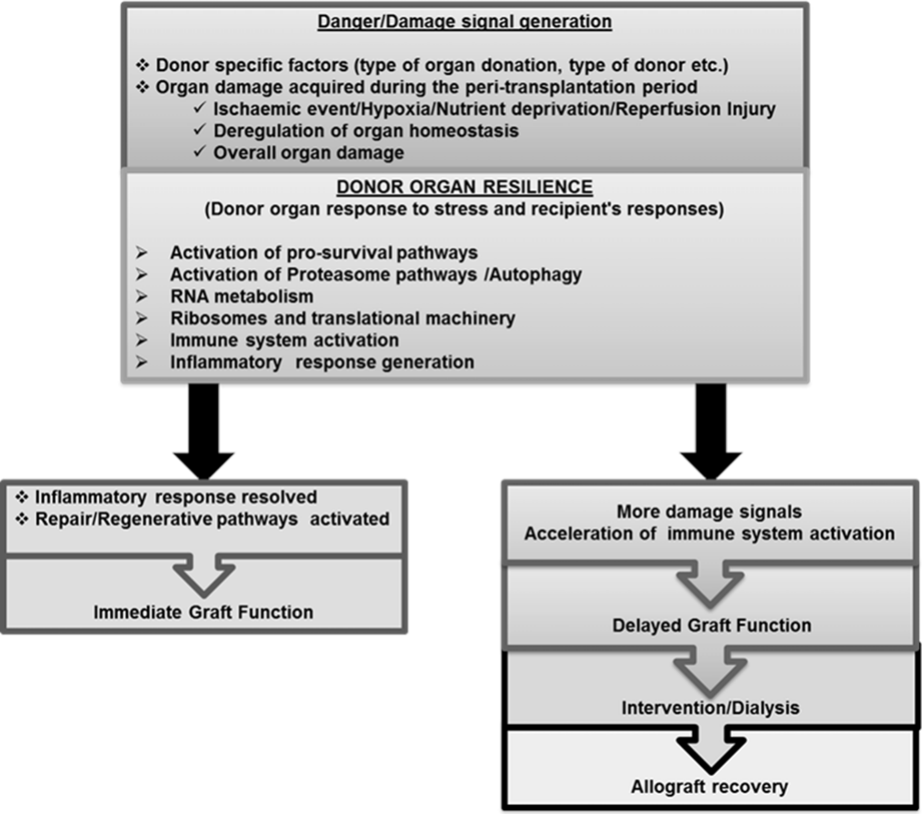
Proposed model for DGF

The activation of the recipient immune system and downstream signalling pathways may be superseded by the subsequent impact of repair/regeneration pathways. It is possible that this inability to sufficiently resolve inflammatory responses manifests as DGF and could be attributed to a differential response to reperfusion injury by biologically older allografts and decreased resilience to stress (Gingell‐Littlejohn et al., 2013; McGuinness et al., 2016; O'Neill et al., 2015; Salvadori, Rosso, & Bertoni, 2015). Such a scenario is supported by the reperfusion signature associated with cellular events (metabolic shift, autophagy, RNA metabolism, ribosomal biogenesis, protein synthesis) activated in response to environmental stress (ischaemic damage, nutrient deprivation and hypoxia), which facilitates cellular repair after insult, or induces apoptosis if the damage is too severe. Recent evidence has indicated that these prosurvival and repair pathways associated with ageing are conserved across taxa, and include the mTOR, AKT and p38 pathways, suggesting that insufficient resolution of the response to peritransplant stresses is associated with dysregulation of cellular homeostasis (Gingell‐Littlejohn et al., 2013); Kennedy & Lamming, 2016; Figure 5). These pathways are critical regulators of cellular metabolism allowing cells to sense and adapt to environmental factors, with some being involved in the regulation of lifespan in model organisms, including mTOR signalling. Notably, sirolimus, a mTOR inhibitor, is already used as an immunosuppressant following renal transplantation. While use of sirolimus is associated with reduced risk of malignancy in transplant recipients, it also correlates with an increased risk of death when the allograft has originated from a cadaveric donor. No such correlation, however, has been observed from living donor allografts, supporting the central role of mTOR signalling and associated pathways for the regulation of cellular metabolism and health span (Knoll et al., 2014).

Our results are in keeping with recent studies linking organ biological age, as opposed to chronological age, to allograft performance post‐transplant and overall kidney function (Gingell‐Littlejohn et al., 2013; McGuinness et al., 2016). Increased biological age of renal allografts can be associated with reduced functional/repair capacity as a result of a greater degree of allostatic load/overload. This is thought to contribute to the DGF phenotype, as a result of an inability to restore physiological homeostasis in the face of peritransplant stress and subsequent recipient immune challenge (Kooman et al., 2014). Our data are in keeping with such a scenario, with organs displaying DGF also exhibiting a greater change in transcriptional amplitude in DGF signature transcripts following transplantation and requiring a longer period to restore physiological homeostasis, which may be related to deficient proteostasis. These data indicate that allografts exhibiting DGF may therefore be displaying features of allostatic overload at a transcriptional level whose effects are extrapolated across the organ as a whole, resulting in functional impairment (Kooman et al., 2014).

Analysis of transcript expression observed solely in DGF, but not IGF, in relation to perfusion status, indicated a transcript biotype shift (Supporting Information Table S1 in Data S8), including an increase in antisense, pseudogenes, noncoding and coding RNAs, for example immunoglobulin gene (IgV and IgV pseudogenes) and T‐cell receptor (TR J) transcripts. These changes in the transcriptome biotypes further support the hypothesis that the response to IRI, both in magnitude and context, are dependent upon donor characteristics and organ response/resilience to stress and may also reflect deregulation of alternative splicing networks and epigenome status overall. The biotype changes observed may reflect a burst of “transcriptional noise” in DGF allografts in response to IRI, as a direct result of changes in the methylation status of promoters and intragenic regions (Huh, Zeng, Park, & Yi, 2013). These observations are also consistent with the derepression of LINE elements in ageing cells (De Cecco et al., 2013).

Immunological response to “danger signals” may lead to excessive activation of proinflammatory cytokines and chemokines, consequently leading to organ damage over time, as observed in the case of autoimmune diseases, where there is a loss in ability to downregulate/attenuate proinflammatory signalling (de Jesus, Canna, Liu, & Goldbach‐Mansky, 2015). This suggests that donor characteristics are important for DGF occurrence and may be linked to organismal/organ stress levels in relation to the type of organ donation (Bon et al., 2012; Morrissey & Monaco, 2014). Ultimately, allograft quality will be related to organ resilience to stress, and this by itself provides an opportunity for the development of new therapeutic interventions aimed at exploiting this phenomenon. Recent studies focusing on the progression of chronic kidney disease (CKD) have exemplified the importance of epigenetic changes and loss of kidney function (Smyth, McKay, Maxwell, & McKnight, 2014; Wing et al., 2014). Our data have indicated rapid changes in the epigenome during perfusion, suggesting that the effect of IRI on long‐term allograft function may be more pronounced than originally anticipated.

The lack of available models for DGF has meant that direct validation of targets and any related mechanism has not been possible. To mitigate the impact of this shortfall, we have therefore used our DGF transcriptomic signature to identify any commonality with other renal/urological transcriptomic expression profiles derived from publicly available data sets. These included renal interstitial fibrosis, kidney transplant failure and rejection, kidney disease, nephrotic syndrome, cystic disease of kidney and renal tubular disorder (Supporting Information Data S3). Notably, the overlap identified encompassed transcripts involved in immune system activation, both supporting the importance of interaction between lymphoid and nonlymphoid cells in the context of renal function and highlighting the biological plausibility of the DGF‐related findings.

Overall, our data suggest that allografts exhibiting DGF present with an impaired ability to restore physiological homeostasis in response to stress, consistent with their biological age and associated allostatic load. This is reflected in changes in epigenome, transcriptome and dysregulation of RNA metabolism. The magnitude of change in transcriptional amplitude in response to physiological stress, along with elevated expression of noncoding RNAs and pseudogenes, raises the possibility that reduction in available cellular resources for activation of damage repair mechanisms slows down physiological and cellular repair processes, resulting in long‐term damage to the allograft (Figure 5).

## 4 EXPERIMENTAL PROCEDURES

### 4.1 Clinical cohort characteristics

Fifty‐five paired preperfusion and postperfusion renal biopsies collected from deceased donors were included in this study, and all kidneys were subsequently transplanted with no occurrence of primary nonfunction. Detailed patient characteristics and follow‐up markers are described in Table 1. This study and consent procedure was approved by the Regional Ethics Committee of North Glasgow NHS Trust (GN10SU334, 10/S0704/42, NHS GG&C Biorepository −276 and 348). Donors from the national pool donated their organs for transplantation. The recipient of the organ provided preoperative written informed consent and records are stored at Queen Elizabeth University Hospital (QEUH). From this cohort, 24 pairs of samples defined as the extreme DGF phenotype or immediate graft function were selected for further analysis (Figure 1a).

### 4.2 Biopsy processing

Total RNA, genomic DNA and protein were sequentially isolated from the same tissue biopsies using TRI^®^Reagent according to the manufacturer's instructions (Invitrogen, UK).

#### 4.2.1 RNA isolation

Total RNA from kidney biopsies was extracted using TRI^®^Reagent according to the manufacturer's instructions, DNase treated (RNA Clean & Concentration, #R1015, Zymo Research, USA) and stored at −80°C for further analysis. RNA underwent spectral analysis (A_260/280 nm_) and determination of RIN number (RNA Nano kit and 2100 BioAnalyzer (#5067–1511, Agilent Technologies, Inc. USA). For further analysis, samples with RIN>6.0 were used.

#### 4.2.2 RNAseq and data analysis

Libraries, from 400 ng total RNA, were created using ribosomal depletion (*n* = 48, 24 paired biopsies; TruSeq Stranded Total RNA Ribo‐Zero H/M/R Gold, Illumina). Prepared libraries were assessed by Qubit® (Life Technologies, Inc. USA) and Bioanalyser (High Sensitivity DNA kit [#5067–4626, Agilent Technologies, Inc., USA]). Libraries were sequenced on a NextSeq500 (Illumina) using a paired‐end 75 × 75 bp run. The raw sequence reads in FASTQ format were further analysed using the following pipeline: Initial QC for RNAseq output was analysed using FastQC v.0.11.2 (https://www.bioinformatics.bbsrc.ac.uk/projects/fastqc), adapters were removed using trim_galore v.0.3.7 (https://www.bioinformatics.babraham.ac.uk/projects/trim_galore/). Three bases from the 3' end of all paired‐reads were trimmed to avoid any biases related to adapter sequence or basecall quality. Poor quality bases (phred score <20) were removed. Two samples 39B1 and 106B were excluded from further analyses as they failed QA and QC checks. The trimmed reads were mapped against the reference human genome (GRCh38 from ensemble) using Tophat v.2.0.13 (Kim et al., 2013). The annotation file (GRCh38 release 78 from Ensemble) was used for mapping of transcript annotations. Mapped fragment counts were summarized using featureCounts with ensemble gene‐ids using subread v1.4.6 (Liao, Smyth, & Shi, 2014). Differential gene expression (DGE) analysis was performed using DESeq2 v1.6.3 (Love, Huber, & Anders, 2014). Raw count data were transformed to log2 scale to normalize expression counts. Multiple testing correction was performed using the Benjamin–Hochberg approach to control false discovery rate (FDR) at 10% (FDR ≤ 0.1 was considered significant). Differentially expressed gene targets were analysed using Ingenuity®Pathway Analysis (IPA®, QIAGEN's, USA) and NextBio Research (Illumina, USA).

#### 4.2.3 Alternative splicing analysis

Alternative splicing events were investigated in the RNAseq cohort (top 100 differentially expressed transcripts, pre vs. post) and DGF‐specific transcripts identified by RNAseq using DEXSeqv1.16.10 (Anders, Reyes, & Huber, 2012). Differential exon usage (DEU), followed by the application of generalized linear modelling, was used to identify alternative splicing events (FDR < 0.1 was considered significant).

#### 4.2.4 Whole genome bisulphite sequencing and analysis

Genomic DNA was isolated from the same biopsy, using TRI®Reagent, after separation of RNA into aqueous fraction and further purified (Genomic DNA Clean & Concentrator, Zymo Research, USA). 100 ng of genomic DNA was bisulphite converted using EZ DNA Methylation‐Gold^TM ^Kit (Zymo Research). Bisulphite‐converted genomic DNA was used to generate indexed libraries using EpiGnome^TM^Methyl‐Seq kit (Epicentre®Illumina) according to the manufacturer's instruction. Library quality and quantity were assessed using DNA High Sensitivity kit (Agilent Technologies, Inc. USA). Sample selection for WGBS included 20 samples (10 matched pairs with five being DGF and five IGF). The resulting 20 libraries were sequenced on a NextSeq500 (Illumina) with 30× coverage, paired‐end run (2 × 150 bp). The raw sequence reads in FASTQ format underwent QC as previously described above. Sample 180b1 was excluded from further analysis as it failed QA and QC.

The human reference genome (GRCh38) was in silico bisulphite converted before aligning trimmed reads with Bismark v0.10.1 and bowtie2 (v2.1.0) as previously described(Krueger & Andrews, 2011; Langmead & Salzberg, 2012). PCR bias was removed by a deduplication step. The methylation content was measured on CpG context sites of DGF‐specific targets. The promoter and intragenic regions were extracted from biomart API (BiomaRt; Durinck, Spellman, Birney, & Huber, 2009), and differences within the methylated CpG sites were further analysed using Kruskal–Wallis test. FDR correction for multiple comparison was applied for all analyses. Adjusted *p*‐value below 0.05 was considered as statistically significant.

#### 4.2.5 QPCR and data validation

For each individual RT reaction, 150 ng of total RNA from each sample was used and reverse transcription was performed using SuperScript^®^II Reverse Transcriptase (# Life Technologies Inc., UK) and then qPCR was performed. Gene expression was analysed using TaqMan®gene expression assays, or custom design assays using Roche UPL (Supporting Information Data S9), which were normalized against HPRT1 and 18S rRNA control primer sets. Taqman® assays, including standards, were performed using the manufacturers recommended qPCR protocols and TaqMan®Master Mix (#4370074, Life Technologies, UK). For UPL probes, primers were used at final concentration 360 nM while probes were used at final concentration of 100 nM. The comparative threshold cycle method (ΔΔ*C*T) was used to quantify relative gene expression, and the obtained quantification was transformed to exponential value 2^−ΔΔ^ *^C^* ^T^. Commercially available RNA was used as a calibrator (#AM7976, Life Technologies, Inc.). Further testing involved Spearman correlations and Kruskal–Wallis test. FDR correction for multiple comparison was applied for all analyses. Adjusted *p*‐value below 0.05 was considered as statistically significant.

#### 4.2.6 RNAscope

In situ hybridization detection for CD45 (601998), REG1B (312058), INGγ (310508), SEMA3A (416568), ACKR3 (441458) and PPIB (313908) mRNA was performed using RNAscope 2.5 LS (Brown) detection kit (Advanced Cell Diagnostics, Hayward, CA) on a Leica Bond Rx autostainer strictly according to the manufacturer's instructions. The analysis was performed using an established method at the Histology Core in the Beatson Institute for Cancer Research, Glasgow, UK.

#### 4.2.7 Western blot

Protein fractions were isolated from the phenol–ethanol fraction after removal of genomic DNA (TRI^®^Reagent, Invitrogen, UK). Protein concentration was estimated using DC^TM^Protein assay (BioRad, UK), and 7.5 µg of total protein was loaded per well. Samples were resolved in 4%–12% or 12% SDS‐PAGE in MOPS buffer using NuPAGE®System (Life Technologies Inc., UK) and transferred onto the polyvinylidene fluoride (PVDF) membrane. After immunoblotting, membranes were washed in Tris‐buffered saline (TBS), blocked in 5% nonfat dry milk in TBS‐0.1% Tween 20 (TBST) for 1 hr at room temperature, followed by the incubation with primary antibodies in blocking solution overnight at 4°C. After incubation, membranes were washed in TBST and incubated with the goat antirabbit IgG (1:10,000; Cell Signalling, #7074S) or goat antimouse IgG (1:5,000, Cell Signalling, #707) horseradish peroxidase (HRP)‐conjugated for 1 hr in room temperature. The reaction was developed using Enhanced Chemiluminescence (ECL) System (Life Technologies Inc.). The following primary antibodies from Abcam (UK) were used: CHGB (1:4,000; ab151568), REG1B (1:1,000; ab87205), Corin (1:500; ab56158), UBD (1:500, ab134077), KLRB1 (1:500, ab197979), SEM3A (1:1,000, ab23393), ACKR3 (1:2,000), ZNF676 (1:500, ab179754), TAGAP (1:1,000, ab187664), HPRT1 (1:10,000, ab109021), FCGR1C (ab119843), FCGR2C (ab125013), CD52 (ab194860) and PTPRC (ab40763) and β‐tubulin/HRP conjugated (1:2,000, ab20058, 2 hr at RT).

#### 4.2.8 Immunohistochemistry

The analysis was performed using an established automated method at the Histology Core in the Beatson Institute for Cancer Research, Glasgow, UK. Heat‐induced antigen retrieval was performed using sodium citrate retrieval buffer (pH = 6, Thermo, UK) at 98°C for 25 min followed by peroxidase block (Dako, UK) for 5 min. The previously optimized primary antibodies from Abcam: ACKR3 (ab72100, 1:500), INFγ (ab9657, 1:500), SEMA3A (ab23393, 1:500) and CD45 (Dako, M0701, 1:1,000), REG1B (MyBiosource, MBS2025956, 1:500) and an appropriate secondary antibody (EnVision, Dako, UK) were used. Staining and counterstaining were performed using 3,3‐diaminobenzidine (DAB) and haematoxylin, respectively. Previously validated CDKN2A/p16 (*M*‐156‐sc759; 1:250; Santa Cruz Biotechnology Inc, USA) and γH2AFX (ab81299, 1:1,000; Abcam, UK) antibodies were stained manually.

### 4.3 Data and materials availability

RNAseq and WGBS data have been deposited at GEO repository with reference no: GSE90865 for publication, and this includes subseries GSE90861 and GSE90863. All relevant data are available upon request. Data from this study are available from the senior author (PGS).

## CONFLICT OF INTEREST

No conflict of interest.

## AUTHORS’ CONTRIBUTION

PGS, RBK, LD, KS and DBK designed and coordinated study. KS, DBK and OS collected clinical samples and compiled clinical data. DMcG, LM and OS performed laboratory work. SM and PAW generated computational data sets. PGS, DMcG, SM, PAW, KS, LD SC and DBK compiled and analysed data. PGS, DMcG and KS drafted manuscript. All authors read and commented on manuscript drafts.
