# Supplementary material for: A molecular signature for delayed graft function
Source: Aging Cell. 2018 Aug 9;17(5):e12825. doi: 10.1111/acel.12825 (PMC6156499; doi:10.1111/acel.12825)
Supplement: Supplementary file 1 [file ACEL-17-e12825-s001.pdf]

SD (Supplementary Data)

The RNAseq cohort characteristics

A molecular signature for Delayed Graft Function

Dagmara McGuinness[1], Suhaib Mohammed[1], Laura Monaghan[1], Paul A. Wilson[2], David B. Kingsmore[3], Oliver Shapter[1,3], Karen S. Stevenson[3], Shana M. Coley[4], Luke Devey[5], Robert B. Kirkpatrick[6] and Paul G. Shiels[1]*

[1]University of Glasgow, College of Medical, Veterinary & Life Sciences, Wolfson Wohl Translational Research Centre, Institute of Cancer Sciences, Garscube Estate, Switchback Road, Glasgow, G61 1QH, Scotland

[2]Computational Biology, GlaxoSmithKline Medicines Research Centre, Gunnels Wood Road, Stevenage, Hertfordshire, SG1 2NY, UK

[3]NHS Greater Glasgow and Clyde, Renal Transplant Unit, Ward 4c, South Glasgow University Hospital, Glasgow, G51 4TF, Scotland

[4]University of Glasgow, College of Medical, Veterinary & Life Sciences, Research Institute of Infection Immunity and Inflammation, 120 University Place, Glasgow, G12 8TA, Scotland

[5]Metabolic Pathways Cardio Therapy Area Unit, GlaxoSmithKline, 709 Swedeland Road, King of Prussia, PA, USA

[6]The Pipeline Futures Group, GlaxoSmithKline, 1250 South Collegeville Road, Collegeville, PA, USA

Corresponding author: Prof Paul G Shiels

University of Glasgow, Wolfson Wohl Translational Research Centre, Institute of Cancer Sciences, Garscube Estate, Switchback Road, Glasgow, G61 1QH

E-mail: paul.shiels@glasgow.ac.uk

SFigure1. Principal Component analysis (PCA) of RNA-seq cohort in relation to perfusion status and DGF and DGF alone (top panel), donor gender alone or in combination with perfusion or DGF status (middle panel) and in relation to the recipent gender in the same conditions (bottom panel)

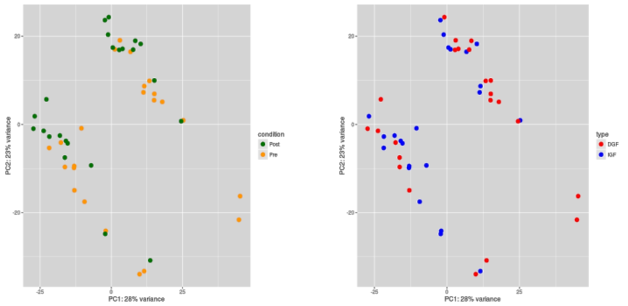

Donor

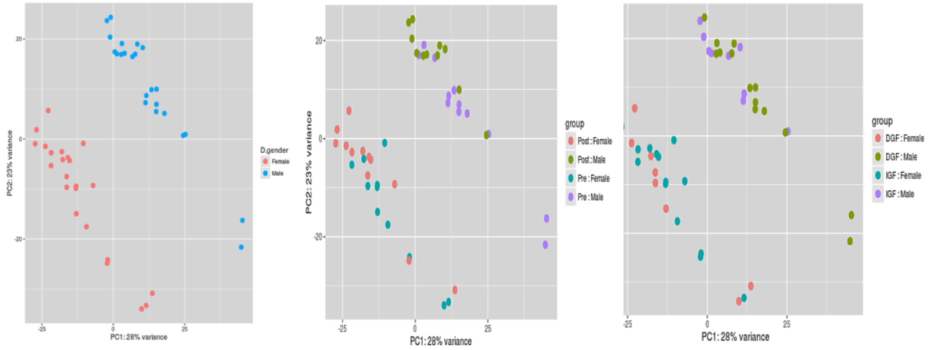

Recipient

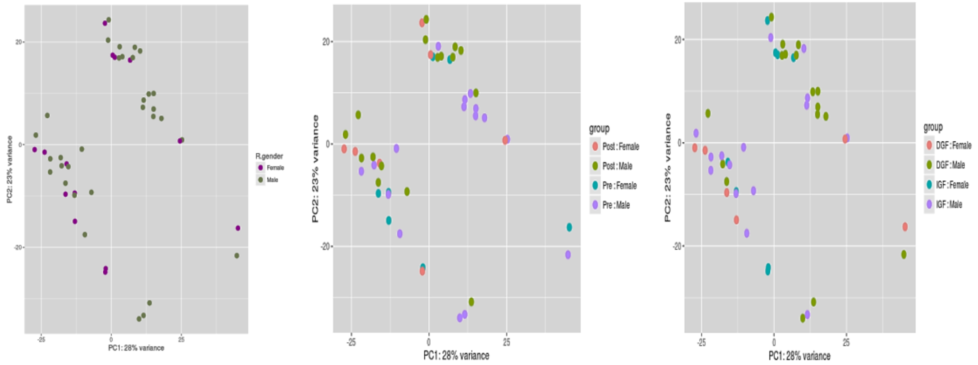

SFigure 2. Differential number of reads for the paired samples in different perfusion status (pre - vs. post-perfusion) in the relation to DGF or IGF outcome. Colour legend reflects matched reads count and samples IDs in the RNA-seq cohort.

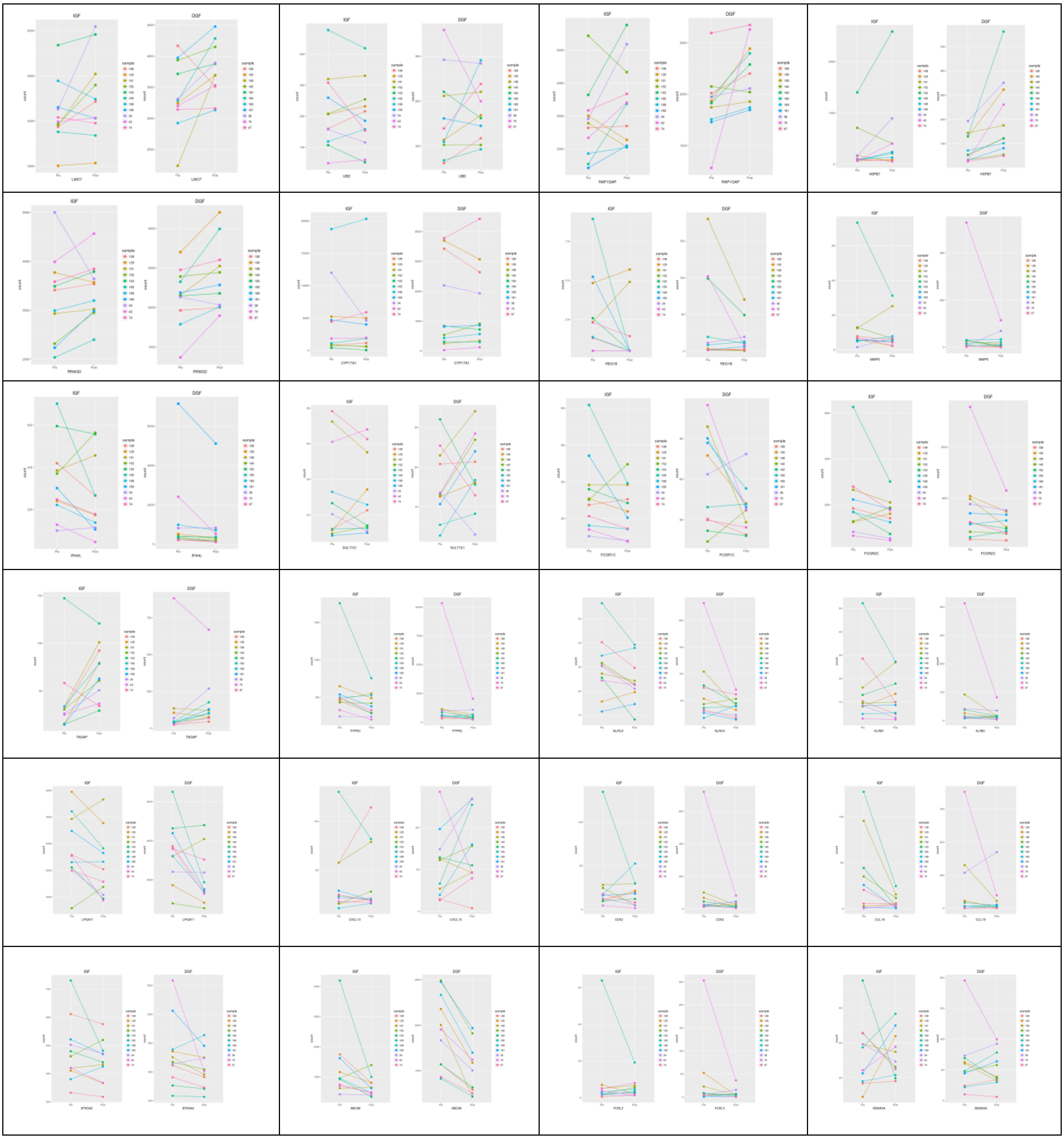

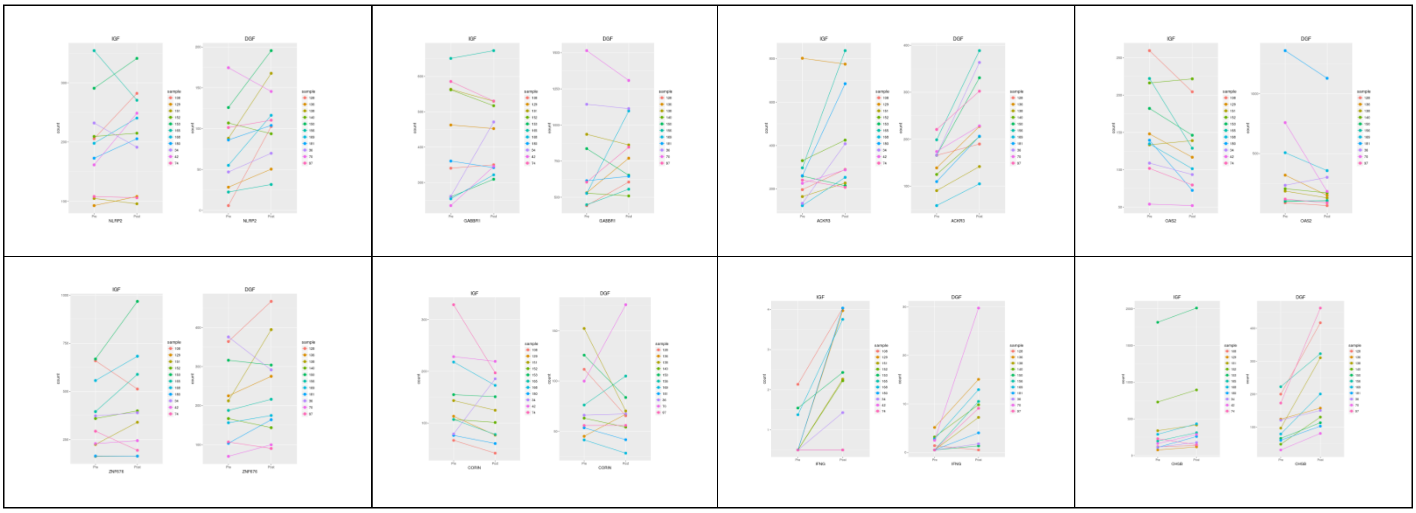
IGF
DGF
NLRP2
GABBR1
ACKR3
OAS2
ZNF676
CORIN
IFNG
CHGB
count
sample
Pre
Post
