# Supplementary material for: A molecular signature for delayed graft function
Source: Aging Cell. 2018 Aug 9;17(5):e12825. doi: 10.1111/acel.12825 (PMC6156499; doi:10.1111/acel.12825)
Supplement: Supplementary file 2 [file ACEL-17-e12825-s002.pdf]

SD1 (Supplementary Data 1): The complete list of DGF targets and results of pathway analyses (S1Table 1-S1Table 5).

S1 Table 1. The complete list of differentially expressed genes based on RNAseq analysis (median count ≥ 10, false discovery rate [p] < 0.1) in human samples with diagnosed Delayed Graft Function in comparison to these with IGF.

S1 Table 2. The list of the canonical pathways associated with Delayed Graft Function (p<0.05).

S1 Table 3. The complete list of upstream regulators involved in Delayed Graft Function.

S1 Table 4. Networks and associated network functions in Delayed Graft Function.

S1 Table 5. Top analysis ready molecules

S1Table 1. The complete list of differentially expressed genes based on RNAseq analysis (median count ≥ 10, false discovery rate [p] < 0.05) in human samples with diagnosed Delayed Graft Function in comparison to these with Immediate function .

Gene Symbol	Base Mean	log2 FC (FoldChange)	log2FCSE	Stats	p value	p adjusted
**GABBR1**	608.5974186	0.71220878	0.134258221	5.304768505	1.13E-07	0.003347027
**REG1B**	10.22722944	1.058973655	0.205748222	5.146939516	2.65E-07	0.003927608
**UBD**	367.7777067	0.91468726	0.18089202	5.05653737	4.27E-07	0.004222125
**RNA5S9**	224.0124977	-1.052146577	0.213767289	-4.921925057	0.000000857	0.006356149
**REG1A**	3235.70114	0.96643192	0.211233814	4.575176207	4.76E-06	0.020758198
**BTN3A2**	592.8306402	0.548529493	0.120051473	4.569119208	4.90E-06	0.020758198
**NLRP2**	143.0112313	-0.836145338	0.180839466	-4.623688385	3.77E-06	0.020758198
**DISP2**	30.13197022	0.842620468	0.189541629	4.445569414	8.77E-06	0.032508477
**TAGAP**	104.1867837	0.920478356	0.21054008	4.371986352	1.23E-05	0.036527605
**CPXM1**	70.74694718	0.859070836	0.196113628	4.380474946	1.18E-05	0.036527605

**FLG**	21.8655036	-0.937897491	0.215942102	-4.34328222	0.000014037	0.037858963
**GRIN3B**	17.86831184	0.889782606	0.208671769	4.264029641	2.01E-05	0.049637727
**NLRC4**	40.6080323	0.760054551	0.181441463	4.188979397	2.80E-05	0.063754921
**ABHD11-AS1**	22.3093198	0.783682804	0.187806513	4.172820169	3.01E-05	0.063754921
**CD74**	12915.20058	0.476845122	0.117112294	4.071691414	0.000046673	0.065937816
**LINC00243**	6.486381783	0.843599608	0.207136357	4.072677626	4.65E-05	0.065937816
**CXorf21**	33.73880347	0.694417794	0.169085725	4.10689781	4.01E-05	0.065937816
**MYRFL**	177.3234652	0.69908133	0.170733407	4.094578452	4.23E-05	0.065937816
**MMP25-AS1**	93.15475951	0.502005417	0.123264618	4.072583226	4.65E-05	0.065937816
**CHGB**	288.1278565	-0.854107759	0.208317774	-4.100023454	4.13E-05	0.065937816
**ABCA7**	304.6919106	0.499461526	0.120613838	4.140996891	0.00003458	0.065937816
**TXLNB**	8.671936337	0.814578898	0.202703682	4.018569807	5.86E-05	0.075527586
**TYMP**	686.3844974	0.652113866	0.162102785	4.022841843	5.75E-05	0.075527586
**CD69**	201.5739178	0.864388493	0.2159966	4.001861562	6.28E-05	0.077635048
**AC003104.1**	82.52242109	0.451818367	0.11317084	3.992356752	6.54E-05	0.077635048
**SIDT1**	100.2065047	0.632764075	0.161777391	3.911325753	9.18E-05	0.083603919
**GPR125**	1364.921372	-0.215078815	0.055161517	-3.899073631	9.66E-05	0.083603919
**CORIN**	106.9488254	-0.641393009	0.164689425	-3.89456098	9.84E-05	0.083603919
**RP11-815N9.2**	5.080788469	0.852302023	0.215269713	3.959228689	7.52E-05	0.083603919
**RN7SL253P**	7.940430519	0.697460265	0.177086437	3.938530109	8.20E-05	0.083603919
**RP11-343L5.2**	34.60390137	-0.589339835	0.150755808	-3.9092347	0.000092589	0.083603919
**BMP6**	168.2267289	-0.798122992	0.204163306	-3.909238189	9.26E-05	0.083603919
**OAS2**	224.1523841	0.778437207	0.197752772	3.936416156	8.27E-05	0.083603919
**VSTM2L**	30.64290351	0.660761737	0.169689796	3.893939142	9.86E-05	0.083603919
**SIGLEC10**	90.09530896	0.826820522	0.209701279	3.942849204	8.05E-05	0.083603919
**CXCR4**	383.8573085	0.78833667	0.203024609	3.882961151	0.000103192	0.084534236
**DUSP18**	159.9950827	0.281641415	0.072630053	3.877753159	0.000105426	0.084534236
**TCN1**	6.072291166	0.760259096	0.197133439	3.856570964	0.000114989	0.087473988
**CPNE7**	45.69483686	0.774930839	0.200664921	3.861815189	0.000112548	0.087473988
**TMEM200B**	255.9930912	0.468333532	0.121764653	3.846219069	0.000119955	0.088970269

**ODF3B**	204.9661365	0.640648047	0.167276375	3.829877623	0.000128207	0.092771837
**ISG20**	105.9181732	0.707504783	0.185324979	3.817643947	0.000134732	0.095172245
**WDR54**	166.0235979	0.442264422	0.117094292	3.776993859	0.000158733	0.098986529
**ACKR3**	265.6798631	-0.647407849	0.171416803	-3.776805066	0.000158853	0.098986529
**IL7R**	276.7285636	0.810171163	0.213915596	3.787340325	0.000152269	0.098986529
**C2**	1367.355259	0.676600366	0.17823143	3.796189967	0.000146937	0.098986529
**RNASE6**	97.93308969	0.775673263	0.205488493	3.774777127	0.000160151	0.098986529
**XAF1**	413.7683494	0.703897674	0.186094483	3.782474695	0.000155277	0.098986529
**CAB39L**	530.7636522	-0.341103108	0.090547634	-3.767112348	0.000165147	0.099991268

S1Table 2. The list of the canonical pathways associated with Delayed Graft Function ($p<0.05$).

**Canonical Pathways**	**-log(p-value)**	**Ratio**	**z-score**	**Molecules**
GABA Receptor Signaling	2.13E+00	2.99E-02	NaN	GABBR1,UBD
TREM1 Signaling	2.04E+00	2.67E-02	NaN	NLRP2,NLRC4
Salvage Pathways of Pyrimidine Deoxyribonucleotides	1.82E+00	1.25E-01	NaN	TYMP
Glycogen Degradation II	1.68E+00	9.09E-02	NaN	TYMP
Role of Pattern Recognition Receptors in Recognition of Bacteria and Viruses	1.62E+00	1.60E-02	NaN	OAS2,NLRC4
Glycogen Degradation III	1.61E+00	7.69E-02	NaN	TYMP
Ephrin Receptor Signaling	1.36E+00	1.15E-02	NaN	GRIN3B,CXCR4

S1Table3. The complete list of activated and inhibited upstream regulators involved in Delayed Graft Function.

Upstream Regulator	Molecule Type	Activation z-score	p-value of overlap	Target molecules in dataset
**Activated**				
IL1B	cytokine	2.412	5.62E-04	CD69,CD74,CXCR4,ISG20,OAS2,TYMP,UBD
poly rI:rC-RNA	biologic drug	2.359	8.07E-05	C2,CD69,CD74,CXCR4,ISG20,OAS2
lipopolysaccharide	chemical drug	2.268	3.79E-04	ACKR3,CD69,CD74,CXCR4,IL7R,ISG20,NLRP2,OAS2,TYMP,XAF1
TNF	cytokine	2.005	2.10E-02	ACKR3,CD69,CXCR4,IL7R,OAS2,TYMP,UBD
TGM2	enzyme	2	6.15E-04	CD74,NLRC4,OAS2,XAF1
Ifnar	group	2	1.47E-05	CD74,ISG20,OAS2,XAF1

S1Table4. Networks and associated network functions in Delayed Graft Function.

ID	Score	Focus Molecules	Top Diseases and Functions	Molecules in Network
1	37	16	Cell Signaling, Nucleic Acid Metabolism, Small Molecule Biochemistry	ABCA7,ACKR1,ACKR3,ADGRA3,Akt,APOA1,ARR3,CASR,CHGB,CORIN,CPNE7,CPXM1,CXorf21,CYP26B1,DISP2,ESR1,FLG, GABBR1, GABBR2,Gpcr,GPR18,GRIN3B,MAPK3,MEMO1,NMDAReceptor,ODF3B,P2RY14,PDGFD,PTGER4,RNASE6,SPP1, TAGAP,TGFB1, TP53,WDR54

2	31	14	Lymphoid Tissue Structure and Development, Hematological System Development and Function, Humoral Immune Response	ACKR3,Alp,BMP6,C2,caspase,CD3,CD69,CD74,CXCR4,ERK1/2,Hsp90,Ifn,IFN Beta,Ifnar,IgG2a,Igm,IL1,IL12 (complex),IL7R, Immunoglobulin, Interferon alpha,ISG20,Jnk,NFkB (complex),NLRC4,NLRP2,OAS2,P38 MAPK,PI3K (complex),SIGLEC10, STAT5a/b,TCR,TYMP,Vegf,XAF1
3	18	9	Cellular Development, Cellular Growth and Proliferation, Hematological System Development and Function	Acot1,ANKS1B,AZGP1,BTN3A1,BTN3A2,CAB39L,CBL,CD46,CD226,DUSP18,EFNA1,ERBB2,HAVCR1,HLA-DMA,IFNAR2, IFNG,IL19,IL27RA,MEFV,MTOR,NUB1,Oas,P2RY14,REG1A,REG1B,SLA2,STAP2,STAT3,TCN1,Tgtp1/Tgtp2,TXLNB,UBD, UCP2,VIM,XAF1
4	2	1	Cancer, Cardiovascular Disease, Developmental Disorder	ATXN1,CCM2,VSTM2L

S1Table5. Top analysis-ready molecules

Symbol	Exp Log Ratio	Networks	Location	Type(s)
**FLG**	-0.938	1	Cytoplasm	other
**CHGB**	-0.854	1	Extracellular Space	other
**NLRP2**	-0.836	2	Nucleus	other
**BMP6**	-0.798	2	Extracellular Space	growth factor
**ACKR3**	-0.647	1, 2	Plasma Membrane	G-protein coupled receptor
**CORIN**	-0.641	1	Plasma Membrane	peptidase
**CAB39L**	-0.341	3	Cytoplasm	kinase
**ADGRA3**	-0.215	1	Plasma Membrane	G-protein coupled receptor
**SIGLEC10**	0.827	2	Plasma Membrane	other
**DISP2**	0.843	1	Plasma Membrane	other
**CPXM1**	0.859	1	Extracellular Space	peptidase
**CD69**	0.864	2	Plasma Membrane	transmembrane receptor
**GRIN3B**	0.89	1	Plasma Membrane	ion channel
**UBD**	0.915	3	Nucleus	other
**TAGAP**	0.92	1	Cytoplasm	other
**REG1A**	0.966	3	Extracellular Space	growth factor
**REG1B**	1.059	3	Extracellular Space	other
