# Supplementary material for: A molecular signature for delayed graft function
Source: Aging Cell. 2018 Aug 9;17(5):e12825. doi: 10.1111/acel.12825 (PMC6156499; doi:10.1111/acel.12825)

**SD2 (Supplementary Data 2)**

# Comparison of the DGF-specific targets to the publicly available datasets.

## A Molecular signature for Delayed Graft Function

Dagmara McGuinness[1], Suhaib Mohammed[1], Laura Monaghan[1], Paul A. Wilson[2], David B. Kingsmore[3], Oliver Shapter[1,3], Karen S. Stevenson[3], Shana M. Coley[4], Luke Devey[5], Robert B. Kirkpatrick[6] and Paul G. Shiels[1]*

[1]University of Glasgow, College of Medical, Veterinary & Life Sciences, Wolfson Wohl Translational Research Centre, Institute of Cancer Sciences, Garscube Estate, Switchback Road, Glasgow, G61 1QH, Scotland

[2]Target Sciences Computational Biology Department, GlaxoSmithKline Medicines Research Centre, Gunnels Wood Road, Stevenage, Hertfordshire, SG1 2NY, UK

[3]NHS Greater Glasgow and Clyde, Renal Transplant Unit, Ward 4c, South Glasgow University Hospital, Glasgow, G51 4TF, Scotland

[4]University of Glasgow, College of Medical, Veterinary & Life Sciences, Research Institute of Infection Immunity and Inflammation, 120 University Place, Glasgow, G12 8TA, Scotland

[5]Metabolic Pathways Cardio Therapy Area Unit, GlaxoSmithKline, 709 Swedeland Road, King of Prussia, PA, USA

[6]The Pipeline Futures Group, GlaxoSmithKline, 1250 South Collegeville Road, Collegeville, PA, USA

**Corresponding author:** Prof Paul G Shiels

University of Glasgow, Wolfson Wohl Translational Research Centre, Institute of Cancer Sciences, Garscube Estate, Switchback Road, Glasgow, G61 1QH

E-mail: paul.shiels@glasgow.ac.uk

The DGF-specific transcript profile (see Supplementary Data1, S1Table 1) ranked by fold change and associated p-value was compared to genitourinary expression profiles derived from publicly available datasets, to determine whether the observed DGF transcriptomic profile was positively correlated with previously reported renal physiology and pathological processes. Illumina's Basespace Correlation Engine® (PMID: 20927376) was used to estimate all correlations.

**S2Figure 1**. The DGF-specific profile was found to be significantly positively correlated with a number of kidney derived expression datasets. The plot summarises high level details of the similarities between the respective expression profiles. The green bar is an Illumina derived scoring system (see Illumina protocol and publication for details of the scoring algorithm) that is used to rank the correlations. "RE" indicates that the profiles are derived from RNA expression data, while the grey number indicates the number of GEO datasets included in the reported correlation. The term to the left is an ontology term used to annotate the respective public datasets.

| Genitourinary Disorders | | | | |
|---|---|---|---|---|
| Chronic interstitial cystitis | 91 | RE | 1 | positive |
| Renal interstitial fibrosis | 90 | RE | 2 | positive |
| Kidney transplant failure and rejection | 89 | RE | 9 | positive |
| Kidney disease | 53 | RE | 5 | positive |
| Cystic disease of kidney | 52 | RE | 1 | positive |
| Nephrotic syndrome | 50 | RE | 4 | positive |
| Renal tubular disorder | 40 | RE | 1 | positive |

Further details of several of these correlations are detailed below.

**S2Figure 2.** The expression profile used for comparison to the DGF-specific profile was derived from public dataset GSE11783 (Gene expression profile of bladder tissue of patients with ulcerative interstitial cystitis). A significant positive correlation was reported with this profile. The lower leftmost section of the plot indicates that there are 23 common gene identifiers and reports the chance probability of this overlap. The rightmost plot further details attributes of the intersect.

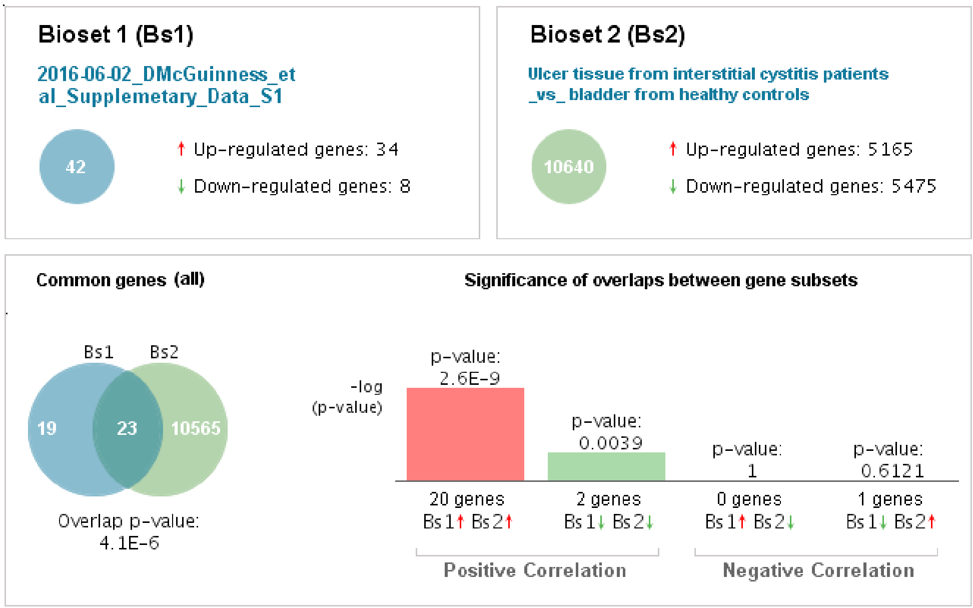


**S2Figure 3.** The expression profile used for comparison to the DGF-specific profile was derived from public dataset GSE36059 (Molecular diagnosis of T cell-mediated rejection in human kidney transplant biopsies). A significant positive correlation was reported with this profile.

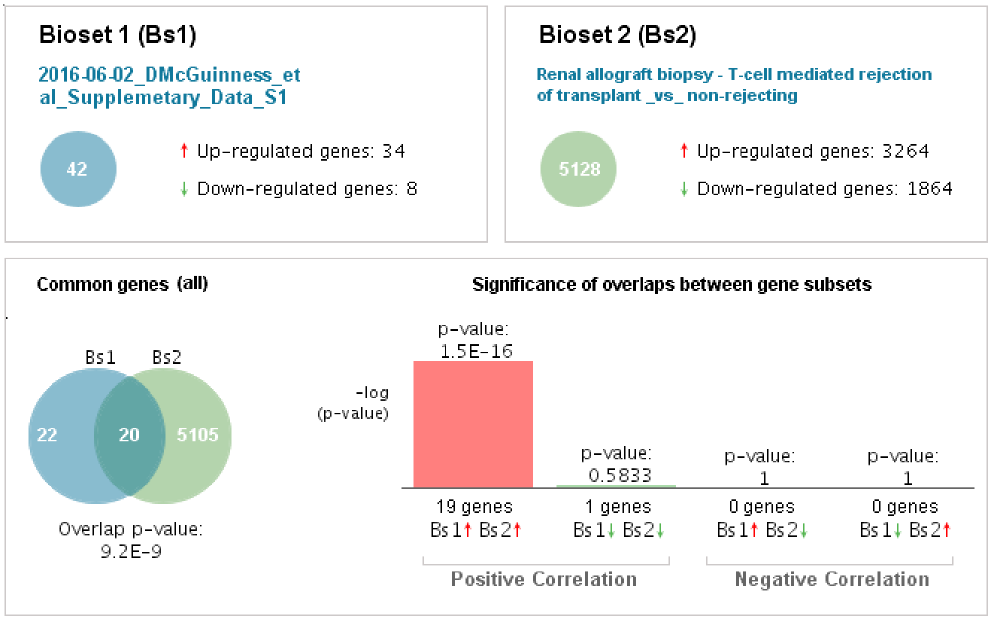

**S2Figure 4.** The expression profile used for comparison to the DGF-specific profile was derived from public dataset GSE50058 (A common rejection module for acute rejection in multiple organs identifies novel therapeutics.). A significant positive correlation was reported with this profile.

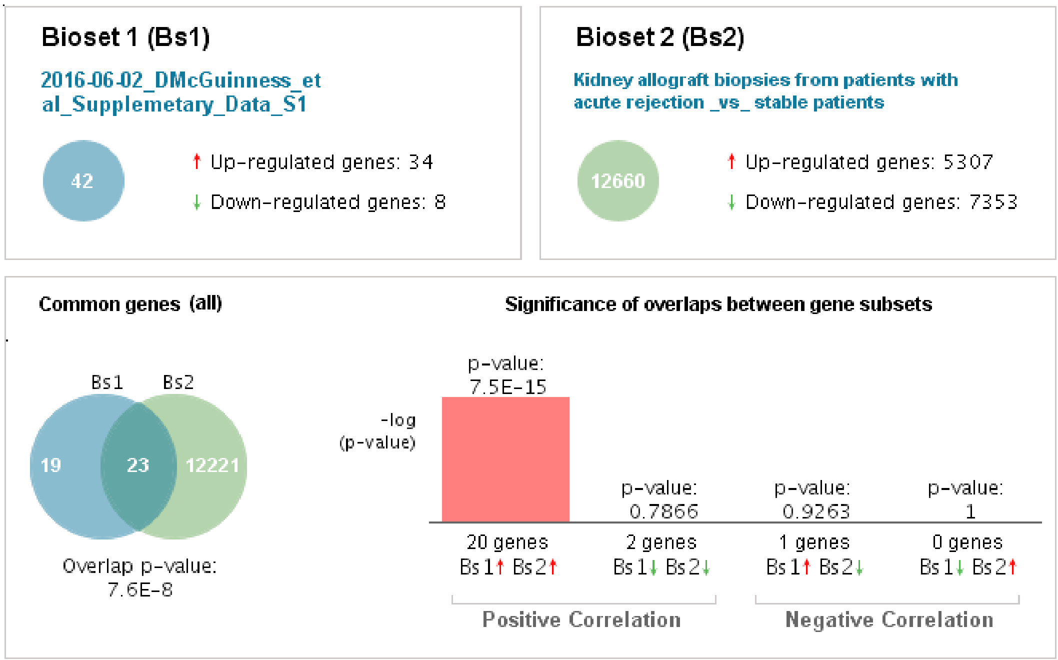

Supplement: Supplementary file 3 [file ACEL-17-e12825-s003.pdf]
