# Supplementary figures and images for: A molecular signature for delayed graft function (part 1 of 2)
Source: Aging Cell. 2018 Aug 9;17(5):e12825. doi: 10.1111/acel.12825 (PMC6156499; doi:10.1111/acel.12825)

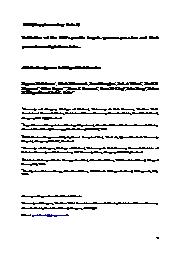

Supplement: Supplementary file 4 [file ACEL-17-e12825-s004.xps › docProps/thumbnail.jpeg]

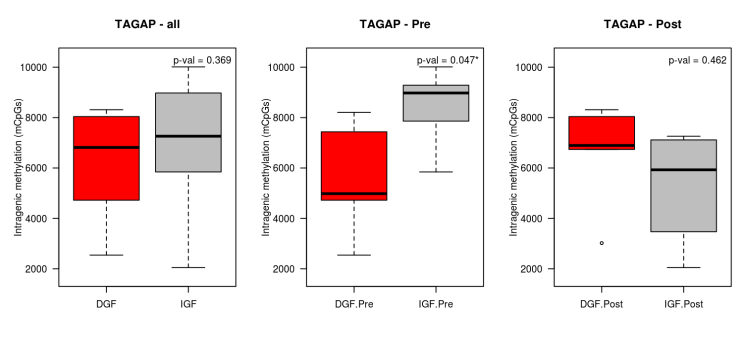

Supplement: Supplementary file 4 [file ACEL-17-e12825-s004.xps › Resources/Images/image_78.png]

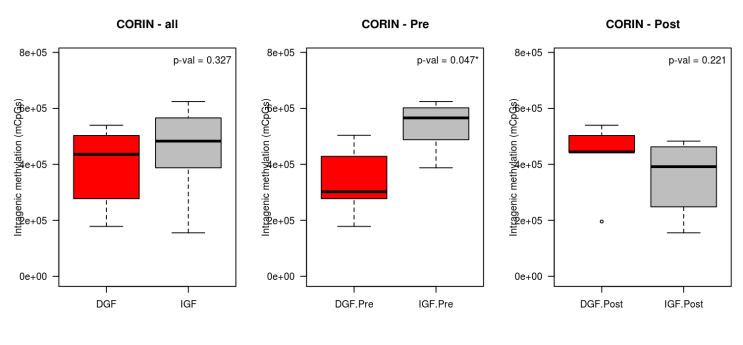

Supplement: Supplementary file 4 [file ACEL-17-e12825-s004.xps › Resources/Images/image_77.png]

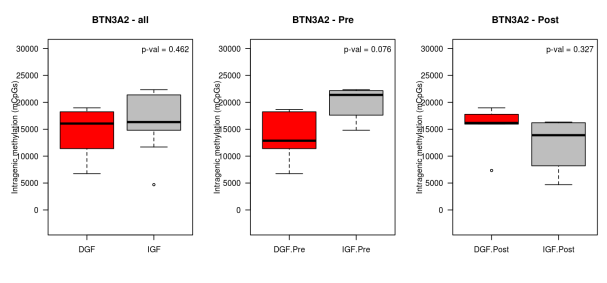

Supplement: Supplementary file 4 [file ACEL-17-e12825-s004.xps › Resources/Images/image_76.png]

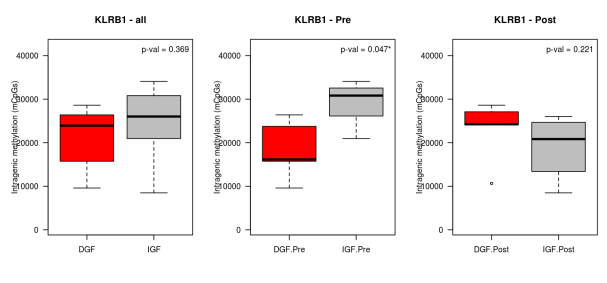

Supplement: Supplementary file 4 [file ACEL-17-e12825-s004.xps › Resources/Images/image_75.png]

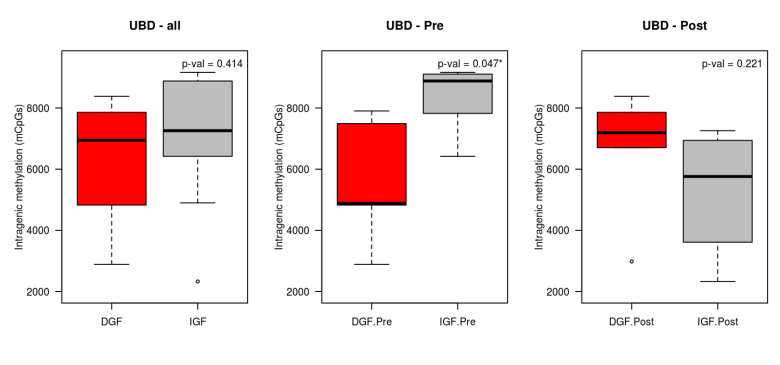

Supplement: Supplementary file 4 [file ACEL-17-e12825-s004.xps › Resources/Images/image_80.png]

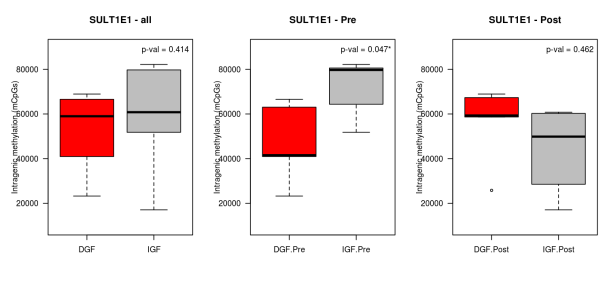

Supplement: Supplementary file 4 [file ACEL-17-e12825-s004.xps › Resources/Images/image_74.png]

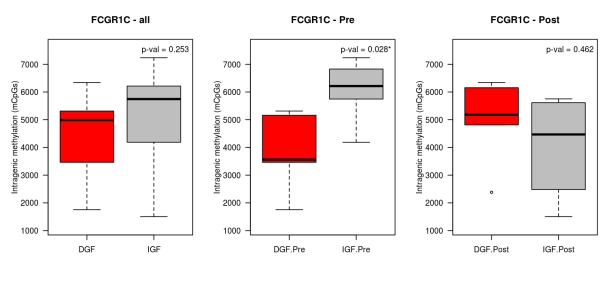

Supplement: Supplementary file 4 [file ACEL-17-e12825-s004.xps › Resources/Images/image_86.png]

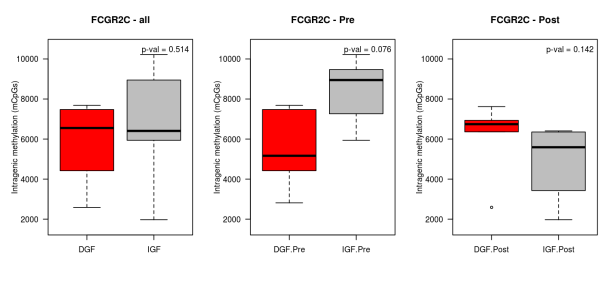

Supplement: Supplementary file 4 [file ACEL-17-e12825-s004.xps › Resources/Images/image_85.png]

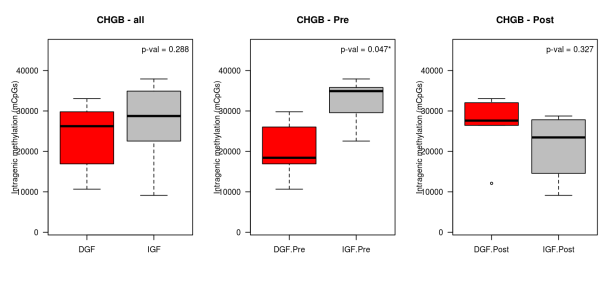

Supplement: Supplementary file 4 [file ACEL-17-e12825-s004.xps › Resources/Images/image_84.png]

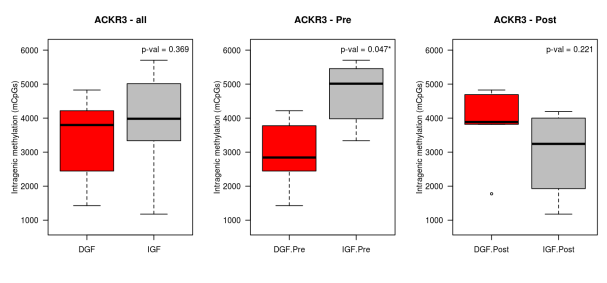

Supplement: Supplementary file 4 [file ACEL-17-e12825-s004.xps › Resources/Images/image_83.png]

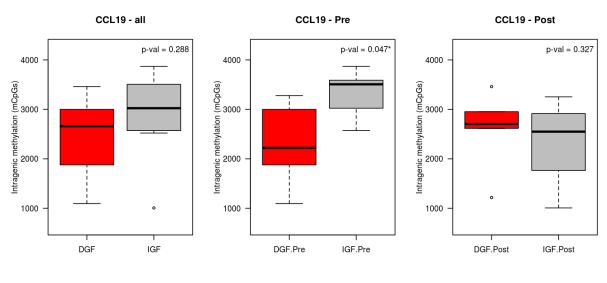

Supplement: Supplementary file 4 [file ACEL-17-e12825-s004.xps › Resources/Images/image_82.png]

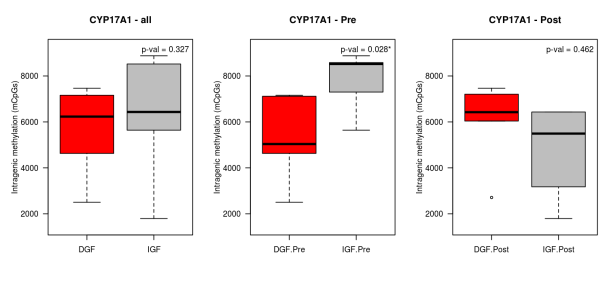

Supplement: Supplementary file 4 [file ACEL-17-e12825-s004.xps › Resources/Images/image_81.png]

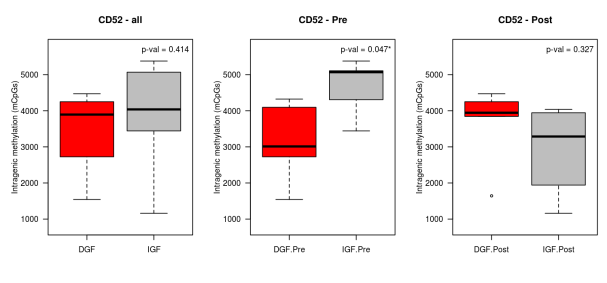

Supplement: Supplementary file 4 [file ACEL-17-e12825-s004.xps › Resources/Images/image_79.png]

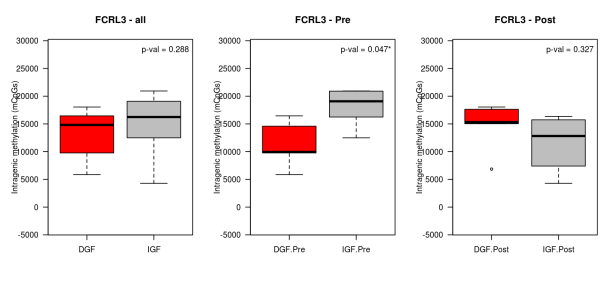

Supplement: Supplementary file 4 [file ACEL-17-e12825-s004.xps › Resources/Images/image_71.png]

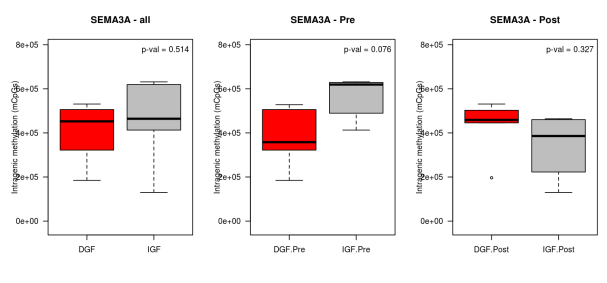

Supplement: Supplementary file 4 [file ACEL-17-e12825-s004.xps › Resources/Images/image_72.png]

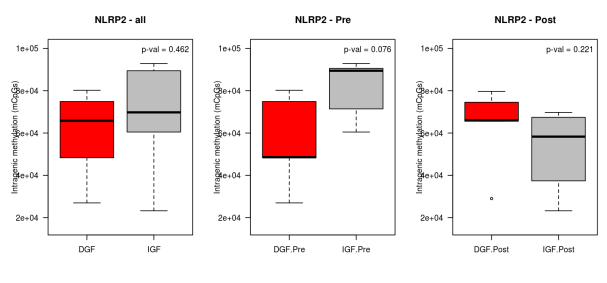

Supplement: Supplementary file 4 [file ACEL-17-e12825-s004.xps › Resources/Images/image_64.png]

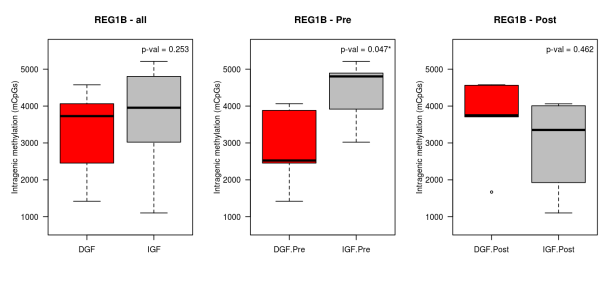

Supplement: Supplementary file 4 [file ACEL-17-e12825-s004.xps › Resources/Images/image_63.png]

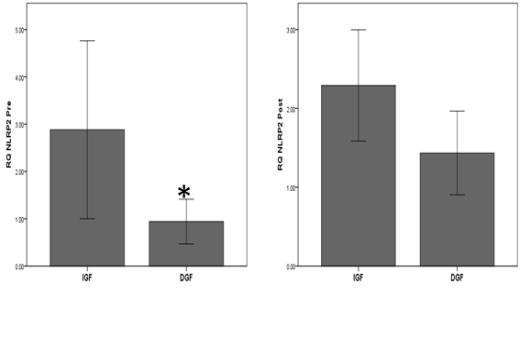

Supplement: Supplementary file 4 [file ACEL-17-e12825-s004.xps › Resources/Images/image_62.png]

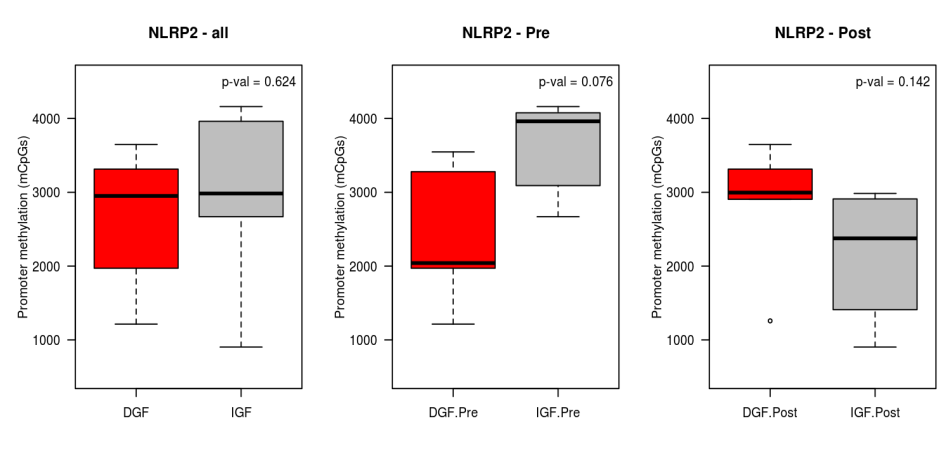

Supplement: Supplementary file 4 [file ACEL-17-e12825-s004.xps › Resources/Images/image_61.png]

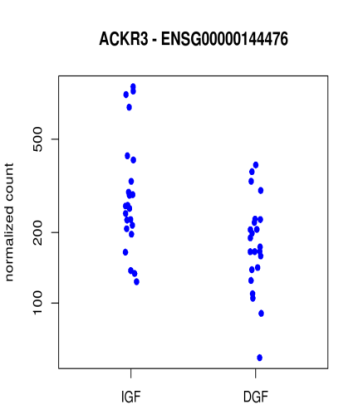

Supplement: Supplementary file 4 [file ACEL-17-e12825-s004.xps › Resources/Images/image_60.png]

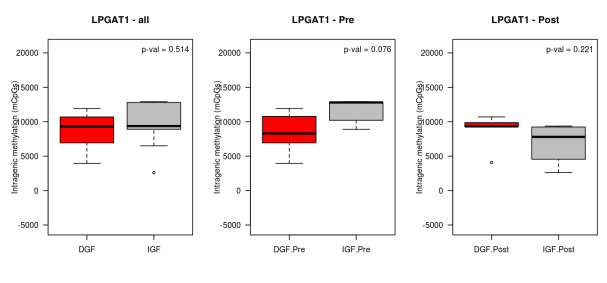

Supplement: Supplementary file 4 [file ACEL-17-e12825-s004.xps › Resources/Images/image_65.png]

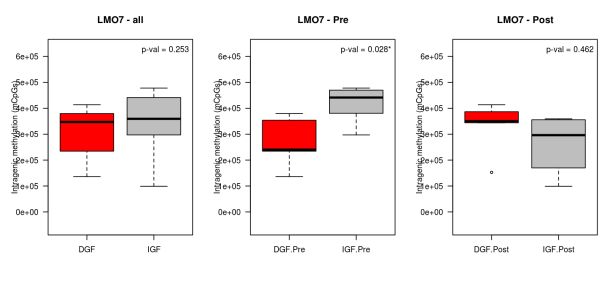

Supplement: Supplementary file 4 [file ACEL-17-e12825-s004.xps › Resources/Images/image_66.png]

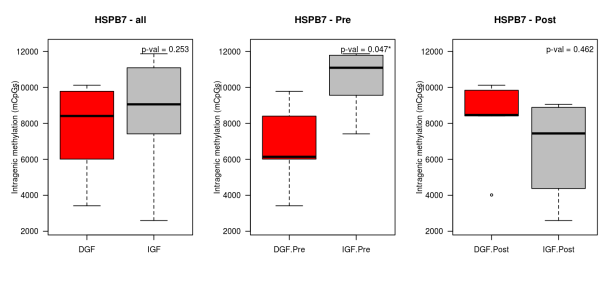

Supplement: Supplementary file 4 [file ACEL-17-e12825-s004.xps › Resources/Images/image_67.png]

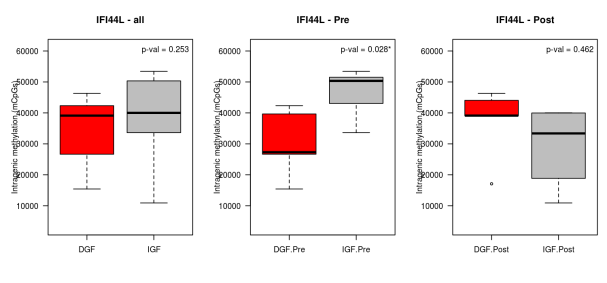

Supplement: Supplementary file 4 [file ACEL-17-e12825-s004.xps › Resources/Images/image_70.png]

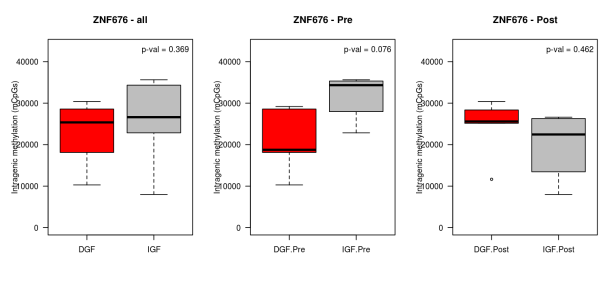

Supplement: Supplementary file 4 [file ACEL-17-e12825-s004.xps › Resources/Images/image_69.png]

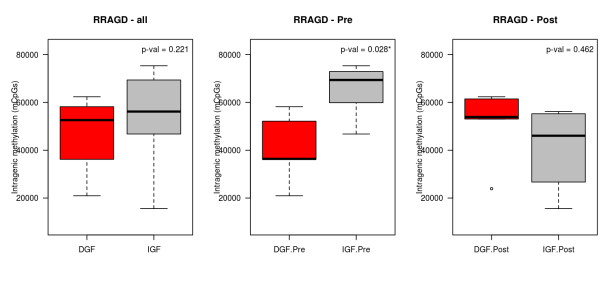

Supplement: Supplementary file 4 [file ACEL-17-e12825-s004.xps › Resources/Images/image_68.png]

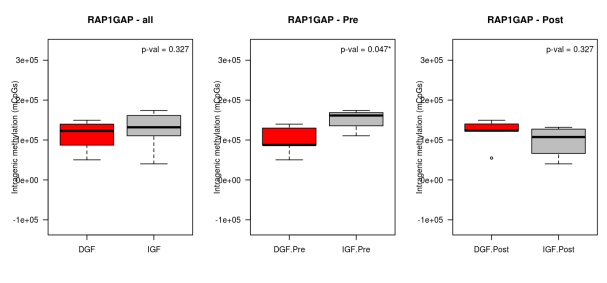

Supplement: Supplementary file 4 [file ACEL-17-e12825-s004.xps › Resources/Images/image_73.png]

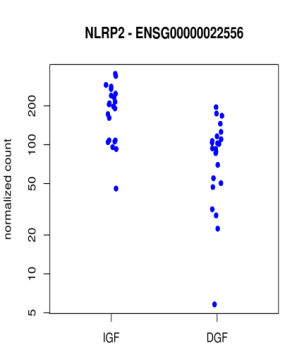

Supplement: Supplementary file 4 [file ACEL-17-e12825-s004.xps › Resources/Images/image_59.png]

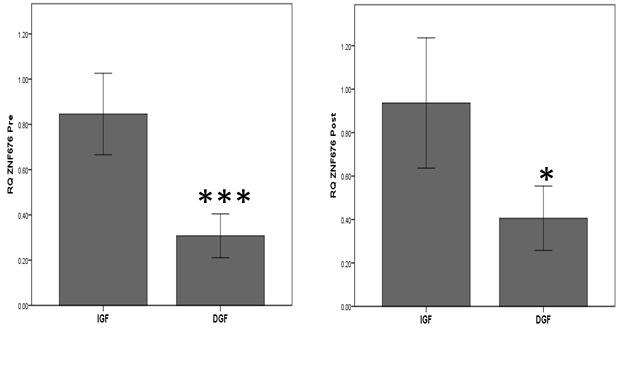

Supplement: Supplementary file 4 [file ACEL-17-e12825-s004.xps › Resources/Images/image_57.png]

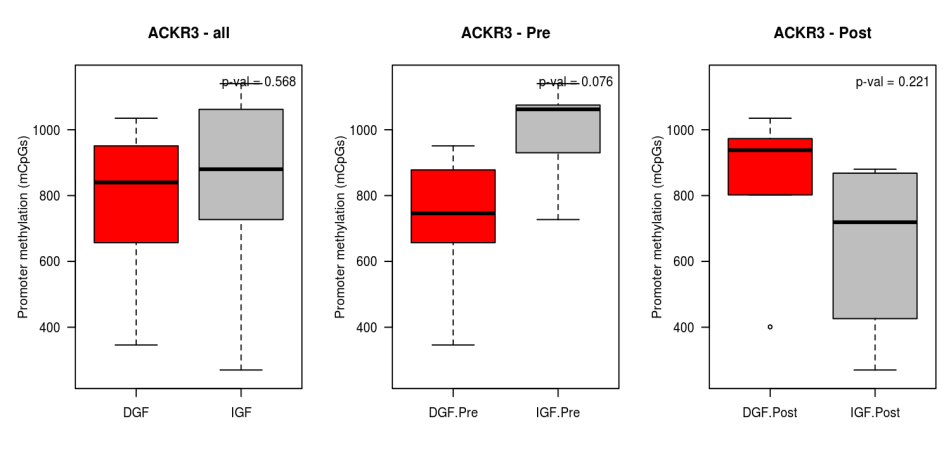

Supplement: Supplementary file 4 [file ACEL-17-e12825-s004.xps › Resources/Images/image_56.png]

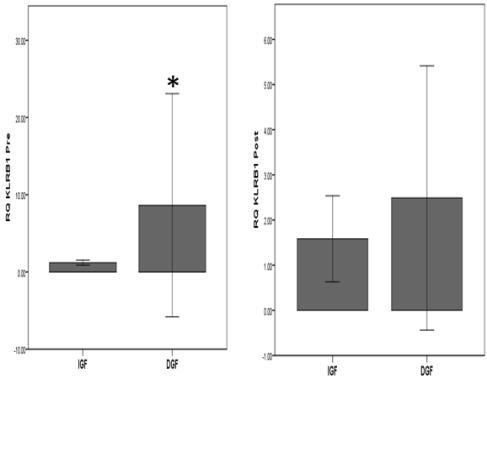

Supplement: Supplementary file 4 [file ACEL-17-e12825-s004.xps › Resources/Images/image_17.png]

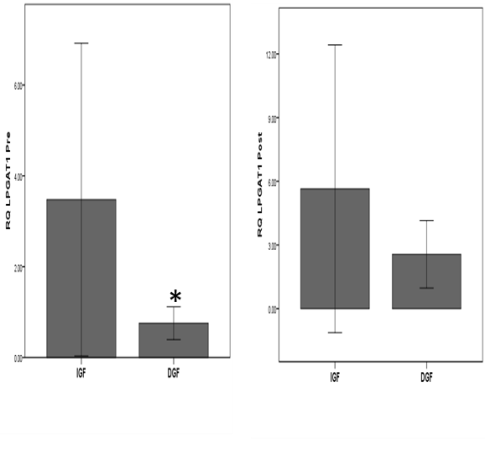

Supplement: Supplementary file 4 [file ACEL-17-e12825-s004.xps › Resources/Images/image_16.png]

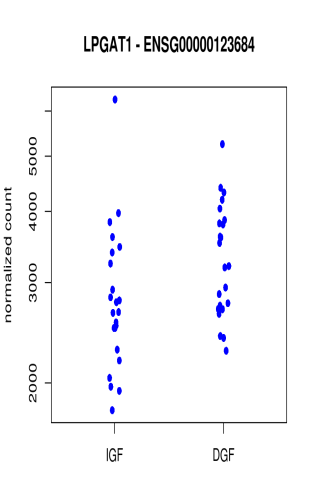

Supplement: Supplementary file 4 [file ACEL-17-e12825-s004.xps › Resources/Images/image_15.png]

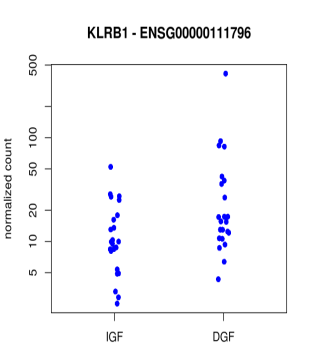

Supplement: Supplementary file 4 [file ACEL-17-e12825-s004.xps › Resources/Images/image_14.png]

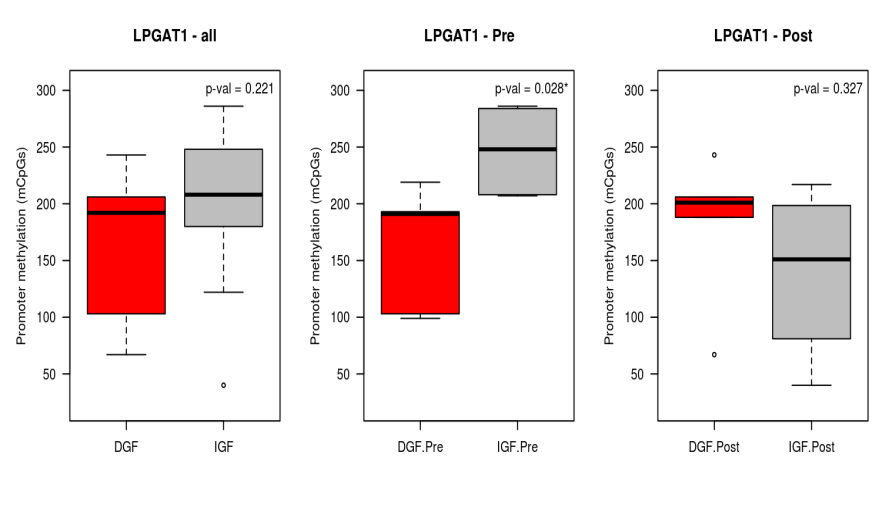

Supplement: Supplementary file 4 [file ACEL-17-e12825-s004.xps › Resources/Images/image_13.png]

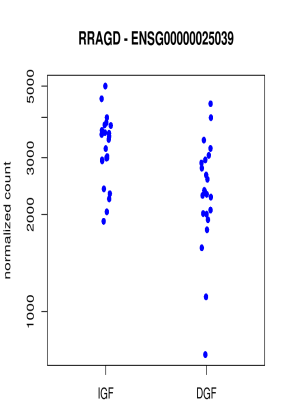

Supplement: Supplementary file 4 [file ACEL-17-e12825-s004.xps › Resources/Images/image_18.png]

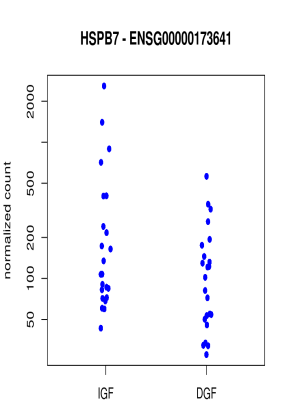

Supplement: Supplementary file 4 [file ACEL-17-e12825-s004.xps › Resources/Images/image_19.png]

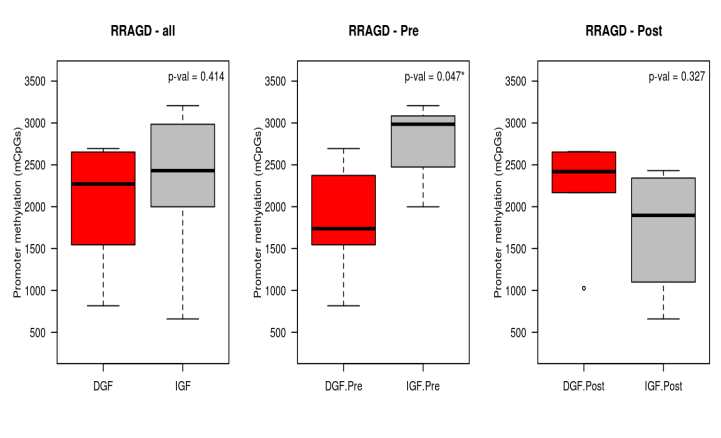

Supplement: Supplementary file 4 [file ACEL-17-e12825-s004.xps › Resources/Images/image_20.png]

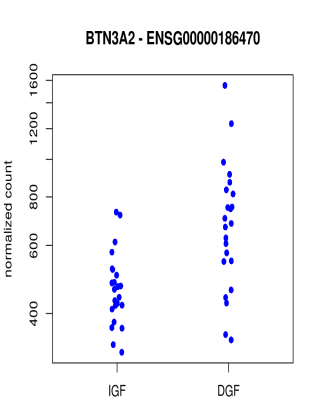

Supplement: Supplementary file 4 [file ACEL-17-e12825-s004.xps › Resources/Images/image_25.png]

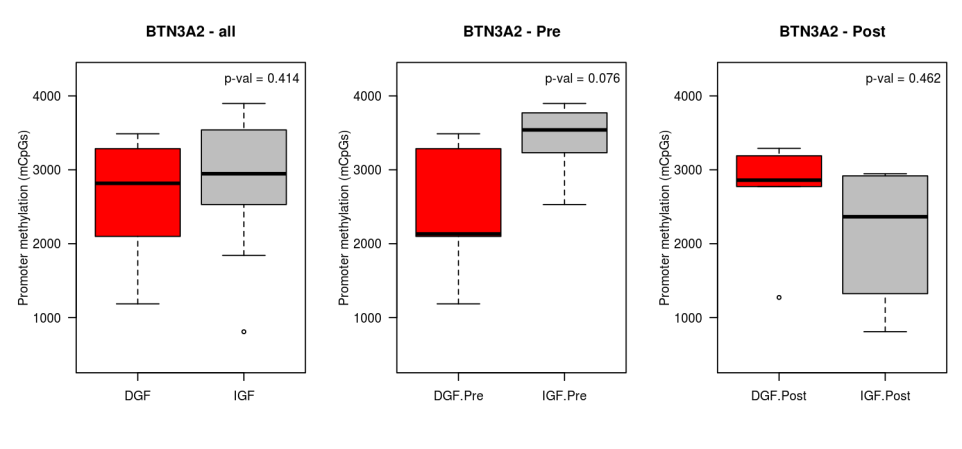

Supplement: Supplementary file 4 [file ACEL-17-e12825-s004.xps › Resources/Images/image_24.png]

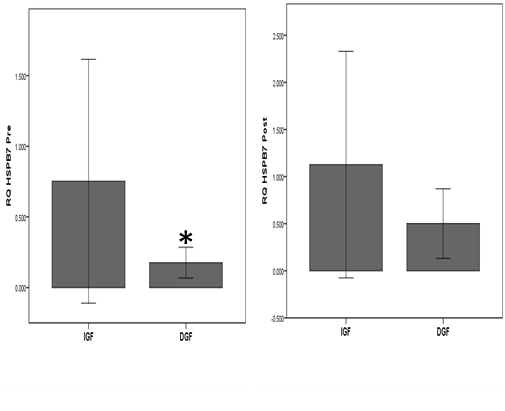

Supplement: Supplementary file 4 [file ACEL-17-e12825-s004.xps › Resources/Images/image_23.png]

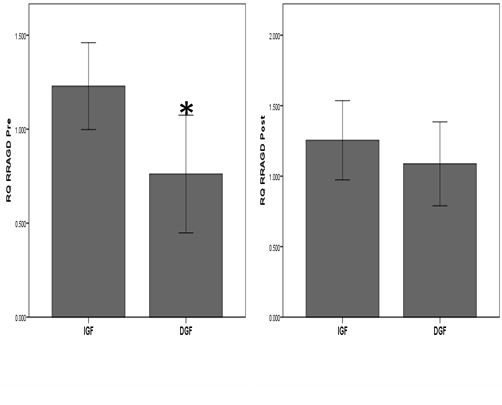

Supplement: Supplementary file 4 [file ACEL-17-e12825-s004.xps › Resources/Images/image_22.png]

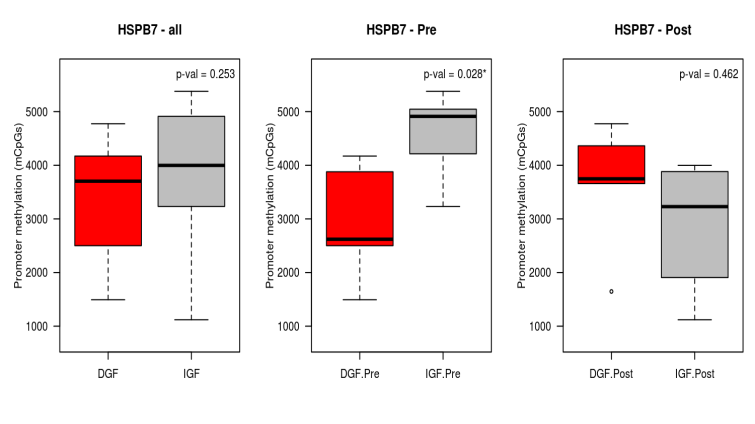

Supplement: Supplementary file 4 [file ACEL-17-e12825-s004.xps › Resources/Images/image_21.png]

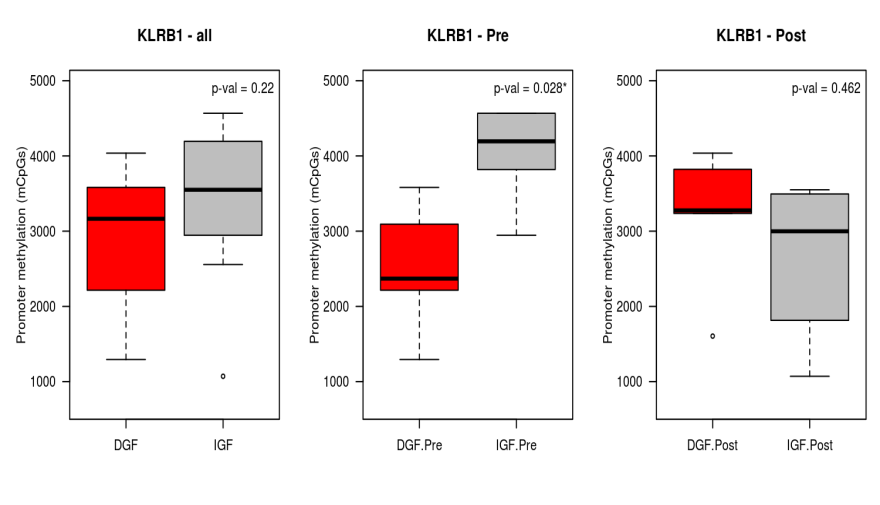

Supplement: Supplementary file 4 [file ACEL-17-e12825-s004.xps › Resources/Images/image_12.png]

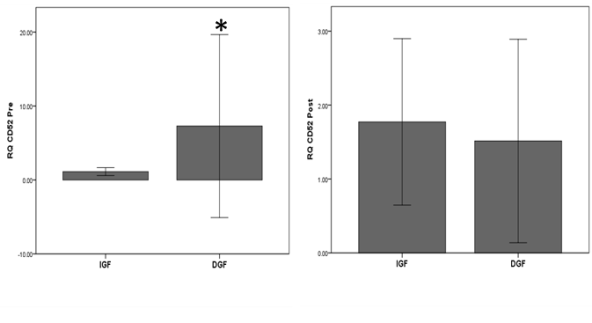

Supplement: Supplementary file 4 [file ACEL-17-e12825-s004.xps › Resources/Images/image_11.png]

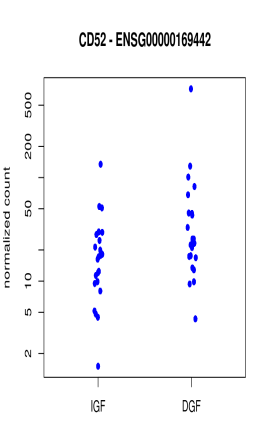

Supplement: Supplementary file 4 [file ACEL-17-e12825-s004.xps › Resources/Images/image_10.png]

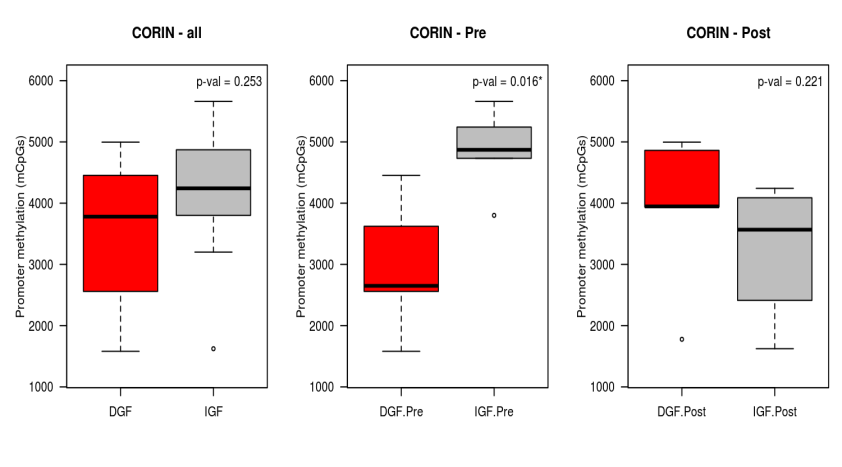

Supplement: Supplementary file 4 [file ACEL-17-e12825-s004.xps › Resources/Images/image_2.png]

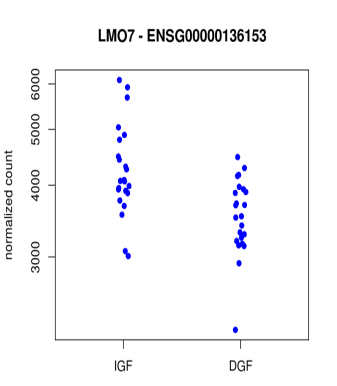

Supplement: Supplementary file 4 [file ACEL-17-e12825-s004.xps › Resources/Images/image_1.png]

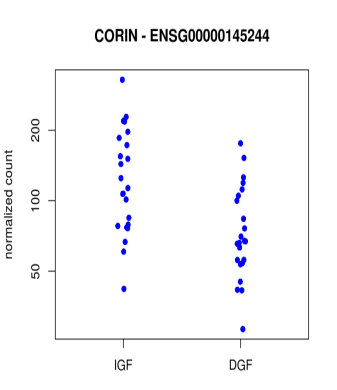

Supplement: Supplementary file 4 [file ACEL-17-e12825-s004.xps › Resources/Images/image_0.png]

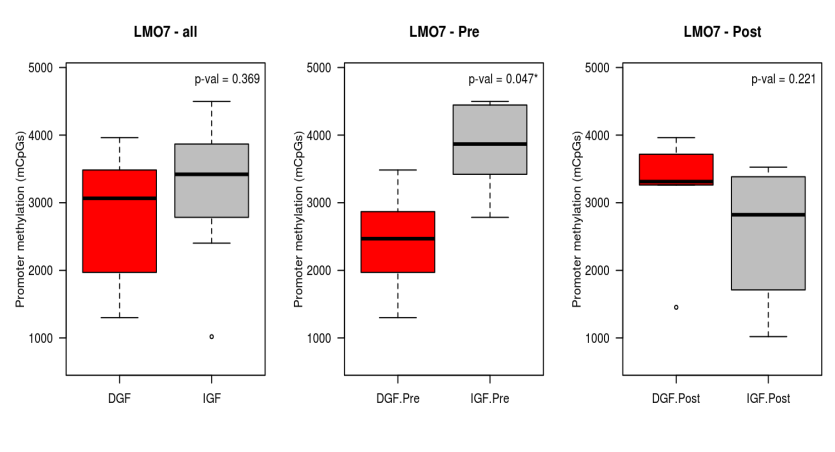

Supplement: Supplementary file 4 [file ACEL-17-e12825-s004.xps › Resources/Images/image_3.png]

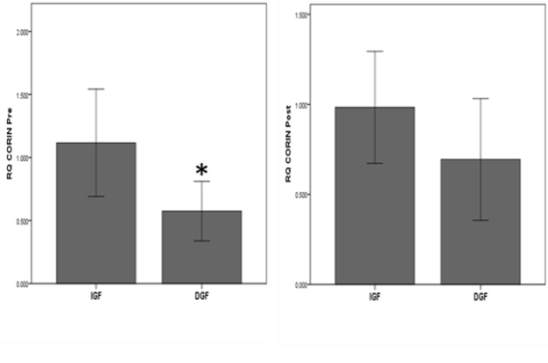

Supplement: Supplementary file 4 [file ACEL-17-e12825-s004.xps › Resources/Images/image_4.png]

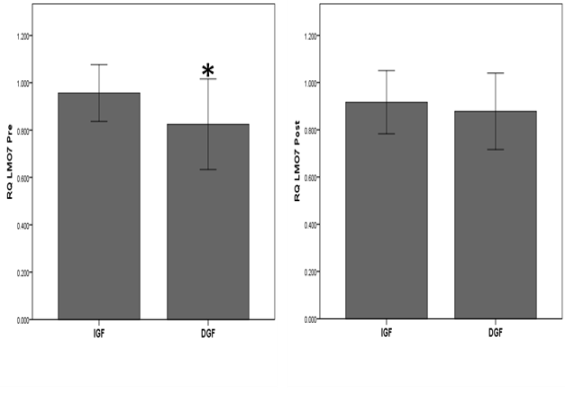

Supplement: Supplementary file 4 [file ACEL-17-e12825-s004.xps › Resources/Images/image_5.png]

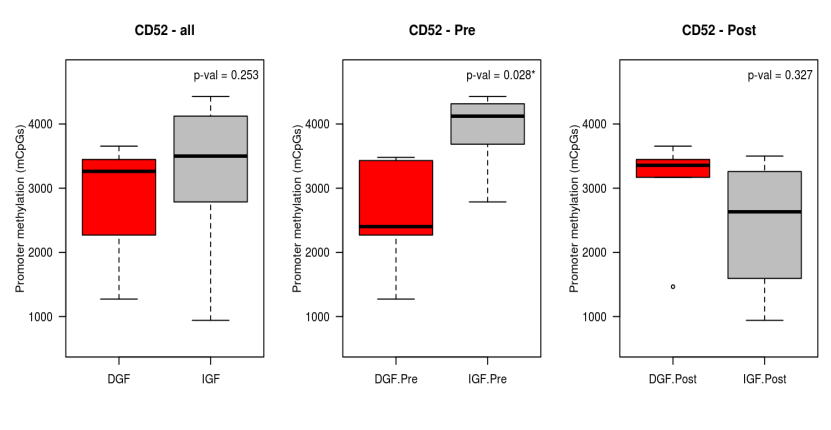

Supplement: Supplementary file 4 [file ACEL-17-e12825-s004.xps › Resources/Images/image_9.png]

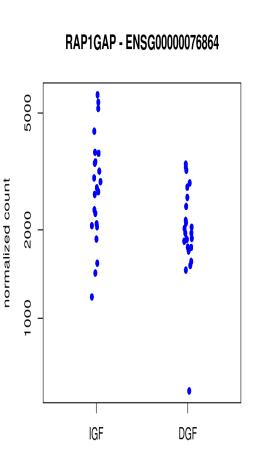

Supplement: Supplementary file 4 [file ACEL-17-e12825-s004.xps › Resources/Images/image_8.png]

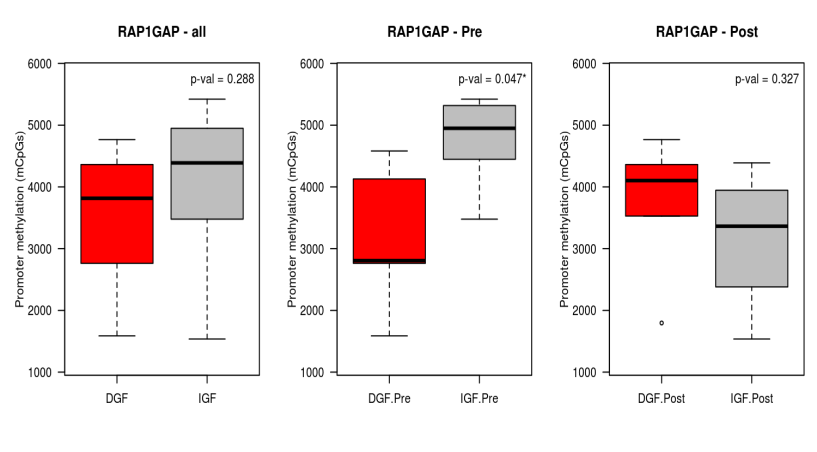

Supplement: Supplementary file 4 [file ACEL-17-e12825-s004.xps › Resources/Images/image_7.png]

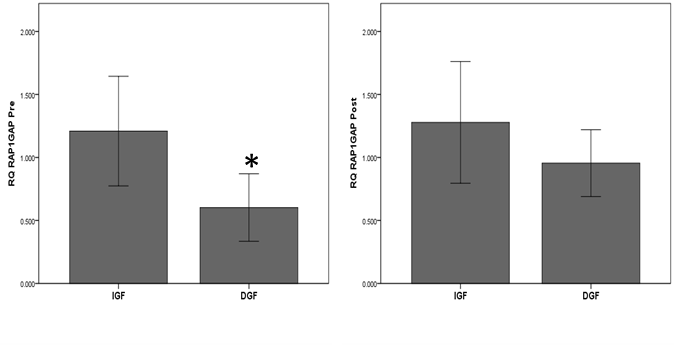

Supplement: Supplementary file 4 [file ACEL-17-e12825-s004.xps › Resources/Images/image_6.png]

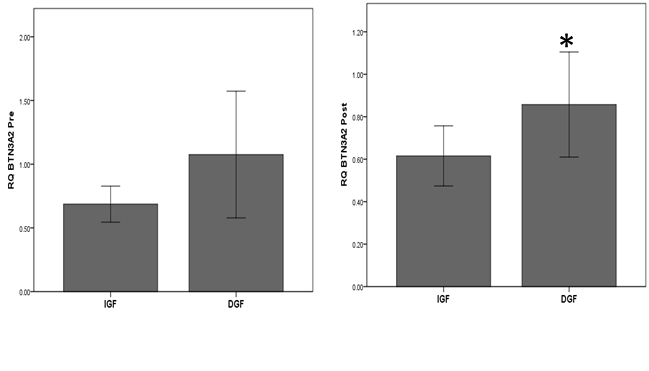

Supplement: Supplementary file 4 [file ACEL-17-e12825-s004.xps › Resources/Images/image_26.png]

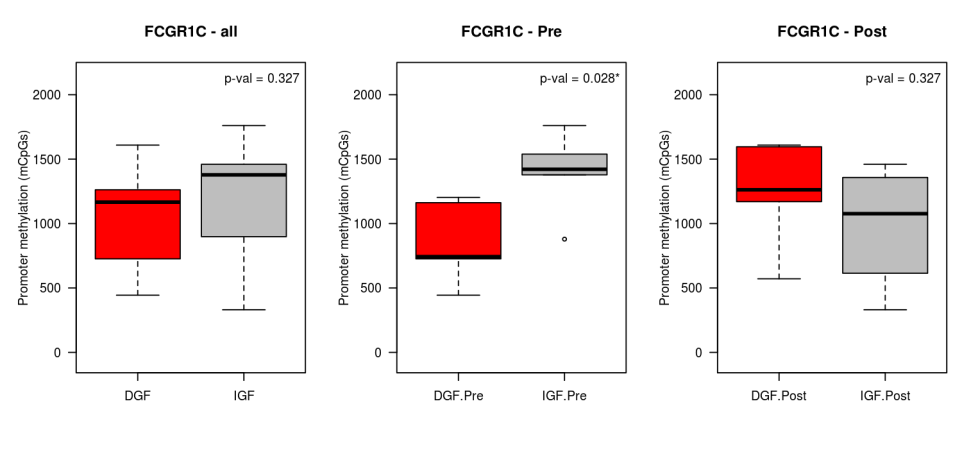

Supplement: Supplementary file 4 [file ACEL-17-e12825-s004.xps › Resources/Images/image_27.png]

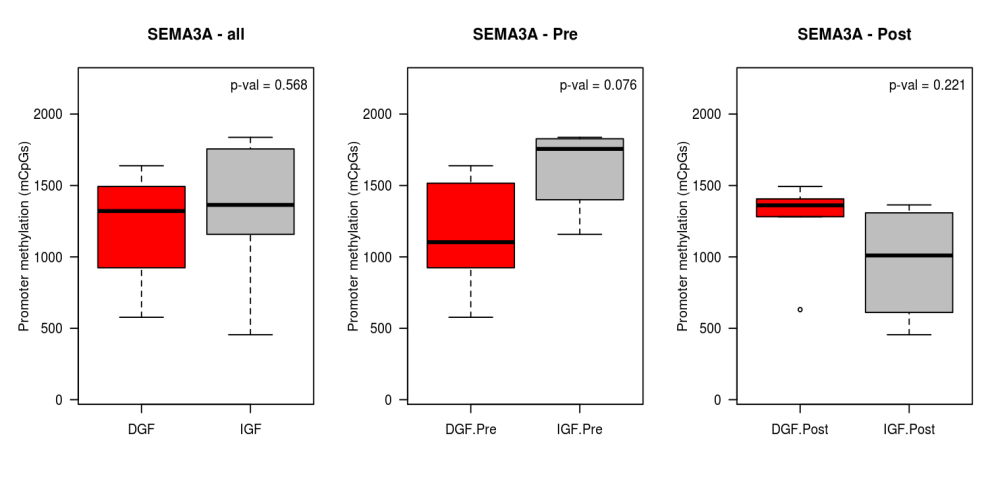

Supplement: Supplementary file 4 [file ACEL-17-e12825-s004.xps › Resources/Images/image_48.png]

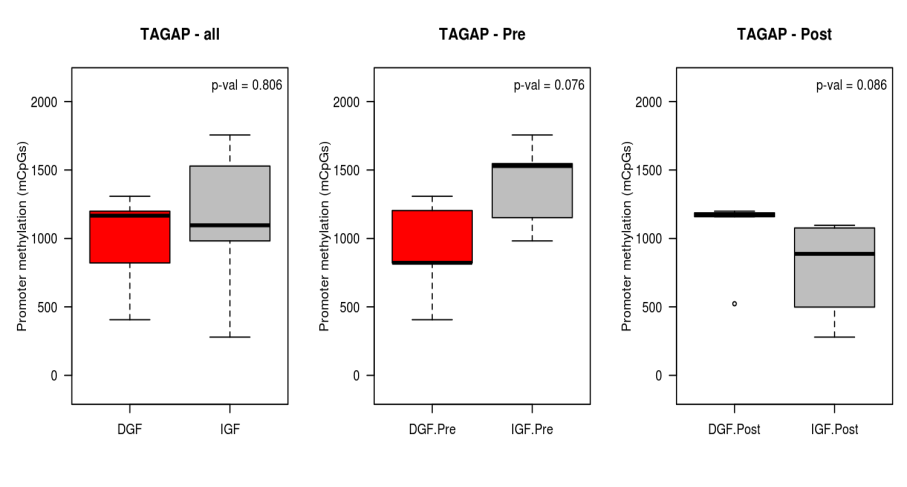

Supplement: Supplementary file 4 [file ACEL-17-e12825-s004.xps › Resources/Images/image_47.png]

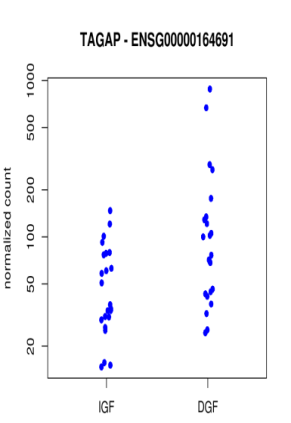

Supplement: Supplementary file 4 [file ACEL-17-e12825-s004.xps › Resources/Images/image_46.png]

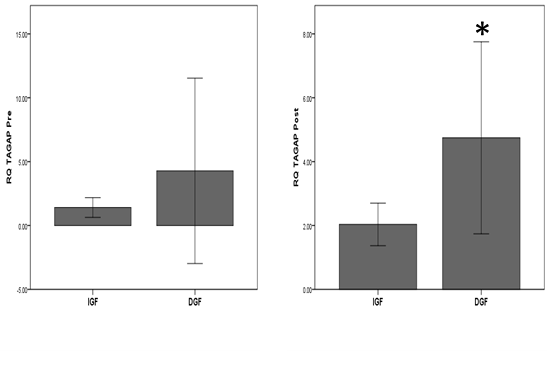

Supplement: Supplementary file 4 [file ACEL-17-e12825-s004.xps › Resources/Images/image_45.png]

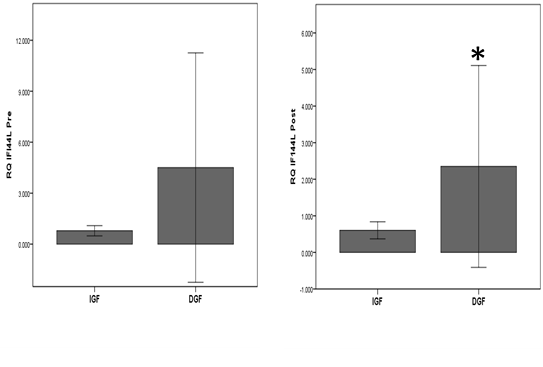

Supplement: Supplementary file 4 [file ACEL-17-e12825-s004.xps › Resources/Images/image_44.png]

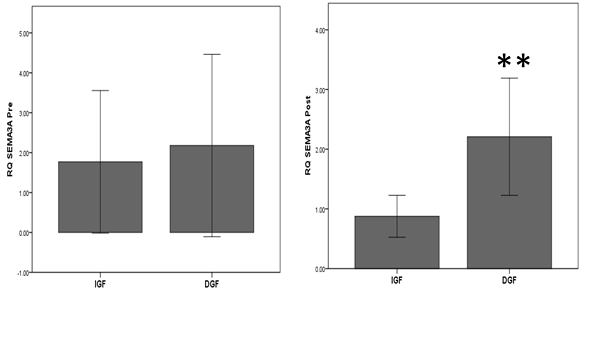

Supplement: Supplementary file 4 [file ACEL-17-e12825-s004.xps › Resources/Images/image_49.png]

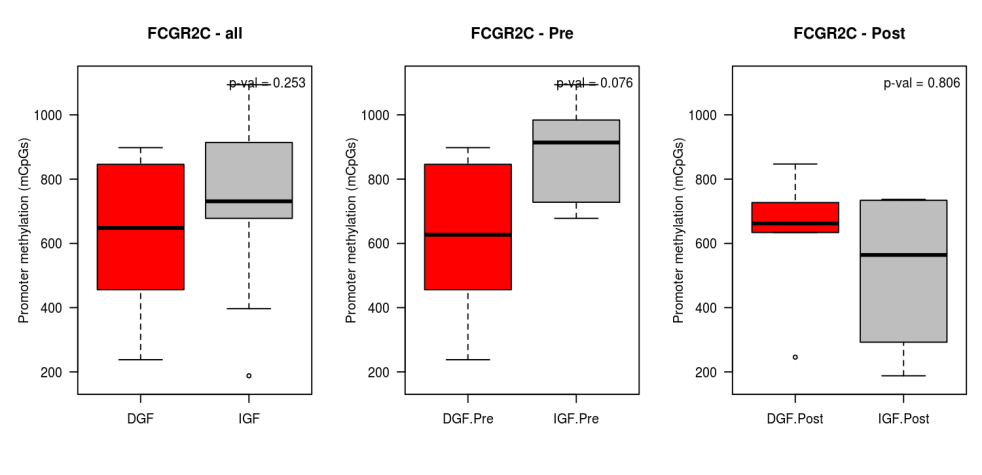

Supplement: Supplementary file 4 [file ACEL-17-e12825-s004.xps › Resources/Images/image_50.png]

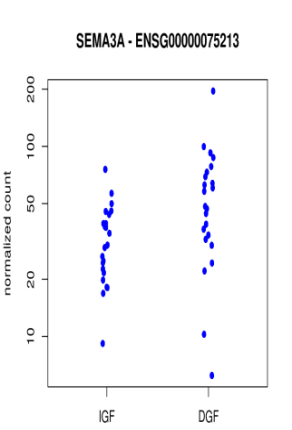

Supplement: Supplementary file 4 [file ACEL-17-e12825-s004.xps › Resources/Images/image_51.png]

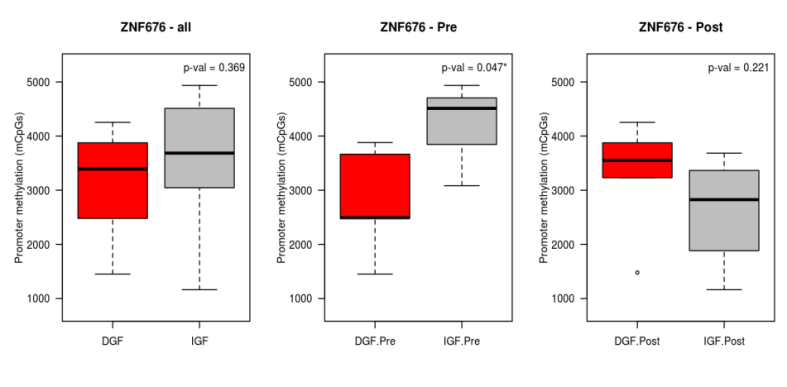

Supplement: Supplementary file 4 [file ACEL-17-e12825-s004.xps › Resources/Images/image_55.png]

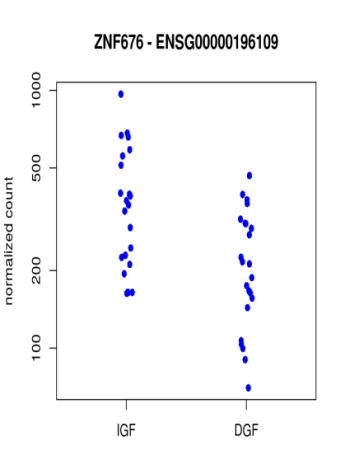

Supplement: Supplementary file 4 [file ACEL-17-e12825-s004.xps › Resources/Images/image_54.png]

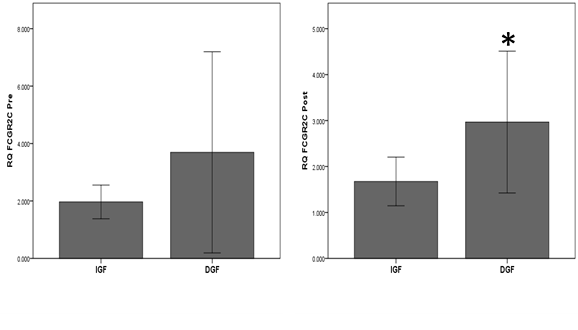

Supplement: Supplementary file 4 [file ACEL-17-e12825-s004.xps › Resources/Images/image_53.png]

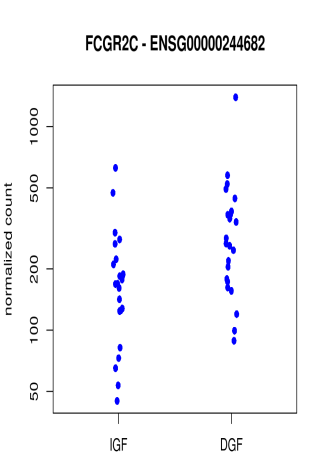

Supplement: Supplementary file 4 [file ACEL-17-e12825-s004.xps › Resources/Images/image_52.png]

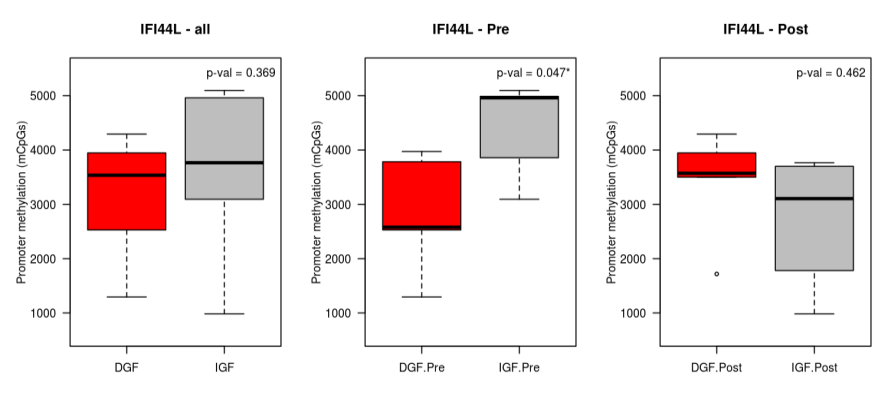

Supplement: Supplementary file 4 [file ACEL-17-e12825-s004.xps › Resources/Images/image_43.png]

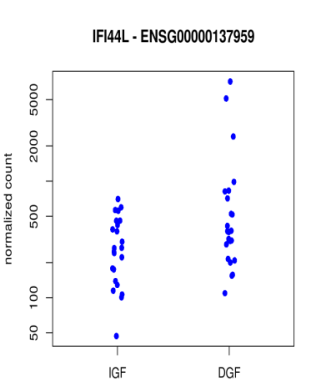

Supplement: Supplementary file 4 [file ACEL-17-e12825-s004.xps › Resources/Images/image_42.png]

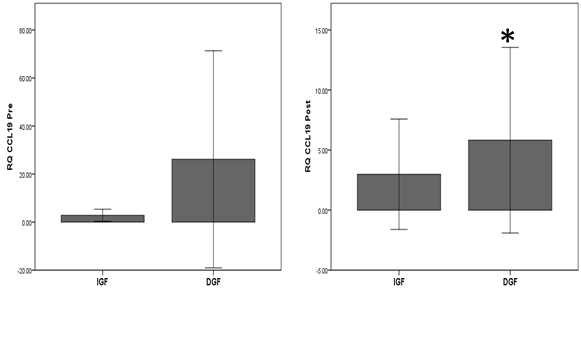

Supplement: Supplementary file 4 [file ACEL-17-e12825-s004.xps › Resources/Images/image_41.png]

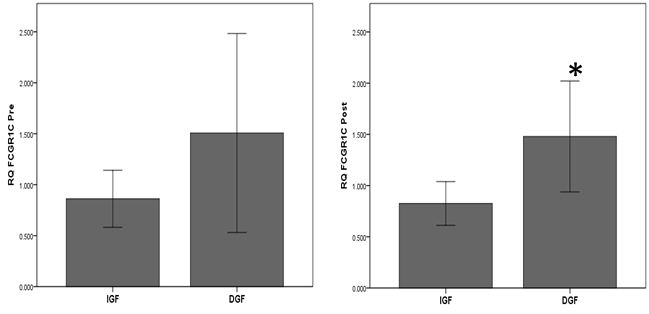

Supplement: Supplementary file 4 [file ACEL-17-e12825-s004.xps › Resources/Images/image_33.png]

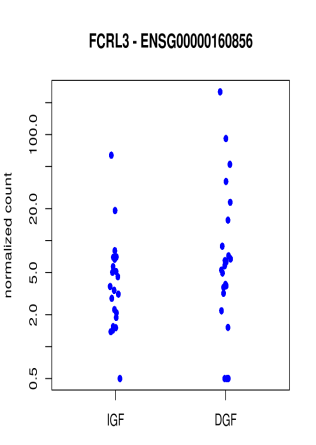

Supplement: Supplementary file 4 [file ACEL-17-e12825-s004.xps › Resources/Images/image_32.png]

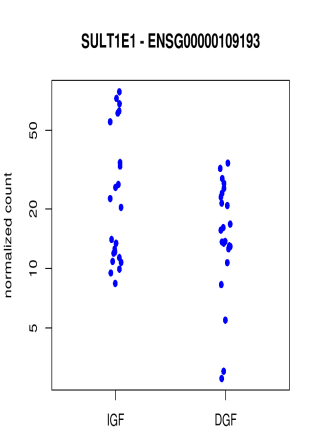

Supplement: Supplementary file 4 [file ACEL-17-e12825-s004.xps › Resources/Images/image_31.png]

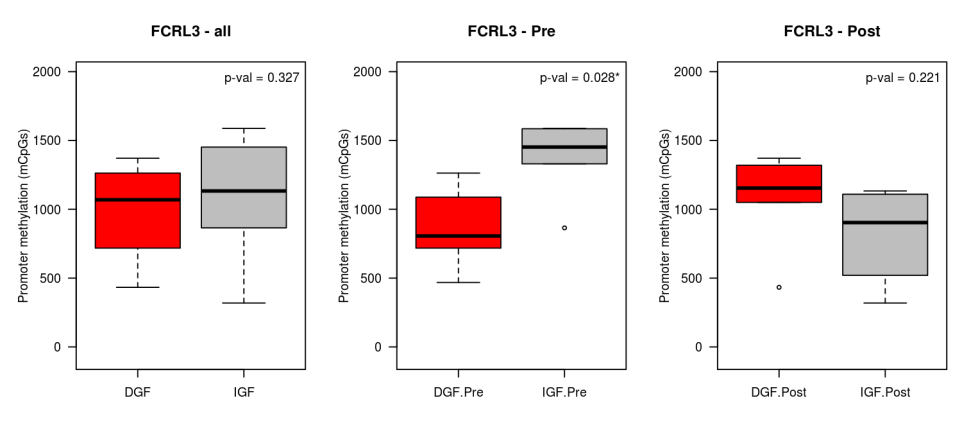

Supplement: Supplementary file 4 [file ACEL-17-e12825-s004.xps › Resources/Images/image_30.png]

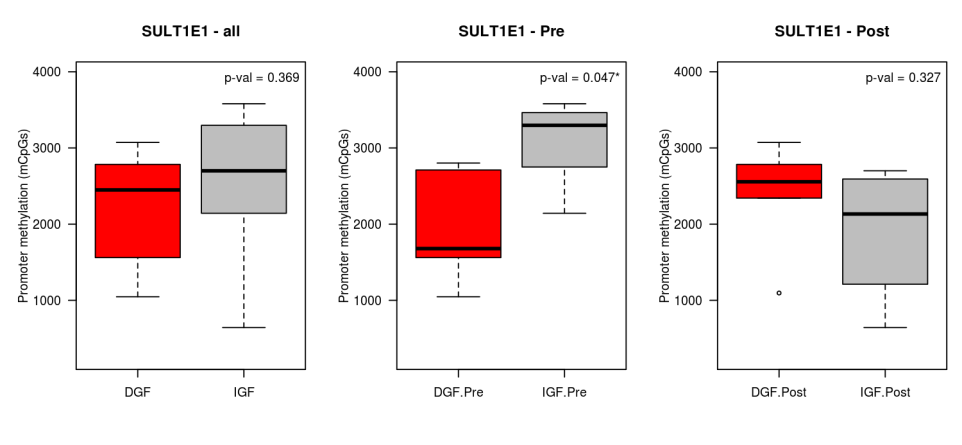

Supplement: Supplementary file 4 [file ACEL-17-e12825-s004.xps › Resources/Images/image_29.png]

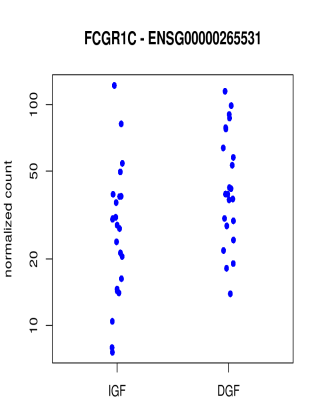

Supplement: Supplementary file 4 [file ACEL-17-e12825-s004.xps › Resources/Images/image_28.png]

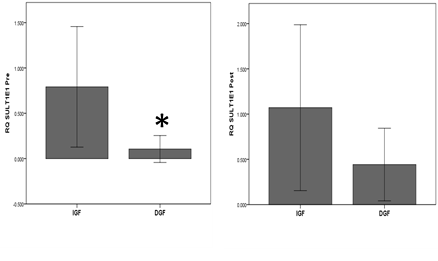

Supplement: Supplementary file 4 [file ACEL-17-e12825-s004.xps › Resources/Images/image_34.png]

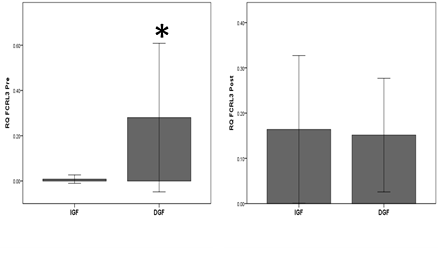

Supplement: Supplementary file 4 [file ACEL-17-e12825-s004.xps › Resources/Images/image_35.png]

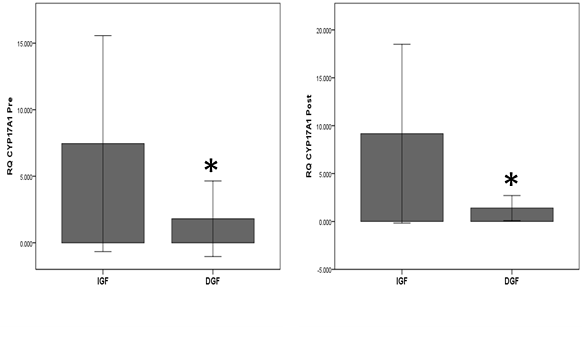

Supplement: Supplementary file 4 [file ACEL-17-e12825-s004.xps › Resources/Images/image_40.png]

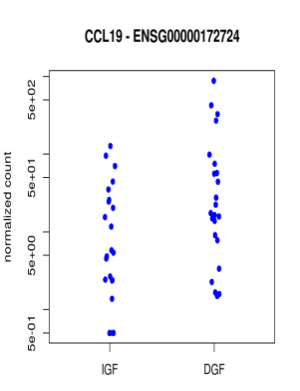

Supplement: Supplementary file 4 [file ACEL-17-e12825-s004.xps › Resources/Images/image_39.png]

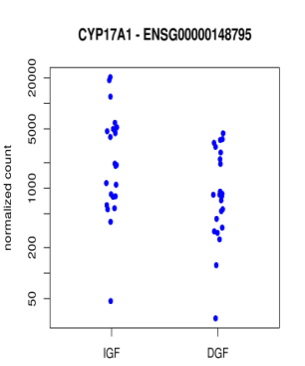

Supplement: Supplementary file 4 [file ACEL-17-e12825-s004.xps › Resources/Images/image_38.png]

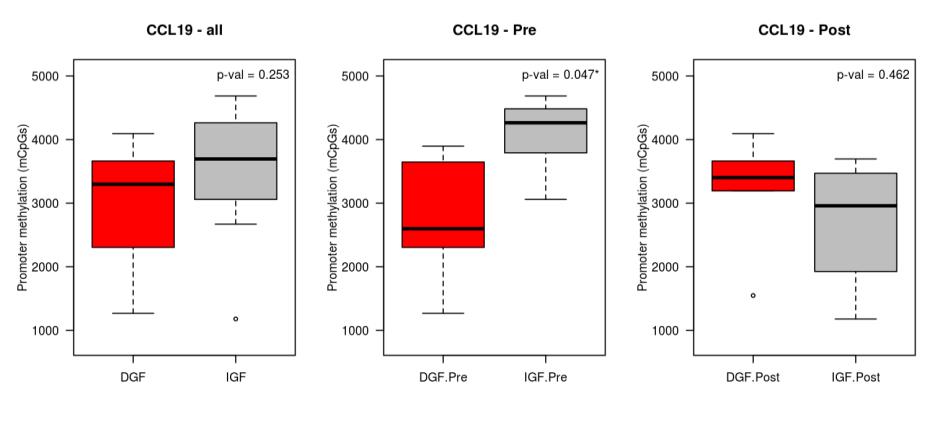

Supplement: Supplementary file 4 [file ACEL-17-e12825-s004.xps › Resources/Images/image_37.jpg]

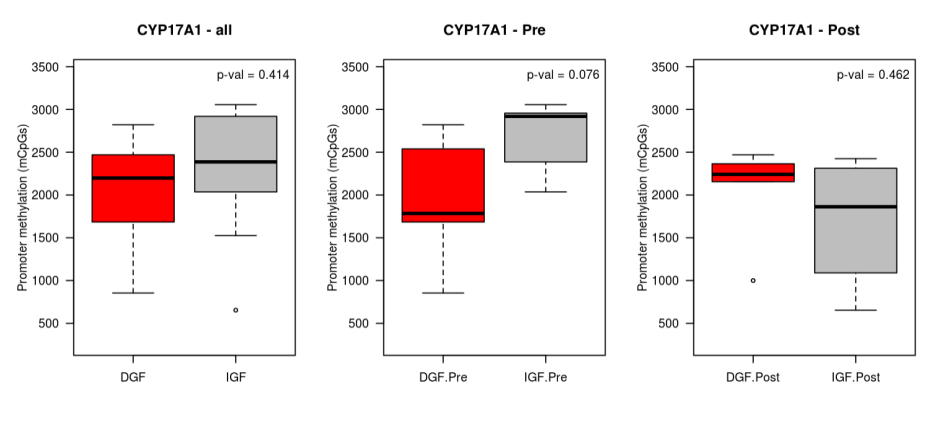

Supplement: Supplementary file 4 [file ACEL-17-e12825-s004.xps › Resources/Images/image_36.png]

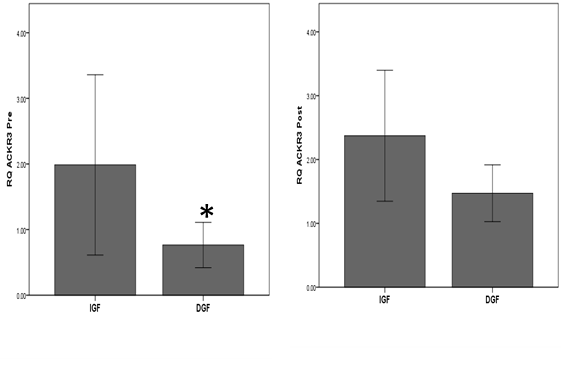

Supplement: Supplementary file 4 [file ACEL-17-e12825-s004.xps › Resources/Images/image_58.png]

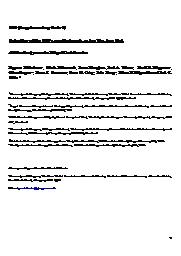

Supplement: Supplementary file 7 [file ACEL-17-e12825-s007.xps › docProps/thumbnail.jpeg]

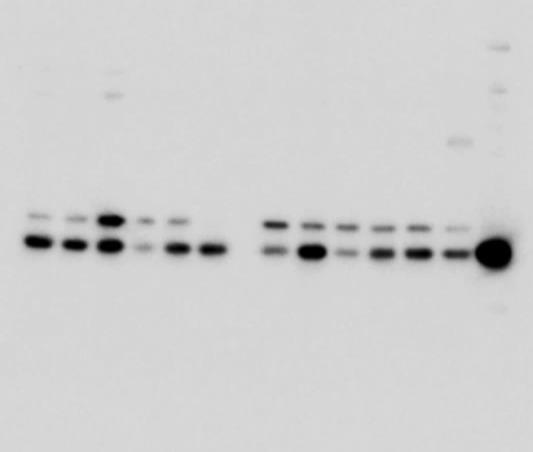

Supplement: Supplementary file 7 [file ACEL-17-e12825-s007.xps › Resources/Images/image_18.jpg]

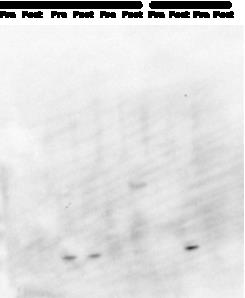

Supplement: Supplementary file 7 [file ACEL-17-e12825-s007.xps › Resources/Images/image_3.jpg]

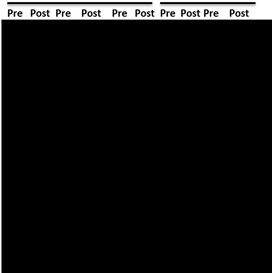

Supplement: Supplementary file 7 [file ACEL-17-e12825-s007.xps › Resources/Images/image_4.png]

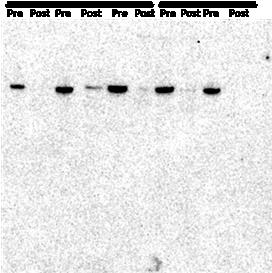

Supplement: Supplementary file 7 [file ACEL-17-e12825-s007.xps › Resources/Images/image_5.jpg]

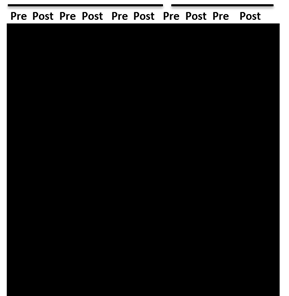

Supplement: Supplementary file 7 [file ACEL-17-e12825-s007.xps › Resources/Images/image_6.png]

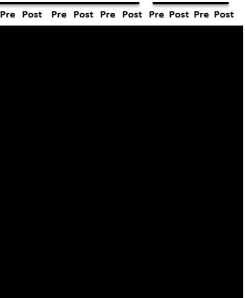

Supplement: Supplementary file 7 [file ACEL-17-e12825-s007.xps › Resources/Images/image_2.png]

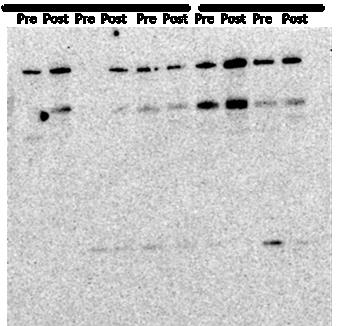

Supplement: Supplementary file 7 [file ACEL-17-e12825-s007.xps › Resources/Images/image_1.jpg]

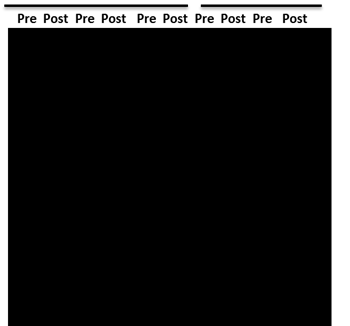

Supplement: Supplementary file 7 [file ACEL-17-e12825-s007.xps › Resources/Images/image_0.png]

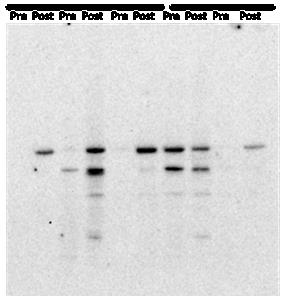

Supplement: Supplementary file 7 [file ACEL-17-e12825-s007.xps › Resources/Images/image_7.jpg]

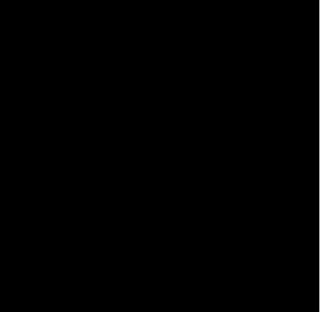

Supplement: Supplementary file 7 [file ACEL-17-e12825-s007.xps › Resources/Images/image_8.png]

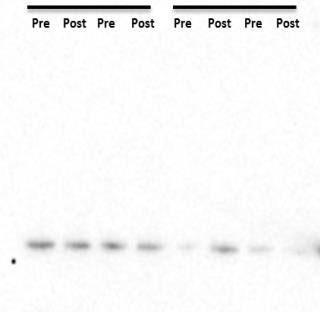

Supplement: Supplementary file 7 [file ACEL-17-e12825-s007.xps › Resources/Images/image_9.jpg]
