# Supplementary material for: A molecular signature for delayed graft function
Source: Aging Cell. 2018 Aug 9;17(5):e12825. doi: 10.1111/acel.12825 (PMC6156499; doi:10.1111/acel.12825)
Supplement: Supplementary file 5 [file ACEL-17-e12825-s005.pdf]

SD4 (Supplementary Data 4). IPA analysis of the differentially expressed targets in relation to perfusion state (post-perfusion samples vs. pre-perfusion samples, S4 Table 1- S4 Table 4). Pathway analysis of differentially expressed targets in relation to perfusion-driven IGF (S4 Table 5- S4 Table 7) and perfusion-driven DGF signature (S4 Table 8- S4 Table 10).

S4 Table 1. The list of the canonical pathways associated with reperfusion injury.

S4 Table 2. The complete list of upstream regulators involved in reperfusion injury.

S4 Table 3. The top networks associated with reperfusion injury.

S4 Table 4. The top regulator effector network associated with reperfusion injury.

S4 Table 5. The list of the canonical pathways associated with IGF pre-perfusion vs post-perfusion.

S4 Table 6. The complete list of upstream regulators involved in IGF pre-perfusion vs post-perfusion.

S4 Table 7. The top networks associated with IGF pre-perfusion vs post-perfusion.

S4 Table 8. The list of the canonical pathways associated with DGF pre-perfusion vs post-perfusion.

S4 Table 9. The complete list of upstream regulators involved in DGF pre-perfusion vs post-perfusion.

S4 Table 10. The top networks associated with DGF pre-perfusion vs post-perfusion.

Canonical Pathways	-log(p-value)	Ratio	z-score
EIF2 Signaling	2.39E01	4.51E-01	-6.351
Protein Ubiquitination Pathway	5.49E00	2.47E-01	NaN
Regulation of eIF4 and p70S6K Signaling	4.81E00	2.74E-01	-1.134
Differential Regulation of Cytokine Production in Intestinal Epithelial Cells by IL-17A and IL-17F	4.78E00	5.22E-01	NaN
Differential Regulation of Cytokine Production in Macrophages and T Helper Cells by IL-17A and IL-17F	4.38E00	5.56E-01	NaN
mTOR Signaling	4.14E00	2.46E-01	0.832
Aldosterone Signaling in Epithelial Cells	4.02E00	2.57E-01	0.577
IL-17A Signaling in Fibroblasts	3.87E00	4E-01	NaN
Glucocorticoid Receptor Signaling	3.85E00	2.22E-01	NaN
Granulocyte Adhesion and Diapedesis	3.78E00	2.43E-01	NaN
Polyamine Regulation in Colon Cancer	3.44E00	4.55E-01	NaN
IL-10 Signaling	3.13E00	2.94E-01	NaN
Role of Osteoblasts, Osteoclasts and Chondrocytes in Rheumatoid Arthritis	3.05E00	2.19E-01	NaN
Dolichyl-diphosphooligosaccharide Biosynthesis	3.03E00	6E-01	NaN
Complement System	2.99E00	3.51E-01	-0.707
IL-6 Signaling	2.96E00	2.5E-01	1.890
Hepatic Fibrosis / Hepatic Stellate Cell Activation	2.88E00	2.24E-01	NaN
BMP signaling pathway	2.87E00	2.76E-01	-1.342
Adipogenesis pathway	2.84E00	2.39E-01	NaN
p53 Signaling	2.76E00	2.55E-01	0.535
PPAR Signaling	2.75E00	2.58E-01	-2.294
Molecular Mechanisms of Cancer	2.66E00	1.95E-01	NaN
B Cell Activating Factor Signaling	2.64E00	3.25E-01	0.632
Agranulocyte Adhesion and Diapedesis	2.61E00	2.17E-01	NaN
Role of Oct4 in Mammalian Embryonic Stem Cell Pluripotency	2.5E00	3.04E-01	-1.000
April Mediated Signaling	2.36E00	3.16E-01	0.577
Sertoli Cell-Sertoli Cell Junction Signaling	2.33E00	2.13E-01	NaN
IL-17A Signaling in Gastric Cells	2.3E00	3.6E-01	NaN

Toll-like Receptor Signaling	2.26E00	2.57E-01	1.155
Unfolded protein response	2.23E00	2.78E-01	NaN
Wnt/β-catenin Signaling	2.23E00	2.13E-01	0.626
Hypoxia Signaling in the Cardiovascular System	2.17E00	2.62E-01	1.890
Role of Macrophages, Fibroblasts and Endothelial Cells in Rheumatoid Arthritis	2.16E00	1.93E-01	NaN
Dendritic Cell Maturation	2.14E00	2.09E-01	1.372
HMGB1 Signaling	2.12E00	2.25E-01	2.746
Actin Nucleation by ARP-WASP Complex	2.07E00	2.68E-01	-1.604
ILK Signaling	2.05E00	2.05E-01	2.000
Phosphatidylcholine Biosynthesis I	2.03E00	5.71E-01	NaN
CD27 Signaling in Lymphocytes	1.99E00	2.69E-01	0.905
GNRH Signaling	1.96E00	2.17E-01	0.756
Circadian Rhythm Signaling	1.92E00	3.03E-01	NaN
Role of Hypercytokinemia/hyperchemokinemia in the Pathogenesis of Influenza	1.9E00	2.79E-01	NaN
IL-1 Signaling	1.89E00	2.31E-01	1.606
TREM1 Signaling	1.86E00	2.4E-01	3.300
Hepatic Cholestasis	1.84E00	2.05E-01	NaN
TNFR2 Signaling	1.84E00	3.1E-01	2.646
Role of IL-17F in Allergic Inflammatory Airway Diseases	1.82E00	2.73E-01	2.714
Airway Pathology in Chronic Obstructive Pulmonary Disease	1.78E00	5E-01	NaN
Mitotic Roles of Polo-Like Kinase	1.75E00	2.42E-01	-1.265
Role of PKR in Interferon Induction and Antiviral Response	1.73E00	2.75E-01	NaN
Putrescine Biosynthesis III	1.71E00	1E00	NaN
Formaldehyde Oxidation II (Glutathione-dependent)	1.71E00	1E00	NaN
Communication between Innate and Adaptive Immune Cells	1.7E00	2.25E-01	NaN
PPARα/RXRα Activation	1.69E00	1.98E-01	-0.378
Atherosclerosis Signaling	1.68E00	2.1E-01	NaN
4-1BB Signaling in T Lymphocytes	1.65E00	2.9E-01	1.134
Signaling by Rho Family GTPases	1.63E00	1.88E-01	-0.156
Pathogenesis of Multiple Sclerosis	1.57E00	4.44E-01	NaN

HIPPO signaling	1.57E00	2.21E-01	1.897
Colorectal Cancer Metastasis Signaling	1.57E00	1.86E-01	1.406
Acute Phase Response Signaling	1.55E00	1.95E-01	1.347
HGF Signaling	1.49E00	2.1E-01	-0.688
CD40 Signaling	1.49E00	2.31E-01	0.000
Role of Cytokines in Mediating Communication between Immune Cells	1.49E00	2.41E-01	NaN
Role of IL-17A in Arthritis	1.49E00	2.41E-01	NaN
RANK Signaling in Osteoclasts	1.47E00	2.16E-01	0.000
RAR Activation	1.47E00	1.89E-01	NaN
TNFR1 Signaling	1.46E00	2.45E-01	2.111
DNA Double-Strand Break Repair by Non-Homologous End Joining	1.45E00	3.57E-01	NaN
Mouse Embryonic Stem Cell Pluripotency	1.42E00	2.11E-01	1.342
Cholecystokinin/Gastrin-mediated Signaling	1.42E00	2.08E-01	2.400
Role of Pattern Recognition Receptors in Recognition of Bacteria and Viruses	1.41E00	2E-01	1.414
GADD45 Signaling	1.4E00	3.16E-01	NaN
IL-9 Signaling	1.4E00	2.65E-01	0.378
NRF2-mediated Oxidative Stress Response	1.4E00	1.89E-01	1.000
Ephrin Receptor Signaling	1.39E00	1.9E-01	-0.229
Wnt/Ca+ pathway	1.37E00	2.32E-01	2.309
fMLP Signaling in Neutrophils	1.37E00	2.04E-01	-0.447
LPS-stimulated MAPK Signaling	1.37E00	2.19E-01	1.000
STAT3 Pathway	1.37E00	2.19E-01	-1.500
Oxidative Phosphorylation	1.33E00	2.02E-01	NaN
Coagulation System	1.33E00	2.57E-01	-0.333
Cell Cycle Regulation by BTG Family Proteins	1.33E00	2.57E-01	1.342
Aryl Hydrocarbon Receptor Signaling	1.3E00	1.93E-01	-0.816
Factors Promoting Cardiogenesis in Vertebrates	1.3E00	2.07E-01	NaN
Rac Signaling	1.3E00	2.02E-01	-1.528

S4Table2. The complete list of activated and inhibited upstream regulators associated with reperfusion injury.

Upstream Regulator	Molecule Type	Activation z-score	p-value of overlap	Mechanistic network
**Activated**				
PDGF BB	complex	7.94	2.69E-30	733 (16)
TNF	cytokine	7.877	1.83E-25	847 (16)
NFkB (complex)	complex	7.565	1.90E-12	727 (13)
Salmonella enterica serotype abortus equi lipopolysaccharide	chemical toxicant	7.53	2.32E-16	651 (18)
lipopolysaccharide	chemical drug	7.424	1.04E-17	690 (14)
IL1B	cytokine	7.303	2.64E-20	612 (13)
RICTOR	other	6.862	3.01E-15	897 (21)
camptothecin	chemical drug	6.644	6.34E-13	615 (13)
poly rI:rC-RNA	biologic drug	6.134	2.39E-12	599 (13)
E. coli B5 lipopolysaccharide	chemical - endogenous non-mammalian	6.12	1.02E-10	573 (13)
NUPR1	transcription regulator	6.102	7.01E-08	615 (7)
TLR3	transmembrane receptor	6.087	1.68E-07	434 (14)
CREB1	transcription regulator	5.968	4.21E-14	680 (21)
TLR7	transmembrane receptor	5.834	1.64E-05	415 (12)
thapsigargin	chemical toxicant	5.757	1.53E-07	633 (21)
MYD88	other	5.741	7.27E-10	594 (14)
CD 437	chemical drug	5.703	1.11E-14	674 (17)
TREM1	transmembrane receptor	5.662	2.52E-14	576 (15)
salmonella minnesota R595 lipopolysaccharides	chemical - endogenous non-mammalian	5.644	6.71E-12	660 (14)
RAF1	kinase	5.621	2.98E-08	631 (19)

ionomycin	chemical reagent	5.614	3.91E-05	734 (17)
TLR2	transmembrane receptor	5.575	2.13E-05	542 (13)
leukotriene D4	chemical - endogenous mammalian	5.571	1.64E-22	547 (18)
IFNG	cytokine	5.539	1.38E-14	577 (12)
TLR4	transmembrane receptor	5.483	2.05E-07	600 (14)
Ca2+	chemical - endogenous mammalian	5.454	1.88E-08	647 (18)
ST1926	chemical drug	5.43	1.77E-14	
HGF	growth factor	5.359	2.94E-09	702 (18)
P38 MAPK	group	5.342	4.42E-11	547 (14)
TICAM1	other	5.297	8.58E-10	412 (14)
peptidoglycan	chemical - endogenous non-mammalian	5.095	5.05E-06	355 (11)
TLR9	transmembrane receptor	5.048	2.83E-06	522 (13)
phorbol myristate acetate	chemical drug	5.025	9.29E-10	852 (17)
IL1	group	5.013	4.90E-04	670 (14)
ERBB2	kinase	4.988	2.13E-10	760 (17)
gentamicin	chemical drug	4.957	6.08E-07	611 (18)
F2	peptidase	4.954	6.50E-08	673 (15)
A23187	chemical reagent	4.929	2.22E-05	611 (17)
cigarette smoke	chemical toxicant	4.883	2.43E-06	711 (19)
Vegf	group	4.854	3.56E-07	490 (15)
dalfampridine	chemical drug	4.796	5.13E-14	558 (18)
cyclic AMP	chemical - endogenous mammalian	4.756	2.50E-05	683 (19)
tributyrin	chemical drug	4.686	7.28E-08	821 (24)
RELA	transcription regulator	4.677	5.99E-11	749 (14)
5-O-mycolyl-beta-araf-(1->2)-5-O-mycolyl-alpha-araf-(1->1')-glycerol	chemical - endogenous non-mammalian	4.667	3.39E-11	532 (11)
Tlr	group	4.65	6.62E-06	527 (15)
ERK	group	4.632	5.30E-13	545 (15)
IL1A	cytokine	4.63	1.46E-05	582 (13)

CSF2	cytokine	4.624	4.65E-15	637 (16)
IL6	cytokine	4.592	2.10E-07	648 (15)
resiquimod	chemical drug	4.567	7.29E-10	416 (13)
bicuculline	chemical - endogenous non-mammalian	4.546	2.43E-13	674 (23)
trovafloxacin	chemical drug	4.536	1.08E-08	565 (12)
STAT4	transcription regulator	4.533	1.66E-11	610 (14)
ERK1/2	group	4.528	2.16E-08	801 (17)
etoposide	chemical drug	4.512	1.60E-06	697 (15)
oblimersen	biologic drug	4.461	9.25E-06	
forskolin	chemical toxicant	4.441	2.69E-13	627 (18)
IL2	cytokine	4.433	2.40E-07	569 (13)
hydrogen peroxide	chemical - endogenous mammalian	4.424	4.22E-09	719 (16)
SAMSN1	other	4.379	1.47E-03	470 (12)
E. coli lipopolysaccharide	chemical - endogenous non-mammalian	4.369	3.40E-08	375 (12)
MAPK14	kinase	4.324	5.29E-04	447 (17)
CpG ODN 1668	chemical reagent	4.249	1.27E-05	384 (15)
kainic acid	chemical toxicant	4.238	9.70E-10	625 (21)
cocaine	chemical drug	4.219	5.23E-05	572 (18)
EGFR	kinase	4.196	6.40E-10	699 (16)
EGF	growth factor	4.174	1.66E-12	711 (15)
CTNNB1	transcription regulator	4.158	5.02E-05	759 (20)
FGF2	growth factor	4.13	3.15E-09	715 (18)
SASH1	other	4.123	1.02E-02	
GnRH-A	chemical reagent	4.117	1.43E-15	581 (18)
IL17A	cytokine	4.108	5.29E-09	588 (13)
NRG1	other	4.089	2.93E-09	581 (15)
Ras	group	4.089	2.43E-06	688 (19)
Pam3-Cys-Ser-Lys4	chemical reagent	4.068	8.18E-07	396 (13)

5-fluorouracil	chemical drug	4.028	9.77E-18	866 (22)
PTPRJ	phosphatase	4.025	1.07E-06	691 (21)
Cg	complex	4.006	1.73E-06	739 (18)
enterotoxin B	biologic drug	4	4.44E-03	576 (12)
fatty acid	chemical - endogenous mammalian	3.968	1.72E-03	620 (17)
FOXL2	transcription regulator	3.96	3.31E-10	431 (8)
TNFSF11	cytokine	3.952	9.54E-09	638 (16)
TCR	complex	3.939	2.92E-08	639 (14)
HIF1A	transcription regulator	3.93	4.58E-10	519 (16)
methyl methanesulfonate	chemical toxicant	3.921	1.58E-05	599 (15)
CCL5	cytokine	3.92	1.08E-08	553 (16)
ECSIT	transcription regulator	3.899	2.69E-08	344 (12)
PRKCE	kinase	3.896	9.91E-05	573 (18)
5-azacytidine	chemical drug	3.892	2.32E-05	765 (21)
L-dopa	chemical - endogenous mammalian	3.88	4.15E-03	656 (17)
DOCK8	other	3.873	3.48E-02	
deferoxamine	chemical drug	3.871	1.58E-08	480 (14)
PAF1	other	3.86	7.66E-04	
TNFRSF1A	transmembrane receptor	3.858	4.10E-04	512 (11)
E. coli B4 lipopolysaccharide	chemical toxicant	3.833	4.99E-03	461 (16)
IGF1	growth factor	3.821	1.88E-07	906 (22)
imiquimod	chemical drug	3.797	5.72E-05	408 (15)
CREM	transcription regulator	3.783	1.19E-07	600 (18)
IL5	cytokine	3.769	6.35E-09	755 (17)
carrageenan	chemical - endogenous non-mammalian	3.763	3.64E-09	558 (14)
Fcer1	complex	3.763	9.94E-09	678 (18)
NFATC2	transcription regulator	3.755	6.18E-05	759 (18)
mycophenolic acid	chemical drug	3.752	5.40E-08	487 (17)
KITLG	growth factor	3.746	1.10E-05	443 (15)

Jnk	group	3.743	3.42E-10	654 (17)
IL12 (complex)	complex	3.74	2.54E-04	455 (14)
palmitic acid	chemical - endogenous mammalian	3.718	9.90E-05	560 (14)
PGR	ligand-dependent nuclear receptor	3.699	2.49E-06	831 (19)
Ap1	complex	3.67	1.28E-05	616 (15)
MAP2K1/2	group	3.669	4.99E-08	600 (18)
CAMP	other	3.659	1.11E-07	393 (15)
F2RL1	g-protein coupled receptor	3.649	4.77E-05	465 (20)
TAC1	other	3.638	5.21E-03	464 (18)
doxorubicin	chemical drug	3.632	1.14E-06	702 (16)
HMGB1	transcription regulator	3.63	1.08E-04	398 (16)
Map3k7	kinase	3.629	3.13E-05	424 (16)
IFN Beta	group	3.625	1.17E-04	508 (13)
MAP2K3	kinase	3.624	4.60E-04	585 (16)
BMP2	growth factor	3.618	3.94E-05	772 (19)
reactive oxygen species	chemical toxicant	3.617	3.29E-03	535 (14)
F7	peptidase	3.61	9.04E-08	420 (17)
IL7	cytokine	3.605	1.37E-04	579 (14)
bee venom	chemical - endogenous non-mammalian	3.593	3.40E-16	672 (16)
TRAF6	enzyme	3.588	1.02E-03	344 (14)
Pkc(s)	group	3.554	8.65E-10	632 (18)
MAPK8	kinase	3.554	4.18E-06	777 (17)
IL17a dimer	complex	3.548	1.19E-06	426 (7)
Gm-csf	group	3.536	2.25E-07	597 (18)
CD40	transmembrane receptor	3.53	3.22E-07	549 (14)
anisomycin	chemical - endogenous non-mammalian	3.511	2.30E-06	441 (15)
MALP-2s	chemical reagent	3.493	7.34E-06	372 (14)
Mapk	group	3.492	6.05E-05	609 (19)

mitomycin C	chemical drug	3.472	4.06E-06	575 (16)
tunicamycin	chemical - endogenous non-mammalian	3.462	1.38E-03	546 (14)
NfkB-RelA	complex	3.45	9.45E-06	
AGT	growth factor	3.446	3.48E-07	556 (13)
L-glutamic acid	chemical - endogenous mammalian	3.446	7.51E-03	699 (23)
bucladesine	chemical toxicant	3.433	1.14E-08	681 (19)
Tnf (family)	group	3.431	1.33E-05	423 (15)
NOD2	other	3.419	1.59E-08	387 (14)
TBK1	kinase	3.396	3.04E-04	366 (14)
CpG oligonucleotide	chemical drug	3.394	1.63E-06	508 (15)
PF4	cytokine	3.394	1.66E-05	417 (17)
TLR5	transmembrane receptor	3.385	2.16E-03	431 (15)
CHUK	kinase	3.381	4.35E-13	620 (11)
Pka	complex	3.379	3.01E-06	750 (22)
IRF6	transcription regulator	3.377	3.38E-05	381 (14)
TGFA	growth factor	3.364	2.49E-04	602 (17)
H2AFB3 (includes others)	other	3.357	1.94E-03	296 (7)
TRADD	other	3.352	4.53E-07	435 (15)
PPRC1	transcription regulator	3.347	4.55E-04	
BMP6	growth factor	3.339	1.31E-02	
clozapine	chemical drug	3.336	1.92E-03	571 (13)
ELK1	transcription regulator	3.333	7.07E-06	651 (19)
IL18	cytokine	3.317	2.03E-03	568 (14)
potassium chloride	chemical drug	3.315	6.88E-09	646 (20)
NFKBIA	transcription regulator	3.308	6.76E-11	732 (15)
F2R	g-protein coupled receptor	3.3	1.08E-04	579 (15)
AKT1	kinase	3.295	3.88E-06	631 (17)
Ni2+	chemical reagent	3.295	1.03E-04	728 (14)
di(2-ethylhexyl) phthalate	chemical toxicant	3.286	1.16E-01	

CD14	transmembrane receptor	3.285	9.25E-06	404 (16)
3M-001	chemical drug	3.274	6.86E-03	276 (7)
paclitaxel	chemical drug	3.27	3.50E-12	806 (16)
CXCR4	g-protein coupled receptor	3.266	2.31E-07	504 (16)
EIF2AK2	kinase	3.266	3.65E-07	532 (13)
NFKB1	transcription regulator	3.263	5.13E-08	732 (13)
AIMP1	cytokine	3.26	1.79E-07	689 (23)
RIPK2	kinase	3.259	7.38E-02	
Pam3-Cys	chemical toxicant	3.252	2.14E-04	362 (12)
ALB	transporter	3.244	2.58E-02	
IKBKB	kinase	3.242	1.34E-08	627 (12)
uric acid	chemical - endogenous mammalian	3.239	9.08E-05	650 (14)
okadaic acid	chemical toxicant	3.222	1.00E-07	578 (18)
RNASE2	enzyme	3.222	5.46E-03	531 (17)
hemozoin	chemical - endogenous non-mammalian	3.221	2.61E-09	457 (14)
SYK	kinase	3.221	1.54E-04	511 (19)
TGM2	enzyme	3.221	3.11E-04	606 (21)
ozone	chemical toxicant	3.217	2.48E-04	337 (12)
bromodeoxyuridine	chemical drug	3.207	7.78E-03	736 (17)
IL3	cytokine	3.206	5.21E-17	479 (16)
MTOR	kinase	3.204	1.80E-04	687 (20)
PGF	growth factor	3.201	1.79E-07	447 (16)
tretinoin	chemical - endogenous mammalian	3.186	3.83E-12	824 (18)
carbonyl cyanide m-chlorophenyl hydrazone	chemical toxicant	3.184	2.53E-04	472 (16)
sphingosine-1-phosphate	chemical - endogenous mammalian	3.184	1.12E-03	560 (18)
SELPLG	other	3.153	1.63E-07	515 (16)
leukotriene C4	chemical - endogenous mammalian	3.152	2.63E-06	668 (17)
haloperidol	chemical drug	3.151	2.84E-02	
MET	kinase	3.15	3.92E-05	614 (17)

IL-17f dimer	complex	3.138	1.32E-05	
SRC	kinase	3.136	1.20E-03	800 (18)
cis-urocanic acid	chemical drug	3.127	2.34E-04	323 (8)
ICAM1	transmembrane receptor	3.124	1.81E-03	473 (16)
TMEM173	other	3.124	5.46E-03	577 (16)
carbamylcholine	chemical drug	3.095	2.33E-02	
AGER	transmembrane receptor	3.092	2.61E-05	565 (17)
CAMK4	kinase	3.084	1.44E-03	597 (19)
CLEC7A	transmembrane receptor	3.07	5.51E-04	444 (18)
CpG ODN 1826	chemical reagent	3.069	1.14E-05	576 (16)
formaldehyde	chemical - endogenous mammalian	3.069	1.12E-04	522 (20)
N-acetylsphingosine	chemical reagent	3.061	1.18E-03	518 (18)
ASCL1	transcription regulator	3.06	2.85E-02	
stallimycin	biologic drug	3.059	5.52E-03	585 (13)
MAPK7	kinase	3.058	3.47E-05	454 (14)
methylnitronitrosoguanidine	chemical toxicant	3.057	3.01E-03	669 (20)
ESRRA	ligand-dependent nuclear receptor	3.053	4.23E-02	
EIF2AK3	kinase	3.045	4.09E-03	727 (21)
methylprednisolone	chemical drug	3.033	2.25E-06	725 (16)
NOTCH1	transcription regulator	3.023	1.29E-06	536 (21)
dinoprost	chemical - endogenous mammalian	3.018	2.49E-04	585 (19)
STAT1	transcription regulator	3.01	1.85E-08	643 (13)
ARHGAP21	other	3	1.08E-01	
ST3-Hel2A-2	chemical reagent	2.999	5.36E-07	352 (9)
amphetamine	chemical drug	2.996	1.56E-03	655 (22)
MIF	cytokine	2.992	3.74E-04	532 (16)
4-hydroxytamoxifen	chemical drug	2.987	1.12E-05	671 (19)
C3	peptidase	2.986	8.70E-03	476 (19)
LCN2	transporter	2.971	6.88E-04	463 (19)
5-hydroxytryptamine	chemical - endogenous mammalian	2.969	4.41E-06	528 (19)

EGR1	transcription regulator	2.957	1.26E-09	882 (18)
phorbol esters	chemical - other	2.957	7.41E-05	499 (17)
VEGFA	growth factor	2.949	2.18E-07	477 (16)
HSPD1	enzyme	2.949	1.66E-05	428 (16)
ATF4	transcription regulator	2.945	1.60E-04	639 (20)
IL12 (family)	group	2.944	1.83E-02	
RET	kinase	2.942	1.85E-06	676 (17)
SB 216763	chemical toxicant	2.935	2.69E-11	558 (17)
vitamin K3	chemical drug	2.934	6.20E-03	716 (20)
IL17C	cytokine	2.929	3.01E-03	648 (16)
NfkB1-RelA	complex	2.929	4.68E-03	351 (4)
JAK2	kinase	2.927	1.24E-02	
PRKCD	kinase	2.925	1.40E-07	498 (14)
D-galactosamine	chemical - endogenous mammalian	2.922	3.38E-05	476 (14)
S-(2,3-bispalmitoyloxypropyl)-cysteine-GDPKHPKSF	chemical reagent	2.921	1.97E-04	444 (16)
cytarabine	chemical drug	2.919	1.10E-02	
IL21	cytokine	2.916	1.83E-03	688 (16)
lysophosphatidic acid	chemical - other	2.913	1.56E-11	444 (16)
LCK	kinase	2.912	1.31E-02	
TLR8	transmembrane receptor	2.912	1.21E-01	
MAPK3	kinase	2.902	2.43E-06	504 (18)
25-hydroxycholesterol	chemical reagent	2.892	4.72E-07	651 (21)
MAVS	other	2.888	2.53E-02	
trichostatin A	chemical drug	2.886	3.33E-10	830 (19)
HNF1B	transcription regulator	2.884	1.20E-03	378 (8)
10E,12Z-octadecadienoic acid	chemical - endogenous mammalian	2.881	3.39E-08	480 (19)
PI3K (complex)	complex	2.879	1.28E-08	742 (18)
GCG	other	2.878	4.44E-03	719 (21)
S100A9	other	2.867	4.48E-04	555 (20)

RHO	g-protein coupled receptor	2.84	6.21E-03	
3-deazaneplanocin	chemical drug	2.839	2.53E-02	
STAT3	transcription regulator	2.831	3.32E-07	662 (15)
E. coli serotype 0127B8 lipopolysaccharide	chemical - endogenous non-mammalian	2.831	1.11E-03	666 (15)
aldosterone	chemical - endogenous mammalian	2.83	9.32E-08	658 (21)
N-acetylmuramyl-L-alanyl-D-isoglutamine	chemical - endogenous non-mammalian	2.829	3.56E-05	448 (15)
NEDD9	other	2.828	1.28E-07	545 (17)
lysophosphatidylcholine	chemical - other	2.828	1.02E-04	532 (16)
tosedostat	chemical drug	2.828	3.75E-02	
IL17F	cytokine	2.82	5.35E-04	596 (15)
ATF2	transcription regulator	2.817	1.36E-04	579 (15)
DPP-23	chemical reagent	2.813	1.84E-03	
S100A8	other	2.812	9.88E-05	439 (12)
lipoteichoic acid	chemical - endogenous non-mammalian	2.81	9.34E-07	450 (14)
GC-GCR dimer	complex	2.804	4.52E-04	
TSH	complex	2.798	1.94E-03	806 (21)
Pdgf Ab	complex	2.797	1.20E-03	520 (18)
CHRM1	g-protein coupled receptor	2.791	2.85E-05	615 (19)
SRC (family)	group	2.791	2.58E-02	
SN-38	chemical drug	2.791	3.52E-02	
3M-011	chemical reagent	2.791	6.24E-02	
MAP3K14	kinase	2.788	4.71E-03	518 (12)
BMP15	growth factor	2.786	5.38E-03	473 (12)
C5AR1	g-protein coupled receptor	2.785	9.11E-04	571 (13)
PTH	other	2.784	3.42E-08	555 (18)
SELP	transmembrane receptor	2.782	2.86E-07	627 (15)
EDN1	cytokine	2.778	1.93E-04	548 (14)

IL24	cytokine	2.778	2.78E-03	589 (14)
APP	other	2.774	1.21E-07	825 (18)
CP-55940	chemical reagent	2.772	2.14E-04	698 (20)
decitabine	chemical drug	2.759	3.69E-08	848 (22)
silicon dioxide	chemical drug	2.756	4.52E-04	707 (19)
IL15	cytokine	2.753	9.75E-12	626 (14)
zymosan	chemical - endogenous non-mammalian	2.753	2.31E-07	407 (13)
OSM	cytokine	2.751	3.94E-06	624 (15)
EPO	cytokine	2.749	5.83E-07	671 (16)
BCR (complex)	complex	2.746	1.58E-03	549 (18)
EPAS1	transcription regulator	2.734	4.42E-07	800 (19)
MAP3K8	kinase	2.723	1.40E-04	568 (12)
TGFB1	growth factor	2.719	4.35E-24	932 (17)
CRH	cytokine	2.719	1.92E-03	546 (17)
KRT17	other	2.714	1.06E-03	
Fibrinogen	complex	2.71	2.66E-06	483 (16)
norepinephrine	chemical - endogenous mammalian	2.708	4.51E-08	631 (20)
GH1	growth factor	2.699	1.04E-03	740 (23)
C5	cytokine	2.688	6.07E-11	721 (17)
carboplatin	chemical drug	2.688	4.45E-03	298 (7)
vinblastine	chemical drug	2.682	2.04E-02	
DDX58	enzyme	2.678	1.09E-02	
LIF	cytokine	2.667	1.47E-05	578 (19)
TNFSF13B	cytokine	2.658	1.47E-04	555 (16)
phorbol 12,13-dibutyrate	chemical - endogenous non-mammalian	2.648	1.06E-02	
S-nitroso-N-acetylpenicillamine	chemical reagent	2.641	6.66E-03	677 (17)
SAA	group	2.637	2.33E-04	574 (16)
galactosylceramide-alpha	chemical reagent	2.632	3.83E-02	

STIM1	ion channel	2.63	1.05E-03	765 (22)
EPHB1	kinase	2.621	5.96E-05	448 (18)
CXCL1	cytokine	2.621	3.08E-04	722 (20)
STAT	group	2.621	1.66E-03	388 (11)
PADI2	enzyme	2.621	1.74E-03	281 (7)
RNASE1	enzyme	2.621	1.53E-02	
Lymphotoxin	complex	2.619	1.84E-03	503 (12)
FN1	enzyme	2.618	1.61E-06	714 (18)
isoproterenol	chemical drug	2.614	3.98E-03	532 (15)
CXCL3	cytokine	2.607	2.74E-03	613 (16)
SMARCA4	transcription regulator	2.605	1.83E-12	790 (19)
IPMK	kinase	2.596	1.99E-02	
PRKD1	kinase	2.596	6.83E-02	
CCL4	cytokine	2.595	5.99E-03	520 (17)
AHR	ligand-dependent nuclear receptor	2.594	7.03E-04	783 (19)
Akt	group	2.593	4.94E-10	688 (17)
ssRNA40	chemical reagent	2.586	1.20E-03	574 (18)
IL31	other	2.586	8.41E-03	589 (17)
PDX1	transcription regulator	2.585	3.16E-05	780 (18)
TLR6	transmembrane receptor	2.583	1.99E-02	
MAP3K3	kinase	2.581	2.50E-03	519 (17)
CEBPB	transcription regulator	2.577	3.65E-08	754 (18)
monophosphoryl lipid A	chemical reagent	2.576	1.74E-03	497 (16)
IRAK2	kinase	2.572	4.52E-04	370 (17)
homocysteine	chemical - endogenous mammalian	2.571	8.90E-05	535 (18)
THPO	cytokine	2.565	4.11E-01	
lauric acid	chemical - endogenous mammalian	2.563	1.74E-03	471 (17)
EP300	transcription regulator	2.556	7.17E-06	865 (19)
Endothelin	group	2.556	4.13E-03	657 (17)
TRAF3IP2	other	2.552	7.48E-02	

PTPRC	phosphatase	2.55	6.20E-03	452 (19)
8-bromo-cAMP	chemical reagent	2.549	1.37E-02	
FOXO1	transcription regulator	2.547	8.64E-05	634 (18)
IFI16	transcription regulator	2.547	5.59E-04	547 (13)
IL32	cytokine	2.546	4.00E-05	430 (16)
CD36	transmembrane receptor	2.546	7.59E-02	
Hmgb1	transcription regulator	2.543	4.13E-03	554 (20)
PRKCA	kinase	2.534	2.22E-05	417 (15)
Nfat (family)	group	2.532	2.08E-05	664 (18)
LHX1	transcription regulator	2.53	2.12E-01	
N-formyl-Met-Leu-Phe	chemical reagent	2.518	6.86E-03	354 (13)
CXCL12	cytokine	2.509	2.58E-08	680 (14)
C5	other	2.507	9.39E-08	672 (17)
ATP	chemical - endogenous mammalian	2.507	9.50E-06	447 (15)
Pdgf (complex)	complex	2.5	9.15E-06	437 (16)
cobalt chloride	chemical reagent	2.496	8.28E-05	668 (18)
TRAF2	enzyme	2.494	2.38E-05	387 (17)
RHOA	enzyme	2.486	1.08E-02	
NRAS	enzyme	2.468	3.59E-04	493 (17)
MAP2K4	kinase	2.467	2.91E-02	
AREG	growth factor	2.466	1.68E-04	757 (18)
KLF6	transcription regulator	2.465	9.34E-07	515 (16)
BDNF	growth factor	2.455	7.16E-06	666 (21)
blinatumomab	biologic drug	2.449	5.42E-06	
IL26	cytokine	2.449	1.19E-04	522 (14)
CNB-001	chemical reagent	2.449	1.38E-03	513 (7)
KLK5	peptidase	2.449	2.43E-02	
CARM1	transcription regulator	2.449	4.16E-02	
GNRH	group	2.447	3.19E-02	
hyaluronic acid	chemical - endogenous mammalian	2.443	1.20E-05	651 (13)

EREG	growth factor	2.442	1.11E-04	855 (24)
quinolinic acid	chemical - endogenous mammalian	2.441	1.18E-03	690 (21)
NR5A2	ligand-dependent nuclear receptor	2.439	6.90E-03	777 (21)
IGF1R	transmembrane receptor	2.438	4.14E-03	837 (21)
Ccl2	cytokine	2.436	1.66E-04	584 (15)
STAT5B	transcription regulator	2.432	4.12E-04	552 (15)
TLR1	transmembrane receptor	2.432	8.41E-03	329 (14)
lipoarabinomannan	chemical - endogenous non-mammalian	2.429	9.45E-06	573 (17)
CSF2RA	transmembrane receptor	2.429	4.03E-03	562 (19)
ELANE	peptidase	2.428	3.53E-04	641 (20)
pCPT-cAMP	chemical - kinase inhibitor	2.428	7.04E-04	747 (18)
FGFR1	kinase	2.426	2.14E-03	471 (17)
IL22	cytokine	2.426	1.31E-02	
Interferon alpha	group	2.425	2.93E-03	525 (14)
LILRA2	other	2.425	4.03E-03	309 (7)
GIP	other	2.425	1.32E-02	
ERVW-1	other	2.425	4.02E-02	
BMPR1A	kinase	2.425	2.33E-01	
Gm21596/Hmgb1	transcription regulator	2.424	9.29E-03	389 (17)
FHIT	enzyme	2.423	1.99E-01	
Lh	complex	2.416	3.65E-07	825 (20)
cyclopiazonic acid	chemical - endogenous non-mammalian	2.416	1.32E-02	
peroxynitrite	chemical toxicant	2.415	1.82E-02	
asbestos	chemical toxicant	2.412	3.17E-04	
IL6ST	transmembrane receptor	2.412	3.79E-02	
GRP	growth factor	2.41	1.04E-01	
MAPK10	kinase	2.409	1.38E-03	549 (16)
BCL10	transcription regulator	2.409	1.74E-03	424 (15)

NOD1	other	2.404	5.91E-04	433 (17)
ADCYAP1R1	g-protein coupled receptor	2.4	3.36E-05	668 (21)
SYK/ZAP	group	2.4	4.03E-03	375 (17)
STAT5A	transcription regulator	2.398	2.51E-05	622 (18)
IL6R	transmembrane receptor	2.397	1.04E-04	605 (17)
4-hydroxynonenal	chemical toxicant	2.396	2.01E-02	
USP7	peptidase	2.395	3.94E-02	
poly dA-dT	chemical reagent	2.392	1.32E-02	
CD244	transmembrane receptor	2.39	1.19E-04	562 (19)
TNFSF15	cytokine	2.382	1.58E-01	
FGF10	growth factor	2.377	8.51E-02	
Am 580	chemical toxicant	2.376	6.66E-03	439 (10)
NAMPT	cytokine	2.373	1.77E-03	611 (18)
NR3C2	ligand-dependent nuclear receptor	2.372	2.69E-07	759 (18)
N-methyl-D-aspartate	chemical reagent	2.362	5.52E-03	738 (22)
MTORC1	complex	2.359	4.45E-02	
Pro-inflammatory Cytokine	group	2.359	5.43E-02	
YAP1	transcription regulator	2.358	9.91E-05	792 (23)
MKNK1	kinase	2.353	5.56E-04	
mitoxantrone	chemical drug	2.353	1.60E-02	
isopentenyl diphosphate	chemical - endogenous mammalian	2.352	4.03E-03	537 (18)
Notch	group	2.342	7.82E-07	620 (19)
FOXC2	transcription regulator	2.335	8.13E-03	527 (17)
MAPK13	kinase	2.333	2.14E-05	640 (19)
UHRF2	enzyme	2.333	2.50E-03	
IRAK4	kinase	2.32	1.82E-14	653 (14)
sirolimus	chemical drug	2.312	6.85E-23	761 (18)
CD40LG	cytokine	2.303	3.98E-13	627 (15)
4-methylnitrosoamino-1-(3-pyridinyl)-1-butanone	chemical toxicant	2.303	1.63E-02	

IL33	cytokine	2.3	1.39E-03	680 (15)
EZH2	transcription regulator	2.296	2.85E-06	876 (26)
RETN	other	2.295	1.09E-05	677 (19)
Mek	group	2.294	3.65E-08	556 (18)
colchicine	chemical drug	2.292	2.40E-03	593 (19)
8-chlorophenylthio-adenosine 3',5'-cyclic monophosphate	chemical - kinase inhibitor	2.291	2.97E-04	787 (24)
IRF5	transcription regulator	2.291	1.25E-03	534 (14)
CREBBP	transcription regulator	2.288	1.60E-06	706 (19)
TBX5	transcription regulator	2.286	2.40E-04	494 (8)
MAPK1	kinase	2.284	7.19E-07	646 (16)
MEF2C	transcription regulator	2.281	1.11E-04	328 (13)
KNG1	other	2.278	5.19E-03	676 (18)
ceruletide	biologic drug	2.267	5.38E-03	563 (14)
cyclophosphamide	chemical drug	2.266	3.13E-02	
IL1R1	transmembrane receptor	2.258	2.14E-04	409 (15)
MAP3K1	kinase	2.257	3.58E-03	580 (17)
HRAS	enzyme	2.249	1.29E-09	790 (19)
JAK1	kinase	2.238	1.28E-02	
Smad2/3-Smad4	complex	2.236	7.55E-04	202 (5)
ponesimod	chemical drug	2.236	1.62E-03	
TCF12	transcription regulator	2.236	4.33E-03	324 (7)
C1q	complex	2.236	5.77E-03	696 (20)
manganese	chemical - endogenous mammalian	2.236	6.27E-03	644 (21)
ZAP70	kinase	2.236	1.28E-02	
1,2-dimethylhydrazine	chemical toxicant	2.236	1.44E-02	
D-sphingosine	chemical - endogenous mammalian	2.236	1.44E-02	
NFkB (family)	group	2.236	2.91E-02	
MYT1	transcription regulator	2.236	6.42E-02	
FAAH	enzyme	2.236	1.58E-01	

dithiothreitol	chemical reagent	2.236	2.84E-01	
OGG1	enzyme	2.233	2.18E-04	562 (14)
domoic acid	chemical toxicant	2.232	3.24E-03	535 (16)
phorbol 12,13-didecanoate	chemical toxicant	2.228	5.35E-04	822 (22)
AVP	other	2.226	5.13E-02	
ICOSLG/LOC102723996	other	2.225	8.84E-02	
quisqualic acid	chemical - endogenous non-mammalian	2.224	4.09E-05	739 (20)
8-hydroxyguanine	chemical - endogenous mammalian	2.219	2.18E-04	
ANKRD42	transcription regulator	2.219	6.80E-04	301 (7)
15-E2-isoketal modified phosphatidylethanolamine	chemical reagent	2.219	6.80E-04	315 (7)
Ck2	complex	2.219	3.24E-03	723 (20)
FBXO42	other	2.219	3.24E-03	287 (7)
Cd2+	chemical toxicant	2.219	6.27E-03	755 (22)
STAT1/3/5 dimer	complex	2.219	1.44E-02	
NMDA Receptor	complex	2.219	6.42E-02	
Angiotensin II receptor type 1	group	2.219	6.42E-02	
SPHK2	kinase	2.217	6.27E-03	599 (16)
CXCL2	cytokine	2.216	5.04E-07	455 (14)
ULBP1	transmembrane receptor	2.213	1.62E-03	342 (12)
11-dehydrocorticosterone	chemical - endogenous mammalian	2.213	3.24E-03	
FAT1	other	2.213	3.24E-03	338 (7)
(E)-4-hydroxy-3-methyl-but-2-enyl pyrophosphate	chemical - endogenous non-mammalian	2.213	9.43E-03	
p85 (pik3r)	group	2.213	1.82E-02	
FGF19	growth factor	2.213	2.55E-02	
idarubicin	chemical drug	2.213	3.16E-02	
PCDH11Y	other	2.213	1.36E-01	
FOXO4	transcription regulator	2.211	2.91E-03	517 (15)

3M-002	chemical reagent	2.211	1.82E-02	
picryl chloride	chemical toxicant	2.211	2.91E-02	
zymosan A	chemical - endogenous non-mammalian	2.211	2.91E-02	
TNFSF4	cytokine	2.209	5.07E-02	
titanium dioxide	chemical drug	2.208	2.53E-02	
DRD1	g-protein coupled receptor	2.207	1.38E-03	752 (21)
tetrachlorodibenzodioxin	chemical toxicant	2.207	5.53E-03	804 (24)
DDX5	enzyme	2.206	1.74E-03	636 (17)
NANOG	transcription regulator	2.204	6.75E-04	409 (11)
dichlorovinylcysteine	chemical toxicant	2.204	8.02E-02	
TGFB3	growth factor	2.203	3.29E-03	717 (20)
2,4-dinitrofluorobenzene	chemical toxicant	2.203	1.82E-02	
ITGB1BP1	transporter	2.201	6.80E-04	546 (12)
irinotecan	chemical drug	2.201	1.44E-02	
palmitoyl-Cys((RS)-2,3-di(palmitoyloxy)-propyl)-Ala-Gly-OH	chemical reagent	2.2	1.62E-03	380 (14)
NCF1	enzyme	2.2	6.27E-03	718 (21)
exenatide	biologic drug	2.2	3.75E-02	
TEK	kinase	2.2	5.07E-02	
IFNL3	other	2.2	1.15E-01	
HOXA9	transcription regulator	2.199	6.60E-10	
acetic acid	chemical - endogenous mammalian	2.196	1.62E-03	361 (11)
LUM	other	2.195	5.77E-03	276 (7)
anti-benzo(a)pyrene-diol-epoxide	chemical toxicant	2.195	9.43E-03	735 (18)
IL2RB	transmembrane receptor	2.194	2.91E-02	
NFATC1	transcription regulator	2.192	2.16E-01	
LAT	kinase	2.191	2.74E-03	426 (20)
reserpine	chemical drug	2.191	5.07E-02	
chenodeoxycholic acid	chemical - endogenous mammalian	2.19	1.21E-01	

Klrk1	other	2.189	6.80E-04	700 (18)
RIPK3	kinase	2.189	3.24E-03	401 (15)
CXCL10	cytokine	2.189	1.26E-02	
PLA2G10	enzyme	2.189	2.09E-02	
ATM	kinase	2.187	1.40E-06	598 (13)
LITAF	transcription regulator	2.186	6.80E-04	470 (16)
MAP2K7	kinase	2.186	2.53E-02	
NOS1	enzyme	2.186	9.67E-02	
HOXA5	transcription regulator	2.184	1.16E-05	
ITGB1	transmembrane receptor	2.184	1.33E-03	419 (15)
CD86	transmembrane receptor	2.184	2.38E-03	569 (15)
aplidine	biologic drug	2.183	9.50E-08	574 (19)
risperidone	chemical drug	2.182	6.16E-02	
PSEN1	peptidase	2.18	3.14E-04	722 (18)
temozolomide	chemical drug	2.178	2.95E-02	
SELL	transmembrane receptor	2.176	6.80E-04	441 (15)
IL18R1	transmembrane receptor	2.173	1.62E-03	359 (13)
prostaglandin A2	chemical - endogenous non-mammalian	2.17	2.91E-02	
miR-16-5p (and other miRNAs w/seed AGCAGCA)	mature microrna	2.169	9.93E-07	
IRF8	transcription regulator	2.169	6.66E-06	753 (19)
platelet activating factor	chemical - endogenous mammalian	2.168	7.27E-05	393 (14)
XIAP	enzyme	2.165	1.27E-07	578 (16)
TRPM2	ion channel	2.162	9.67E-02	
IFNA2	cytokine	2.161	1.59E-04	530 (13)
EGR3	transcription regulator	2.157	1.08E-01	
naltrexone	chemical drug	2.156	9.67E-02	
PLC	group	2.155	2.63E-06	545 (19)
clenbuterol	chemical drug	2.151	6.55E-05	505 (16)

Ins1	other	2.151	1.31E-03	645 (23)
FGF7	growth factor	2.151	7.58E-03	513 (17)
apomorphine	chemical drug	2.151	3.11E-01	
NCR1	transmembrane receptor	2.15	5.77E-03	511 (18)
HSP90B1	other	2.147	2.57E-01	
HMGA1	transcription regulator	2.142	2.68E-04	817 (21)
nickel chloride	chemical toxicant	2.141	3.24E-03	562 (17)
TSLP	cytokine	2.138	2.95E-02	
PIK3CG	kinase	2.137	2.19E-02	
IFNL1	cytokine	2.136	1.76E-01	
SREBF1	transcription regulator	2.135	6.75E-02	
PTGER2	g-protein coupled receptor	2.125	1.74E-03	614 (17)
NTRK1	kinase	2.121	1.22E-03	674 (22)
CYP1A1	enzyme	2.121	1.08E-02	
LHX2	transcription regulator	2.121	1.62E-02	
CYP1A2	enzyme	2.121	2.60E-01	
CYP1B1	enzyme	2.121	4.48E-01	
IKBKG	kinase	2.118	2.27E-07	627 (12)
IFNB1	cytokine	2.118	1.81E-04	590 (14)
CD5	transmembrane receptor	2.115	7.78E-03	557 (20)
Fgf	group	2.111	1.94E-03	547 (19)
MAPK12	kinase	2.109	4.88E-05	462 (17)
trinitrobenzenesulfonic acid	chemical reagent	2.104	7.41E-05	614 (19)
TACR1	g-protein coupled receptor	2.1	1.11E-04	489 (18)
ELAVL1	other	2.092	7.03E-07	369 (11)
SAA1	transporter	2.091	1.26E-02	
GW501516	chemical drug	2.081	1.68E-01	
cephaloridine	chemical drug	2.076	1.00E+00	
ERN1	kinase	2.074	8.14E-03	463 (18)
AMH	growth factor	2.074	1.62E-02	

CD38	enzyme	2.069	4.11E-03	644 (20)
SMAD4	transcription regulator	2.068	3.29E-06	548 (15)
3-methylcholanthrene	chemical toxicant	2.067	2.12E-01	
SMAD3	transcription regulator	2.059	4.99E-08	745 (16)
IL27	cytokine	2.055	9.90E-09	587 (14)
IL23A	cytokine	2.045	1.39E-01	
TNFRSF8	transmembrane receptor	2.043	5.43E-02	
dibutyl phthalate	chemical toxicant	2.039	3.94E-02	
TGIF1	transcription regulator	2.033	2.74E-03	691 (19)
1-methyl-4-phenyl-1,2,3,6-tetrahydropyridine	chemical toxicant	2.029	3.72E-04	457 (16)
RIPK1	kinase	2.027	1.99E-02	
R5020	chemical reagent	2.022	7.50E-02	
ITGA1	other	2.015	3.19E-02	
tetracycline	chemical drug	2.01	2.02E-01	
IL-1R	group	2.007	9.71E-03	358 (15)
PARP1	enzyme	2.006	1.73E-05	554 (13)
Hsp27	group	2.006	8.52E-03	658 (23)
IRF7	transcription regulator	2.002	8.18E-03	480 (17)
TAZ	enzyme	2	9.99E-07	
6,7-dinitroquinoxaline-2,3-dione	chemical reagent	2	2.18E-04	578 (17)
sGC	complex	2	3.09E-04	800 (24)
CAY10397	chemical reagent	2	3.09E-04	
hemin	chemical - endogenous mammalian	2	6.70E-04	641 (24)
MNK1/2	group	2	1.38E-03	
TLR10	transmembrane receptor	2	1.38E-03	511 (18)
Tlr12	other	2	1.38E-03	515 (19)
KIDINS220	transcription regulator	2	1.38E-03	341 (9)
COCH	other	2	3.24E-03	
psychosine	chemical - endogenous mammalian	2	3.71E-03	561 (20)
CLEC6A	transmembrane receptor	2	3.71E-03	450 (16)

F13A1	enzyme	2	3.71E-03	
Pdgfr	group	2	5.77E-03	491 (17)
IL1RL2	transmembrane receptor	2	7.74E-03	327 (9)
TSC22D1	transcription regulator	2	7.74E-03	528 (14)
fingolimod phosphate	chemical - endogenous mammalian	2	1.39E-02	
GPR84	g-protein coupled receptor	2	1.39E-02	
U1 snRNP	complex	2	1.39E-02	
CTSZ	peptidase	2	2.24E-02	
NEK6	kinase	2	2.24E-02	
ALOX12	enzyme	2	4.71E-02	
CHRM3	g-protein coupled receptor	2	4.71E-02	
clioquinol	chemical drug	2	4.71E-02	
BRD2	kinase	2	6.78E-02	
IL1RAP	transmembrane receptor	2	8.24E-02	
PTPRE	phosphatase	2	1.04E-01	
Fgfr	group	2	1.27E-01	
PPARÎ±-RXRÎ±	complex	2	1.27E-01	
OGT	enzyme	2	1.93E-01	
finasteride	chemical drug	2	2.69E-01	
ATP7B	transporter	2	1.00E+00	
**Inhibited**				
Ho	group	-2	1.38E-03	
BPI	transporter	-2	1.38E-03	581 (10)
STX11	transporter	-2	1.38E-03	591 (15)
tranexamic acid	chemical drug	-2	1.38E-03	421 (12)
HLA-DQ	complex	-2	3.71E-03	424 (7)
SAR-20347	chemical - kinase inhibitor	-2	3.71E-03	
belnacasan	chemical drug	-2	7.74E-03	342 (7)
PPP5C	phosphatase	-2	7.74E-03	686 (19)

pimozide	chemical drug	-2	2.24E-02	
calmidazolium	chemical drug	-2	3.34E-02	
chlorogenic acid	chemical drug	-2	9.67E-02	
1'-acetoxychavicol acetate	chemical reagent	-2	1.27E-01	
STK3	kinase	-2	1.27E-01	
p70 S6k	group	-2	1.58E-01	
artesunic acid	chemical drug	-2	2.09E-01	
L-carnitine	chemical - endogenous mammalian	-2	2.09E-01	
creatine	chemical - endogenous mammalian	-2	2.39E-01	
miR-293-5p (and other miRNAs w/seed CUCAAAC)	mature microrna	-2	3.01E-01	
vasoactive intestinal peptide	biologic drug	-2	3.64E-01	
HOXC8	transcription regulator	-2	1.00E+00	
SNAI1	transcription regulator	-2.016	3.80E-06	781 (25)
iron	chemical - endogenous mammalian	-2.028	2.94E-03	455 (17)
mir-8	microrna	-2.042	7.76E-05	
telmisartan	chemical drug	-2.049	4.33E-03	776 (20)
Igm	complex	-2.065	5.52E-04	407 (18)
cilostazol	chemical drug	-2.067	5.46E-03	550 (19)
cyclosporin A	biologic drug	-2.083	1.64E-06	597 (17)
DICER1	enzyme	-2.095	1.44E-04	858 (23)
Ro31-8220	chemical - kinase inhibitor	-2.104	3.39E-06	569 (19)
inosine	chemical - endogenous mammalian	-2.117	1.94E-04	
LAT2	other	-2.117	3.24E-03	403 (17)
RB1	transcription regulator	-2.119	3.55E-03	726 (16)
DUSP5	phosphatase	-2.121	2.71E-03	300 (7)
PAX1	transcription regulator	-2.121	2.60E-01	
RAB1B	other	-2.132	6.24E-02	
ZNF217	transcription regulator	-2.137	1.17E-02	
TNFRSF4	transmembrane receptor	-2.138	7.43E-02	

aspirin	chemical drug	-2.144	7.27E-06	422 (14)
BIRC5	other	-2.148	2.53E-02	
CD3	complex	-2.158	1.14E-12	685 (16)
PROC	peptidase	-2.162	2.32E-06	455 (15)
IL18BP	other	-2.162	1.62E-03	507 (12)
eritoran	chemical drug	-2.163	4.09E-05	485 (10)
miR-125b-5p (and other miRNAs w/seed CCCUGAG)	mature microrna	-2.165	1.35E-01	
L-histidine	chemical - endogenous mammalian	-2.166	1.44E-02	
sulfasalazine	chemical drug	-2.169	2.91E-02	
TSC22D3	transcription regulator	-2.17	7.23E-05	588 (16)
FOXG1	transcription regulator	-2.17	2.09E-02	
mir-214	microrna	-2.174	1.74E-03	258 (6)
Irgm1	other	-2.18	2.03E-01	
SIRPA	phosphatase	-2.182	2.44E-03	542 (14)
DTX1	transcription regulator	-2.184	4.18E-06	370 (12)
AURKB	kinase	-2.186	9.67E-02	
vinpocetine	chemical drug	-2.19	6.80E-04	305 (8)
TSC2	other	-2.195	1.38E-03	577 (19)
clarithromycin	chemical drug	-2.195	6.27E-03	536 (18)
hexamethoxyflavone	chemical toxicant	-2.197	3.24E-03	680 (17)
glaucocalyxin A	chemical - endogenous non-mammalian	-2.2	2.18E-04	495 (9)
polymyxin B	biologic drug	-2.2	1.62E-03	540 (13)
ATG16L1	enzyme	-2.2	3.24E-03	569 (15)
ENTPD1	enzyme	-2.2	3.24E-03	373 (15)
ethyl pyruvate	chemical drug	-2.2	7.95E-02	
TNFAIP8L2	other	-2.201	2.91E-02	
NR0B2	ligand-dependent nuclear receptor	-2.201	1.00E+00	
mir-373	microrna	-2.205	9.43E-03	522 (10)

2-aminopurine	chemical reagent	-2.207	7.31E-03	623 (20)
nicotinic acid	chemical - endogenous mammalian	-2.211	3.39E-01	
grepafloxacin	chemical drug	-2.213	4.09E-05	315 (7)
TPCA-1	chemical - kinase inhibitor	-2.213	2.18E-04	395 (12)
diazepam	chemical drug	-2.213	6.80E-04	488 (14)
AMBP	transporter	-2.213	1.62E-03	625 (17)
CD200	other	-2.213	2.43E-02	
iguratimod	chemical drug	-2.216	6.80E-04	543 (14)
6-cyano-7-nitroquinoxaline-2,3-dione	chemical reagent	-2.219	5.42E-06	247 (7)
PDCD4	other	-2.219	1.44E-02	
mir-150	microrna	-2.219	1.39E-01	
ZC3H12A	enzyme	-2.221	2.31E-01	
miR-31-5p (and other miRNAs w/seed GGCAAGA)	mature microrna	-2.23	5.29E-04	
PRTN3	peptidase	-2.232	6.80E-04	494 (20)
NKX2-3	transcription regulator	-2.233	3.00E-04	
1-palmitoyl-2-oleoylglycero-3-phosphoglycerol	chemical reagent	-2.236	4.09E-05	575 (19)
prazosin	chemical drug	-2.236	5.91E-04	657 (18)
rabeprazole	chemical drug	-2.236	6.80E-04	
PPT1	enzyme	-2.236	1.62E-03	
Ighg2b	other	-2.236	3.24E-03	298 (7)
astragalin	chemical - endogenous non-mammalian	-2.236	9.43E-03	287 (7)
NELFB	other	-2.236	1.44E-02	
metoprolol	chemical drug	-2.236	1.44E-02	
SIN3A	transcription regulator	-2.236	1.50E-02	
NT5E	phosphatase	-2.236	5.07E-02	
SIRT3	enzyme	-2.236	6.42E-02	
Ubiquitin	group	-2.236	1.39E-01	
mir-181	microrna	-2.238	6.86E-03	546 (12)

IL10	cytokine	-2.242	1.53E-09	636 (15)
miR-155-5p (miRNAs w/seed UAAUGCU)	mature microrna	-2.272	4.20E-11	332 (7)
mir-146	microrna	-2.275	5.30E-04	427 (12)
ethylene glycol tetraacetic acid	chemical reagent	-2.275	8.52E-03	420 (15)
erythromycin	chemical drug	-2.28	4.52E-04	563 (17)
TNFAIP3	enzyme	-2.281	2.91E-03	357 (15)
thalidomide	chemical drug	-2.284	4.98E-04	444 (14)
Ciap	group	-2.284	5.51E-04	584 (16)
APC	enzyme	-2.298	8.50E-03	663 (17)
diphenyleneiodonium	chemical reagent	-2.306	1.66E-04	588 (21)
HIC1	transcription regulator	-2.308	1.19E-02	
RAE1	other	-2.333	3.30E-04	
S100A6	transporter	-2.333	1.04E-02	
verapamil	chemical drug	-2.333	6.53E-02	
ABCA1	transporter	-2.337	3.63E-03	644 (17)
carbon monoxide	chemical - endogenous mammalian	-2.344	1.40E-04	465 (15)
MAX	transcription regulator	-2.358	9.04E-05	107 (2)
10-nitrooleate	chemical - endogenous mammalian	-2.36	2.95E-05	595 (14)
CD3E	transmembrane receptor	-2.37	1.87E-03	505 (18)
tempol	chemical drug	-2.376	2.45E-02	
bardoxolone methyl	chemical drug	-2.379	2.46E-04	610 (17)
CLDN7	other	-2.393	1.28E-03	
trans-hydroxytamoxifen	chemical drug	-2.396	1.87E-02	
L-685,458	chemical - protease inhibitor	-2.4	9.29E-03	739 (19)
SERCA	group	-2.401	5.42E-06	542 (16)
azithromycin	chemical drug	-2.401	3.08E-04	631 (19)
REST	transcription regulator	-2.401	1.00E+00	
etanercept	biologic drug	-2.407	2.46E-04	538 (16)
MTDH	transcription regulator	-2.415	2.54E-02	
1,2-dithiol-3-thione	chemical reagent	-2.417	4.16E-01	

DNMT1	enzyme	-2.418	2.47E-02	
NTN1	other	-2.423	1.53E-02	
tocilizumab	biologic drug	-2.425	1.19E-04	367 (14)
edaravone	chemical drug	-2.425	7.04E-04	551 (15)
8-oxo-7-hydrodeoxyguanosine	chemical - endogenous mammalian	-2.433	7.04E-04	660 (17)
USP18	peptidase	-2.433	5.99E-03	542 (16)
SL 327	chemical - protease inhibitor	-2.433	1.39E-01	
andrographolide	chemical drug	-2.433	2.68E-01	
resolvin D1	chemical - endogenous mammalian	-2.438	5.90E-07	635 (17)
ubiquinone 9	chemical - endogenous non-mammalian	-2.449	3.36E-05	522 (16)
cyclomaltodextrin	chemical drug	-2.449	4.03E-03	387 (11)
PCSK1	peptidase	-2.449	4.13E-03	725 (20)
W7	chemical reagent	-2.449	5.99E-03	696 (20)
PPP2R5C	other	-2.449	1.82E-02	
PAEP	other	-2.449	2.43E-02	
Cdc42	enzyme	-2.449	2.92E-01	
FOSL1	transcription regulator	-2.466	4.14E-07	593 (16)
alpha-tocopherol	chemical drug	-2.556	1.63E-04	532 (17)
RPSA	translation regulator	-2.559	3.98E-03	485 (17)
Sn50 peptide	chemical toxicant	-2.577	4.17E-03	572 (20)
tetrodotoxin	chemical drug	-2.582	4.79E-02	
SIGIRR	transmembrane receptor	-2.586	2.74E-03	390 (15)
H-7	chemical - kinase inhibitor	-2.591	1.28E-02	
BCL6	transcription regulator	-2.62	1.64E-05	423 (13)
apigenin	chemical - endogenous non-mammalian	-2.62	8.14E-03	397 (14)
VPRBP	other	-2.621	1.53E-02	
N,N-dimethylsphingosine	chemical reagent	-2.63	3.08E-04	387 (14)
tosylphenylalanyl chloromethyl ketone	chemical - protease inhibitor	-2.638	1.45E-04	618 (15)

MNT	transcription regulator	-2.646	3.30E-04	
kaempferol	chemical toxicant	-2.646	8.09E-04	390 (14)
nimodipine	chemical drug	-2.646	1.74E-03	600 (21)
ITCH	enzyme	-2.646	2.74E-03	615 (17)
fontolizumab	biologic drug	-2.646	4.13E-03	
SOX7	transcription regulator	-2.646	1.69E-01	
pyrrolidine dithiocarbamate	chemical reagent	-2.652	1.10E-06	631 (11)
curcumin	chemical drug	-2.658	5.38E-07	561 (16)
triptolide	chemical drug	-2.671	4.41E-04	540 (12)
beta-carotene	chemical - endogenous mammalian	-2.673	1.54E-04	562 (16)
CDKN1B	kinase	-2.673	3.68E-02	
PP1	chemical - kinase inhibitor	-2.674	5.07E-05	485 (20)
MEOX2	transcription regulator	-2.68	5.90E-07	565 (11)
miR-34a-5p (and other miRNAs w/seed GGCAGUG)	mature microrna	-2.696	7.61E-02	
KN-62	chemical - kinase inhibitor	-2.72	6.36E-05	390 (15)
lipoxin A4	chemical - endogenous mammalian	-2.725	7.57E-05	713 (18)
SKIL	transcription regulator	-2.728	3.63E-03	363 (8)
NLRP12	other	-2.742	7.57E-05	645 (12)
calphostin C	chemical - kinase inhibitor	-2.743	1.04E-03	529 (15)
SR 144528	chemical reagent	-2.746	2.32E-09	528 (19)
IL37	cytokine	-2.749	1.35E-04	525 (13)
TRAIP	enzyme	-2.764	2.71E-03	377 (16)
KLF2	transcription regulator	-2.767	5.27E-06	547 (17)
anakinra	biologic drug	-2.771	1.16E-03	580 (19)
LYN	kinase	-2.776	1.80E-02	
mir-22	microrna	-2.789	2.04E-02	
H89	chemical - kinase inhibitor	-2.803	6.66E-06	614 (16)
CBX7	other	-2.828	1.84E-03	
COL18A1	other	-2.862	5.53E-03	527 (18)

infliximab	biologic drug	-2.882	5.52E-04	772 (21)
tyrphostin AG 1478	chemical - kinase inhibitor	-2.886	3.00E-04	583 (15)
amino acids	chemical - endogenous mammalian	-2.892	4.18E-06	546 (20)
SCD	enzyme	-2.912	1.04E-03	740 (17)
triflusal	chemical drug	-2.923	3.30E-04	356 (10)
PP2/AG1879 tyrosine kinase inhibitor	chemical - kinase inhibitor	-2.937	3.02E-03	419 (16)
RCAN1	transcription regulator	-2.941	1.16E-03	448 (19)
SLC13A1	transporter	-3	1.00E+00	
AG490	chemical - kinase inhibitor	-3.065	1.36E-04	605 (17)
ZFP36	transcription regulator	-3.078	2.64E-10	579 (13)
bisindolylmaleimide I	chemical - kinase inhibitor	-3.091	3.99E-04	486 (17)
SOCS1	other	-3.175	3.15E-05	584 (14)
N-acetyl-L-cysteine	chemical drug	-3.212	5.49E-08	473 (17)
Nr1h	group	-3.218	6.53E-05	695 (17)
ABCG1	transporter	-3.218	1.44E-03	759 (21)
SOCS3	phosphatase	-3.252	5.59E-04	467 (15)
PS-1145	chemical - kinase inhibitor	-3.267	5.93E-06	640 (17)
SP600125	chemical - kinase inhibitor	-3.284	9.47E-07	550 (15)
Bay 11-7082	chemical - kinase inhibitor	-3.462	8.32E-05	595 (15)
CTLA4	transmembrane receptor	-3.469	1.47E-02	
DACH1	transcription regulator	-3.56	2.14E-04	485 (12)
GFI1	transcription regulator	-3.596	1.73E-05	517 (11)
bexarotene	chemical drug	-3.741	6.96E-08	592 (15)
nifedipine	chemical drug	-3.81	1.72E-05	767 (22)
IL1RN	cytokine	-3.821	1.30E-04	490 (14)
SU6656	chemical toxicant	-3.843	1.09E-06	830 (23)
Sb202190	chemical - kinase inhibitor	-3.872	3.78E-05	568 (16)
SFTPA1	transporter	-3.981	2.16E-10	699 (14)
2-amino-5-phosphonovaleric acid	chemical - other	-4.008	2.28E-11	743 (23)
genistein	chemical drug	-4.018	1.70E-05	490 (17)

MYCN	transcription regulator	-4.284	1.55E-22	
actinomycin D	chemical drug	-4.346	1.37E-04	670 (15)
DUSP1	phosphatase	-4.619	1.05E-10	512 (15)
SB203580	chemical - kinase inhibitor	-5.289	1.89E-08	606 (14)
ESR1	ligand-dependent nuclear receptor	-5.546	1.47E-07	936 (21)
PD98059	chemical - kinase inhibitor	-5.94	2.43E-15	610 (15)
LY294002	chemical - kinase inhibitor	-6.434	1.75E-21	551 (13)
U0126	chemical - kinase inhibitor	-6.895	9.35E-19	588 (14)

S4Table3. Networks and associated network functions associated with reperfusion injury

ID	Score	Focus Molecules	Top Diseases and Functions
1	34	35	Auditory Disease, Carbohydrate Metabolism, Cell Morphology
2	31	34	RNA Post-Transcriptional Modification, Gene Expression, Protein Synthesis
3	31	34	Cancer, Cell Death and Survival, Organismal Injury and Abnormalities
4	31	34	Molecular Transport, Nucleic Acid Metabolism, Small Molecule Biochemistry
5	29	33	Cellular Function and Maintenance, Molecular Transport, Small Molecule Biochemistry
6	29	33	Tissue Morphology, Developmental Disorder, Hereditary Disorder
7	29	33	Post-Translational Modification, Auditory Disease, Dermatological Diseases and Conditions
8	29	33	Auditory Disease, Hereditary Disorder, Neurological Disease
9	29	33	Post-Translational Modification, Protein Synthesis, Neurological Disease
10	29	33	Developmental Disorder, Hereditary Disorder, Metabolic Disease
11	29	33	Cell-To-Cell Signaling and Interaction, Cellular Function and Maintenance, Nervous System Development and Function
12	29	33	Carbohydrate Metabolism, Small Molecule Biochemistry, Embryonic Development
13	29	33	Cellular Development, Cellular Growth and Proliferation, Cell Death and Survival
14	27	32	Post-Translational Modification, Developmental Disorder, Hereditary Disorder
15	27	32	DNA Replication, Recombination, and Repair, Small Molecule Biochemistry, Amino Acid Metabolism
16	27	32	Developmental Disorder, Hereditary Disorder, Organismal Injury and Abnormalities
17	27	32	RNA Post-Transcriptional Modification, Cellular Assembly and Organization, Cancer
18	27	32	Cell Signaling, Post-Translational Modification, Protein Synthesis
19	27	32	Cellular Development, Embryonic Development, Organismal Development
20	27	32	Post-Translational Modification, Protein Folding, Energy Production
21	27	32	Endocrine System Disorders, Metabolic Disease, Organismal Injury and Abnormalities
22	27	32	Cell-mediated Immune Response, Cellular Development, Cellular Function and Maintenance
23	25	31	Embryonic Development, Organismal Development, Cell Signaling

24	25	31	Post-Translational Modification, Cancer, Organismal Injury and Abnormalities
25	25	31	Cell-To-Cell Signaling and Interaction, Connective Tissue Development and Function, Cellular Assembly and Organization

S4Table4. Top Regulator effect network

ID	Consistency Score	Node Total	Regulator Total	Regulators	Target Total	Disease & Function Total	Diseases & Functions
1	5.196	29	1	sirolimus	27	1	cell death of osteosarcoma cells
2	4.903	28	1	MYCN	26	1	cell death of osteosarcoma cells
3	4.69	24	1	RICTOR	22	1	cell death of osteosarcoma cells
4	4	18	1	CCL5	16	1	recruitment of blood cells
5	4	18	1	CCL5	16	1	recruitment of cells
6	3.881	19	1	CpG oligonucleotide	17	1	activation of mononuclear leukocytes
7	3.881	19	1	okadaic acid	17	1	transcription of DNA
8	3.873	17	1	CCL5	15	1	recruitment of leukocytes
9	3.75	18	1	TAC1	16	1	activation of cells
10	3.75	18	1	Tnf (family)	16	1	recruitment of neutrophils
11	3.75	18	1	okadaic acid	16	1	cell movement of tumor cell lines
12	3.606	15	1	miR-155-5p (miRNAs w/seed UAAUGCU)	13	1	recruitment of neutrophils
13	3.5	18	1	F2RL1	16	1	activation of cells
14	3.479	12	1	C5AR1	10	1	recruitment of cells
15	3.474	16	1	COL18A1	14	1	growth of muscle tissue
16	3.474	16	1	COL18A1	14	1	proliferation of muscle cells
17	3.464	14	1	Tnf (family)	12	1	activation of neutrophils
18	3.357	17	1	RELA	15	1	activation of neutrophils
19	3.333	11	1	IL1B	9	1	mobilization of neutrophils
20	3.333	11	1	PF4	9	1	activation of neutrophils
21	3.333	11	1	PGF	9	1	chemotaxis
22	3.333	11	1	PGF	9	1	chemotaxis of cells
23	3.333	11	1	PGF	9	1	homing of cells
24	3.333	11	1	TNF	9	1	mobilization of neutrophils
25	3.328	15	1	MIF	13	1	expression of RNA
26	3.328	15	1	MIF	13	1	transcription
27	3.328	15	1	MIF	13	1	transcription of RNA
28	3.328	15	1	okadaic acid	13	1	migration of tumor cell lines

29	3.317	13	1	3M-001	11	1	activation of cells
30	3.317	13	1	Ap1	11	1	activation of neutrophils
31	3.317	13	1	IL17F	11	1	cell movement
32	3.317	13	1	IL17F	11	1	homing of cells
33	3.317	13	1	IL17F	11	1	migration of cells
34	3.317	13	1	IL1B	11	1	mobilization of myeloid cells
35	3.317	13	1	MEOX2	11	1	recruitment of phagocytes
36	3.317	13	1	PF4	11	1	cell movement of myeloid cells
37	3.317	13	1	PP2/AG1879 tyrosine kinase inhibitor	11	1	cell movement of tumor cell lines
38	3.317	13	1	TRADD	11	1	recruitment of phagocytes
39	3.317	13	1	ozone	11	1	cell movement
40	3.317	13	1	poly rI:rC-RNA	11	1	mobilization of myeloid cells
41	3.317	13	1	uric acid	11	1	mobilization of blood cells
42	3.182	10	1	C5AR1	8	1	stimulation of cells
43	3.182	10	1	C5AR1	8	1	stimulation of leukocytes
44	3.182	10	1	LCN2	8	1	activation of neutrophils
45	3.182	10	1	PF4	8	1	stimulation of cells
46	3.182	10	1	PGF	8	1	proliferation of connective tissue cells
47	3.182	10	1	RCAN1	8	1	recruitment of cells
48	3.175	14	1	5-hydroxytryptamine	12	1	recruitment of cells
49	3.175	14	1	E. coli B4 lipopolysaccharide	12	1	mobilization of blood cells
50	3.175	14	1	IFI16	12	1	cell movement of tumor cell lines
51	3.175	14	1	Jnk	12	1	activation of neutrophils
52	3.175	14	1	NFkB (complex)	12	1	mobilization of myeloid cells
53	3.175	14	1	TLR2	12	1	migration of muscle cells
54	3.175	14	1	TLR2	12	1	migration of smooth muscle cells
55	3.175	14	1	diphenyleneiodonium	12	1	activation of cells
56	3.175	14	1	lysophosphatidylcholine	12	1	chemotaxis of cells
57	3.162	12	1	3M-001	10	1	activation of blood cells

58	3.162	12	1	3M-001	10	1	activation of leukocytes
59	3.162	12	1	ALB	10	1	migration of tumor cell lines
60	3.162	12	1	CAMK4	10	1	transcription
61	3.162	12	1	CAMK4	10	1	transcription of RNA
62	3.162	12	1	F2R	10	1	cell movement of tumor cell lines
63	3.162	12	1	F2R	10	1	organization of cytoplasm
64	3.162	12	1	F2R	10	1	organization of cytoskeleton
65	3.162	12	1	F7	10	1	proliferation of muscle cells
66	3.162	12	1	IL17F	10	1	activation of blood cells
67	3.162	12	1	IL17F	10	1	activation of leukocytes
68	3.162	12	1	IL1B	10	1	mobilization of granulocytes
69	3.162	12	1	L-glutamic acid	10	1	cell movement of tumor cell lines
70	3.162	12	1	L-glutamic acid	10	1	survival of organism
71	3.162	12	1	Ni2+	10	1	cell movement of myeloid cells
72	3.162	12	1	Ni2+	10	1	cell survival
73	3.162	12	1	Ni2+	10	1	cell viability
74	3.162	12	1	Ni2+	10	1	recruitment of phagocytes
75	3.162	12	1	PF4	10	1	cell movement of neutrophils
76	3.162	12	1	PF4	10	1	development of connective tissue
77	3.162	12	1	PF4	10	1	development of connective tissue cells
78	3.162	12	1	aspirin	10	1	mobilization of blood cells
79	3.162	12	1	enterotoxin B	10	1	mobilization of blood cells
80	3.162	12	1	lipoarabinomannan	10	1	migration of cells
81	3.162	12	1	nifedipine	10	1	recruitment of cells
82	3.162	12	1	peptidoglycan	10	1	mobilization of myeloid cells
83	3.162	12	1	poly rI:rC-RNA	10	1	mobilization of granulocytes
84	3.162	12	1	trovafloxacin	10	1	recruitment of leukocytes
85	3.051	15	1	COL18A1	13	1	growth of smooth muscle
86	3.051	15	1	COL18A1	13	1	proliferation of smooth muscle cells
87	3.051	15	1	carbon monoxide	13	1	cell movement of myeloid cells

88	3.024	9	1	CCL4	7	1	recruitment of leukocytes
89	3.015	13	1	1-methyl-4-phenyl-1,2,3,6-tetrahydropyridine	11	1	proliferation of connective tissue cells
90	3.015	13	1	F7	11	1	generation of cells
91	3.015	13	1	MKNK1	11	1	expression of RNA
92	3.015	13	1	MKNK1	11	1	transcription
93	3.015	13	1	NLRP12	11	1	chemotaxis of cells
94	3.015	13	1	U0126	11	1	mobilization of myeloid cells
95	3.015	13	1	bromodeoxyuridine	11	1	activation of cells
96	3.015	13	1	lipoxin A4	11	1	recruitment of cells
97	3.015	13	1	quinolinic acid	11	1	expression of RNA
98	3.015	13	1	quinolinic acid	11	1	transcription
99	3	11	1	AG490	9	1	migration of smooth muscle cells
100	3	11	1	AGT	9	1	activation of neutrophils
101	3	11	1	CXCL2	9	1	chemotaxis of cells
102	3	11	1	ECSIT	9	1	transcription of DNA
103	3	11	1	ERK1/2	9	1	mobilization of myeloid cells
104	3	11	1	IL17A	9	1	mobilization of myeloid cells
105	3	11	1	IL1A	9	1	mobilization of myeloid cells
106	3	11	1	ITGB1	9	1	infection of mammalia
107	3	18	1	KLF2	16	1	recruitment of cells
108	3	11	1	MAP3K8	9	1	migration of smooth muscle cells
109	3	11	1	N-acetylmuramyl-L-alanyl-D-isoglutamine	9	1	migration of smooth muscle cells
110	3	11	1	NfkB1-RelA	9	1	activation of cells
111	3	11	1	NfkB1-RelA	9	1	migration of cells
112	3	11	1	PP1	9	1	expression of RNA
113	3	11	1	PRKCE	9	1	differentiation of bone
114	3	11	1	PRKCE	9	1	differentiation of bone cells
115	3	11	1	TLR3	9	1	mobilization of neutrophils

116	3	11	1	TREM1	9	1	mobilization of blood cells
117	3	11	1	TSC22D3	9	1	recruitment of phagocytes
118	3	11	1	alpha-tocopherol	9	1	migration of cells
119	3	11	1	carrageenan	9	1	cell movement of neutrophils
120	3	11	1	carrageenan	9	1	proliferation of muscle cells
121	3	11	1	etanercept	9	1	activation of mononuclear leukocytes
122	3	11	1	etanercept	9	1	recruitment of cells
123	3	11	1	leukotriene C4	9	1	cell movement
124	3	11	1	leukotriene C4	9	1	generation of cells
125	3	11	1	poly rI:rC-RNA	9	1	lipolysis
126	3	11	1	resolvin D1	9	1	activation of leukocytes
127	3	11	1	resolvin D1	9	1	migration of smooth muscle cells
128	3	11	1	resolvin D1	9	1	recruitment of neutrophils
129	3	11	1	stallimycin	9	1	activation of mononuclear leukocytes
130	3	11	1	triflusal	9	1	activation of leukocytes
131	3	11	1	triflusal	9	1	migration of cells
132	3	11	1	zymosan	9	1	mobilization of blood cells
133	2.985	24	1	RET	22	1	migration of cells
134	2.887	14	1	CXCR4	12	1	activation of cells
135	2.887	14	1	Ccl2	12	1	transcription
136	2.887	14	1	Ccl2	12	1	transcription of RNA
137	2.887	14	1	IL3	12	1	induction of cells
138	2.858	8	1	CXCL1	6	1	activation of cells
139	2.858	8	1	CXCL1	6	1	recruitment of leukocytes
140	2.858	8	1	CXCL3	6	1	cell movement of myeloid cells
141	2.858	8	1	CXCL3	6	1	chemotaxis of cells
142	2.858	8	1	CXCL3	6	1	recruitment of neutrophils
143	2.858	8	1	IL6	6	1	lipolysis
144	2.858	8	1	ITCH	6	1	activation of cells
145	2.858	8	1	MAPK10	6	1	cell movement

146	2.858	8	1	PF4	6	1	differentiation of antigen presenting cells
147	2.846	12	1	C5	10	1	mobilization of myeloid cells
148	2.846	12	1	E. coli lipopolysaccharide	10	1	mobilization of blood cells
149	2.846	12	1	IRF6	10	1	migration of tumor cell lines
150	2.846	12	1	KLF6	10	1	cell movement of neutrophils
151	2.846	12	1	KLF6	10	1	development of connective tissue cells
152	2.846	12	1	NfkB-RelA	10	1	cell movement of tumor cell lines
153	2.846	12	1	SASH1	10	1	activation of leukocytes
154	2.846	12	1	TLR7	10	1	mobilization of blood cells
155	2.846	12	1	TNFAIP3	10	1	activation of leukocytes
156	2.846	12	1	Tlr	10	1	mobilization of blood cells
157	2.846	12	1	aldosterone	10	1	differentiation of bone cells
158	2.846	12	1	cilostazol	10	1	migration of cells
159	2.846	12	1	dinoprost	10	1	development of connective tissue
160	2.846	12	1	fatty acid	10	1	development of connective tissue
161	2.846	12	1	fatty acid	10	1	proliferation of connective tissue cells
162	2.846	12	1	resiquimod	10	1	mobilization of blood cells
163	2.846	12	1	triptolide	10	1	recruitment of cells
164	2.828	10	1	AIMP1	8	1	mobilization of blood cells
165	2.828	10	1	AREG	8	1	growth of smooth muscle
166	2.828	10	1	AREG	8	1	proliferation of smooth muscle cells
167	2.828	10	1	CD 437	8	1	cell death of osteosarcoma cells
168	2.828	10	1	CXCL10	8	1	activation of cells
169	2.828	10	1	CXCL10	8	1	cell movement
170	2.828	10	1	E. coli B5 lipopolysaccharide	8	1	mobilization of neutrophils
171	2.828	10	1	FOXL2	8	1	chemotaxis of cells
172	2.828	10	1	Fcer1	8	1	mobilization of myeloid cells
173	2.828	10	1	IL-17f dimer	8	1	cell movement of tumor cell lines
174	2.828	10	1	IL15	8	1	mobilization of neutrophils

175	2.828	10	1	IL17C	8	1	activation of leukocytes
176	2.828	10	1	IL17C	8	1	recruitment of leukocytes
177	2.828	10	1	IL17a dimer	8	1	mobilization of blood cells
178	2.828	10	1	IL18	8	1	mobilization of myeloid cells
179	2.828	10	1	IL1	8	1	mobilization of myeloid cells
180	2.828	10	1	IL2	8	1	mobilization of neutrophils
181	2.828	10	1	IL37	8	1	activation of leukocytes
182	2.828	10	1	IL37	8	1	migration of cells
183	2.828	10	1	IL37	8	1	recruitment of cells
184	2.828	10	1	IL37	8	1	stimulation of cells
185	2.828	10	1	IL37	8	1	transcription
186	2.828	10	1	IL37	8	1	transcription of RNA
187	2.828	10	1	MALP-2s	8	1	migration of smooth muscle cells
188	2.828	10	1	NUPR1	8	1	stimulation of cells
189	2.828	10	1	PD98059	8	1	mobilization of neutrophils
190	2.828	10	1	Pam3-Cys	8	1	migration of smooth muscle cells
191	2.828	10	1	S-(2,3-bispalmitoyloxypropyl)-cysteine-GDPKHPKSF	8	1	activation of leukocytes
192	2.828	10	1	S-(2,3-bispalmitoyloxypropyl)-cysteine-GDPKHPKSF	8	1	chemotaxis of cells
193	2.828	10	1	S-(2,3-bispalmitoyloxypropyl)-cysteine-GDPKHPKSF	8	1	migration of cells
194	2.828	10	1	S-(2,3-bispalmitoyloxypropyl)-cysteine-GDPKHPKSF	8	1	recruitment of cells
195	2.828	10	1	SP600125	8	1	mobilization of myeloid cells
196	2.828	10	1	ST1926	8	1	cell death of osteosarcoma cells
197	2.828	10	1	Salmonella enterica serotype abortus equi lipopolysaccharide	8	1	mobilization of myeloid cells
198	2.828	10	1	TACR1	8	1	recruitment of leukocytes
199	2.828	10	1	TLR5	8	1	migration of smooth muscle cells
200	2.828	10	1	bisindolylmaleimide I	8	1	differentiation of bone cells

S4Table5. The list of the canonical pathways associated with   associated with perfusion driven IGF

**Canonical Pathways**	**-log(p-value)**	**Ratio**	**z-score**
Granulocyte Adhesion and Diapedesis	6.62E+00	6.15E-02	NaN
Agranulocyte Adhesion and Diapedesis	6.36E+00	5.79E-02	NaN
TREM1 Signaling	5.57E+00	9.21E-02	2.646
Glucocorticoid Receptor Signaling	5.49E+00	4.27E-02	NaN
IL-6 Signaling	5.33E+00	6.90E-02	1.414
HMGB1 Signaling	5.22E+00	6.67E-02	2.646
Differential Regulation of Cytokine Production in Macrophages and T Helper Cells by IL-17A and IL-17F	4.94E+00	2.22E-01	NaN
IGF-1 Signaling	4.80E+00	7.07E-02	-0.447
Role of Hypercytokinemia/hyperchemokinemia in the Pathogenesis of Influenza	4.61E+00	1.16E-01	NaN
Airway Pathology in Chronic Obstructive Pulmonary Disease	4.55E+00	3.75E-01	NaN
Prolactin Signaling	4.46E+00	7.89E-02	0.816
IL-17A Signaling in Gastric Cells	4.34E+00	1.60E-01	NaN
TNFR2 Signaling	4.08E+00	1.38E-01	NaN
Communication between Innate and Adaptive Immune Cells	4.04E+00	6.67E-02	NaN
PPAR Signaling	3.97E+00	6.45E-02	-1.633
Colorectal Cancer Metastasis Signaling	3.81E+00	3.73E-02	3
IL-17A Signaling in Fibroblasts	3.75E+00	1.14E-01	NaN
Aryl Hydrocarbon Receptor Signaling	3.73E+00	4.79E-02	NaN
Cholecystokinin/Gastrin-mediated Signaling	3.65E+00	5.66E-02	2.449
IL-10 Signaling	3.53E+00	6.94E-02	NaN
JAK/Stat Signaling	3.53E+00	6.94E-02	0.447

Role of IL-17F in Allergic Inflammatory Airway Diseases	3.36E+00	9.09E-02	NaN
Acute Phase Response Signaling	3.31E+00	4.09E-02	1.134
TNFR1 Signaling	3.18E+00	8.16E-02	NaN
NRF2-mediated Oxidative Stress Response	3.18E+00	3.89E-02	0.816
Role of Macrophages, Fibroblasts and Endothelial Cells in Rheumatoid Arthritis	3.09E+00	2.98E-02	NaN
Differential Regulation of Cytokine Production in Intestinal Epithelial Cells by IL-17A and IL-17F	3.09E+00	1.30E-01	NaN
Hepatic Fibrosis / Hepatic Stellate Cell Activation	3.08E+00	3.74E-02	NaN
ILK Signaling	3.08E+00	3.74E-02	2.449
IL-8 Signaling	3.07E+00	3.72E-02	2.646
Role of Cytokines in Mediating Communication between Immune Cells	3.02E+00	7.41E-02	NaN
Role of JAK family kinases in IL-6-type Cytokine Signaling	2.98E+00	1.20E-01	NaN
PCP pathway	2.77E+00	6.35E-02	1
HGF Signaling	2.73E+00	4.63E-02	NaN
CD40 Signaling	2.72E+00	6.15E-02	NaN
Pathogenesis of Multiple Sclerosis	2.65E+00	2.22E-01	NaN
Tight Junction Signaling	2.62E+00	3.59E-02	NaN
Erythropoietin Signaling	2.60E+00	5.71E-02	NaN
PXR/RXR Activation	2.58E+00	5.63E-02	NaN
p38 MAPK Signaling	2.58E+00	4.27E-02	0.447
IL-17 Signaling	2.56E+00	5.56E-02	NaN
Circadian Rhythm Signaling	2.55E+00	8.57E-02	NaN
Cardiac Hypertrophy Signaling	2.55E+00	3.02E-02	1.134
Growth Hormone Signaling	2.54E+00	5.48E-02	NaN
Corticotropin Releasing Hormone Signaling	2.52E+00	4.13E-02	NaN
CCR5 Signaling in Macrophages	2.52E+00	5.41E-02	NaN
Toll-like Receptor Signaling	2.52E+00	5.41E-02	NaN
Production of Nitric Oxide and Reactive Oxygen Species in Macrophages	2.39E+00	3.23E-02	2.449
Role of IL-17A in Psoriasis	2.32E+00	1.54E-01	NaN
D-myo-inositol (1,4,5,6)-Tetrakisphosphate Biosynthesis	2.32E+00	3.70E-02	NaN
D-myo-inositol (3,4,5,6)-tetrakisphosphate Biosynthesis	2.32E+00	3.70E-02	NaN

ErbB Signaling	2.28E+00	4.65E-02	NaN
MIF Regulation of Innate Immunity	2.27E+00	6.82E-02	NaN
iNOS Signaling	2.19E+00	6.38E-02	NaN
D-myo-inositol-5-phosphate Metabolism	2.10E+00	3.29E-02	NaN
3-phosphoinositide Degradation	2.09E+00	3.27E-02	NaN
p53 Signaling	2.08E+00	4.08E-02	NaN
Semaphorin Signaling in Neurons	2.05E+00	5.66E-02	NaN
CXCR4 Signaling	2.01E+00	3.12E-02	2
IL-2 Signaling	2.00E+00	5.45E-02	NaN
Role of Osteoblasts, Osteoclasts and Chondrocytes in Rheumatoid Arthritis	2.00E+00	2.67E-02	NaN
GADD45 Signaling	1.99E+00	1.05E-01	NaN
Role of IL-17A in Arthritis	1.98E+00	5.36E-02	NaN
Thrombopoietin Signaling	1.92E+00	5.08E-02	NaN
ATM Signaling	1.92E+00	5.08E-02	NaN
3-phosphoinositide Biosynthesis	1.92E+00	2.96E-02	NaN
Type I Diabetes Mellitus Signaling	1.90E+00	3.60E-02	0
Role of Tissue Factor in Cancer	1.89E+00	3.57E-02	NaN
Activation of IRF by Cytosolic Pattern Recognition Receptors	1.86E+00	4.84E-02	NaN
Hepatic Cholestasis	1.85E+00	2.84E-02	NaN
IL-15 Signaling	1.82E+00	4.69E-02	NaN
ERK5 Signaling	1.82E+00	4.69E-02	NaN
Mitotic Roles of Polo-Like Kinase	1.79E+00	4.55E-02	NaN
Estrogen-Dependent Breast Cancer Signaling	1.77E+00	4.48E-02	NaN
Sertoli Cell-Sertoli Cell Junction Signaling	1.75E+00	2.69E-02	NaN
Role of Pattern Recognition Receptors in Recognition of Bacteria and Viruses	1.73E+00	3.20E-02	NaN
ERK/MAPK Signaling	1.71E+00	2.62E-02	0.447
IL-15 Production	1.70E+00	7.41E-02	NaN
STAT3 Pathway	1.66E+00	4.05E-02	NaN
Chemokine Signaling	1.64E+00	4.00E-02	NaN
Adipogenesis pathway	1.62E+00	2.96E-02	NaN

IL-12 Signaling and Production in Macrophages	1.60E+00	2.92E-02	NaN
VDR/RXR Activation	1.58E+00	3.80E-02	NaN
Regulation of IL-2 Expression in Activated and Anergic T Lymphocytes	1.55E+00	3.70E-02	NaN
PDGF Signaling	1.53E+00	3.61E-02	NaN
Ceramide Signaling	1.51E+00	3.57E-02	NaN
IL-9 Signaling	1.51E+00	5.88E-02	NaN
Role of JAK2 in Hormone-like Cytokine Signaling	1.51E+00	5.88E-02	NaN
Superpathway of Inositol Phosphate Compounds	1.51E+00	2.31E-02	NaN
Systemic Lupus Erythematosus Signaling	1.49E+00	2.29E-02	NaN
Bladder Cancer Signaling	1.48E+00	3.45E-02	NaN
TGF-Î² Signaling	1.48E+00	3.45E-02	NaN
Interferon Signaling	1.47E+00	5.56E-02	NaN
Glioblastoma Multiforme Signaling	1.47E+00	2.65E-02	1
Apoptosis Signaling	1.45E+00	3.37E-02	NaN
Crosstalk between Dendritic Cells and Natural Killer Cells	1.44E+00	3.33E-02	NaN
GÎ±q Signaling	1.43E+00	2.58E-02	0
April Mediated Signaling	1.42E+00	5.26E-02	NaN
Neuregulin Signaling	1.40E+00	3.23E-02	NaN
Airway Inflammation in Asthma	1.40E+00	2.00E-01	NaN
Tec Kinase Signaling	1.39E+00	2.50E-02	NaN
Role of PKR in Interferon Induction and Antiviral Response	1.38E+00	5.00E-02	NaN
B Cell Activating Factor Signaling	1.38E+00	5.00E-02	NaN
SAPK/JNK Signaling	1.38E+00	3.16E-02	NaN
Protein Kinase A Signaling	1.37E+00	1.76E-02	-.134
UVC-Induced MAPK Signaling	1.35E+00	4.76E-02	NaN

S4Table6. The complete list of activated and inhibited upstream regulators associated with perfusion driven IGF

Upstream Regulator	Molecule Type	Activation z-score	p-value of overlap
**Activated**			
TNF	cytokine	7.078	7.18E-44
lipopolysaccharide	chemical drug	6.713	1.11E-40
PDGF BB	complex	6.565	2.49E-47
phorbol myristate acetate	chemical drug	6.071	1.44E-30
IL1B	cytokine	5.881	1.42E-40
IFNG	cytokine	5.774	1.68E-19
NFkB (complex)	complex	5.721	1.43E-26
poly rI:rC-RNA	biologic drug	5.636	1.11E-28
EGF	growth factor	5.273	1.09E-27
Salmonella enterica serotype abortus equi lipopolysaccharide	chemical toxicant	5.246	1.46E-29
F2	peptidase	5.019	2.27E-23
CREB1	transcription regulator	5.007	1.24E-29
leukotriene D4	chemical - endogenous mammalian	4.982	2.84E-41
TLR3	transmembrane receptor	4.753	7.60E-24
hydrogen peroxide	chemical - endogenous mammalian	4.736	4.26E-19
salmonella minnesota R595 lipopolysaccharides	chemical - endogenous non-mammalian	4.681	6.09E-26
ERK	group	4.656	7.04E-26
IL6	cytokine	4.645	2.38E-17
GnRH-A	chemical reagent	4.518	4.31E-28
IL2	cytokine	4.516	1.20E-17
E. coli B5 lipopolysaccharide	chemical - endogenous non-mammalian	4.505	1.17E-20

P38 MAPK	group	4.492	1.08E-20
IL1	group	4.486	8.78E-16
thapsigargin	chemical toxicant	4.485	4.44E-21
dalfampridine	chemical drug	4.472	2.41E-35
forskolin	chemical toxicant	4.443	4.28E-26
cisplatin	chemical drug	4.427	1.42E-18
bicuculline	chemical - endogenous non-mammalian	4.396	7.03E-33
doxorubicin	chemical drug	4.389	8.42E-20
TLR4	transmembrane receptor	4.365	1.62E-24
CSF2	cytokine	4.354	4.26E-22
TGFB1	growth factor	4.341	1.33E-23
kainic acid	chemical toxicant	4.312	2.32E-21
TREM1	transmembrane receptor	4.288	7.50E-25
HGF	growth factor	4.281	6.58E-20
TICAM1	other	4.227	1.06E-21
IL3	cytokine	4.173	8.53E-15
FGF2	growth factor	4.153	1.89E-15
deferoxamine	chemical drug	4.135	4.54E-20
5-O-mycolyl-beta-araf-(1->2)-5-O-mycolyl-alpha-araf-(1->1')-glycerol	chemical - endogenous non-mammalian	4.123	7.57E-20
TLR9	transmembrane receptor	4.122	1.14E-17
CD40LG	cytokine	4.043	1.10E-18
ERK1/2	group	4.025	2.08E-16
IL17A	cytokine	4.019	4.18E-18
gentamicin	chemical drug	4	5.91E-11
TNFSF11	cytokine	3.993	1.08E-19
Jnk	group	3.962	2.27E-22
MYD88	other	3.944	1.45E-22
IGF1	growth factor	3.903	3.94E-17

ionomycin	chemical reagent	3.9	2.21E-13
palmitic acid	chemical - endogenous mammalian	3.891	8.54E-14
Tlr	group	3.887	1.25E-17
peptidoglycan	chemical - endogenous non-mammalian	3.885	5.82E-17
TCR	complex	3.881	2.32E-20
SAMSN1	other	3.873	2.60E-16
IL1A	cytokine	3.82	4.68E-15
STAT4	transcription regulator	3.806	1.23E-10
TP53	transcription regulator	3.786	3.36E-20
A23187	chemical reagent	3.78	4.27E-15
cocaine	chemical drug	3.778	2.91E-18
camptothecin	chemical drug	3.761	2.05E-17
C5	cytokine	3.754	4.22E-16
cigarette smoke	chemical toxicant	3.731	1.03E-17
Ca2+	chemical - endogenous mammalian	3.719	1.57E-19
AGT	growth factor	3.712	3.43E-14
APP	other	3.684	3.54E-13
RELA	transcription regulator	3.677	4.57E-21
NUPR1	transcription regulator	3.674	7.44E-13
TLR7	transmembrane receptor	3.657	1.52E-13
MAP2K1/2	group	3.652	3.86E-17
STAT3	transcription regulator	3.651	2.63E-17
IL18	cytokine	3.646	2.62E-12
tunicamycin	chemical - endogenous non-mammalian	3.646	3.00E-10
IL12 (complex)	complex	3.64	2.96E-12
carrageenan	chemical - endogenous non-mammalian	3.63	2.19E-20
RAF1	kinase	3.604	4.95E-13

trovafloxacin	chemical drug	3.592	3.50E-15
MET	kinase	3.592	3.75E-15
Cg	complex	3.591	2.38E-14
TLR2	transmembrane receptor	3.553	5.24E-13
E. coli B4 lipopolysaccharide	chemical toxicant	3.547	3.26E-10
FOXO1	transcription regulator	3.543	1.21E-15
IL15	cytokine	3.54	4.59E-12
lysophosphatidic acid	chemical - other	3.524	4.01E-18
EGR1	transcription regulator	3.5	5.78E-21
cyclic AMP	chemical - endogenous mammalian	3.5	5.41E-16
IL5	cytokine	3.472	4.58E-11
VEGFA	growth factor	3.411	7.38E-10
5-hydroxytryptamine	chemical - endogenous mammalian	3.409	1.38E-13
MAPK3	kinase	3.395	1.91E-18
carbon tetrachloride	chemical toxicant	3.385	9.52E-17
anisomycin	chemical - endogenous non-mammalian	3.382	8.28E-19
resiquimod	chemical drug	3.381	7.60E-15
CXCL12	cytokine	3.38	1.61E-09
EDN1	cytokine	3.364	2.45E-10
25-hydroxycholesterol	chemical reagent	3.341	5.32E-19
Vegf	group	3.316	6.04E-15
norepinephrine	chemical - endogenous mammalian	3.313	3.38E-10
OSM	cytokine	3.304	4.74E-10
LIF	cytokine	3.285	3.93E-17
F2R	g-protein coupled receptor	3.264	5.38E-13
NRG1	other	3.263	1.39E-17
tretinoin	chemical - endogenous mammalian	3.248	1.16E-12
F7	peptidase	3.22	3.61E-16
beta-estradiol	chemical - endogenous mammalian	3.215	4.24E-32

Ap1	complex	3.208	2.23E-14
phorbol esters	chemical - other	3.208	3.74E-12
PTPRJ	phosphatase	3.207	2.73E-20
DOCK8	other	3.162	1.05E-10
SASH1	other	3.162	1.40E-10
tributyrin	chemical drug	3.132	5.65E-11
FOXO3	transcription regulator	3.131	8.14E-16
uric acid	chemical - endogenous mammalian	3.13	1.72E-09
okadaic acid	chemical toxicant	3.127	1.28E-16
LEP	growth factor	3.126	9.67E-11
Pkc(s)	group	3.119	2.39E-20
NOD2	other	3.117	4.01E-12
IKBKB	kinase	3.115	5.66E-15
E. coli lipopolysaccharide	chemical - endogenous non-mammalian	3.105	7.73E-11
10E,12Z-octadecadienoic acid	chemical - endogenous mammalian	3.104	1.41E-12
TBK1	kinase	3.101	6.05E-10
Nfat (family)	group	3.098	7.06E-11
CD14	transmembrane receptor	3.095	2.32E-13
reactive oxygen species	chemical toxicant	3.088	6.25E-12
Pdgf (complex)	complex	3.086	2.63E-09
zymosan	chemical - endogenous non-mammalian	3.078	1.19E-11
CpG ODN 1668	chemical reagent	3.07	9.86E-12
MAP3K8	kinase	3.068	1.17E-09
TAC1	other	3.067	6.84E-10
CCL5	cytokine	3.055	7.71E-15
Map3k7	kinase	3.047	5.38E-13
FOXL2	transcription regulator	3.046	6.03E-21
MAPK1	kinase	3.043	1.13E-13

AKT1	kinase	3.042	3.38E-09
LDL	complex	3.037	6.06E-23
L-dopa	chemical - endogenous mammalian	3.021	5.08E-05
Mek	group	3.02	1.62E-12
IL27	cytokine	3.008	9.24E-13
MAP2K1	kinase	2.998	1.53E-14
PAF1	other	2.985	6.72E-11
Tnf (family)	group	2.98	8.47E-10
cis-urocanic acid	chemical drug	2.971	2.88E-14
F3	transmembrane receptor	2.961	9.09E-10
TGM2	enzyme	2.961	8.73E-06
ICAM1	transmembrane receptor	2.957	1.80E-11
SB 216763	chemical toxicant	2.952	4.48E-15
MAPK8	kinase	2.951	9.03E-12
methyl methanesulfonate	chemical toxicant	2.95	2.86E-11
PTGS2	enzyme	2.948	6.34E-14
EGFR	kinase	2.944	4.56E-20
trinitrobenzenesulfonic acid	chemical reagent	2.944	2.64E-08
mitomycin C	chemical drug	2.941	6.71E-10
TGFA	growth factor	2.939	1.03E-08
KITLG	growth factor	2.938	9.07E-06
SMAD3	transcription regulator	2.937	7.01E-14
EIF2AK2	kinase	2.932	1.01E-13
platelet activating factor	chemical - endogenous mammalian	2.931	1.76E-10
PRKCE	kinase	2.926	1.85E-09
sphingosine-1-phosphate	chemical - endogenous mammalian	2.924	1.43E-08
MAP3K1	kinase	2.923	2.51E-10
IFN Beta	group	2.923	5.93E-09
FN1	enzyme	2.912	4.23E-07
ozone	chemical toxicant	2.911	1.77E-13

formaldehyde	chemical - endogenous mammalian	2.905	3.85E-13
D-galactosamine	chemical - endogenous mammalian	2.905	4.14E-13
fatty acid	chemical - endogenous mammalian	2.905	3.21E-08
D-glucose	chemical - endogenous mammalian	2.903	6.57E-11
thioacetamide	chemical toxicant	2.897	6.07E-09
Mapk	group	2.892	2.44E-14
Pam3-Cys-Ser-Lys4	chemical reagent	2.892	5.60E-12
CpG oligonucleotide	chemical drug	2.891	2.10E-10
Lh	complex	2.883	5.55E-13
L-glutamic acid	chemical - endogenous mammalian	2.879	5.11E-08
NFATC2	transcription regulator	2.875	2.00E-14
SRC	kinase	2.875	2.60E-07
JAK2	kinase	2.874	3.00E-07
HIF1A	transcription regulator	2.868	8.46E-15
CTNNB1	transcription regulator	2.867	3.57E-06
nitric oxide	chemical - endogenous mammalian	2.834	1.82E-11
JUN	transcription regulator	2.83	8.75E-13
oblimersen	biologic drug	2.828	6.79E-09
gentamicin C	chemical drug	2.828	2.39E-08
leukotriene C4	chemical - endogenous mammalian	2.823	5.39E-14
EP300	transcription regulator	2.819	1.74E-08
NFKB1	transcription regulator	2.818	1.46E-16
IL22	cytokine	2.813	4.02E-07
acetaminophen	chemical drug	2.804	5.24E-13
di(2-ethylhexyl) phthalate	chemical toxicant	2.804	9.16E-09
triamterene	chemical drug	2.804	3.47E-08
fenamic acid	chemical reagent	2.8	4.96E-08
NGF	growth factor	2.793	8.81E-12
TRADD	other	2.789	2.40E-11
MAPK14	kinase	2.773	5.42E-09

potassium chloride	chemical drug	2.769	1.38E-12
Gm-csf	group	2.769	5.44E-12
PDGFB	growth factor	2.768	2.98E-10
DDX58	enzyme	2.763	7.90E-09
MAPK7	kinase	2.76	1.04E-10
quinolinic acid	chemical - endogenous mammalian	2.758	3.57E-11
MALP-2s	chemical reagent	2.735	1.22E-11
MIF	cytokine	2.733	1.61E-11
ELK1	transcription regulator	2.728	2.88E-12
BMP2	growth factor	2.725	2.42E-07
IL21	cytokine	2.722	7.94E-08
tamoxifen	chemical drug	2.721	8.13E-12
paclitaxel	chemical drug	2.719	2.12E-18
PI3K (complex)	complex	2.717	1.29E-18
etoposide	chemical drug	2.715	1.96E-10
mycophenolic acid	chemical drug	2.714	4.23E-11
E. coli serotype 0127B8 lipopolysaccharide	chemical - endogenous non-mammalian	2.707	1.67E-07
NFKBIA	transcription regulator	2.705	2.91E-15
decitabine	chemical drug	2.677	8.84E-11
bucladesine	chemical toxicant	2.659	7.57E-19
CSF1	cytokine	2.658	2.53E-12
ARHGAP21	other	2.646	3.11E-08
lomustine	chemical drug	2.646	5.24E-07
Ras	group	2.645	2.39E-09
BCR (complex)	complex	2.639	1.68E-09
BDNF	growth factor	2.637	1.00E-09
phenacetin	chemical drug	2.63	2.62E-08
ERBB2	kinase	2.623	1.66E-11
EPHB1	kinase	2.621	1.43E-13

C5	other	2.621	2.21E-11
ECSIT	transcription regulator	2.621	9.48E-10
2-bromoethylamine	chemical reagent	2.621	7.97E-08
MAP2K4	kinase	2.618	2.06E-07
SP1	transcription regulator	2.617	2.93E-13
CHRM1	g-protein coupled receptor	2.613	9.30E-13
PF4	cytokine	2.613	3.76E-10
3M-001	chemical drug	2.613	1.03E-08
CD86	transmembrane receptor	2.611	5.91E-08
Fcer1	complex	2.61	6.71E-10
HMGB1	transcription regulator	2.61	1.87E-06
3M-011	chemical reagent	2.608	4.39E-09
EPAS1	transcription regulator	2.607	2.31E-08
BMP6	growth factor	2.607	4.02E-07
cobalt chloride	chemical reagent	2.607	1.07E-06
IRF6	transcription regulator	2.604	6.78E-10
TLR5	transmembrane receptor	2.602	1.03E-08
IRF8	transcription regulator	2.594	5.96E-07
carbamylcholine	chemical drug	2.592	1.26E-08
dinoprost	chemical - endogenous mammalian	2.585	1.71E-06
IL7	cytokine	2.584	1.94E-12
SMARCA4	transcription regulator	2.584	4.13E-07
HOXA5	transcription regulator	2.578	8.65E-11
aldosterone	chemical - endogenous mammalian	2.576	4.13E-05
N-acetylmuramyl-L-alanyl-D-isoglutamine	chemical - endogenous non-mammalian	2.575	1.59E-07
TGFB3	growth factor	2.572	1.23E-05
lipoteichoic acid	chemical - endogenous non-mammalian	2.569	5.24E-07
hyaluronic acid	chemical - endogenous mammalian	2.565	2.78E-15

IL33	cytokine	2.563	4.72E-10
F2RL1	g-protein coupled receptor	2.562	7.90E-09
REL	transcription regulator	2.56	2.30E-10
imiquimod	chemical drug	2.552	4.16E-10
CREM	transcription regulator	2.548	8.11E-24
CEBPB	transcription regulator	2.546	2.83E-12
HRAS	enzyme	2.531	3.63E-10
AGER	transmembrane receptor	2.525	1.40E-10
IRF5	transcription regulator	2.515	8.82E-07
amphetamine	chemical drug	2.512	2.82E-07
CD40	transmembrane receptor	2.504	6.37E-11
TNFSF12	cytokine	2.493	5.76E-06
GH1	growth factor	2.454	6.56E-10
ST3-Hel2A-2	chemical reagent	2.449	7.46E-11
lipoarabinomannan	chemical - endogenous non-mammalian	2.449	2.68E-10
KRT17	other	2.449	8.51E-08
NEDD9	other	2.449	2.69E-07
hexachlorobenzene	chemical toxicant	2.449	4.40E-06
MKNK1	kinase	2.449	1.17E-04
CHUK	kinase	2.448	8.64E-17
GNRH	group	2.447	2.00E-08
NfkB-RelA	complex	2.441	1.51E-08
crocidolite asbestos	chemical toxicant	2.436	5.47E-08
C3	peptidase	2.435	5.39E-06
ATF2	transcription regulator	2.43	5.35E-14
EIF2AK3	kinase	2.426	2.04E-10
stearic acid	chemical - endogenous mammalian	2.425	2.00E-08
CDK9	kinase	2.423	3.04E-09
PRKCB	kinase	2.423	2.06E-07

PRKCA	kinase	2.42	6.40E-09
cyclopiazonic acid	chemical - endogenous non-mammalian	2.416	1.25E-09
ELANE	peptidase	2.416	1.05E-07
ATF4	transcription regulator	2.415	6.93E-09
C5AR1	g-protein coupled receptor	2.415	8.06E-07
IFI16	transcription regulator	2.414	2.18E-05
IRF7	transcription regulator	2.412	1.49E-03
GRP	growth factor	2.41	2.62E-08
MAPK10	kinase	2.409	7.46E-11
N-acetylsphingosine	chemical reagent	2.408	9.27E-07
RETN	other	2.407	2.62E-08
CP-55940	chemical reagent	2.402	3.54E-10
sulindac sulfide	chemical drug	2.402	1.59E-05
IPMK	kinase	2.401	1.12E-08
9,10-dimethyl-1,2-benzanthracene	chemical toxicant	2.401	2.62E-08
colchicine	chemical drug	2.401	2.54E-06
methylnitronitrosoguanidine	chemical toxicant	2.4	6.85E-08
enterotoxin B	biologic drug	2.397	1.59E-05
AREG	growth factor	2.395	6.85E-08
bromodeoxyuridine	chemical drug	2.395	2.85E-06
CREBBP	transcription regulator	2.394	1.43E-12
Pam3-Cys	chemical toxicant	2.392	3.76E-07
CpG ODN 1826	chemical reagent	2.39	1.40E-08
IL12 (family)	group	2.39	4.27E-06
Fc gamma receptor	group	2.385	2.74E-09
hemozoin	chemical - endogenous non-mammalian	2.382	4.32E-08
LCN2	transporter	2.379	6.56E-06
SYK	kinase	2.377	6.55E-12

PTH	other	2.376	1.09E-16
vancomycin	biologic drug	2.376	1.76E-08
dopamine	chemical - endogenous mammalian	2.376	1.30E-04
silicon dioxide	chemical drug	2.375	1.25E-09
CAMK4	kinase	2.375	1.05E-07
GAST	other	2.369	2.35E-05
CD 437	chemical drug	2.367	4.54E-03
Fibrinogen	complex	2.366	8.23E-09
vinblastine	chemical drug	2.359	4.32E-08
PDX1	transcription regulator	2.355	8.62E-09
CXCR4	g-protein coupled receptor	2.353	3.96E-06
Pka	complex	2.346	5.07E-15
5-azacytidine	chemical drug	2.345	1.19E-06
Akt	group	2.344	6.11E-17
Interferon alpha	group	2.34	1.05E-08
TSH	complex	2.324	3.53E-10
EPO	cytokine	2.319	3.38E-08
DETA-NONOate	chemical reagent	2.3	1.00E-10
8-bromo-cAMP	chemical reagent	2.269	9.20E-09
Ccl2	cytokine	2.263	7.90E-09
PIK3R1	kinase	2.243	1.40E-10
GNRH1	other	2.243	1.06E-08
bleomycin	chemical drug	2.242	3.66E-07
D-sphingosine	chemical - endogenous mammalian	2.236	1.73E-08
Pkg	group	2.236	1.80E-07
CYR61	other	2.236	3.76E-07
carboplatin	chemical drug	2.236	3.67E-05
SPIB	transcription regulator	2.236	4.35E-04
TET2	other	2.236	1.79E-03
mono-(2-ethylhexyl)phthalate	chemical toxicant	2.236	8.10E-03

N-formyl-Met-Leu-Phe	chemical reagent	2.232	1.55E-05
phorbol 12,13-dibutyrate	chemical - endogenous non-mammalian	2.23	5.31E-06
ceruletide	biologic drug	2.229	5.40E-07
lysophosphatidylcholine	chemical - other	2.228	5.49E-10
4-methylnitrosoamino-1-(3-pyridinyl)-1-butanone	chemical toxicant	2.225	3.30E-06
TRPV4	ion channel	2.224	8.61E-07
PLC	group	2.219	7.88E-10
CD244	transmembrane receptor	2.219	1.25E-09
CSF1R	kinase	2.219	4.32E-08
PADI2	enzyme	2.219	6.43E-08
ANXA2	other	2.219	2.78E-06
ethionine	chemical toxicant	2.219	1.07E-05
ETS2	transcription regulator	2.219	1.04E-04
pCPT-cAMP	chemical - kinase inhibitor	2.216	5.56E-09
ADCYAP1R1	g-protein coupled receptor	2.213	4.72E-10
FGFR1	kinase	2.207	5.41E-05
INHBA	growth factor	2.205	1.24E-09
NOD1	other	2.204	2.79E-08
CXCL3	cytokine	2.201	9.30E-08
asbestos	chemical toxicant	2.2	2.80E-09
TET1	other	2.2	5.40E-07
LCK	kinase	2.2	2.78E-06
PPRC1	transcription regulator	2.2	2.18E-05
homocysteine	chemical - endogenous mammalian	2.196	2.64E-06
AIMP1	cytokine	2.195	6.43E-08
BCL10	transcription regulator	2.194	7.88E-10
CAMP	other	2.193	1.29E-08
STAT1	transcription regulator	2.191	2.97E-14
IRF1	transcription regulator	2.191	1.70E-04

NCR2	transmembrane receptor	2.19	1.31E-07
stallimycin	biologic drug	2.19	2.43E-05
trichostatin A	chemical drug	2.189	1.20E-08
ALB	transporter	2.189	1.95E-05
N-methyl-D-aspartate	chemical reagent	2.189	2.43E-05
RIPK2	kinase	2.188	1.85E-04
DDIT3	transcription regulator	2.186	6.37E-05
S-(2,3-bispalmitoyloxypropyl)-cysteine-GDPKHPKSF	chemical reagent	2.185	1.80E-07
IL32	cytokine	2.184	4.44E-05
TNFSF13B	cytokine	2.183	3.67E-05
MTPN	transcription regulator	2.18	1.95E-07
STAT	group	2.18	2.69E-07
clozapine	chemical drug	2.179	9.63E-05
NfkB1-RelA	complex	2.178	1.32E-06
TRAF6	enzyme	2.177	4.88E-05
MAP3K3	kinase	2.176	8.61E-07
HSPD1	enzyme	2.176	8.61E-07
dibutyl phthalate	chemical toxicant	2.176	1.07E-06
FSH	complex	2.174	1.64E-18
CLEC7A	transmembrane receptor	2.172	8.61E-07
PGF	growth factor	2.169	6.43E-08
TRH	other	2.169	7.10E-06
lauric acid	chemical - endogenous mammalian	2.161	6.43E-08
IL4	cytokine	2.158	3.95E-15
NR5A2	ligand-dependent nuclear receptor	2.158	8.68E-10
Endothelin	group	2.157	1.31E-07
Ifn	group	2.155	2.79E-07
cyclophosphamide	chemical drug	2.154	8.66E-08
streptozocin	chemical drug	2.144	3.06E-05
Il3	cytokine	2.138	4.41E-08

BMP4	growth factor	2.138	8.75E-07
nitrofurantoin	chemical drug	2.137	5.52E-08
IGF1R	transmembrane receptor	2.133	4.19E-05
EZH2	transcription regulator	2.129	2.17E-11
PLG	peptidase	2.129	2.26E-04
S100A9	other	2.128	2.69E-10
NFYA	transcription regulator	2.121	6.05E-10
methapyrilene	chemical drug	2.121	1.07E-05
histamine	chemical - endogenous mammalian	2.108	2.74E-14
Ifn gamma	complex	2.088	6.14E-05
methamphetamine	chemical drug	2.078	6.96E-08
cycloheximide	chemical reagent	2.058	1.44E-27
IFNA2	cytokine	2.056	9.37E-07
ESR2	ligand-dependent nuclear receptor	2.047	8.62E-12
IL6R	transmembrane receptor	2.036	2.36E-13
Insulin	group	2.021	1.05E-12
tert-butyl-hydroquinone	chemical reagent	2.016	2.23E-06
IKBKG	kinase	2.005	1.44E-10
NEK6	kinase	2	3.79E-07
misoprostol	chemical drug	2	5.25E-06
NTRK1	kinase	2	2.04E-05
tosedostat	chemical drug	2	7.28E-05
Collagen type I	complex	2	1.21E-04
PIN1	enzyme	2	6.79E-04
seocalcitol	chemical drug	2	1.02E-03
SYVN1	transporter	2	1.91E-02
ciprofibrate	chemical drug	2	2.46E-02
**Inhibited**			
U0126	chemical - kinase inhibitor	-6.13	4.18E-40

PD98059	chemical - kinase inhibitor	-5.758	9.36E-25
LY294002	chemical - kinase inhibitor	-5.305	5.65E-37
SB203580	chemical - kinase inhibitor	-4.415	1.93E-18
2-amino-5-phosphonovaleric acid	chemical - other	-4.246	8.60E-23
SP600125	chemical - kinase inhibitor	-4.179	3.20E-19
CD3	complex	-3.681	2.35E-12
SFTPA1	transporter	-3.643	2.47E-19
miR-155-5p (miRNAs w/seed UAAUGCU)	mature microrna	-3.642	1.08E-11
wortmannin	chemical - kinase inhibitor	-3.543	9.75E-13
Hdac	group	-3.31	6.70E-17
triamcinolone acetonide	chemical drug	-3.293	3.38E-14
DACH1	transcription regulator	-3.274	3.24E-15
KLF2	transcription regulator	-3.25	1.18E-08
N-acetyl-L-cysteine	chemical drug	-3.198	3.67E-10
Sb202190	chemical - kinase inhibitor	-3.158	2.19E-09
DUSP1	phosphatase	-3.1	1.10E-15
bexarotene	chemical drug	-3.087	3.52E-08
curcumin	chemical drug	-2.991	1.81E-12
SU6656	chemical toxicant	-2.975	2.08E-12
COL18A1	other	-2.952	2.39E-08
actinomycin D	chemical drug	-2.928	5.77E-13
Bay 11-7082	chemical - kinase inhibitor	-2.889	4.16E-10
genistein	chemical drug	-2.863	2.45E-12
Alpha catenin	group	-2.861	3.33E-13
Nr1h	group	-2.777	1.19E-05
herbimycin	chemical - kinase inhibitor	-2.775	1.79E-09
geldanamycin	chemical - endogenous non-mammalian	-2.711	1.31E-05
IL1RN	cytokine	-2.706	1.06E-11
VIP	other	-2.653	5.66E-14

MEOX2	transcription regulator	-2.646	1.52E-08
ACOX1	enzyme	-2.646	1.12E-04
apigenin	chemical - endogenous non-mammalian	-2.635	9.63E-08
FOSL1	transcription regulator	-2.622	1.50E-16
baicalein	chemical - endogenous non-mammalian	-2.607	3.67E-08
tyrphostin AG 1478	chemical - kinase inhibitor	-2.6	7.20E-07
Sn50 peptide	chemical toxicant	-2.597	3.34E-07
Ro31-8220	chemical - kinase inhibitor	-2.587	3.11E-08
LYN	kinase	-2.581	1.77E-07
carbon monoxide	chemical - endogenous mammalian	-2.575	2.97E-07
amino acids	chemical - endogenous mammalian	-2.56	8.50E-12
resolvin D1	chemical - endogenous mammalian	-2.549	5.66E-11
bisindolylmaleimide I	chemical - kinase inhibitor	-2.513	2.28E-10
NS-398	chemical reagent	-2.508	2.14E-07
CD28	transmembrane receptor	-2.499	1.09E-14
AG490	chemical - kinase inhibitor	-2.481	6.84E-10
DUSP5	phosphatase	-2.449	5.91E-09
iron	chemical - endogenous mammalian	-2.449	2.85E-06
CGS 21680	chemical reagent	-2.433	2.69E-07
THRA	ligand-dependent nuclear receptor	-2.433	1.63E-06
PP1	chemical - kinase inhibitor	-2.425	3.76E-07
tosylphenylalanyl chloromethyl ketone	chemical - protease inhibitor	-2.423	8.23E-09
CORT	other	-2.418	1.28E-07
pyrrolidine dithiocarbamate	chemical reagent	-2.417	1.22E-12
triptolide	chemical drug	-2.417	5.74E-10
bardoxolone methyl	chemical drug	-2.406	8.51E-08
calphostin C	chemical - kinase inhibitor	-2.404	4.70E-07
SERCA	group	-2.401	1.67E-13

sulforafan	chemical drug	-2.398	3.57E-04
SOCS3	phosphatase	-2.353	5.17E-09
sirolimus	chemical drug	-2.317	2.09E-07
losartan potassium	chemical drug	-2.297	1.52E-06
docosahexaenoic acid	chemical drug	-2.268	3.07E-07
prazosin	chemical drug	-2.236	2.74E-10
nimodipine	chemical drug	-2.236	6.43E-08
USP18	peptidase	-2.236	1.80E-07
MNT	transcription regulator	-2.236	2.43E-07
GPX1	enzyme	-2.236	1.74E-05
mir-146	microrna	-2.226	6.78E-10
tempol	chemical drug	-2.216	4.55E-06
edaravone	chemical drug	-2.213	5.56E-09
GADD45A	other	-2.213	5.40E-07
ZFP36	transcription regulator	-2.207	7.30E-14
CEP-1347	chemical drug	-2.207	2.43E-07
Go 6976	chemical - kinase inhibitor	-2.205	4.44E-05
ENTPD1	enzyme	-2.2	2.80E-09
PS-1145	chemical - kinase inhibitor	-2.2	2.43E-07
2-aminopurine	chemical reagent	-2.2	6.86E-07
ADA	enzyme	-2.2	1.61E-06
nifedipine	chemical drug	-2.2	2.41E-04
triflusal	chemical drug	-2.198	2.43E-07
ABCG1	transporter	-2.196	3.30E-06
pentobarbital	chemical drug	-2.192	4.31E-08
fingolimod	chemical drug	-2.19	4.88E-05
RCAN1	transcription regulator	-2.186	1.32E-06
APOE	transporter	-2.182	1.55E-07
NLRP12	other	-2.18	6.15E-06
SOCS1	other	-2.177	7.75E-12

luteolin	chemical - endogenous non-mammalian	-2.176	1.59E-07
thalidomide	chemical drug	-2.17	1.28E-07
H-7	chemical - kinase inhibitor	-2.169	5.17E-07
silibinin	chemical drug	-2.158	1.04E-05
SCD	enzyme	-2.153	2.64E-07
NR3C1	ligand-dependent nuclear receptor	-2.142	5.73E-27
KN-62	chemical - kinase inhibitor	-2.138	3.90E-12
TSC2	other	-2.135	4.67E-08
lipoxin A4	chemical - endogenous mammalian	-2.126	6.15E-06
BCL6	transcription regulator	-2.126	1.01E-05
Ciap	group	-2.116	2.00E-08
THRB	ligand-dependent nuclear receptor	-2.111	1.34E-08
diphenyleneiodonium	chemical reagent	-2.092	2.98E-10
PRDM1	transcription regulator	-2.067	1.18E-08
pyridoxamine	chemical - endogenous mammalian	-2	4.59E-08
8-oxo-7-hydrodeoxyguanosine	chemical - endogenous mammalian	-2	6.27E-07
N,N-dimethylsphingosine	chemical reagent	-2	1.46E-06
cyclomaltodextrin	chemical drug	-2	2.10E-06
Sod	group	-2	3.96E-06
SIN3A	transcription regulator	-2	4.40E-06
ITCH	enzyme	-2	5.25E-06
RNF20	enzyme	-2	8.72E-06
L-tryptophan	chemical - endogenous mammalian	-2	1.10E-05
FASN	enzyme	-2	2.04E-05
kaempferol	chemical toxicant	-2	3.46E-05
FBXO32	enzyme	-2	3.74E-03
daidzein	chemical drug	-2	4.24E-03
INSIG1	other	-2	7.42E-03
miR-30c-5p (and other miRNAs w/seed GUAAACA)	mature microrna	-2	9.33E-03

PKD1	ion channel	-2	4.19E-02

S4Table7. Networks and associated network functions associated with perfusion driven IGF

ID	Score	Focus Molecules	Top Diseases and Functions
1	27	17	Cell Death and Survival, Carbohydrate Metabolism, Molecular Transport
2	27	17	Humoral Immune Response, Protein Synthesis, Neurological Disease
3	25	16	Neurological Disease, Post-Translational Modification, Cellular Compromise
4	25	16	Gastrointestinal Disease, Inflammatory Disease, Inflammatory Response
5	21	14	Cell Morphology, Nervous System Development and Function, Neurological Disease
6	19	13	Infectious Diseases, Organismal Development, Cardiovascular Disease
7	19	13	Gene Expression, Cell Cycle, Connective Tissue Development and Function
8	15	11	Hereditary Disorder, Neurological Disease, Organismal Injury and Abnormalities
9	13	10	Organismal Injury and Abnormalities, Gene Expression, Cell Cycle
10	12	9	Connective Tissue Disorders, Immunological Disease, Inflammatory Disease
11	12	9	Cell-mediated Immune Response, Cellular Development, Cellular Function and Maintenance
12	12	9	Neurological Disease, Small Molecule Biochemistry, Cellular Development
13	10	8	DNA Replication, Recombination, and Repair, Neurological Disease, Cell-mediated Immune Response
14	10	8	Cellular Compromise, Cellular Growth and Proliferation, Organismal Development
15	7	6	Cellular Movement, Hematological System Development and Function, Immune Cell Trafficking
16	2	1	Cell Death and Survival, Cellular Movement, Nervous System Development and Function
17	2	1	Cellular Assembly and Organization, Cellular Function and Maintenance, Cancer
18	2	1	
19	2	1	Organismal Injury and Abnormalities, Reproductive System Disease

S4Table8. The list of the canonical pathways associated with associated with perfusion driven DGF

Canonical Pathways	-log(p-value)	Ratio	z-score
Granulocyte Adhesion and Diapedesis	1.29E+01	1.12E-01	NaN
Agranulocyte Adhesion and Diapedesis	1.14E+01	1.00E-01	NaN
Differential Regulation of Cytokine Production in Intestinal Epithelial Cells by IL-17A and IL-17F	1.13E+01	3.91E-01	NaN
Differential Regulation of Cytokine Production in Macrophages and T Helper Cells by IL-17A and IL-17F	8.87E+00	3.89E-01	NaN
HMGB1 Signaling	8.38E+00	1.08E-01	3.464
Glucocorticoid Receptor Signaling	6.92E+00	6.05E-02	NaN
Role of Cytokines in Mediating Communication between Immune Cells	6.43E+00	1.48E-01	NaN
Role of Hypercytokinemia/hyperchemokinemia in the Pathogenesis of Influenza	5.98E+00	1.63E-01	NaN
TREM1 Signaling	5.28E+00	1.05E-01	2.828
Communication between Innate and Adaptive Immune Cells	4.73E+00	8.89E-02	NaN
Hepatic Fibrosis / Hepatic Stellate Cell Activation	4.56E+00	5.88E-02	NaN
Atherosclerosis Signaling	4.51E+00	7.20E-02	NaN
Unfolded protein response	4.22E+00	1.11E-01	NaN
Cholecystokinin/Gastrin-mediated Signaling	4.22E+00	7.55E-02	2.828
IL-6 Signaling	3.94E+00	6.90E-02	1.414
PPAR Signaling	3.75E+00	7.53E-02	-1.89
IL-17A Signaling in Gastric Cells	3.59E+00	1.60E-01	NaN
IL-10 Signaling	3.53E+00	8.33E-02	NaN
NRF2-mediated Oxidative Stress Response	3.31E+00	5.00E-02	0.816
Aryl Hydrocarbon Receptor Signaling	3.26E+00	5.48E-02	NaN
IL-17A Signaling in Fibroblasts	3.02E+00	1.14E-01	NaN
Retinoic acid Mediated Apoptosis Signaling	2.89E+00	7.81E-02	2.236
Acute Phase Response Signaling	2.82E+00	4.68E-02	1.414
p53 Signaling	2.82E+00	6.12E-02	NaN

GADD45 Signaling	2.78E+00	1.58E-01	NaN
JAK/Stat Signaling	2.66E+00	6.94E-02	-<br>0.447
Role of IL-17F in Allergic Inflammatory Airway Diseases	2.64E+00	9.09E-02	2
Toll-like Receptor Signaling	2.61E+00	6.76E-02	NaN
Production of Nitric Oxide and Reactive Oxygen Species in Macrophages	2.59E+00	4.30E-02	2.828
Sertoli Cell-Sertoli Cell Junction Signaling	2.59E+00	4.30E-02	NaN
Chemokine Signaling	2.59E+00	6.67E-02	2.236
ILK Signaling	2.57E+00	4.28E-02	2.828
Prolactin Signaling	2.56E+00	6.58E-02	0.447
IL-8 Signaling	2.56E+00	4.26E-02	2.828
Type I Diabetes Mellitus Signaling	2.54E+00	5.41E-02	0.816
iNOS Signaling	2.54E+00	8.51E-02	NaN
VDR/RXR Activation	2.49E+00	6.33E-02	NaN
Graft-versus-Host Disease Signaling	2.47E+00	8.16E-02	NaN
Airway Pathology in Chronic Obstructive Pulmonary Disease	2.37E+00	2.50E-01	NaN
CXCR4 Signaling	2.37E+00	4.37E-02	2.449
Semaphorin Signaling in Neurons	2.35E+00	7.55E-02	NaN
Aldosterone Signaling in Epithelial Cells	2.34E+00	4.32E-02	NaN
ErbB Signaling	2.33E+00	5.81E-02	NaN
Altered T Cell and B Cell Signaling in Rheumatoid Arthritis	2.29E+00	5.68E-02	NaN
Role of Macrophages, Fibroblasts and Endothelial Cells in Rheumatoid Arthritis	2.28E+00	3.31E-02	NaN
Pathogenesis of Multiple Sclerosis	2.27E+00	2.22E-01	NaN
Tight Junction Signaling	2.27E+00	4.19E-02	NaN
TNFR2 Signaling	2.24E+00	1.03E-01	NaN
ATM Signaling	2.18E+00	6.78E-02	NaN
Dendritic Cell Maturation	2.12E+00	3.93E-02	2.646
IL-12 Signaling and Production in Macrophages	2.10E+00	4.38E-02	NaN
PCP pathway	2.08E+00	6.35E-02	1
IGF-1 Signaling	2.07E+00	5.05E-02	0

Estrogen-Dependent Breast Cancer Signaling	1.99E+00	5.97E-02	NaN
Interferon Signaling	1.98E+00	8.33E-02	NaN
Role of IL-17A in Psoriasis	1.95E+00	1.54E-01	NaN
Colorectal Cancer Metastasis Signaling	1.93E+00	3.32E-02	2.828
Erythropoietin Signaling	1.93E+00	5.71E-02	NaN
Role of PKR in Interferon Induction and Antiviral Response	1.86E+00	7.50E-02	NaN
CCR5 Signaling in Macrophages	1.84E+00	5.41E-02	NaN
Leukocyte Extravasation Signaling	1.82E+00	3.43E-02	2
p38 MAPK Signaling	1.78E+00	4.27E-02	1.342
Cyclins and Cell Cycle Regulation	1.77E+00	5.13E-02	-2
Germ Cell-Sertoli Cell Junction Signaling	1.75E+00	3.68E-02	NaN
Systemic Lupus Erythematosus Signaling	1.68E+00	3.21E-02	NaN
MSP-RON Signaling Pathway	1.67E+00	6.38E-02	NaN
LXR/RXR Activation	1.63E+00	3.91E-02	-<br>1.342
TNFR1 Signaling	1.62E+00	6.12E-02	NaN
Cell Cycle: G2/M DNA Damage Checkpoint Regulation	1.62E+00	6.12E-02	NaN
Hepatic Cholestasis	1.61E+00	3.41E-02	NaN
Neuregulin Signaling	1.52E+00	4.30E-02	1
IL-2 Signaling	1.49E+00	5.45E-02	NaN
Role of IL-17A in Arthritis	1.47E+00	5.36E-02	NaN
Actin Nucleation by ARP-WASP Complex	1.47E+00	5.36E-02	NaN
Role of JAK1, JAK2 and TYK2 in Interferon Signaling	1.44E+00	8.33E-02	NaN
Glioma Invasiveness Signaling	1.43E+00	5.17E-02	NaN
Role of JAK family kinases in IL-6-type Cytokine Signaling	1.40E+00	8.00E-02	NaN
Protein Ubiquitination Pathway	1.33E+00	2.70E-02	NaN
HGF Signaling	1.32E+00	3.70E-02	NaN
CD40 Signaling	1.31E+00	4.62E-02	NaN
phagosome formation	1.31E+00	3.67E-02	NaN
Tec Kinase Signaling	1.28E+00	3.12E-02	2

Role of Tissue Factor in Cancer	1.27E+00	3.57E-02	NaN
4-1BB Signaling in T Lymphocytes	1.24E+00	6.45E-02	NaN
PXR/RXR Activation	1.22E+00	4.23E-02	NaN
Airway Inflammation in Asthma	1.21E+00	2.00E-01	NaN
Renin-Angiotensin Signaling	1.20E+00	3.39E-02	2
IL-17 Signaling	1.20E+00	4.17E-02	NaN
Role of MAPK Signaling in the Pathogenesis of Influenza	1.20E+00	4.17E-02	NaN
14-3-3-mediated Signaling	1.19E+00	3.36E-02	NaN
Growth Hormone Signaling	1.19E+00	4.11E-02	NaN
Role of Osteoblasts, Osteoclasts and Chondrocytes in Rheumatoid Arthritis	1.18E+00	2.67E-02	NaN
STAT3 Pathway	1.17E+00	4.05E-02	NaN
Corticotropin Releasing Hormone Signaling	1.17E+00	3.31E-02	NaN
IL-9 Signaling	1.16E+00	5.88E-02	NaN
Role of JAK2 in Hormone-like Cytokine Signaling	1.16E+00	5.88E-02	NaN
Role of Pattern Recognition Receptors in Recognition of Bacteria and Viruses	1.13E+00	3.20E-02	NaN
Signaling by Rho Family GTPases	1.10E+00	2.54E-02	2.449
April Mediated Signaling	1.08E+00	5.26E-02	NaN
Hereditary Breast Cancer Signaling	1.07E+00	3.05E-02	NaN
Ceramide Signaling	1.04E+00	3.57E-02	NaN
B Cell Activating Factor Signaling	1.04E+00	5.00E-02	NaN
FXR/RXR Activation	1.02E+00	2.92E-02	NaN
Type II Diabetes Mellitus Signaling	1.02E+00	2.92E-02	NaN
TGF-Î² Signaling	1.01E+00	3.45E-02	NaN
UVC-Induced MAPK Signaling	1.01E+00	4.76E-02	NaN
Molecular Mechanisms of Cancer	9.93E-01	2.14E-02	NaN
UVA-Induced MAPK Signaling	9.88E-01	3.37E-02	NaN
Apoptosis Signaling	9.88E-01	3.37E-02	NaN
Crosstalk between Dendritic Cells and Natural Killer Cells	9.77E-01	3.33E-02	NaN
MIF Regulation of Innate Immunity	9.73E-01	4.55E-02	NaN
Death Receptor Signaling	9.56E-01	3.26E-02	NaN

Regulation of Actin-based Motility by Rho	9.45E-01	3.23E-02	NaN
IL-1 Signaling	9.35E-01	3.19E-02	NaN
Hematopoiesis from Pluripotent Stem Cells	9.25E-01	4.26E-02	NaN
Estrogen Biosynthesis	9.25E-01	4.26E-02	NaN
Glioblastoma Multiforme Signaling	9.05E-01	2.65E-02	1
GÎ±q Signaling	8.75E-01	2.58E-02	1
CD27 Signaling in Lymphocytes	8.54E-01	3.85E-02	NaN
Hematopoiesis from Multipotent Stem Cells	8.49E-01	8.33E-02	NaN
UVB-Induced MAPK Signaling	8.41E-01	3.77E-02	NaN
HIF1Î± Signaling	8.40E-01	2.88E-02	NaN
Tetrapyrrole Biosynthesis II	8.17E-01	7.69E-02	NaN
Pancreatic Adenocarcinoma Signaling	8.06E-01	2.78E-02	NaN
LPS/IL-1 Mediated Inhibition of RXR Function	8.00E-01	2.21E-02	2
Calcium Transport I	7.88E-01	7.14E-02	NaN
Thrombopoietin Signaling	7.67E-01	3.39E-02	NaN
Sphingosine-1-phosphate Signaling	7.65E-01	2.65E-02	NaN
Induction of Apoptosis by HIV1	7.56E-01	3.33E-02	NaN
EGF Signaling	7.56E-01	3.33E-02	NaN
Huntington's Disease Signaling	7.52E-01	2.13E-02	NaN
Activation of IRF by Cytosolic Pattern Recognition Receptors	7.34E-01	3.23E-02	NaN
Role of JAK1 and JAK3 in Î³c Cytokine Signaling	7.34E-01	3.23E-02	NaN
ERK5 Signaling	7.13E-01	3.12E-02	NaN
Cell Cycle: G1/S Checkpoint Regulation	7.13E-01	3.12E-02	NaN
Hypoxia Signaling in the Cardiovascular System	7.03E-01	3.08E-02	NaN
Mitotic Roles of Polo-Like Kinase	6.93E-01	3.03E-02	NaN
IL-17A Signaling in Airway Cells	6.93E-01	3.03E-02	NaN
Remodeling of Epithelial Adherens Junctions	6.74E-01	2.94E-02	NaN
phagosome maturation	6.64E-01	2.36E-02	NaN
PI3K/AKT Signaling	6.58E-01	2.34E-02	NaN
Agrin Interactions at Neuromuscular Junction	6.55E-01	2.86E-02	NaN

IL-3 Signaling	6.46E-01	2.82E-02	NaN
GDNF Family Ligand-Receptor Interactions	6.46E-01	2.82E-02	NaN
mTOR Signaling	6.43E-01	2.07E-02	NaN
T Helper Cell Differentiation	6.37E-01	2.78E-02	NaN
Neurotrophin/TRK Signaling	6.37E-01	2.78E-02	NaN
PI3K Signaling in B Lymphocytes	6.32E-01	2.27E-02	NaN
Endoplasmic Reticulum Stress Pathway	6.30E-01	4.76E-02	NaN
LPS-stimulated MAPK Signaling	6.29E-01	2.74E-02	NaN
Renal Cell Carcinoma Signaling	6.29E-01	2.74E-02	NaN
RAR Activation	6.23E-01	2.03E-02	NaN
GNRH Signaling	6.14E-01	2.22E-02	NaN
D-myo-inositol (1,4,5,6)-Tetrakisphosphate Biosynthesis	6.14E-01	2.22E-02	NaN
D-myo-inositol (3,4,5,6)-tetrakisphosphate Biosynthesis	6.14E-01	2.22E-02	NaN
Adipogenesis pathway	6.14E-01	2.22E-02	NaN
Zymosterol Biosynthesis	6.13E-01	4.55E-02	NaN
HER-2 Signaling in Breast Cancer	6.03E-01	2.63E-02	NaN
Polyamine Regulation in Colon Cancer	5.96E-01	4.35E-02	NaN
Role of Lipids/Lipid Rafts in the Pathogenesis of Influenza	5.96E-01	4.35E-02	NaN
Heme Biosynthesis II	5.96E-01	4.35E-02	NaN
Fatty Acid Î±-oxidation	5.96E-01	4.35E-02	NaN
Role of BRCA1 in DNA Damage Response	5.87E-01	2.56E-02	NaN
IL-22 Signaling	5.80E-01	4.17E-02	NaN
Tumoricidal Function of Hepatic Natural Killer Cells	5.80E-01	4.17E-02	NaN
Estrogen-mediated S-phase Entry	5.80E-01	4.17E-02	NaN
Integrin Signaling	5.71E-01	1.92E-02	2
Regulation of IL-2 Expression in Activated and Anergic T Lymphocytes	5.64E-01	2.47E-02	NaN
PDGF Signaling	5.50E-01	2.41E-02	NaN
Epithelial Adherens Junction Signaling	5.41E-01	2.03E-02	NaN
IL-15 Production	5.37E-01	3.70E-02	NaN
Allograft Rejection Signaling	5.36E-01	2.35E-02	NaN

Bladder Cancer Signaling	5.22E-01	2.30E-02	NaN
D-myo-inositol-5-phosphate Metabolism	5.21E-01	1.97E-02	NaN
3-phosphoinositide Degradation	5.16E-01	1.96E-02	NaN
RANK Signaling in Osteoclasts	5.09E-01	2.25E-02	NaN
eNOS Signaling	5.07E-01	1.94E-02	NaN
Cardiac Hypertrophy Signaling	4.73E-01	1.72E-02	2
SAPK/JNK Signaling	4.71E-01	2.11E-02	NaN
Telomerase Signaling	4.48E-01	2.02E-02	NaN
Circadian Rhythm Signaling	4.45E-01	2.86E-02	NaN
Coagulation System	4.45E-01	2.86E-02	NaN
Cell Cycle Regulation by BTG Family Proteins	4.45E-01	2.86E-02	NaN
3-phosphoinositide Biosynthesis	4.44E-01	1.78E-02	NaN
T Cell Receptor Signaling	4.32E-01	1.96E-02	NaN
NF-ÎºB Signaling	4.32E-01	1.74E-02	NaN
CDK5 Signaling	4.27E-01	1.94E-02	NaN
Antigen Presentation Pathway	4.26E-01	2.70E-02	NaN
Complement System	4.17E-01	2.63E-02	NaN
Role of NFAT in Regulation of the Immune Response	4.12E-01	1.69E-02	NaN
Glioma Signaling	4.12E-01	1.89E-02	NaN
Inhibition of Angiogenesis by TSP1	4.08E-01	2.56E-02	NaN
Cholesterol Biosynthesis I	4.08E-01	2.56E-02	NaN
Cholesterol Biosynthesis II (via 24,25-dihydrolanosterol)	4.08E-01	2.56E-02	NaN
Cholesterol Biosynthesis III (via Desmosterol)	4.08E-01	2.56E-02	NaN
RhoGDI Signaling	4.04E-01	1.68E-02	NaN
Transcriptional Regulatory Network in Embryonic Stem Cells	3.99E-01	2.50E-02	NaN
Inhibition of Matrix Metalloproteases	3.99E-01	2.50E-02	NaN
Salvage Pathways of Pyrimidine Ribonucleotides	3.97E-01	1.83E-02	NaN
PPARÎ±/RXRÎ± Activation	3.93E-01	1.65E-02	NaN
Thyroid Cancer Signaling	3.91E-01	2.44E-02	NaN
Regulation of the Epithelial-Mesenchymal Transition Pathway	3.86E-01	1.63E-02	NaN

Endothelin-1 Signaling	3.76E-01	1.60E-02	NaN
Androgen Signaling	3.74E-01	1.75E-02	NaN
G-Protein Coupled Receptor Signaling	3.69E-01	1.52E-02	NaN
Melanoma Signaling	3.68E-01	2.27E-02	NaN
ERK/MAPK Signaling	3.62E-01	1.57E-02	NaN
Docosahexaenoic Acid (DHA) Signaling	3.61E-01	2.22E-02	NaN
Role of Oct4 in Mammalian Embryonic Stem Cell Pluripotency	3.54E-01	2.17E-02	NaN
Neuroprotective Role of THOP1 in Alzheimer's Disease	3.54E-01	2.17E-02	NaN
Thrombin Signaling	3.46E-01	1.53E-02	NaN
PKCÎ¸ Signaling in T Lymphocytes	3.40E-01	1.64E-02	NaN
Xenobiotic Metabolism Signaling	3.36E-01	1.45E-02	NaN
CD28 Signaling in T Helper Cells	3.36E-01	1.63E-02	NaN
Axonal Guidance Signaling	3.17E-01	1.36E-02	NaN
Estrogen Receptor Signaling	3.17E-01	1.56E-02	NaN
P2Y Purigenic Receptor Signaling Pathway	3.13E-01	1.55E-02	NaN
Role of CHK Proteins in Cell Cycle Checkpoint Control	2.98E-01	1.82E-02	NaN
Lymphotoxin Î² Receptor Signaling	2.93E-01	1.79E-02	NaN
Superpathway of Inositol Phosphate Compounds	2.88E-01	1.39E-02	NaN
ErbB2-ErbB3 Signaling	2.88E-01	1.75E-02	NaN
Nur77 Signaling in T Lymphocytes	2.82E-01	1.72E-02	NaN
Regulation of Cellular Mechanics by Calpain Protease	2.82E-01	1.72E-02	NaN
cAMP-mediated signaling	2.71E-01	1.35E-02	NaN
Heparan Sulfate Biosynthesis (Late Stages)	2.68E-01	1.64E-02	NaN
Relaxin Signaling	2.61E-01	1.38E-02	NaN
IL-15 Signaling	2.54E-01	1.56E-02	NaN
Glutamate Receptor Signaling	2.54E-01	1.56E-02	NaN
Role of PI3K/AKT Signaling in the Pathogenesis of Influenza	2.50E-01	1.54E-02	NaN
Antiproliferative Role of Somatostatin Receptor 2	2.41E-01	1.49E-02	NaN
Calcium-induced T Lymphocyte Apoptosis	2.37E-01	1.47E-02	NaN
Heparan Sulfate Biosynthesis	2.22E-01	1.39E-02	NaN

Caveolar-mediated Endocytosis Signaling	2.18E-01	1.37E-02	NaN
Small Cell Lung Cancer Signaling	2.15E-01	1.35E-02	NaN
Pyridoxal 5'-phosphate Salvage Pathway	2.15E-01	1.35E-02	NaN
Ephrin B Signaling	2.11E-01	1.33E-02	NaN
Role of Wnt/GSK-3Î² Signaling in the Pathogenesis of Influenza	2.08E-01	1.32E-02	NaN
IL-4 Signaling	2.04E-01	1.30E-02	NaN
BMP signaling pathway	2.04E-01	1.30E-02	NaN
Leptin Signaling in Obesity	2.01E-01	1.28E-02	NaN
GPCR-Mediated Integration of Enteroendocrine Signaling Exemplified by an L Cell	1.98E-01	1.27E-02	NaN

S4Table9. The complete list of activated and inhibited upstream regulators associated with perfusion driven DGF

Upstream Regulator	Molecule Type	Activation z-score	p-value of overlap
**Activated**			
lipopolysaccharide	chemical drug	7.73	1.92E-31
TNF	cytokine	7.287	1.57E-35
IL1B	cytokine	6.724	6.07E-37
PDGF BB	complex	6.222	1.27E-32
phorbol myristate acetate	chemical drug	6.109	1.87E-20
NFkB (complex)	complex	6.083	3.97E-25
poly rI:rC-RNA	biologic drug	5.885	2.16E-24
IFNG	cytokine	5.859	3.03E-21
IL2	cytokine	5.484	1.09E-21
Salmonella enterica serotype abortus equi lipopolysaccharide	chemical toxicant	5.445	1.67E-26
P38 MAPK	group	5.361	2.72E-25
IL6	cytokine	5.233	6.18E-19

ERK	group	5.13	3.68E-26
F2	peptidase	5.125	2.99E-20
CREB1	transcription regulator	5.099	2.68E-28
CSF2	cytokine	4.997	1.89E-22
hydrogen peroxide	chemical - endogenous mammalian	4.997	6.21E-19
IL1	group	4.891	2.81E-13
MYD88	other	4.876	9.54E-21
TNFSF11	cytokine	4.79	3.65E-18
leukotriene D4	chemical - endogenous mammalian	4.787	4.02E-32
salmonella minnesota R595 lipopolysaccharides	chemical - endogenous non-mammalian	4.786	4.68E-24
TLR3	transmembrane receptor	4.772	1.41E-18
RELA	transcription regulator	4.738	2.68E-19
E. coli B5 lipopolysaccharide	chemical - endogenous non-mammalian	4.736	6.71E-18
tretinoin	chemical - endogenous mammalian	4.66	1.29E-08
5-O-mycolyl-beta-araf-(1->2)-5-O-mycolyl-alpha-araf-(1->1')-glycerol	chemical - endogenous non-mammalian	4.656	1.22E-23
cisplatin	chemical drug	4.622	2.09E-16
ERK1/2	group	4.619	4.18E-19
thapsigargin	chemical toxicant	4.589	9.40E-23
EGF	growth factor	4.586	3.04E-21
kainic acid	chemical toxicant	4.525	1.89E-18
Ca2+	chemical - endogenous mammalian	4.458	4.88E-19
TLR9	transmembrane receptor	4.451	8.76E-17
TGFB1	growth factor	4.377	5.42E-21
IL1A	cytokine	4.345	1.86E-12

cocaine	chemical drug	4.341	1.28E-16
TLR4	transmembrane receptor	4.315	1.60E-18
peptidoglycan	chemical - endogenous non-mammalian	4.314	5.37E-19
TICAM1	other	4.302	4.70E-20
AGT	growth factor	4.287	3.25E-12
IL17A	cytokine	4.248	1.68E-19
dalfampridine	chemical drug	4.243	5.07E-27
EGR1	transcription regulator	4.24	2.21E-21
Jnk	group	4.234	4.04E-21
IL3	cytokine	4.234	3.96E-15
doxorubicin	chemical drug	4.232	2.47E-16
TLR7	transmembrane receptor	4.228	2.68E-16
GnRH-A	chemical reagent	4.172	7.66E-24
bicuculline	chemical - endogenous non-mammalian	4.165	6.23E-25
E. coli B4 lipopolysaccharide	chemical toxicant	4.162	1.74E-11
CD40LG	cytokine	4.156	3.84E-18
gentamicin	chemical drug	4.133	1.92E-12
SMARCA4	transcription regulator	4.119	1.46E-11
IL5	cytokine	4.105	5.39E-15
camptothecin	chemical drug	4.094	3.23E-17
NUPR1	transcription regulator	4.082	9.80E-09
cigarette smoke	chemical toxicant	4.052	1.03E-13
OSM	cytokine	3.961	1.97E-13
FOXO1	transcription regulator	3.957	2.83E-12
IKBKB	kinase	3.944	3.95E-19
carbon tetrachloride	chemical toxicant	3.93	5.80E-14
STAT4	transcription regulator	3.922	8.86E-09
palmitic acid	chemical - endogenous	3.919	8.44E-12

	mammalian		
Ap1	complex	3.916	1.32E-14
RAF1	kinase	3.912	1.01E-12
IL18	cytokine	3.898	9.17E-12
IL4	cytokine	3.883	5.38E-15
FGF2	growth factor	3.84	1.09E-14
IGF1	growth factor	3.84	2.26E-12
TLR2	transmembrane receptor	3.828	2.95E-12
Vegf	group	3.816	4.44E-11
TP53	transcription regulator	3.81	7.37E-18
carrageenan	chemical - endogenous non-mammalian	3.786	2.74E-19
Tlr	group	3.766	3.96E-13
Cg	complex	3.762	7.53E-13
PTGS2	enzyme	3.706	5.45E-13
CXCL12	cytokine	3.678	8.64E-11
EDN1	cytokine	3.67	1.68E-10
E. coli lipopolysaccharide	chemical - endogenous non-mammalian	3.669	4.39E-14
MAP2K1/2	group	3.657	2.22E-14
APP	other	3.655	4.89E-11
SMAD3	transcription regulator	3.648	1.03E-19
resiquimod	chemical drug	3.646	4.53E-17
D-glucose	chemical - endogenous mammalian	3.637	5.51E-12
CD40	transmembrane receptor	3.627	7.94E-12
EPO	cytokine	3.614	5.01E-13
forskolin	chemical toxicant	3.576	6.72E-29
acetaminophen	chemical drug	3.541	3.30E-11
KITLG	growth factor	3.537	9.81E-08

A23187	chemical reagent	3.525	5.89E-10
bleomycin	chemical drug	3.51	8.01E-11
TCR	complex	3.493	6.50E-11
ionomycin	chemical reagent	3.491	1.32E-07
IL15	cytokine	3.487	3.53E-14
PTPRJ	phosphatase	3.464	2.92E-14
Pam3-Cys-Ser-Lys4	chemical reagent	3.413	7.88E-13
CTNNB1	transcription regulator	3.403	1.70E-04
CSF1	cytokine	3.401	1.59E-12
methyl methanesulfonate	chemical toxicant	3.396	1.85E-15
TREM1	transmembrane receptor	3.394	1.37E-18
anisomycin	chemical - endogenous non-mammalian	3.392	1.67E-14
IL7	cytokine	3.377	5.17E-13
trinitrobenzenesulfonic acid	chemical reagent	3.369	7.36E-10
trovafloxacin	chemical drug	3.357	2.25E-15
SAMSN1	other	3.357	2.17E-13
lysophosphatidic acid	chemical - other	3.353	5.57E-15
TAC1	other	3.351	2.80E-10
Interferon alpha	group	3.342	2.30E-07
Tnf (family)	group	3.316	1.63E-09
FOXL2	transcription regulator	3.311	2.93E-16
PAF1	other	3.302	6.39E-12
MET	kinase	3.302	4.92E-10
LEP	growth factor	3.284	2.10E-09
Nfat (family)	group	3.268	6.44E-10
C5	cytokine	3.258	1.58E-20
AGER	transmembrane receptor	3.257	2.19E-12
5-hydroxytryptamine	chemical - endogenous mammalian	3.255	5.31E-10

CpG ODN 1668	chemical reagent	3.239	1.70E-12
CCL5	cytokine	3.232	1.27E-13
trichostatin A	chemical drug	3.226	9.50E-12
enterotoxin B	biologic drug	3.212	4.20E-11
MAP2K1	kinase	3.211	2.38E-12
hemozoin	chemical - endogenous non-mammalian	3.204	5.90E-15
CHUK	kinase	3.203	5.26E-14
Pdgf (complex)	complex	3.191	6.82E-12
PI3K (complex)	complex	3.187	5.49E-17
TNFRSF1A	transmembrane receptor	3.181	4.26E-17
S100A9	other	3.181	7.72E-09
Ras	group	3.169	1.98E-11
HIF1A	transcription regulator	3.134	4.27E-13
IFI16	transcription regulator	3.131	1.34E-09
AKT1	kinase	3.13	2.55E-08
PDX1	transcription regulator	3.13	1.73E-07
IL12 (complex)	complex	3.118	1.96E-08
10E,12Z-octadecadienoic acid	chemical - endogenous mammalian	3.112	3.44E-09
PF4	cytokine	3.107	7.93E-14
NGF	growth factor	3.099	6.67E-11
okadaic acid	chemical toxicant	3.097	1.09E-13
NFATC2	transcription regulator	3.092	2.35E-10
TGFB3	growth factor	3.081	3.82E-07
EIF2AK2	kinase	3.078	1.96E-12
MAPK14	kinase	3.078	1.69E-11
mitomycin C	chemical drug	3.077	2.09E-09
CpG oligonucleotide	chemical drug	3.073	3.59E-09
IRF8	transcription regulator	3.071	4.80E-09

decitabine	chemical drug	3.07	2.20E-11
SRC	kinase	3.067	1.37E-06
HGF	growth factor	3.065	2.84E-19
L-glutamic acid	chemical - endogenous mammalian	3.045	2.37E-07
deferoxamine	chemical drug	3.036	1.04E-12
HMGB1	transcription regulator	3.013	1.13E-10
tunicamycin	chemical - endogenous non-mammalian	3.01	2.14E-10
GH1	growth factor	3.01	2.10E-07
lipoarabinomannan	chemical - endogenous non-mammalian	3	4.67E-14
SELPLG	other	3	1.22E-10
zymosan	chemical - endogenous non-mammalian	2.985	2.15E-12
BMP6	growth factor	2.985	1.16E-07
Mek	group	2.98	1.02E-09
MAPK3	kinase	2.975	2.05E-11
thioacetamide	chemical toxicant	2.975	4.24E-05
reactive oxygen species	chemical toxicant	2.97	1.22E-11
TNFSF12	cytokine	2.968	1.36E-06
IL17a dimer	complex	2.966	2.88E-12
PGR	ligand-dependent nuclear receptor	2.964	7.52E-07
MIF	cytokine	2.962	7.70E-12
CD14	transmembrane receptor	2.956	3.26E-11
TGM2	enzyme	2.949	8.69E-05
IFNA2	cytokine	2.947	9.45E-09
F7	peptidase	2.945	9.00E-11
ATF4	transcription regulator	2.943	8.40E-08
cyclic AMP	chemical - endogenous	2.94	3.55E-11

	mammalian		
JAK2	kinase	2.935	1.26E-05
TLR5	transmembrane receptor	2.934	3.79E-10
IL27	cytokine	2.931	2.12E-13
AGN194204	chemical drug	2.931	1.48E-04
CpG ODN 1826	chemical reagent	2.93	1.76E-09
FN1	enzyme	2.929	1.56E-07
F2R	g-protein coupled receptor	2.928	1.48E-09
IL6R	transmembrane receptor	2.926	4.99E-11
VEGFA	growth factor	2.926	1.21E-09
MAP3K8	kinase	2.923	2.35E-09
NOD2	other	2.922	6.79E-14
LCN2	transporter	2.921	4.12E-08
MALP-2s	chemical reagent	2.919	1.07E-11
platelet activating factor	chemical - endogenous mammalian	2.916	9.59E-09
LDL	complex	2.913	3.67E-21
ELK1	transcription regulator	2.909	3.82E-13
formaldehyde	chemical - endogenous mammalian	2.905	2.31E-11
D-galactosamine	chemical - endogenous mammalian	2.905	3.79E-11
uric acid	chemical - endogenous mammalian	2.904	3.72E-12
beta-estradiol	chemical - endogenous mammalian	2.887	6.23E-31
TBK1	kinase	2.876	1.49E-11
Ifn gamma	complex	2.851	1.24E-06
SRF	transcription regulator	2.847	1.49E-12
CREBBP	transcription regulator	2.835	3.70E-11

MAPK8	kinase	2.832	3.79E-10
IFNB1	cytokine	2.83	2.48E-08
NEDD9	other	2.828	8.02E-09
DOCK8	other	2.828	1.58E-06
SASH1	other	2.828	1.97E-06
leukotriene C4	chemical - endogenous mammalian	2.821	2.12E-12
EIF2AK3	kinase	2.816	2.55E-06
TRADD	other	2.813	8.94E-10
oblimersen	biologic drug	2.813	2.29E-07
di(2-ethylhexyl) phthalate	chemical toxicant	2.804	3.06E-07
NRG1	other	2.801	1.33E-14
C5AR1	g-protein coupled receptor	2.791	3.57E-08
Ccl2	cytokine	2.791	2.65E-07
PRKCA	kinase	2.787	3.25E-10
AIMP1	cytokine	2.784	2.12E-12
bromodeoxyuridine	chemical drug	2.781	1.97E-07
carbamylcholine	chemical drug	2.777	1.27E-08
imiquimod	chemical drug	2.775	2.37E-09
IL32	cytokine	2.774	9.59E-09
HSPD1	enzyme	2.772	2.20E-10
EP300	transcription regulator	2.77	2.81E-08
N-acetylmuramyl-L-alanyl-D-isoglutamine	chemical - endogenous non-mammalian	2.766	1.36E-08
Mapk	group	2.76	2.26E-09
TRAF6	enzyme	2.76	1.97E-07
Gm-csf	group	2.759	9.59E-09
dinoprost	chemical - endogenous mammalian	2.759	3.31E-06

SP1	transcription regulator	2.745	3.09E-13
etoposide	chemical drug	2.74	5.57E-09
E. coli serotype 0127B8 lipopolysaccharide	chemical - endogenous non-mammalian	2.74	4.91E-08
cyclophosphamide	chemical drug	2.739	2.70E-06
PGF	growth factor	2.736	2.12E-12
ozone	chemical toxicant	2.735	4.59E-10
phorbol esters	chemical - other	2.729	1.16E-07
CREM	transcription regulator	2.728	1.66E-20
hyaluronic acid	chemical - endogenous mammalian	2.726	3.14E-11
CXCR4	g-protein coupled receptor	2.726	1.89E-08
S100A8	other	2.72	2.39E-10
IL21	cytokine	2.715	6.99E-06
STAT3	transcription regulator	2.714	1.08E-17
NFKB1	transcription regulator	2.712	1.51E-15
methylprednisolone	chemical drug	2.703	2.84E-08
NFKBIA	transcription regulator	2.7	6.05E-14
FOXO3	transcription regulator	2.691	2.25E-11
bucladesine	chemical toxicant	2.677	5.88E-18
KRT17	other	2.646	4.92E-08
gentamicin C	chemical drug	2.646	9.58E-06
MKNK1	kinase	2.646	2.03E-04
CAMP	other	2.642	1.00E-12
NfkB-RelA	complex	2.638	6.36E-09
ATP	chemical - endogenous mammalian	2.63	1.97E-10
F3	transmembrane receptor	2.63	8.60E-06
ETS2	transcription regulator	2.63	8.60E-06

triamterene	chemical drug	2.63	1.31E-05
IGF1R	transmembrane receptor	2.63	1.86E-04
homocysteine	chemical - endogenous mammalian	2.627	5.04E-07
PRKCE	kinase	2.625	9.54E-08
IL-17f dimer	complex	2.621	5.37E-10
C5	other	2.621	5.37E-10
MTPN	transcription regulator	2.621	5.43E-05
ICAM1	transmembrane receptor	2.619	3.79E-11
fenamic acid	chemical reagent	2.619	1.76E-05
CCL3	cytokine	2.613	3.21E-10
3M-001	chemical drug	2.613	2.32E-07
ERBB2	kinase	2.608	7.57E-10
IL17F	cytokine	2.605	2.92E-08
TNFRSF8	transmembrane receptor	2.6	7.71E-06
stallimycin	biologic drug	2.598	1.10E-06
tamoxifen	chemical drug	2.595	2.01E-06
norepinephrine	chemical - endogenous mammalian	2.587	1.16E-10
GLI1	transcription regulator	2.586	8.84E-03
PRKCD	kinase	2.583	9.14E-12
CAMK4	kinase	2.583	6.29E-08
GNRH1	other	2.583	4.86E-06
ETS1	transcription regulator	2.582	3.30E-08
aldosterone	chemical - endogenous mammalian	2.582	6.73E-04
F2RL1	g-protein coupled receptor	2.581	2.65E-07
silicon dioxide	chemical drug	2.577	3.19E-10
fatty acid	chemical - endogenous mammalian	2.575	1.39E-05

Fibrinogen	complex	2.574	6.29E-11
MAPK7	kinase	2.573	3.41E-12
methylnitronitrosoguanidine	chemical toxicant	2.571	3.81E-08
quinolinic acid	chemical - endogenous mammalian	2.565	4.32E-08
Pam3-Cys	chemical toxicant	2.561	2.81E-07
JUN	transcription regulator	2.553	3.06E-15
Lh	complex	2.552	2.93E-07
tributyrin	chemical drug	2.55	4.72E-09
STAT5A	transcription regulator	2.547	4.47E-08
LIF	cytokine	2.543	2.54E-12
5-azacytidine	chemical drug	2.538	6.92E-06
nitrofurantoin	chemical drug	2.538	3.06E-05
Map3k7	kinase	2.535	3.35E-12
TGFA	growth factor	2.535	3.11E-09
PLG	peptidase	2.533	2.55E-05
NFYA	transcription regulator	2.53	1.49E-11
PIK3R1	kinase	2.53	1.14E-08
ANGPT2	growth factor	2.53	1.70E-05
Pka	complex	2.518	2.30E-15
RETN	other	2.499	4.93E-11
Fcer1	complex	2.497	5.15E-12
RET	kinase	2.482	3.43E-13
C3	peptidase	2.463	4.64E-07
paclitaxel	chemical drug	2.458	9.18E-19
25-hydroxycholesterol	chemical reagent	2.455	5.02E-16
BCR (complex)	complex	2.449	1.82E-07
ARHGAP21	other	2.449	1.11E-05
SPIB	transcription regulator	2.449	4.93E-04
cardiotoxin	chemical - other	2.449	9.38E-03

mono-(2-ethylhexyl)phthalate	chemical toxicant	2.449	1.37E-02
calcitriol	chemical drug	2.447	1.23E-09
Ni2+	chemical reagent	2.438	2.96E-07
crocidolite asbestos	chemical toxicant	2.436	7.96E-07
sulindac sulfide	chemical drug	2.435	2.12E-05
cuprizone	chemical toxicant	2.433	4.33E-08
phenacetin	chemical drug	2.433	9.64E-06
9,10-dimethyl-1,2-benzanthracene	chemical toxicant	2.433	9.64E-06
ALB	transporter	2.433	1.27E-05
topotecan	chemical drug	2.428	5.49E-09
EPHB1	kinase	2.425	5.31E-10
SP3	transcription regulator	2.425	3.68E-09
2-bromoethylamine	chemical reagent	2.425	2.40E-05
cis-urocanic acid	chemical drug	2.423	1.67E-07
BMP2	growth factor	2.423	4.98E-04
CXCL3	cytokine	2.418	1.91E-08
lysophosphatidylcholine	chemical - other	2.415	2.90E-11
CHRM1	g-protein coupled receptor	2.415	2.29E-09
ATF2	transcription regulator	2.415	4.06E-09
PPRC1	transcription regulator	2.414	1.45E-05
CCND1	transcription regulator	2.414	4.77E-03
PIK3CG	kinase	2.413	1.11E-05
PRKCB	kinase	2.412	4.31E-06
cobalt chloride	chemical reagent	2.408	1.97E-04
CRH	cytokine	2.406	7.71E-06
potassium chloride	chemical drug	2.405	2.80E-10
haloperidol	chemical drug	2.405	5.25E-04
MAP2K4	kinase	2.404	5.19E-05
colchicine	chemical drug	2.401	2.59E-06

HOXA5	transcription regulator	2.4	8.87E-08
seocalcitol	chemical drug	2.4	7.00E-05
GATA1	transcription regulator	2.4	7.69E-04
GATA4	transcription regulator	2.4	1.19E-02
sphingosine-1-phosphate	chemical - endogenous mammalian	2.399	5.97E-08
JAK1	kinase	2.396	8.07E-10
Akt	group	2.392	5.02E-12
Il3	cytokine	2.391	1.60E-05
CD244	transmembrane receptor	2.39	7.22E-11
TLR8	transmembrane receptor	2.387	1.27E-05
STAT	group	2.382	1.90E-07
BDNF	growth factor	2.381	7.37E-11
NR3C2	ligand-dependent nuclear receptor	2.378	4.58E-07
IL12 (family)	group	2.377	5.95E-04
lipoteichoic acid	chemical - endogenous non-mammalian	2.374	6.34E-08
RNASE2	enzyme	2.372	3.80E-06
CLEC7A	transmembrane receptor	2.368	2.96E-07
N-methyl-D-aspartate	chemical reagent	2.367	1.65E-05
SYK	kinase	2.359	3.79E-10
L-dopa	chemical - endogenous mammalian	2.348	2.87E-03
chenodeoxycholic acid	chemical - endogenous mammalian	2.346	4.33E-04
IL33	cytokine	2.345	4.52E-07
PTH	other	2.327	4.04E-11
SREBF1	transcription regulator	2.31	2.59E-03
AR	ligand-dependent nuclear receptor	2.306	1.67E-04

REL	transcription regulator	2.298	3.15E-10
methamphetamine	chemical drug	2.279	1.31E-08
vinblastine	chemical drug	2.279	6.32E-07
1-methyl-4-phenyl-1,2,3,6-tetrahydropyridine	chemical toxicant	2.276	1.09E-07
MAPK1	kinase	2.27	3.22E-13
diethylstilbestrol	chemical drug	2.269	2.42E-04
Pkc(s)	group	2.265	7.73E-13
tert-butyl-hydroquinone	chemical reagent	2.247	4.45E-06
dextran sulfate	chemical drug	2.244	2.87E-06
ST3-Hel2A-2	chemical reagent	2.236	9.84E-08
KLK5	peptidase	2.236	1.71E-06
NTRK1	kinase	2.236	5.04E-06
H2AFB3 (includes others)	other	2.236	3.40E-04
hexachlorobenzene	chemical toxicant	2.236	5.71E-04
TBX5	transcription regulator	2.236	1.12E-03
OGG1	enzyme	2.233	1.34E-09
Notch	group	2.233	2.19E-06
GNRH	group	2.233	7.98E-06
ceruletide	biologic drug	2.229	5.04E-06
N-formyl-Met-Leu-Phe	chemical reagent	2.226	1.34E-04
IRF1	transcription regulator	2.226	2.47E-03
YAP1	transcription regulator	2.224	1.28E-03
clozapine	chemical drug	2.222	7.81E-04
8-hydroxyguanine	chemical - endogenous mammalian	2.219	1.34E-09
ACVR1C	kinase	2.219	1.71E-06
DPP-23	chemical reagent	2.219	2.29E-06
dimethylnitrosamine	chemical toxicant	2.219	1.45E-05
ELANE	peptidase	2.219	2.98E-05

D-fructose	chemical - endogenous mammalian	2.219	1.34E-04
phenylbutazone	chemical drug	2.219	7.24E-04
EIF4E	translation regulator	2.219	5.93E-03
thyroid hormone	chemical - endogenous mammalian	2.219	6.33E-03
pCPT-cAMP	chemical - kinase inhibitor	2.216	5.42E-08
TACR1	g-protein coupled receptor	2.216	1.24E-06
SRC (family)	group	2.216	1.27E-05
FOXO4	transcription regulator	2.215	1.11E-05
PSEN1	peptidase	2.214	3.01E-06
Raf	group	2.213	6.32E-07
CSF1R	kinase	2.213	6.32E-07
cyclopiazonic acid	chemical - endogenous non-mammalian	2.213	8.86E-07
Lymphotoxin	complex	2.213	2.29E-06
RORC	ligand-dependent nuclear receptor	2.213	8.71E-05
CEBPB	transcription regulator	2.211	3.04E-13
picryl chloride	chemical toxicant	2.211	4.14E-07
IL-1R	group	2.207	2.96E-07
fluoxetine	chemical drug	2.207	2.87E-04
2,4-dinitrofluorobenzene	chemical toxicant	2.203	1.24E-06
S-(2,3-bispalmitoyloxypropyl)-cysteine-GDPKHPKSF	chemical reagent	2.2	1.71E-06
TET1	other	2.2	5.04E-06
cytarabine	chemical drug	2.2	1.32E-04
N-acetylsphingosine	chemical reagent	2.2	1.68E-04
isotretinoin	biologic drug	2.2	1.87E-03

TLR6	transmembrane receptor	2.198	5.04E-06
PDGFB	growth factor	2.197	4.68E-05
CP-55940	chemical reagent	2.196	5.28E-06
NfkB1-RelA	complex	2.196	1.21E-05
4-methylnitrosoamino-1-(3-pyridinyl)-1-butanone	chemical toxicant	2.196	2.98E-05
AMH	growth factor	2.194	1.21E-05
CYR61	other	2.19	5.28E-06
IL17C	cytokine	2.19	2.12E-05
LAMA5	other	2.186	1.24E-06
1,4-bis[2-(3,5-dichloropyridyloxy)]benzene	chemical toxicant	2.186	7.39E-03
EPAS1	transcription regulator	2.183	4.76E-07
ssRNA40	chemical reagent	2.183	1.71E-06
CXCL10	cytokine	2.183	9.88E-06
TNFSF13B	cytokine	2.183	3.09E-04
amphetamine	chemical drug	2.182	8.43E-07
MAP2K5	kinase	2.182	3.93E-06
Ifn	group	2.178	1.48E-06
vancomycin	biologic drug	2.178	8.42E-06
NR1I3	ligand-dependent nuclear receptor	2.177	7.39E-03
DDX58	enzyme	2.174	4.84E-04
TRH	other	2.169	6.30E-05
MAP2K3	kinase	2.166	1.76E-05
LCK	kinase	2.16	2.52E-05
dimethyl sulfoxide	chemical drug	2.158	2.00E-05
IFN alpha/beta	group	2.156	2.49E-03
dopamine	chemical - endogenous mammalian	2.156	7.70E-03
cycloheximide	chemical reagent	2.14	5.67E-24

PTPRC	phosphatase	2.14	6.32E-07
CXCL2	cytokine	2.138	4.19E-12
CCL11	cytokine	2.138	9.59E-09
allopurinol	chemical drug	2.138	7.06E-06
PARP1	enzyme	2.128	1.59E-07
Ins1	other	2.118	5.80E-06
progesterone	chemical - endogenous mammalian	2.105	4.98E-10
NOTCH1	transcription regulator	2.089	2.87E-06
RIPK2	kinase	2.083	1.93E-05
NFKBIB	transcription regulator	2.078	1.69E-07
SPP1	cytokine	2.077	3.21E-09
KLF6	transcription regulator	2.068	1.41E-11
STAT1	transcription regulator	2.058	2.41E-12
STAT5B	transcription regulator	2.058	9.27E-08
PTGER2	g-protein coupled receptor	2.055	3.86E-06
DETA-NONOate	chemical reagent	2.048	5.52E-09
EGFR	kinase	2.038	4.92E-18
CD38	enzyme	2.027	2.65E-03
IL12B	cytokine	2.025	1.89E-08
SELP	transmembrane receptor	2.024	4.77E-12
prostaglandin E2	chemical - endogenous mammalian	2.023	5.23E-25
Growth hormone	group	2.022	3.53E-07
nitric oxide	chemical - endogenous mammalian	2.016	7.84E-10
KRAS	enzyme	2.003	2.11E-07
6,7-dinitroquinoxaline-2,3-dione	chemical reagent	2	1.34E-09
ANKRD42	transcription regulator	2	6.58E-07

U1 snRNP	complex	2	1.30E-06
TGFB2	growth factor	2	3.28E-06
Pdgfr	group	2	3.84E-06
tridecanoic acid	chemical - endogenous mammalian	2	3.84E-06
Ikb	group	2	5.04E-06
ALOX12	enzyme	2	5.97E-06
Pdgf Ab	complex	2	5.19E-05
CARM1	transcription regulator	2	6.30E-05
RNASE1	enzyme	2	9.86E-05
RNF31	enzyme	2	1.19E-04
nitroarginine	chemical reagent	2	3.16E-04
BRD2	kinase	2	3.40E-04
tosedostat	chemical drug	2	4.15E-04
carboplatin	chemical drug	2	2.90E-03
RETNLB	other	2	1.33E-02
ISL1	transcription regulator	2	2.00E-02
**Inhibited**			
prazosin	chemical drug	-2	2.69E-07
belnacasan	chemical drug	-2	6.58E-07
PPT1	enzyme	-2	1.30E-06
RNF20	enzyme	-2	1.71E-06
USP18	peptidase	-2	1.71E-06
ENTPD1	enzyme	-2	2.32E-06
kaempferol	chemical toxicant	-2	9.88E-06
ethylenediaminetetraacetic acid	chemical drug	-2	1.27E-05
ITCH	enzyme	-2	3.14E-05
fontolizumab	biologic drug	-2	4.07E-05
chelerythrine	chemical drug	-2	8.06E-05
NFIL3	transcription regulator	-2	1.43E-04

JAK inhibitor I	chemical - kinase inhibitor	-2	1.70E-04
bisindolylmaleimide	chemical - kinase inhibitor	-2	2.35E-04
CR1L	other	-2	5.35E-04
SIN3A	transcription regulator	-2	5.71E-04
andrographolide	chemical drug	-2	6.03E-04
paricalcitol	chemical drug	-2	7.57E-04
GPX1	enzyme	-2	1.66E-03
alefacept	biologic drug	-2	4.10E-03
miR-199a-5p (and other miRNAs w/seed CCAGUGU)	mature microrna	-2	9.60E-03
losartan potassium	chemical drug	-2.053	2.90E-04
BAPTA-AM	chemical reagent	-2.079	9.10E-16
MEOX2	transcription regulator	-2.1	6.32E-10
KLF2	transcription regulator	-2.116	1.68E-08
SOX2	transcription regulator	-2.121	1.04E-06
THRB	ligand-dependent nuclear receptor	-2.121	3.24E-04
rottlerin	chemical toxicant	-2.126	3.63E-07
alvocidib	chemical drug	-2.134	9.35E-05
carbon monoxide	chemical - endogenous mammalian	-2.138	3.05E-08
mifepristone	chemical drug	-2.147	3.19E-11
PP2/AG1879 tyrosine kinase inhibitor	chemical - kinase inhibitor	-2.156	2.70E-06
pentoxifylline	chemical drug	-2.156	6.30E-05
resolvin D1	chemical - endogenous mammalian	-2.157	2.38E-11
luteolin	chemical - endogenous non-mammalian	-2.166	4.21E-05

CD28	transmembrane receptor	-2.177	6.71E-10
miR-30c-5p (and other miRNAs w/seed GUAAACA)	mature microrna	-2.177	9.70E-03
SIGIRR	transmembrane receptor	-2.183	8.86E-07
triflusal	chemical drug	-2.183	2.29E-06
valsartan	chemical drug	-2.183	1.06E-04
triptolide	chemical drug	-2.184	4.64E-07
CGS 21680	chemical reagent	-2.186	6.30E-05
pentobarbital	chemical drug	-2.192	4.14E-07
H-7	chemical - kinase inhibitor	-2.194	7.20E-06
NR4A1	ligand-dependent nuclear receptor	-2.195	4.00E-03
SERCA	group	-2.2	1.34E-09
infliximab	biologic drug	-2.2	6.89E-06
eplerenone	chemical drug	-2.2	2.52E-05
thioctic acid	chemical drug	-2.2	4.68E-05
3-aminobenzamide	chemical toxicant	-2.207	4.14E-07
fasudil	chemical drug	-2.213	1.19E-04
ARRB2	other	-2.216	6.45E-10
JAG2	growth factor	-2.216	9.35E-05
candesartan	chemical drug	-2.219	1.87E-04
epigallocatechin-gallate	chemical drug	-2.22	2.07E-18
DICER1	enzyme	-2.22	7.68E-10
herbimycin	chemical - kinase inhibitor	-2.224	6.24E-08
rabeprazole	chemical drug	-2.236	4.65E-09
ubiquinone 9	chemical - endogenous non-mammalian	-2.236	4.65E-09
cyclomaltodextrin	chemical drug	-2.236	2.69E-07
Sod	group	-2.236	6.15E-07

PAEP	other	-2.236	1.71E-06
DUSP5	phosphatase	-2.236	3.03E-06
beta-carotene	chemical - endogenous mammalian	-2.236	7.21E-05
fish oils	chemical drug	-2.236	2.55E-04
sulforafan	chemical drug	-2.258	2.33E-06
apigenin	chemical - endogenous non-mammalian	-2.287	1.97E-08
PTGER4	g-protein coupled receptor	-2.305	1.35E-09
Hdac	group	-2.331	1.50E-14
dexamethasone	chemical drug	-2.351	1.95E-21
TNFAIP3	enzyme	-2.362	1.11E-05
thalidomide	chemical drug	-2.371	7.97E-08
amino acids	chemical - endogenous mammalian	-2.372	1.22E-08
APOE	transporter	-2.378	1.39E-08
spironolactone	chemical drug	-2.391	3.08E-04
IL37	cytokine	-2.393	7.29E-09
PP1	chemical - kinase inhibitor	-2.393	5.28E-06
TRAIP	enzyme	-2.401	8.87E-08
anakinra	biologic drug	-2.401	4.96E-07
minocycline	chemical drug	-2.404	8.45E-05
lipoxin A4	chemical - endogenous mammalian	-2.412	6.30E-09
INSIG1	other	-2.414	1.37E-03
TSC22D3	transcription regulator	-2.419	6.32E-10
Go 6976	chemical - kinase inhibitor	-2.425	2.59E-06
SU6656	chemical toxicant	-2.433	2.23E-06

MAX	transcription regulator	-2.433	7.38E-05
cilostazol	chemical drug	-2.434	3.80E-06
MNT	transcription regulator	-2.449	6.27E-08
IRF9	transcription regulator	-2.449	2.23E-06
baicalein	chemical - endogenous non-mammalian	-2.449	1.27E-05
iron	chemical - endogenous mammalian	-2.449	3.78E-05
HOXA10	transcription regulator	-2.449	1.38E-03
ACOX1	enzyme	-2.449	7.51E-03
tyrphostin AG 1478	chemical - kinase inhibitor	-2.468	9.54E-08
IL1RN	cytokine	-2.478	1.73E-12
bexarotene	chemical drug	-2.53	2.33E-06
NS-398	chemical reagent	-2.55	4.06E-09
nifedipine	chemical drug	-2.557	1.72E-08
FOSL1	transcription regulator	-2.562	1.40E-16
Sn50 peptide	chemical toxicant	-2.581	6.89E-06
mir-8	microrna	-2.592	1.31E-05
LYN	kinase	-2.599	4.19E-08
ABCG1	transporter	-2.603	6.29E-08
NLRP12	other	-2.607	1.55E-07
calphostin C	chemical - kinase inhibitor	-2.607	9.58E-06
sirolimus	chemical drug	-2.613	6.51E-08
etanercept	biologic drug	-2.619	4.92E-08
vitamin E	chemical drug	-2.63	7.71E-06
VIP	other	-2.646	4.98E-08
actinomycin D	chemical drug	-2.724	1.11E-11
Ro31-8220	chemical - kinase inhibitor	-2.744	3.57E-08

GFI1	transcription regulator	-2.758	1.64E-06
miR-16-5p (and other miRNAs w/seed AGCAGCA)	mature microrna	-2.772	2.75E-03
ZFP36	transcription regulator	-2.774	9.75E-18
HMOX1	enzyme	-2.78	4.48E-09
CORT	other	-2.781	2.90E-09
DACH1	transcription regulator	-2.789	1.27E-08
Nr1h	group	-2.804	2.87E-04
SOCS3	phosphatase	-2.847	4.57E-12
diphenyleneiodonium	chemical reagent	-2.949	1.61E-08
COL18A1	other	-2.969	1.13E-06
triamcinolone acetonide	chemical drug	-2.994	4.86E-09
SOCS1	other	-3.012	1.17E-12
Sb202190	chemical - kinase inhibitor	-3.085	1.76E-11
Bay 11-7082	chemical - kinase inhibitor	-3.099	1.24E-09
SFTPA1	transporter	-3.168	3.63E-16
pyrrolidine dithiocarbamate	chemical reagent	-3.18	1.90E-12
AG490	chemical - kinase inhibitor	-3.205	4.06E-09
curcumin	chemical drug	-3.213	1.27E-15
CD3	complex	-3.236	2.95E-09
bisindolylmaleimide I	chemical - kinase inhibitor	-3.253	2.68E-08
wortmannin	chemical - kinase inhibitor	-3.366	9.16E-15
Alpha catenin	group	-3.405	1.88E-08
DUSP1	phosphatase	-3.449	5.82E-17
2-amino-5-phosphonovaleric acid	chemical - other	-3.883	6.88E-15
N-acetyl-L-cysteine	chemical drug	-4.002	3.36E-13

miR-155-5p (miRNAs w/seed UAAUGCU)	mature microrna	-4.022	4.08E-12
SP600125	chemical - kinase inhibitor	-4.814	1.23E-21
SB203580	chemical - kinase inhibitor	-4.94	4.05E-22
LY294002	chemical - kinase inhibitor	-5.698	1.01E-29
U0126	chemical - kinase inhibitor	-6.303	1.94E-42
PD98059	chemical - kinase inhibitor	-6.533	8.92E-29

S4Table10. Networks and associated network functions associated with perfusion driven DGF

ID	Score	Focus Molecules	Top Diseases and Functions
1	37	23	Neurological Disease, Post-Translational Modification, Protein Folding
2	28	19	Cellular Growth and Proliferation, Endocrine System Disorders, Organismal Injury and Abnormalities
3	26	18	Connective Tissue Disorders, Respiratory Disease, Inflammatory Response
4	24	17	Cellular Movement, Hematological System Development and Function, Immune Cell Trafficking
5	24	17	Cell Cycle, Cellular Development, Cell-To-Cell Signaling and Interaction
6	22	16	Neurological Disease, Cardiovascular Disease, Cell Death and Survival
7	20	15	Organismal Injury and Abnormalities, Gene Expression, Cell Cycle
8	17	13	Cellular Development, Cellular Growth and Proliferation, Hematological System Development and Function
9	17	13	Cell Death and Survival, Nervous System Development and Function, Amino Acid Metabolism
10	15	12	Cellular Compromise, Endocrine System Disorders, Organ Morphology
11	15	12	Cell Morphology, Cellular Assembly and Organization, Cellular Development
12	15	12	Cell Cycle, Cellular Development, Cellular Growth and Proliferation
13	13	11	Neurological Disease, Cell Cycle, Cellular Growth and Proliferation

14	13	11	Cellular Movement, Hematological System Development and Function, Immune Cell Trafficking
15	13	11	Cancer, Endocrine System Disorders, Gastrointestinal Disease
16	13	11	Cancer, Dermatological Diseases and Conditions, Organismal Injury and Abnormalities
17	13	11	Embryonic Development, Tissue Morphology, Molecular Transport
18	12	10	Cellular Assembly and Organization, Developmental Disorder, Skeletal and Muscular Disorders
19	10	9	Cell-To-Cell Signaling and Interaction, Cellular Movement, Hematological System Development and Function
20	7	7	Cancer, Cellular Movement, Organismal Injury and Abnormalities
21	2	1	Cellular Assembly and Organization, Cellular Function and Maintenance, Cancer
22	2	1	Inflammatory Disease, Inflammatory Response, Organismal Injury and Abnormalities
23	2	1	Organismal Injury and Abnormalities, Reproductive System Disease
24	2	1	
25	2	1	
