# Supplementary material for: A molecular signature for delayed graft function
Source: Aging Cell. 2018 Aug 9;17(5):e12825. doi: 10.1111/acel.12825 (PMC6156499; doi:10.1111/acel.12825)
Supplement: Supplementary file 6 [file ACEL-17-e12825-s006.pdf]

SD5 (Supplementary Data 5). Reperfusion related genes affected by alternative splicing based on RNAseq analysis, sorted by group ID (median count ≥ 10, false discovery rate [p] < 0.1)

group ID	featureID	exon BaseMean	Dispersion	statististic	p value	p value adj	Post	Pre	log2 fold_Pre_Post	ensembl_gene_id	external_gene_name
ENSG00000067082	E002	84.66581656	0.066486945	31.05137452	2.51E-08	3.00E-07	14.483106	11.9740349	-0.274461648	ENSG00000067082	KLF6
ENSG00000067082	E016	34.37241824	0.063206587	22.34026441	2.28E-06	1.81E-05	10.5717639	8.75120502	-0.272662513	ENSG00000067082	KLF6
ENSG00000067082	E001	10.60531172	0.070970422	19.63977577	9.35E-06	6.75E-05	6.76793783	5.05027455	-0.422354497	ENSG00000067082	KLF6
ENSG00000067082	E011	545.8362402	0.057738255	9.775018891	0.00176899	0.008043525	22.6217278	20.9524079	-0.110593068	ENSG00000067082	KLF6
ENSG00000067082	E003	3085.637742	0.013910252	9.753678806	0.00178964	0.008092722	29.8383411	30.3914133	0.026496436	ENSG00000067082	KLF6
ENSG00000067082	E010	162.4238069	0.050737922	6.429918714	0.01122137	0.039466618	16.9324081	15.4069309	-0.136207665	ENSG00000067082	KLF6
ENSG00000067082	E009	115.1406386	0.058894374	5.065072852	0.02441268	0.076263217	15.3400123	13.9572993	-0.136279833	ENSG00000067082	KLF6
ENSG00000095397	E016	4.623142691	0.111721988	78.11988218	9.70E-19	7.26E-17	6.08413473	2.22495638	-1.45127505	ENSG00000095397	DFNB31
ENSG00000095397	E005	19.46502995	0.004389497	48.78161178	2.86E-12	7.14E-11	6.18310875	7.80006575	0.335153903	ENSG00000095397	DFNB31
ENSG00000095397	E018	34.40237698	0.015784937	40.16289058	2.34E-10	4.18E-09	10.4069193	8.74461865	-0.251075687	ENSG00000095397	DFNB31
ENSG00000095397	E019	27.48243829	0.008981511	37.60954549	8.64E-10	1.32E-08	9.48532991	7.99580243	-0.246455129	ENSG00000095397	DFNB31
ENSG00000095397	E013	4.562588496	0.095002211	33.90757363	5.78E-09	7.67E-08	5.37074422	3.07054482	-0.806627352	ENSG00000095397	DFNB31
ENSG00000095397	E015	2.949454017	0.175297182	26.72194331	2.35E-07	2.30E-06	4.60681522	2.28047711	-1.014434041	ENSG00000095397	DFNB31
ENSG00000095397	E002	28.86720202	0.044438264	22.35230838	2.27E-06	1.81E-05	7.61268363	9.06886296	0.252516555	ENSG00000095397	DFNB31
ENSG00000095397	E007	13.27923809	0.028470181	21.59655422	3.36E-06	2.61E-05	5.24176572	6.52789524	0.316565031	ENSG00000095397	DFNB31
ENSG00000095397	E006	7.19577037	0.006032565	19.90002364	8.16E-06	5.94E-05	3.88912024	5.05651029	0.378698225	ENSG00000095397	DFNB31
ENSG00000095397	E017	20.74701364	0.016631303	16.61071706	4.59E-05	0.000304583	8.35020258	7.25531319	-0.202773305	ENSG00000095397	DFNB31
ENSG00000095397	E004	12.02205194	0.016426796	16.06993814	6.10E-05	0.000392503	5.1522255	6.26546821	0.282226585	ENSG00000095397	DFNB31
ENSG00000095397	E010	17.26559076	0.066772817	15.36885446	8.84E-05	0.000535156	6.12802093	7.34551565	0.261442545	ENSG00000095397	DFNB31
ENSG00000095397	E008	14.35431791	0.043479749	13.61598496	0.00022427	0.001198522	5.62924404	6.72012188	0.255546206	ENSG00000095397	DFNB31
ENSG00000095397	E003	8.727827492	0.011903419	10.77071497	0.00103119	0.005021696	4.55114349	5.43522356	0.256110303	ENSG00000095397	DFNB31
ENSG00000095397	E011	14.56760506	0.031232578	10.52323061	0.00117883	0.005673561	5.77615798	6.72180273	0.218738002	ENSG00000095397	DFNB31
ENSG00000095397	E009	7.833350682	0.063872962	7.955316538	0.00479463	0.018971069	4.20093697	5.0751818	0.272748364	ENSG00000095397	DFNB31
ENSG00000111837	E019	1.717501166	0.155871976	30.80806176	2.85E-08	3.35E-07	3.12304832	0.65410429	-2.255362302	ENSG00000111837	MAK
ENSG00000111837	E002	2.532438402	0.084710931	16.89551556	3.95E-05	0.000266428	2.3888164	3.73142195	0.643429535	ENSG00000111837	MAK

ENSG00000111837	E017	4.261998027	0.025087069	15.38440071	8.77E-05	0.000534702	4.44182516	3.08286862	-0.526879196	ENSG00000111837	MAK
ENSG00000111837	E003	1.39179877	0.129243267	11.98700278	0.00053573	0.002738539	1.71637635	2.88374605	0.748578192	ENSG00000111837	MAK
ENSG00000111837	E018	0.498426346	0.187088913	9.413697969	0.0021537	0.009478603	1.76606891	0.08481772	-4.380032152	ENSG00000111837	MAK
ENSG00000111837	E010	2.146236304	0.052044263	6.056328174	0.01385655	0.047715231	2.54202169	3.27368343	0.364938476	ENSG00000111837	MAK
ENSG00000111837	E020	0.662373805	0.07013464	6.048191986	0.01392054	0.047735865	1.87098144	0.92371613	-1.018273779	ENSG00000111837	MAK
ENSG00000111837	E006	1.083477996	0.253402504	4.605131409	0.03187641	0.094708612	1.65409992	2.51383007	0.603840745	ENSG00000111837	MAK
ENSG00000113070	E004	4.033798276	0.065407083	14.17860299	0.00016625	0.000906119	4.49902568	3.0533917	-0.559199926	ENSG00000113070	HBEGF
ENSG00000113070	E008	7.02208194	0.112161683	4.661359991	0.03084875	0.091987389	5.55157198	4.45423148	-0.317719809	ENSG00000113070	HBEGF
ENSG00000123358	E006	12.19719864	0.13802694	26.90794644	2.13E-07	2.12E-06	5.25644705	7.55563835	0.523465669	ENSG00000123358	NR4A1
ENSG00000123358	E003	5.888759472	0.206175099	21.55165464	3.44E-06	2.65E-05	3.62929153	5.5880258	0.622650731	ENSG00000123358	NR4A1
ENSG00000123358	E010	13.53961907	0.061832911	20.58072219	5.72E-06	4.32E-05	5.86228796	7.54336744	0.363744865	ENSG00000123358	NR4A1
ENSG00000123358	E009	7.986689831	0.074028748	12.18642587	0.00048139	0.002476126	4.66370415	5.95797873	0.353346704	ENSG00000123358	NR4A1
ENSG00000123358	E031	53.01992647	0.150496849	6.605586356	0.01016593	0.036218884	11.557347	11.0847117	-0.060239019	ENSG00000123358	NR4A1
ENSG00000123358	E030	132.9179622	0.155137115	5.58849153	0.01807886	0.058809884	15.376523	15.0325033	-0.03264403	ENSG00000123358	NR4A1
ENSG00000123358	E014	9.215851921	0.014470931	4.504958931	0.03379671	0.09933818	5.92409444	5.33701396	-0.150561855	ENSG00000123358	NR4A1
ENSG00000125347	E026	69.16205132	0.057456375	75.44065728	3.77E-18	2.38E-16	14.1006576	10.185017	-0.469314053	ENSG00000125347	IRF1
ENSG00000125347	E024	89.75747507	0.053228027	73.56121048	9.76E-18	5.74E-16	15.1628685	11.3400411	-0.419116838	ENSG00000125347	IRF1
ENSG00000125347	E025	63.68946089	0.062358768	58.99162099	1.58E-14	5.21E-13	13.676578	10.0307113	-0.447283389	ENSG00000125347	IRF1
ENSG00000125347	E003	485.6983269	0.013764134	45.20777861	1.77E-11	3.65E-10	20.6595325	21.8432147	0.080377587	ENSG00000125347	IRF1
ENSG00000125347	E030	24.98869217	0.090428273	43.9733548	3.33E-11	6.68E-10	10.0848807	6.61318669	-0.608776487	ENSG00000125347	IRF1
ENSG00000125347	E019	20.88067031	0.070784047	43.71959689	3.79E-11	7.25E-10	9.30485698	6.26033848	-0.571743313	ENSG00000125347	IRF1
ENSG00000125347	E007	98.36677532	0.056033554	41.81321304	1.00E-10	1.84E-09	15.2774277	12.377539	-0.303677157	ENSG00000125347	IRF1
ENSG00000125347	E023	18.7456177	0.074462703	41.853893	9.84E-11	1.84E-09	8.9556672	5.77261683	-0.633575451	ENSG00000125347	IRF1
ENSG00000125347	E029	13.13106061	0.081822161	39.02027587	4.19E-10	7.19E-09	7.8853069	4.86956466	-0.695374107	ENSG00000125347	IRF1
ENSG00000125347	E028	34.21468097	0.087011321	37.9343355	7.32E-10	1.15E-08	11.1252574	7.88369965	-0.496893998	ENSG00000125347	IRF1
ENSG00000125347	E018	134.2811513	0.004644016	33.849182	5.96E-09	7.78E-08	14.9940292	15.7540899	0.071338296	ENSG00000125347	IRF1
ENSG00000125347	E006	165.1860558	0.01012923	33.20798745	8.28E-09	1.05E-07	15.7399358	16.7900256	0.093174773	ENSG00000125347	IRF1
ENSG00000125347	E031	5.689050358	0.09126314	33.11708134	8.68E-09	1.08E-07	5.64360333	3.09974384	-0.864467593	ENSG00000125347	IRF1
ENSG00000125347	E016	102.4163958	0.005647355	31.77025271	1.74E-08	2.10E-07	13.7689901	14.5835162	0.082915857	ENSG00000125347	IRF1
ENSG00000125347	E008	105.638131	0.013993427	30.31530687	3.67E-08	4.20E-07	13.6569985	14.8847668	0.124196169	ENSG00000125347	IRF1

ENSG00000125347	E017	21.58328221	0.090754233	28.55181772	9.12E-08	9.88E-07	9.27438777	6.60118749	-0.490526474	ENSG00000125347	IRF1
ENSG00000125347	E013	31.32445227	0.052084521	28.38970252	9.92E-08	1.05E-06	10.4217671	8.13798618	-0.356856187	ENSG00000125347	IRF1
ENSG00000125347	E011	134.0861832	0.007792802	28.02399116	1.20E-07	1.23E-06	14.8884341	15.7439275	0.080603459	ENSG00000125347	IRF1
ENSG00000125347	E005	27.94275504	0.060035738	26.12659685	3.20E-07	3.02E-06	10.0427144	7.76634279	-0.370841971	ENSG00000125347	IRF1
ENSG00000125347	E020	93.46264041	0.006166746	24.14960501	8.91E-07	7.80E-06	13.4159088	14.1450505	0.076352539	ENSG00000125347	IRF1
ENSG00000125347	E012	90.3183981	0.011784401	23.77340661	1.08E-06	9.10E-06	13.1184434	14.0550568	0.099492733	ENSG00000125347	IRF1
ENSG00000125347	E009	25.58901567	0.058472114	21.4742068	3.59E-06	2.73E-05	9.65529039	7.60107181	-0.345116787	ENSG00000125347	IRF1
ENSG00000125347	E010	23.26538297	0.064476426	20.31906478	6.55E-06	4.90E-05	9.33834414	7.15418866	-0.384378593	ENSG00000125347	IRF1
ENSG00000125347	E015	117.5414045	0.005409269	19.01892945	1.29E-05	9.18E-05	14.4920333	15.0475156	0.054265283	ENSG00000125347	IRF1
ENSG00000125347	E014	129.5077582	0.004668482	17.55577319	2.79E-05	0.000194592	14.9490407	15.4404119	0.046658341	ENSG00000125347	IRF1
ENSG00000125347	E036	6.682775733	0.019537365	15.76220609	7.18E-05	0.000447806	5.64085743	4.17156818	-0.43532465	ENSG00000125347	IRF1
ENSG00000125347	E021	205.1857313	0.005464699	14.27017869	0.00015835	0.000886567	17.0678981	17.4744188	0.033959072	ENSG00000125347	IRF1
ENSG00000125347	E004	94.70767354	0.014983027	10.44302806	0.00123113	0.005801643	13.5110554	14.1666347	0.068356714	ENSG00000125347	IRF1
ENSG00000125347	E037	5.194777286	0.028078032	10.43926713	0.00123364	0.005801643	5.00122992	3.7517513	-0.414718735	ENSG00000125347	IRF1
ENSG00000125347	E027	105.9555185	0.007066071	6.538722077	0.01055511	0.037443359	14.1854627	14.4485316	0.026509673	ENSG00000125347	IRF1
ENSG00000125347	E002	19.64040103	0.016648481	5.480609426	0.01922855	0.061576264	7.61069144	8.06705318	0.084014237	ENSG00000125347	IRF1
ENSG00000125740	E002	468.6662626	0.006374883	23.15367931	1.50E-06	1.21E-05	15.9121207	17.8118435	0.162710711	ENSG00000125740	FOSB
ENSG00000125740	E023	302.7178442	0.064357622	5.651618545	0.01743927	0.057410072	14.0553232	12.0305699	-0.224411648	ENSG00000125740	FOSB
ENSG00000125740	E016	68.4921267	0.015596765	4.967458339	0.02582858	0.079913224	8.33524327	6.71485402	-0.311868269	ENSG00000125740	FOSB
ENSG00000127528	E001	10.80940086	0.040628905	7.594326199	0.00585523	0.022518001	5.55614665	6.54720429	0.236794316	ENSG00000127528	KLF2
ENSG00000128016	E009	254.5693149	0.003445138	11.17042701	0.00083111	0.004095854	18.3713087	18.0291656	-0.027121769	ENSG00000128016	ZFP36
ENSG00000128016	E008	649.9524454	0.002079032	6.397830465	0.01142599	0.040015279	22.6769395	22.5606677	-0.007416178	ENSG00000128016	ZFP36
ENSG00000136527	E018	327.6989353	0.004151756	93.74029459	3.60E-22	9.87E-20	17.9537029	18.8827434	0.072786954	ENSG00000136527	TRA2B
ENSG00000136527	E004	338.7659903	0.008538401	91.17395049	1.32E-21	2.71E-19	17.7794739	19.1719644	0.108785532	ENSG00000136527	TRA2B
ENSG00000136527	E030	788.4763297	0.017632744	86.71037637	1.26E-20	2.07E-18	24.9176728	22.0070945	-0.179200647	ENSG00000136527	TRA2B
ENSG00000136527	E007	625.7987346	0.008893096	85.34919869	2.50E-20	3.43E-18	20.8773982	22.0670107	0.079949277	ENSG00000136527	TRA2B
ENSG00000136527	E009	233.4816969	0.006713804	82.23276264	1.21E-19	1.24E-17	16.2301023	17.3842363	0.099107598	ENSG00000136527	TRA2B
ENSG00000136527	E033	146.2385486	0.015589309	80.85708859	2.43E-19	2.22E-17	17.1149753	14.1804524	-0.271355649	ENSG00000136527	TRA2B
ENSG00000136527	E008	182.0484136	0.006885748	79.9497016	3.84E-19	3.16E-17	15.0631648	16.2647831	0.110726668	ENSG00000136527	TRA2B
ENSG00000136527	E005	133.7528507	0.009320409	71.05872029	3.47E-17	1.90E-15	13.5219115	14.9164997	0.141609918	ENSG00000136527	TRA2B

ENSG00000136527	E006	133.1097605	0.009230267	70.88828735	3.78E-17	1.94E-15	13.507209	14.8937046	0.14097305	ENSG00000136527	TRA2B
ENSG00000136527	E038	233.4390807	0.022941901	68.68089844	1.16E-16	5.60E-15	19.3662107	16.1953146	-0.257965199	ENSG00000136527	TRA2B
ENSG00000136527	E019	326.0506721	0.005699462	68.35418132	1.37E-16	6.25E-15	18.0004464	18.8680217	0.067910479	ENSG00000136527	TRA2B
ENSG00000136527	E036	595.3086316	0.023059028	66.47333397	3.55E-16	1.54E-14	23.6534084	20.6366681	-0.196838033	ENSG00000136527	TRA2B
ENSG00000136527	E014	311.7873076	0.005494347	63.9583383	1.27E-15	5.00E-14	17.8226406	18.64178	0.064828513	ENSG00000136527	TRA2B
ENSG00000136527	E031	69.65090401	0.012201053	63.87162618	1.33E-15	5.00E-14	13.619242	11.1478048	-0.288886766	ENSG00000136527	TRA2B
ENSG00000136527	E034	104.1506567	0.019855223	63.85849043	1.34E-15	5.00E-14	15.6135491	12.7295645	-0.294615451	ENSG00000136527	TRA2B
ENSG00000136527	E016	274.9539665	0.029618317	59.82779824	1.04E-14	3.55E-13	20.2276634	16.8953844	-0.259700507	ENSG00000136527	TRA2B
ENSG00000136527	E003	387.995319	0.011985847	58.6008789	1.93E-14	6.11E-13	18.5780756	19.8015631	0.09201325	ENSG00000136527	TRA2B
ENSG00000136527	E032	89.36851001	0.018633362	54.47533138	1.57E-13	4.47E-12	14.8081879	12.1691455	-0.283167231	ENSG00000136527	TRA2B
ENSG00000136527	E015	222.3992063	0.006118087	53.28583929	2.88E-13	7.91E-12	16.2699041	17.0760002	0.069764335	ENSG00000136527	TRA2B
ENSG00000136527	E037	133.1187086	0.025965471	51.80989737	6.11E-13	1.62E-11	16.6641842	13.7628801	-0.275968278	ENSG00000136527	TRA2B
ENSG00000136527	E017	217.8572524	0.005933365	51.69783876	6.47E-13	1.66E-11	16.1907054	16.9693703	0.067767184	ENSG00000136527	TRA2B
ENSG00000136527	E013	256.3480808	0.006968257	46.36330371	9.82E-12	2.25E-10	16.9529934	17.7233915	0.064114667	ENSG00000136527	TRA2B
ENSG00000136527	E026	514.721324	0.008008529	46.39609337	9.66E-12	2.25E-10	20.3056061	21.0263603	0.050321044	ENSG00000136527	TRA2B
ENSG00000136527	E035	81.23330546	0.018553021	45.67026442	1.40E-11	3.03E-10	14.2598725	11.7711217	-0.276709286	ENSG00000136527	TRA2B
ENSG00000136527	E010	116.862546	0.008935057	45.23859027	1.74E-11	3.65E-10	13.2369521	14.22694	0.104054415	ENSG00000136527	TRA2B
ENSG00000136527	E029	43.3013292	0.013409539	43.85796142	3.53E-11	6.92E-10	11.5204111	9.33648574	-0.303240679	ENSG00000136527	TRA2B
ENSG00000136527	E027	376.0124762	0.009842311	38.51470424	5.43E-10	9.13E-09	18.7875616	19.5487858	0.057301171	ENSG00000136527	TRA2B
ENSG00000136527	E011	167.8993765	0.013641763	37.91966173	7.37E-10	1.15E-08	14.8533429	15.8758731	0.09604828	ENSG00000136527	TRA2B
ENSG00000136527	E022	35.98188923	0.030492097	37.04641853	1.15E-09	1.70E-08	11.1946235	8.52841074	-0.392457185	ENSG00000136527	TRA2B
ENSG00000136527	E012	265.0707115	0.008208392	36.91504253	1.23E-09	1.75E-08	17.1888364	17.8711051	0.056156962	ENSG00000136527	TRA2B
ENSG00000136527	E021	134.9986754	0.031954959	35.71223303	2.29E-09	3.14E-08	16.7003293	13.871819	-0.267719571	ENSG00000136527	TRA2B
ENSG00000136527	E001	70.23274925	0.018731844	33.61443387	6.72E-09	8.64E-08	13.4207208	11.2649933	-0.252615695	ENSG00000136527	TRA2B
ENSG00000136527	E028	154.1159017	0.008098002	28.87010921	7.74E-08	8.49E-07	14.712589	15.3601257	0.06213888	ENSG00000136527	TRA2B
ENSG00000136527	E024	19.82897936	0.029284301	25.3509476	4.78E-07	4.47E-06	8.8373119	6.733809	-0.392184802	ENSG00000136527	TRA2B
ENSG00000136527	E023	24.70135239	0.030963813	24.46149448	7.58E-07	6.93E-06	9.59271683	7.42166103	-0.370197361	ENSG00000136527	TRA2B
ENSG00000136527	E025	45.04449154	0.044071727	24.24143494	8.50E-07	7.60E-06	12.0118358	9.45749892	-0.344926046	ENSG00000136527	TRA2B
ENSG00000136527	E039	262.0893953	0.013900778	16.26702455	5.50E-05	0.000359331	17.3538424	17.7982909	0.036483584	ENSG00000136527	TRA2B
ENSG00000136527	E040	42.82145254	0.030771042	13.32182226	0.00026234	0.001392915	9.30842804	10.0903025	0.116359963	ENSG00000136527	TRA2B

ENSG00000136527	E041	28.58780995	0.027756333	10.79589217	0.00101726	0.004983342	7.96793412	8.61439816	0.112544288	ENSG00000136527	TRA2B
ENSG00000136527	E020	19.83099609	0.047218762	7.038196438	0.00797893	0.029185168	8.33206244	6.88553401	-0.275105103	ENSG00000136527	TRA2B
ENSG00000136826	E006	138.8270541	0.004374742	8.932453099	0.00280148	0.012071292	15.0337952	14.6006401	-0.042177632	ENSG00000136826	KLF4
ENSG00000136826	E015	1.463264094	0.170851125	6.008704618	0.01423547	0.048613258	1.86534585	2.85349278	0.613285768	ENSG00000136826	KLF4
ENSG00000136826	E002	377.5030361	0.015313583	5.852674123	0.0155534	0.05267674	19.2912917	19.8325779	0.039922466	ENSG00000136826	KLF4
ENSG00000136826	E007	182.7525989	0.003402311	4.919443512	0.02655607	0.081550915	16.206789	15.9996998	-0.018553447	ENSG00000136826	KLF4
ENSG00000138166	E004	12.03631725	0.071569511	5.061466059	0.02446354	0.076263217	6.83990057	5.93776724	-0.204054811	ENSG00000138166	DUSP5
ENSG00000141682	E005	2.183768484	0.176843328	5.638796632	0.01756726	0.057601018	3.16558051	1.99357705	-0.667110722	ENSG00000141682	PMAIP1
ENSG00000142178	E016	0.124896475	0.090739887	5.082317083	0.02417105	0.07592661	0.27770157	0.94298953	1.763706409	ENSG00000142178	SIK1
ENSG00000144655	E006	48.7956093	0.01068494	10.18881214	0.00141295	0.006532923	11.5992364	10.817509	-0.100661517	ENSG00000144655	CSRNP1
ENSG00000144655	E007	7.445069173	0.087405639	7.40957555	0.00648776	0.024719578	4.49955351	5.65452743	0.32962461	ENSG00000144655	CSRNP1
ENSG00000148339	E002	22.18980876	0.094846319	47.22646981	6.32E-12	1.53E-10	6.17751589	9.35057454	0.598028197	ENSG00000148339	SLC25A25
ENSG00000148339	E010	28.75760299	0.080948293	16.86280816	4.02E-05	0.000268855	10.0024474	8.13749299	-0.297696748	ENSG00000148339	SLC25A25
ENSG00000148339	E001	2.476513766	0.055781014	14.84456719	0.00011674	0.000676621	2.29155646	3.47586482	0.601044144	ENSG00000148339	SLC25A25
ENSG00000148339	E006	59.59280246	0.140207149	14.42403683	0.00014593	0.000828266	12.9416212	10.6669287	-0.278873515	ENSG00000148339	SLC25A25
ENSG00000148339	E005	229.7598036	0.014402234	9.595210811	0.00195086	0.008738716	18.0621866	17.5219104	-0.043812474	ENSG00000148339	SLC25A25
ENSG00000148339	E020	5.604258744	0.152821959	9.592502483	0.00195373	0.008738716	5.08190616	3.72015322	-0.450007693	ENSG00000148339	SLC25A25
ENSG00000148339	E011	244.8463511	0.001370532	8.07619766	0.00448505	0.017831847	17.9966549	18.1479639	0.012078925	ENSG00000148339	SLC25A25
ENSG00000148339	E007	45.53162867	0.160811784	7.364250529	0.00665333	0.025233607	11.6801737	9.88724393	-0.240421403	ENSG00000148339	SLC25A25
ENSG00000148339	E003	9.822409472	0.214251377	5.888581011	0.01523938	0.05182647	5.06080531	6.30780572	0.31777125	ENSG00000148339	SLC25A25
ENSG00000154479	E014	3.139863162	0.135990022	15.29480988	9.20E-05	0.000552484	2.46683662	3.92084646	0.668502977	ENSG00000154479	CCDC173
ENSG00000154479	E010	5.042871042	0.009844246	10.63696806	0.00110849	0.005366408	4.85847041	3.92194262	-0.308933753	ENSG00000154479	CCDC173
ENSG00000154479	E012	2.226966999	0.030223861	10.302458	0.00132853	0.006212394	3.47006787	2.41472575	-0.523104534	ENSG00000154479	CCDC173
ENSG00000154479	E004	2.921899796	0.085009604	8.148599953	0.00430944	0.017557776	2.6825117	3.70347017	0.465293253	ENSG00000154479	CCDC173
ENSG00000154479	E009	3.526098296	0.020775228	5.569743704	0.01827344	0.059208808	3.92782327	3.20119543	-0.295119263	ENSG00000154479	CCDC173
ENSG00000154479	E002	2.369750277	0.023658419	4.746422494	0.02935927	0.088568784	2.57247382	3.24934007	0.336990347	ENSG00000154479	CCDC173
ENSG00000160888	E002	10.4638387	0.049675141	178.5658966	9.97E-41	8.20E-38	3.41475013	8.08944062	1.244259929	ENSG00000160888	IER2
ENSG00000160888	E003	75.66960363	0.111689965	128.9274642	7.03E-30	2.89E-27	9.27259644	15.8394661	0.772478435	ENSG00000160888	IER2
ENSG00000160888	E005	1062.700675	0.004494567	57.80245813	2.90E-14	8.83E-13	24.9602498	24.5102773	-0.026245566	ENSG00000160888	IER2
ENSG00000160888	E001	0.634895193	0.165883995	26.98473155	2.05E-07	2.06E-06	0.58710437	2.25025976	1.938402659	ENSG00000160888	IER2

ENSG00000160888	E006	546.8870998	0.00472867	10.46005289	0.00121984	0.005801643	21.788568	21.4156146	-0.024908305	ENSG00000160888	IER2
ENSG00000162772	E011	241.5513659	0.001886991	8.100229881	0.00442596	0.017682373	16.1797169	15.8134666	-0.033032697	ENSG00000162772	ATF3
ENSG00000162772	E012	130.1416398	0.002531625	7.252442178	0.00708047	0.026487377	13.4771436	12.9752163	-0.054756165	ENSG00000162772	ATF3
ENSG00000163660	E069	50.51243555	0.131282347	76.17141599	2.60E-18	1.78E-16	NA	NA	NA	ENSG00000163660	CCNL1
ENSG00000163660	E068	15.86057388	0.104522759	23.6502905	1.16E-06	9.60E-06	NA	NA	NA	ENSG00000163660	CCNL1
ENSG00000163660	E036	273.478019	0.0010591	16.31899379	5.35E-05	0.000352407	NA	NA	NA	ENSG00000163660	CCNL1
ENSG00000163660	E025	360.4398688	0.003808032	15.78307229	7.10E-05	0.000446275	NA	NA	NA	ENSG00000163660	CCNL1
ENSG00000163660	E013	41.37651331	0.035638729	14.86710934	0.00011536	0.000673324	NA	NA	NA	ENSG00000163660	CCNL1
ENSG00000163660	E015	79.51020881	0.045866593	14.29909953	0.00015594	0.000879029	NA	NA	NA	ENSG00000163660	CCNL1
ENSG00000163660	E023	314.7365299	0.005389252	14.22487911	0.00016221	0.000895238	NA	NA	NA	ENSG00000163660	CCNL1
ENSG00000163660	E043	309.5382061	0.002345075	13.92153746	0.0001906	0.001025264	NA	NA	NA	ENSG00000163660	CCNL1
ENSG00000163660	E002	51.24289213	0.027217837	12.56935614	0.00039212	0.002055527	NA	NA	NA	ENSG00000163660	CCNL1
ENSG00000163660	E042	292.9397807	0.00407857	11.86707549	0.00057135	0.002902602	NA	NA	NA	ENSG00000163660	CCNL1
ENSG00000163660	E009	125.8297205	0.029751153	11.26676403	0.00078907	0.003935793	NA	NA	NA	ENSG00000163660	CCNL1
ENSG00000163660	E052	202.0088569	0.037478392	10.23597536	0.00137728	0.006403952	NA	NA	NA	ENSG00000163660	CCNL1
ENSG00000163660	E016	425.4881851	0.006002075	10.09471782	0.00148695	0.006836644	NA	NA	NA	ENSG00000163660	CCNL1
ENSG00000163660	E014	55.28034171	0.040035444	9.512098451	0.00204122	0.009043883	NA	NA	NA	ENSG00000163660	CCNL1
ENSG00000163660	E047	580.7926642	0.015027153	7.679957059	0.00558375	0.021676538	NA	NA	NA	ENSG00000163660	CCNL1
ENSG00000163660	E012	443.7288122	0.00603332	7.425406319	0.00643093	0.024617003	NA	NA	NA	ENSG00000163660	CCNL1
ENSG00000163660	E022	277.1003373	0.005450956	6.943219215	0.00841376	0.030504497	NA	NA	NA	ENSG00000163660	CCNL1
ENSG00000163660	E037	196.006976	0.001440871	6.819932973	0.0090146	0.032539525	NA	NA	NA	ENSG00000163660	CCNL1
ENSG00000163660	E057	63.76308206	0.114295352	6.23334154	0.01253669	0.043719066	NA	NA	NA	ENSG00000163660	CCNL1
ENSG00000163660	E011	43.13163574	0.05367242	6.204729818	0.01274094	0.044057955	NA	NA	NA	ENSG00000163660	CCNL1
ENSG00000163660	E010	376.3055903	0.00689965	5.787958042	0.01613632	0.053984523	NA	NA	NA	ENSG00000163660	CCNL1
ENSG00000163660	E048	184.5493948	0.007498909	5.655591562	0.0173998	0.057410072	NA	NA	NA	ENSG00000163660	CCNL1
ENSG00000163660	E056	102.7471151	0.097455475	5.592334246	0.01803924	0.058809884	NA	NA	NA	ENSG00000163660	CCNL1
ENSG00000163660	E020	51.26625555	0.065127505	5.393468641	0.02021225	0.064475524	NA	NA	NA	ENSG00000163660	CCNL1
ENSG00000163660	E050	169.1735977	0.003788728	5.181519695	0.02282835	0.071983645	NA	NA	NA	ENSG00000163660	CCNL1
ENSG00000163660	E051	250.1766442	0.003189128	4.924018831	0.02648584	0.081550915	NA	NA	NA	ENSG00000163660	CCNL1
ENSG00000163660	E017	193.0084152	0.005067479	4.848026123	0.02767779	0.084365998	NA	NA	NA	ENSG00000163660	CCNL1

ENSG00000163660	E055	220.9138112	0.003412961	4.74524097	0.02937944	0.088568784	NA	NA	NA	ENSG00000163660	CCNL1
ENSG00000163660	E008	60.8701464	0.042696286	4.571579059	0.03250659	0.095888612	NA	NA	NA	ENSG00000163660	CCNL1
ENSG00000165046	E011	3.276494318	0.075121684	30.6164784	3.14E-08	3.64E-07	4.45874118	2.3438319	-0.92776735	ENSG00000165046	LETM2
ENSG00000165046	E009	8.777272801	0.1337979	26.2394761	3.02E-07	2.92E-06	6.63891792	4.22384332	-0.652391795	ENSG00000165046	LETM2
ENSG00000165046	E017	33.4473874	0.010214569	26.16983808	3.13E-07	2.99E-06	8.84579487	10.0435021	0.183198724	ENSG00000165046	LETM2
ENSG00000165046	E029	2.339869448	0.050985994	20.05825587	7.51E-06	5.57E-05	3.86645658	2.21275563	-0.805167882	ENSG00000165046	LETM2
ENSG00000165046	E021	2.150422166	0.050431947	19.94925903	7.95E-06	5.84E-05	3.62088082	2.00048466	-0.855991127	ENSG00000165046	LETM2
ENSG00000165046	E010	2.838037181	0.112902475	16.22799319	5.62E-05	0.000363922	4.00505615	2.40399217	-0.736390273	ENSG00000165046	LETM2
ENSG00000165046	E030	3.204679173	0.071397139	15.01113205	0.00010688	0.000628295	4.24363875	2.75439244	-0.623567723	ENSG00000165046	LETM2
ENSG00000165046	E018	8.131211802	0.020409702	14.51442661	0.00013909	0.00079494	4.63553677	5.77700084	0.3175843	ENSG00000165046	LETM2
ENSG00000165046	E016	11.265885	0.004206443	14.05698693	0.00017735	0.000960277	5.64988701	6.55333993	0.214008351	ENSG00000165046	LETM2
ENSG00000165046	E040	9.794234974	0.04092027	12.27804324	0.00045832	0.002372304	6.60222082	5.1923206	-0.346571928	ENSG00000165046	LETM2
ENSG00000165046	E015	11.96043735	0.005977854	11.68463502	0.00063018	0.003181847	5.82770465	6.6611369	0.192840667	ENSG00000165046	LETM2
ENSG00000165046	E014	23.42703205	0.010609499	11.44960267	0.00071509	0.003588545	7.90685999	8.69623977	0.137286839	ENSG00000165046	LETM2
ENSG00000165046	E019	7.934394387	0.02296103	10.45145994	0.00122553	0.005801643	4.63024339	5.61390601	0.277916879	ENSG00000165046	LETM2
ENSG00000165046	E026	0.637569065	0.080404121	9.38843958	0.00218358	0.009558969	2.11487102	0.96567055	-1.13096669	ENSG00000165046	LETM2
ENSG00000165046	E013	10.93556576	0.020360476	4.77701463	0.02884208	0.087590526	5.73800251	6.32071985	0.139540273	ENSG00000165046	LETM2
ENSG00000170345	E014	216.0097962	0.118391249	34.44467571	4.39E-09	5.92E-08	15.005237	19.5586907	0.382343692	ENSG00000170345	FOS
ENSG00000170345	E013	278.591572	0.127792836	28.12222305	1.14E-07	1.19E-06	16.2101648	20.5920284	0.345187186	ENSG00000170345	FOS
ENSG00000170345	E006	242.7835293	0.143580993	24.31104664	8.20E-07	7.41E-06	15.4819922	20.1903653	0.383075882	ENSG00000170345	FOS
ENSG00000170345	E005	60.65191406	0.145653297	24.21169381	8.63E-07	7.64E-06	9.71113699	14.0099	0.528734534	ENSG00000170345	FOS
ENSG00000170345	E020	3276.337255	0.002752327	24.0094627	9.59E-07	8.30E-06	28.9237264	28.6814812	-0.012133904	ENSG00000170345	FOS
ENSG00000170345	E017	1100.14866	0.002716861	23.93056339	9.99E-07	8.56E-06	23.7484426	23.4123332	-0.020564186	ENSG00000170345	FOS
ENSG00000170345	E008	270.4561433	0.155157904	23.44107344	1.29E-06	1.06E-05	16.0722655	20.4628879	0.348436463	ENSG00000170345	FOS
ENSG00000170345	E018	137.6728526	0.159179348	23.33443002	1.36E-06	1.11E-05	13.0624568	17.4578359	0.418448471	ENSG00000170345	FOS
ENSG00000170345	E012	141.1840003	0.142393707	22.17817234	2.48E-06	1.95E-05	13.2857438	17.3097039	0.381702044	ENSG00000170345	FOS
ENSG00000170345	E007	112.6532243	0.153533826	18.53248452	1.67E-05	0.000117494	12.2672549	16.4476855	0.423072137	ENSG00000170345	FOS
ENSG00000170345	E015	512.6664779	0.002888366	17.22297454	3.32E-05	0.000227966	20.1630051	19.8343574	-0.023709017	ENSG00000170345	FOS
ENSG00000170345	E019	827.363348	0.003387109	15.57212753	7.94E-05	0.000491428	22.3847964	22.1162122	-0.017414877	ENSG00000170345	FOS
ENSG00000170345	E016	821.2232524	0.002228004	14.25702073	0.00015946	0.000886754	22.3508431	22.1772567	-0.011248335	ENSG00000170345	FOS

ENSG00000170345	E011	1504.202051	0.001815652	10.0706287	0.00150652	0.006888127	25.1904764	25.2133732	0.001310743	ENSG00000170345	FOS
ENSG00000170345	E021	3202.325312	0.005217466	7.048968406	0.00793109	0.02913968	28.7809977	28.6736535	-0.005390864	ENSG00000170345	FOS
ENSG00000170345	E004	1232.624624	0.006393309	4.877380645	0.02721088	0.083251137	24.2121215	24.192334	-0.001179537	ENSG00000170345	FOS
ENSG00000172602	E010	2.583145519	0.091894207	28.48882706	9.42E-08	1.01E-06	3.79575352	1.59715098	-1.248885611	ENSG00000172602	RND1
ENSG00000172602	E012	4.791073254	0.151368663	14.80824613	0.00011901	0.000684954	4.65749735	3.09837442	-0.588043452	ENSG00000172602	RND1
ENSG00000173334	E007	31.5446111	0.065775065	11.25001846	0.00079622	0.003947536	10.1757963	8.63880195	-0.236238545	ENSG00000173334	TRIB1
ENSG00000173334	E001	335.9902303	0.01394387	7.29209922	0.00692585	0.026146673	19.7872837	19.1936396	-0.043945274	ENSG00000173334	TRIB1
ENSG00000173334	E002	12.55098841	0.093356105	7.273563241	0.00699768	0.026297234	6.98526514	5.98242958	-0.223583368	ENSG00000173334	TRIB1
ENSG00000173334	E003	32.7847238	0.08891562	6.509726913	0.0107286	0.037895435	10.147016	8.93475654	-0.183555201	ENSG00000173334	TRIB1
ENSG00000173334	E010	648.2473003	0.008631904	5.68701475	0.0170909	0.056716994	22.4146421	22.6060758	0.012269115	ENSG00000173334	TRIB1
ENSG00000173846	E015	10.6925125	0.066672036	29.50067288	5.59E-08	6.30E-07	6.94300649	4.69799826	-0.563514342	ENSG00000173846	PLK3
ENSG00000173846	E010	6.185574279	0.061105749	15.92867314	6.58E-05	0.000416404	5.44843082	3.90018562	-0.482298	ENSG00000173846	PLK3
ENSG00000173846	E027	32.11740436	0.009898215	9.310031039	0.00227903	0.009924014	9.23895341	9.94822407	0.10670957	ENSG00000173846	PLK3
ENSG00000173846	E028	42.61206494	0.006379512	9.262066732	0.00233949	0.01013368	10.3451184	10.9524526	0.082303805	ENSG00000173846	PLK3
ENSG00000173846	E026	24.53744061	0.005904085	8.566423575	0.00342419	0.014378109	8.35418337	9.01722551	0.110184791	ENSG00000173846	PLK3
ENSG00000173846	E024	8.344700424	0.046163235	7.880375439	0.00499741	0.019492269	5.83239718	4.91166868	-0.247875723	ENSG00000173846	PLK3
ENSG00000173846	E020	6.025983458	0.102724772	5.79683152	0.01605508	0.053931966	5.04301641	3.99939079	-0.334506661	ENSG00000173846	PLK3
ENSG00000173846	E025	5.644729375	0.06164428	4.591919948	0.03212301	0.095097981	4.98178481	4.22931497	-0.236238699	ENSG00000173846	PLK3
ENSG00000179388	E001	32.72730161	0.043698462	8.792449325	0.0030248	0.012832006	8.84016946	9.75250178	0.141698332	ENSG00000179388	EGR3
ENSG00000179388	E009	0.631794971	0.054981195	5.825486569	0.0157956	0.053277777	1.62288832	0.12784039	-3.666148144	ENSG00000179388	EGR3
ENSG00000182950	E004	6.471097917	0.142810684	5.017540464	0.02509179	0.077926564	4.4425075	5.0143228	0.174680661	ENSG00000182950	ODF3L1
ENSG00000184545	E010	1.872095948	0.051926006	17.1788197	3.40E-05	0.000231398	3.37319895	2.02086079	-0.739147468	ENSG00000184545	DUSP8
ENSG00000184545	E009	21.74962306	0.007958768	8.807066658	0.00300066	0.012822501	8.37087574	7.74897899	-0.11137233	ENSG00000184545	DUSP8
ENSG00000184545	E007	4.797283715	0.064025894	7.095325836	0.00772852	0.028522759	4.68439125	3.75035048	-0.320836153	ENSG00000184545	DUSP8
ENSG00000184545	E002	65.27830626	0.016006688	6.958452398	0.00834242	0.030379684	11.9151217	12.4174717	0.059577769	ENSG00000184545	DUSP8
ENSG00000184557	E006	0.3768286	0.128023038	4.701015954	0.03014479	0.090544397	0.89299723	1.59281857	0.834854343	ENSG00000184557	SOCS3
ENSG00000186352	E009	55.53553414	0.132438016	15.51132575	8.20E-05	0.000503703	13.0507928	10.0164723	-0.381762947	ENSG00000186352	ANKRD37
ENSG00000186352	E012	138.0472709	0.008604066	9.509651752	0.00204394	0.009043883	15.168377	15.6205776	0.042381072	ENSG00000186352	ANKRD37
ENSG00000186352	E013	85.19112257	0.011967005	8.803229718	0.00300698	0.012822501	13.0099684	13.5454905	0.058195179	ENSG00000186352	ANKRD37
ENSG00000186352	E011	85.97425618	0.001931055	8.12978484	0.00435439	0.017638283	13.1971951	13.3964685	0.021621403	ENSG00000186352	ANKRD37

ENSG00000186352	E003	12.06503966	0.117664506	6.722332691	0.0095213	0.034218476	7.25014137	5.49793914	-0.399118191	ENSG00000186352	ANKRD37
ENSG00000186352	E002	25.89469322	0.088194988	6.221503062	0.01262079	0.043826634	9.54165157	7.90858825	-0.270818822	ENSG00000186352	ANKRD37
ENSG00000186352	E014	27.7247205	0.117671153	5.554636455	0.0184318	0.05926254	9.77660359	8.17401204	-0.25828899	ENSG00000186352	ANKRD37
ENSG00000186352	E015	119.6704783	0.009039711	5.349880136	0.0207237	0.065598466	14.6445179	14.9268715	0.027551122	ENSG00000186352	ANKRD37
ENSG00000189143	E002	0.637105991	0.044015018	8.109991748	0.00440219	0.017673193	0.95181538	1.74225471	0.872201883	ENSG00000189143	CLDN4
ENSG00000189143	E001	2.115973502	0.540346815	7.885807803	0.00498242	0.019492269	1.97049435	2.83605876	0.525329813	ENSG00000189143	CLDN4
ENSG00000189143	E011	15.42912543	0.049933942	7.663110495	0.00563613	0.021777142	6.39472992	7.32328813	0.195608131	ENSG00000189143	CLDN4
ENSG00000196843	E005	40.03715456	0.121524077	82.40498857	1.11E-19	1.24E-17	12.5713321	7.63507008	-0.71942422	ENSG00000196843	ARID5A
ENSG00000196843	E014	176.1062273	0.020771846	63.61840894	1.51E-15	5.40E-14	15.7671798	16.9720752	0.106238341	ENSG00000196843	ARID5A
ENSG00000196843	E010	14.08380211	0.030015165	39.65314102	3.03E-10	5.31E-09	7.94026208	5.88665513	-0.431738517	ENSG00000196843	ARID5A
ENSG00000196843	E006	3.743335727	0.029427021	36.9126669	1.24E-09	1.75E-08	4.71142106	2.64612486	-0.832281132	ENSG00000196843	ARID5A
ENSG00000196843	E012	34.17491997	0.004430479	19.22982935	1.16E-05	8.29E-05	9.13086947	9.99746499	0.130810079	ENSG00000196843	ARID5A
ENSG00000196843	E013	27.26760998	0.011362537	15.9809038	6.40E-05	0.000408212	8.2702579	9.1838928	0.151173483	ENSG00000196843	ARID5A
ENSG00000196843	E008	18.27082276	0.010620428	8.516295273	0.0035198	0.014668272	8.09390007	7.24006922	-0.160831547	ENSG00000196843	ARID5A
ENSG00000231856	E003	43.06548214	0.012299653	8.208047516	0.0041705	0.017076228	9.74745962	10.3292308	0.083634643	ENSG00000231856	RP11-327P2.5
ENSG00000231856	E004	4.555317146	0.050483241	7.943329414	0.0048265	0.019005769	3.14141963	4.05392012	0.367900991	ENSG00000231856	RP11-327P2.5
ENSG00000231856	E002	45.15270911	0.00800526	5.554424312	0.01843403	0.05926254	10.5300826	10.1223125	-0.056977841	ENSG00000231856	RP11-327P2.5
ENSG00000237054	E015	0.955987911	0.385434608	45.79277918	1.31E-11	2.92E-10	0.35079099	3.07461457	3.131721966	ENSG00000237054	PRMT5-AS1
ENSG00000237054	E016	0.876227769	0.422856859	38.1865453	6.43E-10	1.06E-08	0.4071397	2.97493491	2.86926228	ENSG00000237054	PRMT5-AS1
ENSG00000237054	E002	6.128597691	0.016775302	32.70672792	1.07E-08	1.32E-07	4.69043866	3.55458929	-0.400039977	ENSG00000237054	PRMT5-AS1
ENSG00000237054	E014	0.203113227	0.491949621	12.42001636	0.00042476	0.002212498	0.02900485	1.29900886	5.484973294	ENSG00000237054	PRMT5-AS1
ENSG00000237054	E001	2.248123008	0.029447204	7.101417326	0.0077023	0.028522759	2.8627992	2.20735063	-0.375110663	ENSG00000237054	PRMT5-AS1
ENSG00000241544	E003	0.26310301	0.120901971	15.26289691	9.35E-05	0.000557826	0.46482755	1.59290392	1.776891764	ENSG00000241544	RP11-6F2.5
ENSG00000241544	E001	3.05116085	0.613486614	14.2138393	0.00016317	0.000895238	3.25308746	2.23333438	-0.542610338	ENSG00000241544	RP11-6F2.5
ENSG00000241544	E002	0.136744599	1.091476863	5.380451879	0.0203636	0.064707511	0.42605775	1.13431185	1.412696415	ENSG00000241544	RP11-6F2.5
ENSG00000253837	E005	1.458954279	0.08986419	8.122440929	0.00437207	0.017638283	1.59898586	2.4393407	0.609334096	ENSG00000253837	RP11-177H13.2
ENSG00000258820	E006	0.230689132	0.548068616	25.0098186	5.70E-07	5.27E-06	0.22530692	1.88892927	3.067605159	ENSG00000258820	RP11-293M10.2
ENSG00000258820	E007	0.025452461	0.249104175	4.671423191	0.0306685	0.091782466	0.27866156	0.08180561	-1.768242224	ENSG00000258820	RP11-293M10.2
ENSG00000259687	E003	11.36580128	0.019533479	56.90663375	4.57E-14	1.34E-12	6.87710375	4.98955175	-0.462890897	ENSG00000259687	LINC01220
ENSG00000259687	E005	8.430576486	0.02510889	37.35406142	9.85E-10	1.47E-08	4.49264091	6.10384582	0.442154768	ENSG00000259687	LINC01220

ENSG00000259687	E004	3.360112932	0.06699958	15.09285424	0.00010235	0.000606	2.70658151	4.06428832	0.586530913	ENSG00000259687	LINC01220
ENSG00000259687	E001	0.68503901	0.117158986	6.631666088	0.01001815	0.03584756	1.17304114	2.0026355	0.771646245	ENSG00000259687	LINC01220
ENSG00000267270	E012	7.163756359	0.230090385	37.90615964	7.42E-10	1.15E-08	3.13321727	6.40387583	1.031300519	ENSG00000267270	PARD6G-AS1
ENSG00000267270	E002	7.073387719	0.222538281	36.74956463	1.34E-09	1.87E-08	3.02897418	6.35096658	1.068146899	ENSG00000267270	PARD6G-AS1
ENSG00000267270	E001	3.912798968	0.27758897	28.89633353	7.64E-08	8.49E-07	2.25325772	4.9136673	1.124787844	ENSG00000267270	PARD6G-AS1
ENSG00000267270	E003	6.270786417	0.191270821	27.97632942	1.23E-07	1.25E-06	3.1105488	5.81558425	0.902754999	ENSG00000267270	PARD6G-AS1
ENSG00000267270	E005	188.2493446	0.054572878	23.7953308	1.07E-06	9.09E-06	17.6223778	15.5180493	-0.183461382	ENSG00000267270	PARD6G-AS1
ENSG00000267270	E011	4.717020771	0.052038163	8.488958968	0.00357308	0.014777118	3.6712614	4.40291513	0.262183197	ENSG00000267270	PARD6G-AS1
ENSG00000270069	E001	54.50975163	0.059985576	8.511577391	0.00352894	0.014668272	10.9005924	11.880846	0.124231025	ENSG00000270069	MIR222HG
ENSG00000270069	E002	142.2145252	0.015236293	7.199059222	0.00729418	0.027163398	15.805402	15.3583679	-0.041392815	ENSG00000270069	MIR222HG
ENSG00000275993	E007	0.071387843	0.102543832	5.719315605	0.01677927	0.055908255	0.02420449	0.81474998	5.073010575	ENSG00000275993	CH507-42P11.8
ENSG00000277632	E002	18.2712058	0.006079455	17.37679147	3.07E-05	0.000212006	7.61497475	6.67374678	-0.190342306	ENSG00000277632	CCL3
ENSG00000277632	E005	4.103666068	0.169275675	13.23009393	0.00027549	0.001453386	2.83578413	4.62355811	0.705255807	ENSG00000277632	CCL3
ENSG00000277632	E006	16.01568511	0.133902705	8.727723262	0.00313408	0.013227408	6.1965716	7.87316814	0.345474056	ENSG00000277632	CCL3
ENSG00000277632	E001	3.480507888	0.033002449	8.325667004	0.00390888	0.016085034	3.73525175	2.70295447	-0.466668271	ENSG00000277632	CCL3
