# Supplementary figures and images for: A molecular signature for delayed graft function (part 2 of 2)
Source: Aging Cell. 2018 Aug 9;17(5):e12825. doi: 10.1111/acel.12825 (PMC6156499; doi:10.1111/acel.12825)

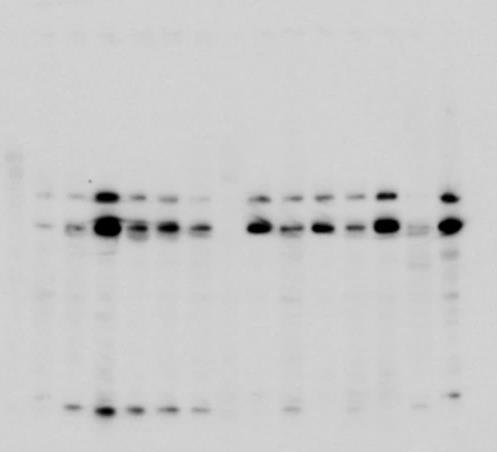

Supplement: Supplementary file 7 [file ACEL-17-e12825-s007.xps › Resources/Images/image_17.jpg]

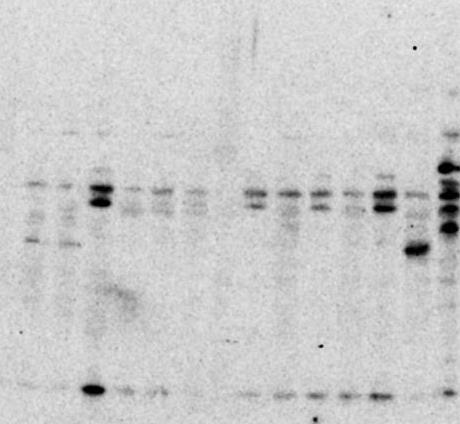

Supplement: Supplementary file 7 [file ACEL-17-e12825-s007.xps › Resources/Images/image_16.jpg]

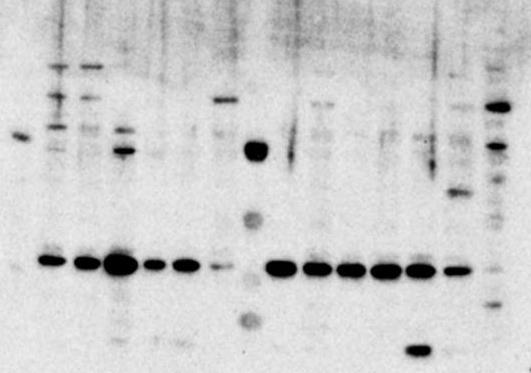

Supplement: Supplementary file 7 [file ACEL-17-e12825-s007.xps › Resources/Images/image_15.jpg]

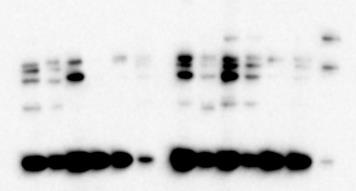

Supplement: Supplementary file 7 [file ACEL-17-e12825-s007.xps › Resources/Images/image_14.jpg]

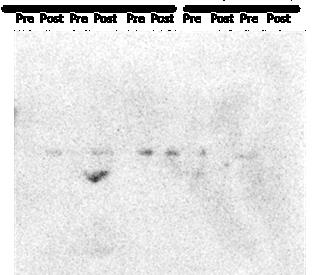

Supplement: Supplementary file 7 [file ACEL-17-e12825-s007.xps › Resources/Images/image_13.jpg]

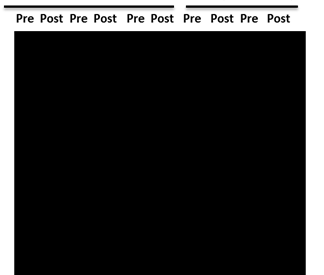

Supplement: Supplementary file 7 [file ACEL-17-e12825-s007.xps › Resources/Images/image_12.png]

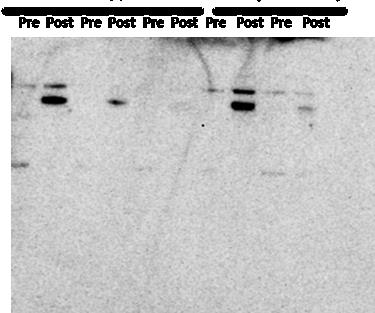

Supplement: Supplementary file 7 [file ACEL-17-e12825-s007.xps › Resources/Images/image_11.jpg]

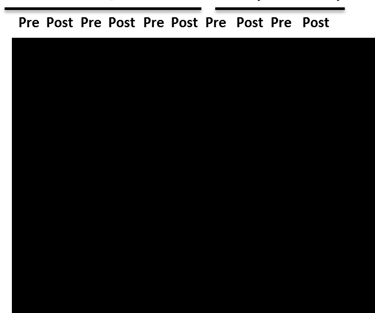

Supplement: Supplementary file 7 [file ACEL-17-e12825-s007.xps › Resources/Images/image_10.png]

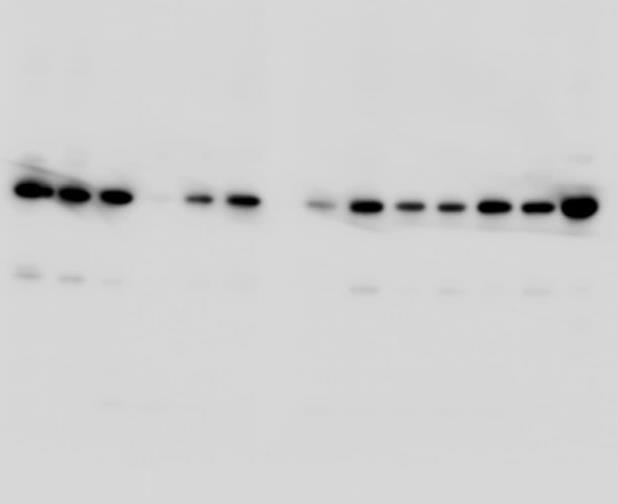

Supplement: Supplementary file 7 [file ACEL-17-e12825-s007.xps › Resources/Images/image_19.jpg]
