# Supplementary material for: A molecular signature for delayed graft function
Source: Aging Cell. 2018 Aug 9;17(5):e12825. doi: 10.1111/acel.12825 (PMC6156499; doi:10.1111/acel.12825)
Supplement: Supplementary file 8 [file ACEL-17-e12825-s008.pdf]

SD7 (Supplementary Data 7)

Number and biotype of detected transcripts in three models of analysis for DGF targets.

A Molecular signature for Delayed Graft Function

Dagmara McGuinness[1], Suhaib Mohammed[1], Laura Monaghan[1], Paul A. Wilson[2], David B. Kingsmore[3], Oliver Shapter[1,3], Karen S. Stevenson[3], Shana M. Coley[4], Luke Devey[5], Robert B. Kirkpatrick[6] and Paul G. Shiels[1]*

[1]University of Glasgow, College of Medical, Veterinary & Life Sciences, Wolfson Wohl Translational Research Centre, Institute of Cancer Sciences, Garscube Estate, Switchback Road, Glasgow, G61 1QH, Scotland

[2]Target Sciences Computational Biology Department, GlaxoSmithKline Medicines Research Centre, Gunnels Wood Road, Stevenage, Hertfordshire, SG1 2NY, UK

[3]NHS Greater Glasgow and Clyde, Renal Transplant Unit, Ward 4c, South Glasgow University Hospital, Glasgow, G51 4TF, Scotland

[4]University of Glasgow, College of Medical, Veterinary & Life Sciences, Research Institute of Infection Immunity and Inflammation, 120 University Place, Glasgow, G12 8TA, Scotland

[5]Metabolic Pathways Cardio Therapy Area Unit, GlaxoSmithKline, 709 Swedeland Road, King of Prussia, PA, USA

[6]The Pipeline Futures Group, GlaxoSmithKline, 1250 South Collegeville Road, Collegeville, PA, USA

Corresponding author: Prof Paul G Shiels

University of Glasgow, Wolfson Wohl Translational Research Centre, Institute of Cancer Sciences, Garscube Estate, Switchback Road, Glasgow, G61 1QH

E-mail: paul.shiels@glasgow.ac.uk

S7Table 1.Number and biotype of detected transcripts in 3 models of analysis for DGF targets. DGF-delayed graft function, IGF-immediate graft function

Transcript biotype	Model 1 (TOM4)	Model 2		Model 3	
	DGF signature independent of perfusion state	IGF (pre vs. post perfusion)	DGF (pre vs post perfusion)	DGF vs. IGF pre-perfusion	DGF vs. IGF post-perfusion
3prime_overlapping_ncrna	-	-	2	-	-
antisense	2	140	339	-	-
IG_V_gene	-	2	6	-	-
IG_V_pseudogene	-	-	1	-	-
Pseudogene		1	3		
lincRNA	2	160	320	-	-
miRNA	-	72	103	-	-
misc_RNA	1	42	42	-	-
Mt_tRNA	-	-	3	-	-
nonsense_mediated_decay	2	20	44	-	-
processed_pseudogene	1	71	150	-	-
processed_transcript	1	51	128	-	-
protein_coding	22	429	906	0	1
retained_intron	4	29	57	-	-
rRNA	1	2	3	-	-
sense_intronic	1	54	68	-	-
sense_overlapping	-	6	13	-	-
snoRNA	-	29	46	-	-
snRNA	-	22	29	-	-
sca-RNA	-	1	-	-	-
sRNA	-	1	-	-	-
TEC	-	33	65	-	-
TR_J_gene	-	-	1	-	-
transcribed_processed_pseudogene		5	11	-	-
transcribed_unprocessed_pseudogene	-	-	3	-	-
unitary_pseudogene	-	2	3	-	-
unprocessed_pseudogene	-	10	24	-	-
TR_C_ gene	-	1	-	-	-
