# Supplementary material for: A molecular signature for delayed graft function
Source: Aging Cell. 2018 Aug 9;17(5):e12825. doi: 10.1111/acel.12825 (PMC6156499; doi:10.1111/acel.12825)
Supplement: Supplementary file 9 [file ACEL-17-e12825-s009.pdf]

SD8 (Supplementary Data 8) TaqMan® and UPL® gene expression assay list.

Target ID	Assay ID/ Primers sequences	UPL probe	Manufacturer
**ABCA8**	Hs00992371_m1	n/a	Life Technologies
**BTN3A2**	Hs00389328_m1	n/a	Life Technologies
**C1QB**	Hs00608019_m1	n/a	Life Technologies
**CCL19**	Hs00171149_m1	n/a	Life Technologies
**CD52**	Hs00174349_m1	n/a	Life Technologies
**CD69**	Hs00934033_m1	n/a	Life Technologies
**CHGB**	Hs01084631_m1	n/a	Life Technologies
**CORIN**	Hs00198141_m1	n/a	Life Technologies
**CXCL10**	Hs01124252_g1	n/a	Life Technologies
**CXCL11**	Hs04187682_g1	n/a	Life Technologies
**CXCL9**	Hs00171065_m1	n/a	Life Technologies
**FAM43A**	Hs00538124_s1	n/a	Life Technologies
**FCGR2B**	Hs01634996_s1	n/a	Life Technologies
**FCGR3B**	Hs04334165_m1	n/a	Life Technologies
**FCRL3**	Hs00364720_m1	n/a	Life Technologies
**IFNG**	Hs00989291_m1	n/a	Life Technologies
**KLRB1**	Hs00174469_m1	n/a	Life Technologies
**MNDA**	Hs00935905_m1	n/a	Life Technologies
**NLRC4**	Hs00892666_m1	n/a	Life Technologies
**PTPRC**	Hs04189704_m1	n/a	Life Technologies
**REG1B**	Hs01888695_s1	n/a	Life Technologies
**RSPO1**	Hs00543475_m1	n/a	Life Technologies
**TAGAP**	Hs00299284_m1	n/a	Life Technologies
**TRIM15**	Hs00264400_m1	n/a	Life Technologies
**UBD**	Hs00197374_m1	n/a	Life Technologies
**18S**	Hs99999901_s1	n/a	Life Technologies
**HPRT1**	Hs02800695_m1	n/a	Life Technologies
**MMP9**	Hs00234579_m1	n/a	Life Technologies
**ACKR3**	Hs00664172_s1	n/a	Life Technologies
**FLG**	Hs00856927_g1	n/a	Life Technologies
**HIST1H3I**	Hs00605800_s1	n/a	Life Technologies
**LRRC10B**	Hs03805919_s1	n/a	Life Technologies
**NLRP2**	Hs01546932_m1	n/a	Life Technologies
**NLRP7**	Hs00373683_m1	n/a	Life Technologies
**S100A2**	Hs00195582_m1	n/a	Life Technologies
**SEMA3A**	Hs00173810_m1	n/a	Life Technologies
**ZNF676**	Hs01939480_s1	n/a	Life Technologies
**GABBR1**	Hs00559488_m1	n/a	Life Technologies
**DISP2**	Hs00394338_m1	n/a	Life Technologies
**GRIN3B**	Hs00879908_m1	n/a	Life Technologies
**ISG20**	Hs00158122_m1	n/a	Life Technologies
**OAS2**	Hs00942643_m1	n/a	Life Technologies
**CDKN2A/p16**	Hs01034249_m1	n/a	Life Technologies

**ARF/p14**	Hs00923894_m1	n/a	Life Technologies
**CDKN2B**	Hs00793225_m1	n/a	Life Technologies
**CDKN1A**	Hs00355782_m1	n/a	Life Technologies
**CDKN1B**	Hs00153277_m1	n/a	Life Technologies
**FCGR1C**	5'-GGCAAGGGAAAGCATCACTA-3'	#18	Roche
	5'-CAGCACTGGAGCTGGAAATA-3'		
**FCGR2C**	5'-AGCGGATTTCAGCCAATTC-3'	#44	Roche
	5'-CTGATGGCAATCATTTGACG-3'		
**IDO1-009**	5'-CTGGGCATCCAGCAGACT-3'	#65	Roche
	5'-GGAAGTTCCTGTGAGCTGGT-3'		
**REG1A**	5'-CAGACCAGCTCATACTTCATGC-3'	#51	Roche
	5'-ATAGGCATTGGTGCCTTCTG-3'		
**HPRT1**	5'-TGACCTTGATTTATTTTGCATACC-3'	#73	Roche
	5'-CGAGCAAGACGTTCAGTCCT-3'		
**18S RNA**	5'-CTCAACACGGGAAACCTCAC-3'	#77	Roche
	5'-CGCTCCACCAACTAAGAACG-3'		
